# Incident type 2 diabetes attributable to suboptimal diet in 184 countries

**DOI:** 10.1038/s41591-023-02278-8

**Published:** 2023-04-17

**Authors:** Meghan O’Hearn, Laura Lara-Castor, Frederick Cudhea, Victoria Miller, Julia Reedy, Peilin Shi, Jianyi Zhang, John B. Wong, Christina D. Economos, Renata Micha, Dariush Mozaffarian, Global Dietary Database, Murat Bas, Jemal Haidar Ali, Suhad Abumweis, Anand Krishnan, Puneet Misra, Nahla Chawkat Hwalla, Chandrashekar Janakiram, Nur Indrawaty Liputo, Abdulrahman Musaiger, Farhad Pourfarzi, Iftikhar Alam, Karin DeRidder, Celine Termote, Anjum Memon, Aida Turrini, Elisabetta Lupotto, Raffaela Piccinelli, Stefania Sette, Karim Anzid, Marieke Vossenaar, Paramita Mazumdar, Ingrid Rached, Alicia Rovirosa, María Elisa Zapata, Tamene Taye Asayehu, Francis Oduor, Julia Boedecker, Lilian Aluso, Johana Ortiz-Ulloa, J. V. Meenakshi, Michelle Castro, Giuseppe Grosso, Anna Waskiewicz, Umber S. Khan, Anastasia Thanopoulou, Reza Malekzadeh, Neville Calleja, Marga Ocke, Zohreh Etemad, Mohannad Al Nsour, Lydiah M. Waswa, Eha Nurk, Joanne Arsenault, Patricio Lopez-Jaramillo, Abla Mehio Sibai, Albertino Damasceno, Carukshi Arambepola, Carla Lopes, Milton Severo, Nuno Lunet, Duarte Torres, Heli Tapanainen, Jaana Lindstrom, Suvi Virtanen, Cristina Palacios, Eva Roos, Imelda Angeles Agdeppa, Josie Desnacido, Mario Capanzana, Anoop Misra, Ilse Khouw, Swee Ai Ng, Edna Gamboa Delgado, Mauricio Caballero, Johanna Otero, Hae-Jeung Lee, Eda Koksal, Idris Guessous, Carl Lachat, Stefaan De Henauw, Ali Reza Rahbar, Alison Tedstone, Androniki Naska, Angie Mathee, Annie Ling, Bemnet Tedla, Beth Hopping, Brahmam Ginnela, Catherine Leclercq, Charmaine Duante, Christian Haerpfer, Christine Hotz, Christos Pitsavos, Colin Rehm, Coline van Oosterhout, Corazon Cerdena, Debbie Bradshaw, Dimitrios Trichopoulos, Dorothy Gauci, Dulitha Fernando, Elzbieta Sygnowska, Erkki Vartiainen, Farshad Farzadfar, Gabor Zajkas, Gillian Swan, Guansheng Ma, Gulden Pekcan, Hajah Masni Ibrahim, Harri Sinkko, Helene Enghardt Barbieri, Isabelle Sioen, Jannicke Myhre, Jean-Michel Gaspoz, Jillian Odenkirk, Kanitta Bundhamcharoen, Keiu Nelis, Khairul Zarina, Lajos Biro, Lars Johansson, Laufey Steingrimsdottir, Leanne Riley, Mabel Yap, Manami Inoue, Maria Szabo, Marja-Leena Ovaskainen, Meei-Shyuan Lee, Mei Fen Chan, Melanie Cowan, Mirnalini Kandiah, Ola Kally, Olof Jonsdottir, Pam Palmer, Peter Vollenweider, Philippos Orfanos, Renzo Asciak, Robert Templeton, Rokiah Don, Roseyati Yaakub, Rusidah Selamat, Safiah Yusof, Sameer Al-Zenki, Shu-Yi Hung, Sigrid Beer-Borst, Suh Wu, Widjaja Lukito, Wilbur Hadden, Wulf Becker, Xia Cao, Yi Ma, Yuen Lai, Zaiton Hjdaud, Jennifer Ali, Ron Gravel, Tina Tao, Jacob Lennert Veerman, Shashi Chiplonkar, Mustafa Arici, Le Tran Ngoan, Demosthenes Panagiotakos, Yanping Li, Antonia Trichopoulou, Noel Barengo, Anuradha Khadilkar, Veena Ekbote, Noushin Mohammadifard, Irina Kovalskys, Avula Laxmaiah, Harikumar Rachakulla, Hemalatha Rajkumar, Indrapal Meshram, Laxmaiah Avula, Nimmathota Arlappa, Rajkumar Hemalatha, Licia lacoviello, Marialaura Bonaccio, Simona Costanzo, Yves Martin-Prevel, Katia Castetbon, Nattinee Jitnarin, Yao-Te Hsieh, Sonia Olivares, Gabriela Tejeda, Aida Hadziomeragic, Amanda de Moura Souza, Wen-Harn Pan, Inge Huybrechts, Alan de Brauw, Mourad Moursi, Maryam Maghroun, Augustin Nawidimbasba Zeba, Nizal Sarrafzadegan, Lital Keinan-Boker, Rebecca Goldsmith, Tal Shimony, Irmgard Jordan, Shivanand C. Mastiholi, Moses Mwangi, Yeri Kombe, Zipporah Bukania, Eman Alissa, Nasser Al-Daghri, Shaun Sabico, Martin Gulliford, Tshilenge S. Diba, Kyungwon Oh, Sanghui Kweon, Sihyun Park, Yoonsu Cho, Suad Al-Hooti, Chanthaly Luangphaxay, Daovieng Douangvichit, Latsamy Siengsounthone, Pedro Marques-Vidal, Constance Rybak, Amy Luke, Noppawan Piaseu, Nipa Rojroongwasinkul, Kalyana Sundram, Donka Baykova, Parvin Abedi, Sandjaja Sandjaja, Fariza Fadzil, Noriklil Bukhary Ismail Bukhary, Pascal Bovet, Yu Chen, Norie Sawada, Shoichiro Tsugane, Lalka Rangelova, Stefka Petrova, Vesselka Duleva, Anna Karin Lindroos, Jessica Petrelius Sipinen, Lotta Moraeus, Per Bergman, Ward Siamusantu, Lucjan Szponar, Hsing-Yi Chang, Makiko Sekiyama, Khanh Le Nguyen Bao, Balakrishna Nagalla, Kalpagam Polasa, Sesikeran Boindala, Jalila El Ati, Ivonne Ramirez Silva, Juan Rivera Dommarco, Simon Barquera, Sonia Rodríguez-Ramírez, Daniel Illescas-Zarate, Luz Maria Sanchez-Romero, Nayu Ikeda, Sahar Zaghloul, Anahita Houshiar-rad, Fatemeh Mohammadi-Nasrabadi, Morteza Abdollahi, Khun-Aik Chuah, Zaleha Abdullah Mahdy, Alison Eldridge, Eric L. Ding, Herculina Kruger, Sigrun Henjum, Anne Fernandez, Milton Fabian Suarez-Ortegon, Nawal Al-Hamad, Veronika Janská, Reema Tayyem, Parvin Mirmiran, Roya Kelishadi, Eva Warensjo Lemming, Almut Richter, Gert Mensink, Lothar Wieler, Daniel Hoffman, Benoit Salanave, Cho-il Kim, Rebecca Kuriyan-Raj, Sumathi Swaminathan, Didier Garriguet, Saeed Dastgiri, Sirje Vaask, Tilakavati Karupaiah, Fatemeh Vida Zohoori, Alireza Esteghamati, Maryam Hashemian, Sina Noshad, Elizabeth Mwaniki, Elizabeth Yakes-Jimenez, Justin Chileshe, Sydney Mwanza, Lydia Lera Marques, Alan Martin Preston, Samuel Duran Aguero, Mariana Oleas, Luz Posada, Angelica Ochoa, Khadijah Shamsuddin, Zalilah Mohd Shariff, Hamid Jan Bin Jan Mohamed, Wan Manan, Anca Nicolau, Cornelia Tudorie, Bee Koon Poh, Pamela Abbott, Mohammadreza Pakseresht, Sangita Sharma, Tor Strand, Ute Alexy, Ute Nöthlings, Jan Carmikle, Ken Brown, Jeremy Koster, Indu Waidyatilaka, Pulani Lanerolle, Ranil Jayawardena, Julie M. Long, K. Michael Hambidge, Nancy F. Krebs, Aminul Haque, Gudrun B. Keding, Liisa Korkalo, Maijaliisa Erkkola, Riitta Freese, Laila Eleraky, Wolfgang Stuetz, Inga Thorsdottir, Ingibjorg Gunnarsdottir, Lluis Serra-Majem, Foong Ming Moy, Simon Anderson, Rajesh Jeewon, Corina Aurelia Zugravu, Linda Adair, Shu Wen Ng, Sheila Skeaff, Dirce Marchioni, Regina Fisberg, Carol Henry, Getahun Ersino, Gordon Zello, Alexa Meyer, Ibrahim Elmadfa, Claudette Mitchell, David Balfour, Johanna M. Geleijnse, Mark Manary, Tatyana El-kour, Laetitia Nikiema, Masoud Mirzaei, Rubina Hakeem

**Affiliations:** 1 Friedman School of Nutrition Science and Policy, Tufts University, Boston, MA USA; 2 Food Systems for the Future Institute, Chicago, IL USA; 3 Department of Medicine, McMaster University, Hamilton, Ontario Canada; 4 Population Health Research Institute, Hamilton, Ontario Canada; 5 Tufts University School of Medicine, Boston, MA USA; 6 Department of Medicine, Tufts Medical Center, Boston, MA USA; 7 Department of Food Science and Nutrition, University of Thessaly, Volos, Greece; 8 Acibadem University, Istanbul, Turkey; 9 Addis Ababa University, Addis Ababa, Ethiopia; 10 Al Ain University, Abu Dhabi, UAE; 11 All India Institute of Medical Sciences, New Delhi, India; 12 American University of Beirut, Beirut, Lebanon; 13 Amrita School of Dentistry, Eranakulum, India; 14 Andalas University, Padang, Indonesia; 15 Arab Center for Nutrition, Manama, Bahrain; 16 Ardabil University of Medical Sciences, Ardabil, Iran; 17 Bacha Khan University, Charsadda, Pakistan; 18 Belgian Public Health Institute, Brussels, Belgium; 19 Biodiversity International, Maccarese, Italy; 20 Brighton and Sussex Medical School, Brighton, UK; 21 CREA—Alimenti e Nutrizione, Rome, Italy; 22 Cadi Ayyad University, Benguerir, Morocco; 23 Center for Studies of Sensory Impairment, Aging and Metabolism (CeSSIAM), Guatemala City, Guatemala; 24 Centre for Media Studies, New Delhi, India; 25 Centro de Atencion Nutricional Antimano (CANIA), Miami, FL USA; 26 Centro de Estudios sobre Nutrición Infantil (CESNI), Buenos Aires, Argentina; 27 College of Applied Sciences, Department of Food Science and Applied Nutrition, Addis Ababa Science and Technology University, Addis Ababa, Ethiopia; 28 Consultative Group on International Agricultural Research (CGIAR), Montpellier, France; 29 Cuenca University, Cuenca, Ecuador; 30 Delhi School of Economics, University of Delhi, Delhi, India; 31 Departamento de Alimentacao Escolar, São Paulo, Brazil; 32 Department of Biomedical and Biotechnological Sciences, University of Catania, Catania, Italy; 33 Department of CVD Epidemiology, Prevention and Health Promotion, Institute of Cardiology, Warsaw, Poland; 34 Department of Community Health Sciences, Aga Khan University, Karachi, Pakistan; 35 Diabetes Center, 2nd Department of Internal Medicine, Athens University, Athens, Greece; 36 Digestive Disease Research Institute, Tehran University of Medical Sciences, Tehran, Iran; 37 Directorate for Health Information and Research, Tarxien, Malta; 38 Dutch National Institute for Public Health and the Environment (RIVM), Bilthoven, the Netherlands; 39 Eastern Mediterranean Public Health Network (EMPHNET), Amman, Jordan; 40 Egerton University, Njoro, Kenya; 41 Estonian National Institute for Health Development, Tallinn, Estonia; 42 FHI Solutions, Washington, DC USA; 43 FOSCAL and UDES, Bucaramanga, Colombia; 44 Faculty of Health Sciences, American University of Beirut, Beirut, Lebanon; 45 Faculty of Medicine, Eduardo Mondlane University, Maputo, Mozambique; 46 Faculty of Medicine, University of Colombo, Sri Lanka, Colombo 5, Sri Lanka; 47 Faculty of Medicine/Institute of Public Health, University of Porto, Porto, Portugal; 48 Faculty of Nutrition and Food Sciences, University of Porto, Porto, Portugal; 49 Finnish Institute for Health and Welfare, Helsinki, Finland; 50 Florida International University, Miami, FL USA; 51 Folkhälsan Research Center, Helsinki, Finland; 52 Food and Nutrition Research Institute (DOST-FNRI), Manila, Philippines; 53 Department of Science and Technology, Food and Nutrition Research Institute, Taguig City, Philippines; 54 Fortis CDOC Center for Excellence for Diabetes, New Delhi, India; 55 FrieslandCampina, Amersfoort, the Netherlands; 56 Fundacion Cardiovascular de Colombia, Bucaramanga, Colombia; 57 Fundacion INFANT and Consejo Nacional de Investigaciones Cientificas y Tecnicas (CONICET), Buenos Aires, Argentina; 58 Fundacion Oftalmologica de Santander (FOSCAL), Floridablanca, Colombia; 59 Gachon University, Seongnam-si, South Korea; 60 Gazi University, Ankara, Turkey; 61 Geneva University Hospitals, Geneva, Switzerland; 62 Ghent University, Ghent, Belgium; 63 Global Dietary Database Consortium, Boston, MA USA; 64 Government of Canada, Statistics Canada, Ottawa, Ontario Canada; 65 Griffith University, Gold Coast, Queensland Australia; 66 HC Jehangir Medical Research Institute, Pune, India; 67 Hacettepe University Faculty of Medicine, Ankara, Turkey; 68 Hanoi Medical University, Hanoi, Vietnam; 69 Harokopio University, Athens, Greece; 70 Harvard School of Public Health, Cambridge, MA USA; 71 Hellenic Health Foundation and University of Athens, Athens, Greece; 72 Herbert Wetheim College of Medicine, Miami, FL USA; 73 Hirabai Cowasji Jehangir Medical Research Institute, Pune, India; 74 Hypertension Research Center, Cardiovascular Research Center, Isfahan University of Medical Sciences, Isfahan, Iran; 75 Instituto para la Cooperacion Científica en Ambiente y Salud (ICCAS), Buenos Aires, Argentina; 76 ICMR—National Institute of Nutrition, Hyderabad, India; 77 IRCCS Neuromed, Pozzilli, Italy; 78 University of Insubria, Varese, Italy; 79 Institut de Recherche pour le Developpement, Montepellier, France; 80 Institut de Veille Sanitaire, Bobigny, France; 81 Institute for International Investigation, NDRI-USA, New York, NY USA; 82 Institute of Biomedical Sciences, Academia Sinica, Taipei, Taiwan; 83 Institute of Nutrition and Food Technology (INTA), University of Chile, Santiago, Chile; 84 Institute of Nutrition in Central America and Panama (INCAP), Guatemala City, Guatemala; 85 Institute of Public Health of Federation of Bosnia and Herzegovina, Sarajevo, Bosnia and Herzegovina; 86 Institute of Studies in Public Health, Federal University of Rio de Janeiro (UFRJ), Rio de Janeiro, Brazil; 87 International Agency for Research on Cancer, Lyon, France; 88 International Food Policy Research Institute (IFPRI), Washington, DC USA; 89 Interventional Cardiology Research Center, Cardiovascular Research Center, Isfahan University of Medical Sciences, Isfahan, Iran; 90 Institut de Recherche en Sciences de la Sante, Bobo Dioulasso, Burkina Faso; 91 Isfahan Cardiovascular Research Center, Cardiovascular Research Center, Isfahan University of Medical Sciences, Isfahan, Iran; 92 Israel Center for Disease Control, Tel-Hashomer, Israel; 93 Justus Liebig University Giessen, Giessen, Germany; 94 Jawaharlal Nehru Medical College, KLE Academy of Higher Education and Research, Belagavi, India; 95 Kenya Medical Research Institute, Nairobi, Kenya; 96 King Abdulaziz University, Jeddah, Saudi Arabia; 97 King Saud University, Riyadh, Saudi Arabia; 98 King’s College London, London, UK; 99 Kinshasa School of Public Health, Kinshasa, Democratic Republic of Congo; 100 Korea Disease Control and Prevention Agency (KDCA), Seoul, Korea; 101 Korea University, Seoul, Korea; 102 Kuwait Institute for Scientific Research, Safat, Kuwait; 103 Lao Tropical and Public Health Institute, Vientiane, Lao PDR; 104 Lausanne University Hospital (CHUV) and University of Lausanne, Lausanne, Switzerland; 105 Leibniz Centre for Agricultural Landscape Research, Muncheberg, Germany; 106 Loyola University Chicago, Chicago, IL USA; 107 Mahidol University, Bangkok, Thailand; 108 Mahidol University, Pathom, Thailand; 109 Malaysian Palm Oil Council (MPOC), Kelana Jaya, Malaysia; 110 Medical Center Markovs, Sofia, Bulgaria; 111 Menopause Andropause Research Center, Ahvaz Jundishapur University of Medical Sciences, Ahvaz, Iran; 112 Ministry of Health, Jakarta, Indonesia; 113 Ministry of Health, Kuala Lumpur, Malaysia; 114 Ministry of Health, Sungai Besar, Malaysia; 115 Ministry of Health, Victoria, Seychelles; 116 University Center for Primary Care and Public Health (Unisanté), Lausanne, Switzerland; 117 NYU School of Medicine, New York, NY USA; 118 National Cancer Center Institute for Cancer Control, Tokyo, Japan; 119 National Centre of Public Health and Analyses (NCPHA), Sofia, Bulgaria; 120 National Food Agency, Uppsala, Sweden; 121 National Food and Nutrition Commission, Lusaka, Zambia; 122 National Food and Nutrition Institute, Warsaw, Poland; 123 National Health Research Institutes, Zhunan, Taiwan; 124 Health and Environmental Risk Division, National Institute for Environmental Studies, Tsukuba, Japan; 125 National Institute of Nutrition, Hanoi, Vietnam; 126 National Institute of Nutrition, Hyderabad, India; 127 National Institute of Nutrition and Food Technology & SURVEN RL, Tunis, Tunisia; 128 National Institute of Public Health (INSP), Cuernavaca, Mexico; 129 National Institute of Public Health (INSP), Mexico City, Mexico; 130 National Institutes of Biomedical Innovation, Health and Nutrition, Tokyo, Japan; 131 National Nutrition Institute, Cairo, Egypt; 132 National Nutrition and Food Technology Research Institute (NNFTRI): SBMU, Tehran, Iran; 133 National University of Malaysia (UKM), Kuala Lumpur, Malaysia; 134 Nestlé Research, Lausanne, Switzerland; 135 New England Complex Systems Institute, Cambridge, MA USA; 136 North-West University, Potchefstroom South Africa, Potchefstroom, South Africa; 137 Oslo Metropolitan University (OsloMet), Oslo, Norway; 138 Perdana University and Royal College of Surgeons in Ireland, Puchong, Malaysia; 139 Pontificia Universidad Javeriana Seccional Cali, Cali, Colombia; 140 Public Authority for Food and Nutrition, Sabah Al Salem, Kuwait; 141 Public Health Authority of the Slovak Republic, Bratislava, Slovak Republic; 142 Qatar University and University of Jordan, Doha, Qatar; 143 Research Institute for Endocrine Sciences, Shahid Beheshti University of Medical Sciences, Tehran, Iran; 144 Research Institute for Primordial Prevention of NCD, Isfahan University of Medical Sciences, Isfahan, Iran; 145 Risk and Benefit Assessment Department, Swedish Food Agency, Uppsala, Sweden; 146 Robert Koch Institute, Berlin, Germany; 147 Rutgers University, New Brunswick, NJ USA; 148 Santé publique France, the French Public Health Agency, Saint Maurice, France; 149 Seoul National University, Seoul, Korea; 150 St John’s Research Institute, Bangalore, India; 151 Statistics Canada, Ottawa, Ontario Canada; 152 Tabriz University of Medical Sciences, Tabriz, Iran; 153 Tallinn University, Tallinn, Estonia; 154 Taylor’s University, Selangor, Malaysia; 155 Teesside University, Middlesbrough, UK; 156 Tehran University of Medical Sciences, Tehran, Iran; 157 Utica University, Tehran, Iran; 158 The Technical University of Kenya, Nairobi, Kenya; 159 The University of New Mexico, Albuquerque, NM USA; 160 Tropical Diseases Research Centre, Ndola, Zambia; 161 Unidad de Nutricion Publica, Macul, Chile; 162 Department of Biochemistry, University of Puerto Rico, Medical Sciences Campus, San Juan, Puerto Rico; 163 Universidad San Sebastian, Providencia, Chile; 164 Universidad Tecnica del Norte, Ibarra, Ecuador; 165 Universidad de Antioquia, Medellín, Colombia; 166 Universidad de Cuenca, Cuenca, Ecuador; 167 Universiti Kebangsaan Malaysia Medical Centre, Kuala Lumpur, Malaysia; 168 Universiti Putra Malaysia, Serdang, Malaysia; 169 Universiti Sains Malaysia, Kubang Kerian, Malaysia; 170 University Dunarea de Jos, Galati, Romania; 171 University Kebangsaan Malaysia, Selangor, Malaysia; 172 University of Aberdeen, Aberdeen, UK; 173 University of Alberta, Edmonton, Alberta Canada; 174 University of Bergen, Bergen, Norway; 175 Department of Nutrition and Food Sciences, University of Bonn, Bonn, Germany; 176 University of California Davis, Davis, CA USA; 177 University of Cincinnati, Cincinnati, OH USA; 178 University of Colombo, Colombo, Sri Lanka; 179 University of Colorado School of Medicine, Aurora, CO USA; 180 University of Dhaka, Dhaka, Bangladesh; 181 University of Goettingen, Goettingen, Germany; 182 Department of Food and Nutrition, University of Helsinki, Helsinki, Finland; 183 University of Hohenheim, Stuttgart, Germany; 184 University of Iceland, Reykjavík, Iceland; 185 University of Las Palmas de Gran Canaria (ULPGC), Canary Islands, Las Palmas, Spain; 186 University of Malaya, Kuala Lumpur, Malaysia; 187 University of Manchester, Manchester, UK; 188 University of Mauritius, Reduit, Mauritius; 189 University of Medicine and Pharmacy Carol Davila, Bucharest, Romania; 190 University of North Carolina at Chapel Hill, Chapel Hill, NC USA; 191 University of Otago, Dunedin, New Zealand; 192 University of São Paulo, São Paulo, Brazil; 193 University of Saskatchewan, Saskatoon, Saskatchewan Canada; 194 University of Vienna, Vienna, Austria; 195 University of the Southern Caribbean, Port of Spain, Trinidad and Tobago; 196 Wageningen University, Wageningen, the Netherlands; 197 Washington University in St. Louis, St. Louis, MO USA; 198 World Health Organization (WHO), Amman, Jordan; 199 World Health Organization (WHO), Geneva, Switzerland; 200 Yazd Cardiovascular Research Centre, Shahid Sadoughi University of Medical Sciences, Yazd, Iran; 201 Ziauddin University Karachi, Karachi, Pakistan

**Keywords:** Risk factors, Type 2 diabetes, Obesity

## Abstract

The global burden of diet-attributable type 2 diabetes (T2D) is not well established. This risk assessment model estimated T2D incidence among adults attributable to direct and body weight-mediated effects of 11 dietary factors in 184 countries in 1990 and 2018. In 2018, suboptimal intake of these dietary factors was estimated to be attributable to 14.1 million (95% uncertainty interval (UI), 13.8–14.4 million) incident T2D cases, representing 70.3% (68.8–71.8%) of new cases globally. Largest T2D burdens were attributable to insufficient whole-grain intake (26.1% (25.0–27.1%)), excess refined rice and wheat intake (24.6% (22.3–27.2%)) and excess processed meat intake (20.3% (18.3–23.5%)). Across regions, highest proportional burdens were in central and eastern Europe and central Asia (85.6% (83.4–87.7%)) and Latin America and the Caribbean (81.8% (80.1–83.4%)); and lowest proportional burdens were in South Asia (55.4% (52.1–60.7%)). Proportions of diet-attributable T2D were generally larger in men than in women and were inversely correlated with age. Diet-attributable T2D was generally larger among urban versus rural residents and higher versus lower educated individuals, except in high-income countries, central and eastern Europe and central Asia, where burdens were larger in rural residents and in lower educated individuals. Compared with 1990, global diet-attributable T2D increased by 2.6 absolute percentage points (8.6 million more cases) in 2018, with variation in these trends by world region and dietary factor. These findings inform nutritional priorities and clinical and public health planning to improve dietary quality and reduce T2D globally.

## Abstract (web summary)

Modeling analysis from the Global Dietary Database estimated that 70% of new global cases of type 2 diabetes are attributable to suboptimal intake of 11 dietary factors, with substantial differences in dietary risks across world regions and nations.

## Main

T2D is a leading determinant of morbidity and mortality globally, with enormous economic and societal consequences (http://www.diabetesatlas.org)^1,2^. Between 1980 and 2020–2021, the number of adults with diabetes (90% of which is T2D) increased from 108 million to 537 million, with corresponding increases in obesity from 100 million to 764 million adults (http://www.diabetesatlas.org)^3–5^. This phenomenon is global: no nation has experienced a decline in diabetes or obesity in the last 40 years (http://www.diabetesatlas.org). Diabetes creates extraordinary burdens on individuals, families, nations and healthcare systems, causing one in eight global deaths and increasing risk of cardiovascular diseases, renal decline, fatty liver disease, blindness, cancers, coronavirus disease 2019 and other infectious diseases (http://www.diabetesatlas.org). Left unchecked and with prevalence only projected to rise (http://www.diabetesatlas.org), T2D will decimate population health, economic productivity and health system capacity worldwide.

Several dietary factors have strong evidence for etiologic effects on incident T2D, either directly (for example, through changes in blood glucose levels, insulin resistance, hepatic steatosis, inflammation, the gut microbiome or other pathways that are independent of body mass index (BMI)) or mediated through weight gain (http://www.diabetesatlas.org)^2^. This includes, for example, direct and BMI-associated relationships associated with high intake of sugar-sweetened beverages (SSBs) and processed meats and low consumption of whole grains and yogurt, as well as BMI-associated relationships with low consumption of nuts and seeds and fruits.

Yet, while it is clear that diet plays an outsized role in the risk of T2D, the absolute and relative contributions of specific dietary factors to global incidence of T2D remain unclear. Previous analyses of disease burdens were focused on isolated dietary factors (such as SSBs in 2010)^6^ or in specific countries^7–9^ or world regions^10,11^. An analysis assessing diabetes globally suggested that dietary risks were responsible for 24.7% of diabetes deaths and 34.9% of diabetes disability-adjusted life years (DALYs), with heterogeneity by World Bank country income level^1^. This analysis used estimates of global diet based largely on Food and Agriculture Organization (FAO) food-balance sheets, rather than individual-level intakes, and did not incorporate updated assessments of dietary factors and both direct and weight-gain-mediated effects. In addition, the global burden of diet-related T2D according to differences in educational attainment or urban or rural residence within world regions or nations, factors known to influence both diet and T2D risk in region-specific ways, has yet to be determined. Such assessment is crucial to further elucidate dietary and health disparities by these factors within world regions and nations.

To address these gaps in knowledge and estimate the global effects of suboptimal diet on T2D, we conducted a comparative risk-assessment model to estimate the impact of 11 dietary factors, separately and jointly, on the absolute and proportional burdens of new T2D cases among adults globally and by age, sex, education, urbanicity, world region and nation, in 1990 and 2018.

## Results

### Datasets

**Extended Data Fig. 1:**
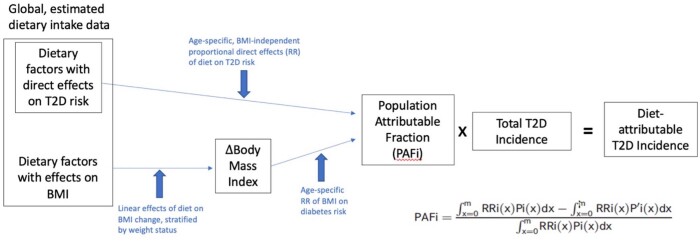
Logic pathway for estimating the direct and BMI-mediated diet-attributable T2D burden due to suboptimal diet globally. Modeling inputs and outputs provided in boxes. Blue text and arrows suggest the logical pathway through which dietary intake impacts T2D incidence. BMI, body mass index; PAF, population attributable fraction; RR, relative risk; T2D, type II diabetes.

We incorporated dietary data from the Global Dietary Database (GDD), population demographics from the United Nations, adiposity and diabetes distributions from the NCD Risk Factor Collaboration (NCD-RisC) and the Global Burden of Disease study, direct and BMI-mediated etiologic effects of dietary factors on T2D from pooled multivariable-adjusted analyses and optimal dietary intakes from published sources into a comparative risk-assessment-modeling framework to estimate the impact of 11 dietary factors, separately and jointly, on the absolute and proportional burdens of new T2D cases globally (Extended Data Fig. 1). See Methods for further details.

### Dietary and T2D distributions

Eleven dietary factors were identified to have probable or convincing evidence of an etiologic effect on T2D or weight gain as well as global availability of consumption data. The optimal intake for each factor was determined based on observed levels with lowest morbidity and mortality in the meta-analyses, feasibility based on observed national consumption levels and consistency with major food-based dietary guidelines (Methods)^12^. In 2018, global mean intakes of these 11 dietary factors estimated by the GDD were suboptimal, including insufficient intake of fruits (observed mean (s.d.): 87.9 g per d (84.9, 90.8) versus optimal, 300.0 g per d), non-starchy vegetables (209.8 g per d (202.2, 217.4) versus 300.0 g per d), nuts and seeds (8.6 g per d (7.7, 9.7) versus 20.3 g per d), whole grains (50.1 g per d (44.2, 55.2) versus 90.0 g per d) and yogurt (21.2 g per d (18.3, 25.1) versus 87.1 g per d) and excess intake of potatoes (47.8 g per d (42.7, 55.2) versus 0.0 g per d), refined rice and wheat (302.9 g per d (265.1, 354.8) versus 0.0 g per d), processed meats (16.8 g per d (14.7, 19.9) versus 0.0 g per d), unprocessed red meats (56.5 g per d (53.3, 59.9) versus 14.3 g per d), SSBs (95.6 g per d (89.1, 103.0) versus 0.0 g per d) and fruit juices (15.1 g per d (14.0, 16.4) versus 0.0 g per d) (Supplementary Tables 1 and 2). In 2018, based on Global Burden of Disease data, a total of 20.1 million (95% UI, 19.9–20.3 million) new T2D cases occurred among adults globally, with the greatest absolute number of annual new cases occurring in southeast and East Asia (5.8 million (5.7, 5.9 million)) and South Asia (4.7 million (4.6, 4.8 million)).

### Estimated T2D cases attributable to suboptimal diet

**Fig. 1:**
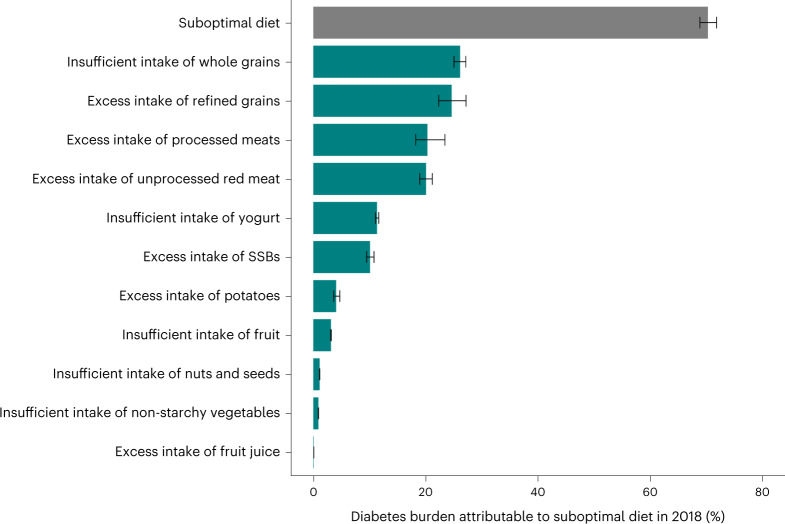
The proportional burden of T2D attributable to suboptimal diet jointly and by each individual dietary factor globally in 2018. Bars represent the estimated percentage of T2D incidence due to suboptimal intake of 11 dietary factors jointly (suboptimal diet) and separately at the global level in 2018. The burden due to suboptimal diet was estimated using proportional multiplication, assuming that half the benefit of whole-grain intake is mediated through replacement of refined rice and wheat intake. Refined rice and wheat were modeled separately but combined for this aggregate analysis using proportional multiplication. The attributable burden of T2D for four dietary factors (insufficient intake of fruit, nuts and seeds, non-starchy vegetables and excess intake of fruit juice) were estimated only based on effects mediated through weight gain (for example, no direct effects on T2D risk were identified in the literature). See Supplementary Table 5 for more details on the inputs for each dietary factor. Data are presented as the central estimate (median) and the corresponding 95% UI, derived from the 2.5th and 97.5th percentiles of 1,000 multiway probabilistic Monte Carlo model simulations.

In 2018, a total of 14.1 million (95% UI: 13.8, 14.4 million) estimated new T2D cases, or 70.3% (95% UI: 68.8–71.8%) of the total, were estimated to be due to suboptimal intake of the 11 dietary factors (Supplementary Table 3 and Fig. 1). Excess intake of six harmful dietary factors jointly (refined rice and wheat, processed meats, unprocessed red meat, SSBs, potatoes, fruit juice) contributed a larger proportion of the total global diet-attributable burden (60.8%) than insufficient intake of five protective dietary factors (whole grains, yogurt, fruits, non-starchy vegetables, nuts and seeds) (39.2%) (Supplementary Table 4). These proportions were generally similar across world regions in 2018 and globally and across world regions in 1990.

Among individual dietary factors in 2018, insufficient whole grains (26.1% (25.0–27.1%)), excess refined rice and wheat (24.6% (22.3–27.2%)), excess processed meats (20.3% (18.3–23.5%)) and excess unprocessed red meats (20.1% (19.0–21.2%)) were associated with the highest estimated attributable burden of T2D incidence globally (Fig. 1). Lowest burdens were attributable to dietary factors having only BMI-mediated effects, such as excess fruit juice (0.09% (0.09–0.1%)), insufficient non-starchy vegetables (0.9% (0.9–1.0%)) and insufficient nuts and seeds (1.1% (1.1–1.2%)).

### Diet-attributable T2D by world region and nation

**Fig. 2:**
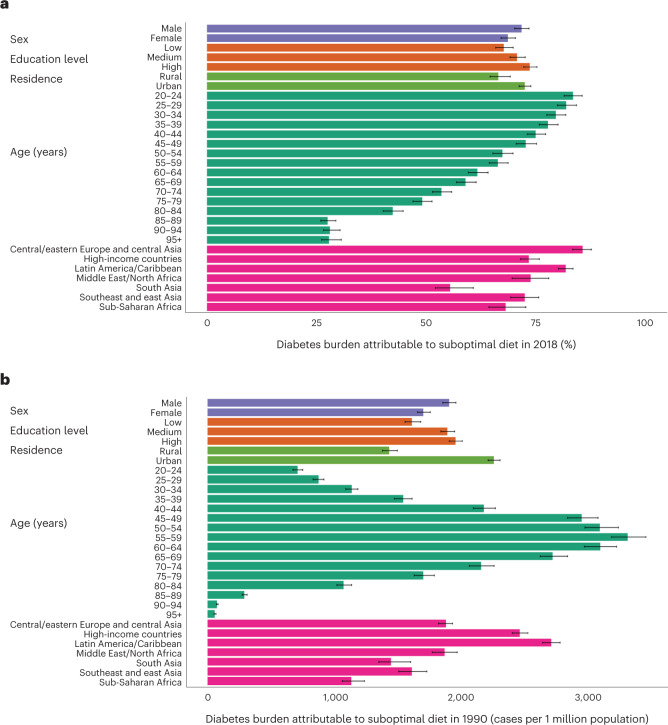
The burden of T2D attributable to suboptimal diet by key sociodemographic factors at the global level in 2018. Bars represent the estimated percentage burden (**a**) and absolute burden per 1 million population (**b**) of T2D incidence due to suboptimal intake of 11 dietary factors jointly: insufficient intake of whole grains, yogurt, fruit, nuts and seeds, and non-starchy vegetables and excess intake of refined rice and wheat, processed meats, unprocessed red meat, SSBs, potatoes and fruit juice. The burden due to suboptimal diet was estimated using proportional multiplication, assuming that half the benefit of whole-grain intake is mediated through replacement of refined rice and wheat intake. See Supplementary Table 5 for more details on the inputs for each dietary factor. Data are presented as the central estimate (median) and the corresponding 95% UI, derived from the 2.5th and 97.5th percentiles of 1,000 multiway probabilistic Monte Carlo model simulations.

Across world regions, highest proportional diet-attributable burdens of T2D were in central and eastern Europe and central Asia (85.6% (95% UI: 83.4–87.7%)) and Latin America and the Caribbean (81.8% (80.1–83.4%)), and lowest proportional diet-attributable burdens of T2D were in South Asia (55.4% (52.1–60.7%)) and sub-Saharan Africa (68.1% (64.3–72.7%)) (Fig. 2). Per 1 million population, T2D cases attributable to diet were highest in Latin America and the Caribbean (4,152 per million population (4,056, 4,254)) followed by the Middle East and North Africa (3,827 per million population (3,607, 4,042)).

**Fig. 3:**
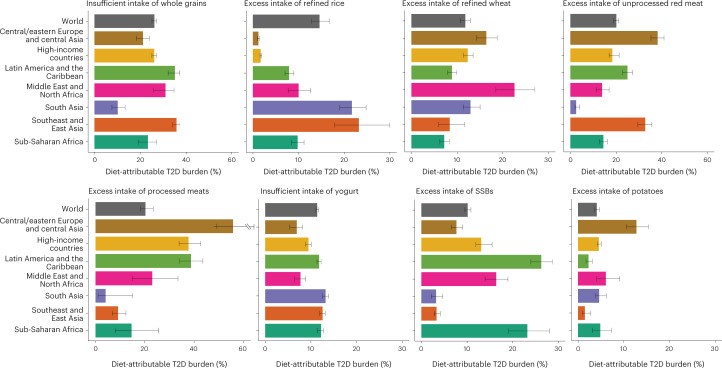
The proportional burden of T2D attributable to suboptimal intake of eight individual risk factors by world region in 2018. Bars represent the estimated percentage of T2D incidence due to suboptimal intake of eight individual dietary factors separately. The attributable burden of T2D for four dietary factors (insufficient intake of fruit, nuts and seeds, and non-starchy vegetables and excess intake of fruit juice) was estimated only based on effects mediated through weight gain (that is, no direct effects on T2D risk were identified in the literature) and is reported in Extended Data Fig. 1. Countries were delineated into world regions by the GDD. Data are presented as the central estimate (median) and the corresponding 95% UI, derived from the 2.5th and 97.5th percentiles of 1,000 multiway probabilistic Monte Carlo model simulations.

**Extended Data Fig. 2:**
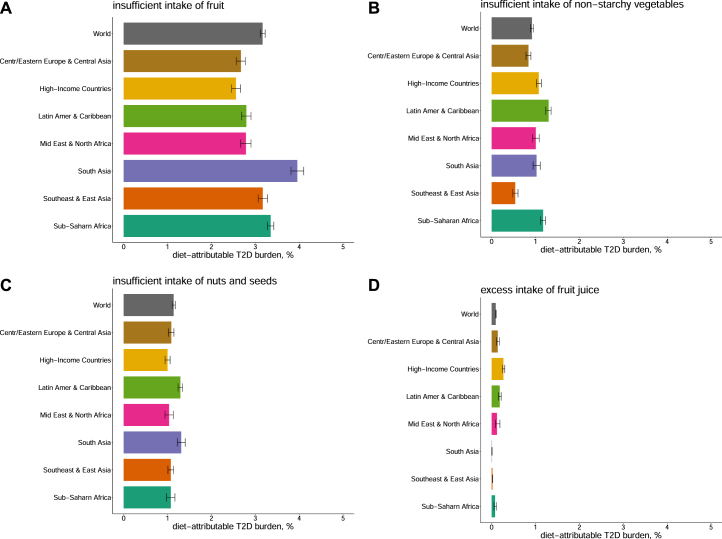
The proportional burden of T2D attributable to suboptimal intake of four individual risk factors by world region in 2018, %. Bars represent the estimated percentage of T2D incidence due to suboptimal intake of 4 individual dietary factors – insufficient intake of (**a**) fruit, (**b**) non-starchy vegetables, (**c**) nuts and seeds; and excess intake of (**d**) fruit juice – that were estimated only based on effects mediated through weight gain (for example, no direct effects on T2D risk were identified in the literature). Remaining dietary factors reported in Fig. 3 in the main text. Countries were delineated into world regions by the Global Dietary Database. Data are presented as the central estimate (median) and corresponding 95% uncertainty interval, derived from the 2.5th and 97.5th percentiles of 1000 multiway probabilistic Monte Carlo model simulations.

We identified heterogeneity in attributable burdens of T2D for specific dietary factors at regional and national levels. About one in three new T2D cases were estimated to be attributable to insufficient whole grains in southeast and East Asia (35.8% (34.1–37.3%)) and Latin America and the Caribbean (35.0% (32.0–37.1%)), compared with one in ten cases in South Asia (10.1% (7.5–13.2%)) (Fig. 3). The estimated attributable T2D burden from excess refined rice was 23.1% (17.9–29.9%) in southeast and East Asia but <2% in central and eastern Europe and central Asia and high-income countries in 2018. Excess refined wheat was associated with the highest estimated T2D burden in the Middle East and North Africa (22.5% (18.5–27.1%)). Large regional differences were seen in the estimated T2D burden of excess unprocessed red meats, ranging from 38.2% (35.3–40.9%) in central and eastern Europe and central Asia to 2.6% (2.1–4.1%) in South Asia. Excess processed meats were estimated to be associated with more than half (55.7% (49.1–61.3%)) of new T2D cases in central and eastern Europe and central Asia but only 4.2% (1.1–15.1%) in South Asia. The burden of T2D cases attributable to excess SSBs was highest in Latin America and the Caribbean (26.2% (24.0–28.7%)) and lowest in South Asia (3.3% (2.3–4.8%)). Excess intake of potatoes was associated with the highest proportional T2D burden in central and eastern Europe and central Asia (12.7% (10.4–15.4%)). Generally, excess intake of fruit juice and insufficient intake of yogurt, fruit, non-starchy vegetables, and nuts and seeds had lower attributable burdens and less heterogeneity by world region (Fig. 3 and Extended Data Fig. 2).

**Fig. 4:**
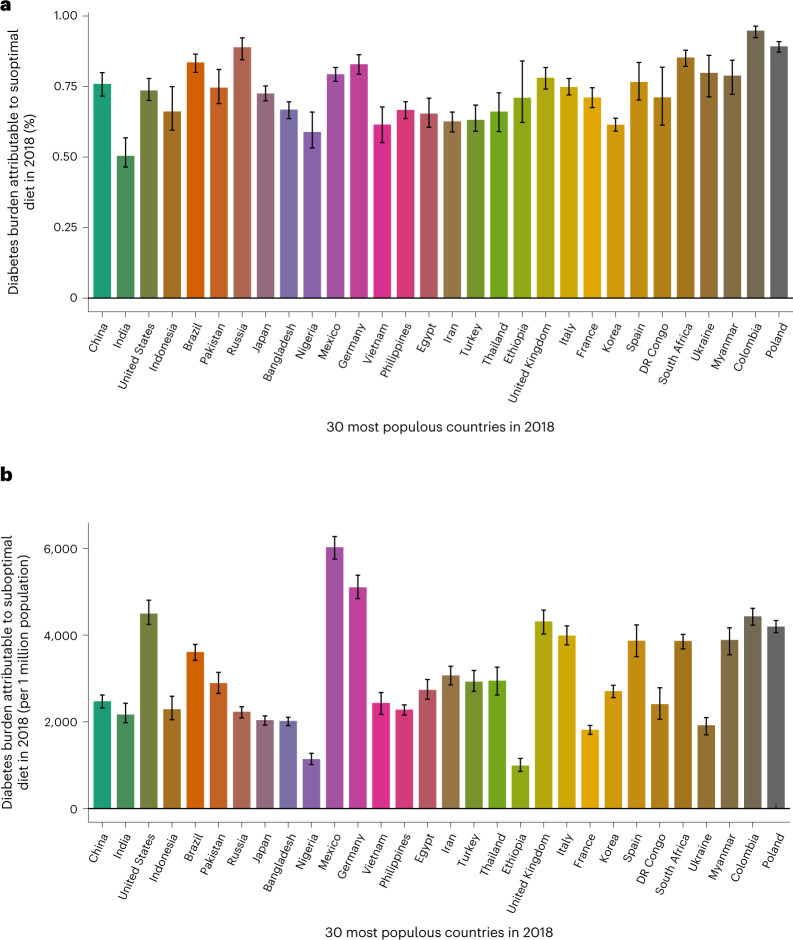
The burden attributable to suboptimal diet at the national level in the top 30 most populous countries in 2018. Bars represent the estimated percentage burden (**a**) and absolute burden per 1 million population (**b**) of T2D incidence due to suboptimal intake of 11 dietary factors jointly: insufficient intake of whole grains, yogurt, fruit, nuts and seeds, and non-starchy vegetables and excess intake of refined rice and wheat, processed meats, unprocessed red meat, SSBs, potatoes and fruit juice. The burden due to suboptimal diet was estimated using proportional multiplication, assuming that half the benefit of whole-grain intake is mediated through replacement of refined rice and wheat intake. Countries are ordered based on population size in 2018, from highest to lowest. See Supplementary Table 5 for more details on the inputs for each dietary factor. Data are presented as the central estimate (median) and the corresponding 95% UI, derived from the 2.5th and 97.5th percentiles of 1,000 multiway probabilistic Monte Carlo model simulations.

**Extended Data Fig. 3:**
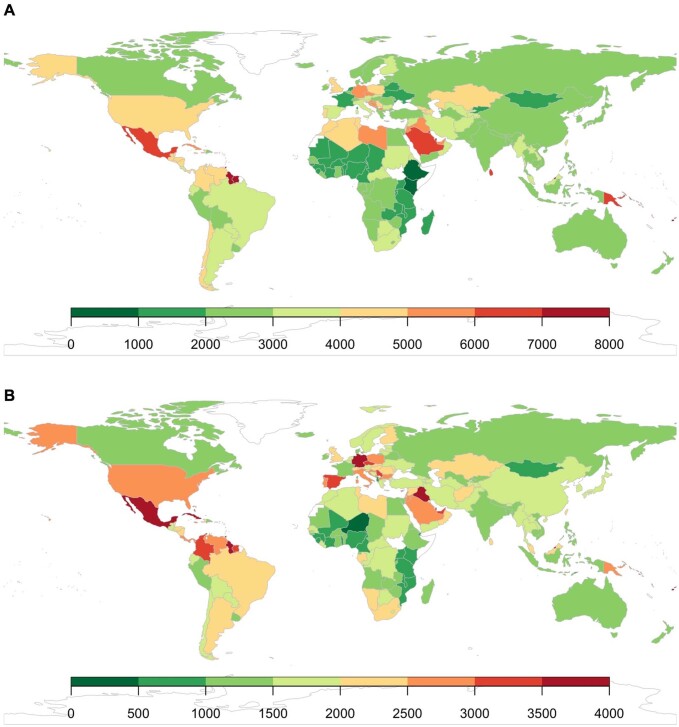
The absolute burden of T2D attributable to suboptimal diet at the national level per 1 M population in (A) 1990 and (B) 2018. Heatmap reflects the estimated absolute burden of T2D incidence due to suboptimal intake of 11 dietary factors jointly: insufficient intake of whole grains, yogurt, fruit, nuts and seeds, and non-starchy vegetables; and excess intake of refined rice and wheat, processed meats, unprocessed red meat, sugar-sweetened beverages, potatoes, and fruit juice. The burden due to suboptimal diet was estimated using proportional multiplication, assuming that half the benefit of whole grains intake is mediated through replacement of refined rice and wheat intake. The absolute burden per 1 million population was calculated by dividing the absolute number of diet-attributable cases by the country population in that year and multiplying by 1 million. Different scales (0 to 4000 cases vs. 0 to 8000 cases) were used to better reflect the absolute case distribution globally in 1990 and 2018, respectively.

Considering the 30 most populous countries, the proportional diet-attributable burden of T2D was highest in Colombia (94.6% (95% UI: 92.4–96.4%)) and Poland (89.0% (87.2–91.0%)) and lowest in India (50.2% (46.5–56.9%)) (Fig. 4). However, per million population, Mexico (6,015 cases (95% UI: 5,751, 6,275)) and Germany (5,091 cases (4,841, 5,383)) had the highest estimated diet-attributable T2D burdens, while Ethiopia (976 cases (856, 1,156)) and Nigeria (1,127 cases (1,013, 1,272)) had the lowest. Global national heat maps and detailed tables of national proportional and absolute T2D burdens attributable to suboptimal diet jointly and separately in 1990 and 2018 for all countries are presented in Extended Data Fig. 3 and Supplementary Tables 3 and 5.

### Trends between 1990 and 2018

**Fig. 5:**
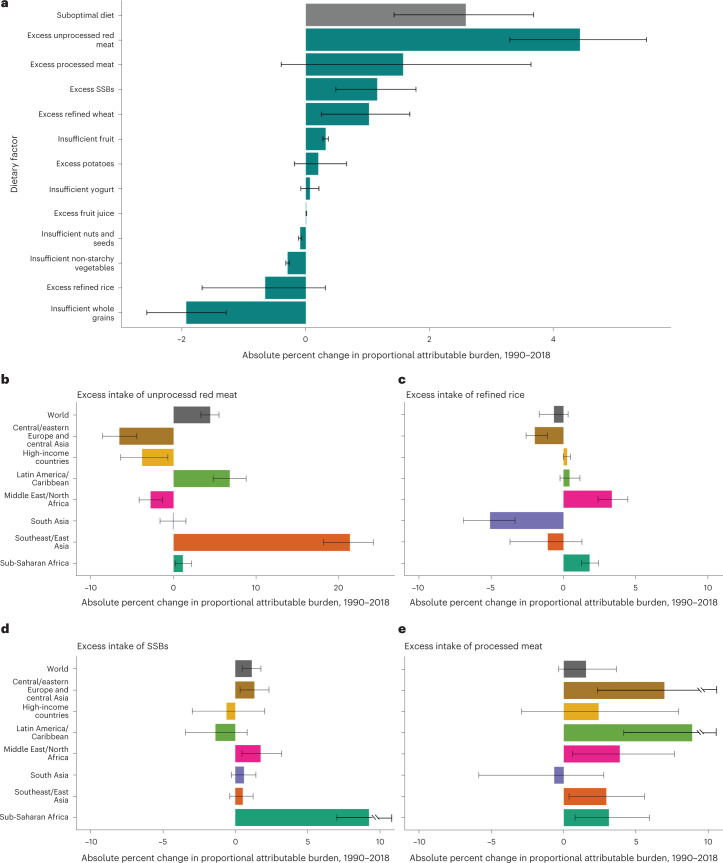
The absolute change in the proportional burden of T2D attributable to suboptimal diet and each individual risk factor between 1990 and 2018 globally and by world region for four select dietary factors. Bars represent the estimated absolute change in proportional burden of T2D incidence (**a**) globally due to suboptimal intake of 12 dietary factors jointly and individually: insufficient intake of whole grains, yogurt, fruit, nuts and seeds, and non-starchy vegetables and excess intake of refined rice, refined wheat, processed meats, unprocessed red meat, SSBs, potatoes and fruit juice. The burden due to suboptimal diet was estimated using proportional multiplication, assuming that half the benefit of whole-grain intake is mediated through replacement of refined rice and wheat intake. In addition, excess intake of four dietary factors (unprocessed red meat (**b**), refined rice (**c**), SSBs (**d**) and processed meat (**e**)) is included as illustrative examples of the estimated absolute change in percentage burden of T2D, with the remaining dietary factors included in Extended Data Figs. 5 and 6. A different *x*-axis range was used for **b** to account for the magnitude of absolute change in T2D burden attributable to excess intake of unprocessed red meat in southeast and East Asia. A negative absolute change in proportional burden indicates a reduction in the diet-attributable burden of T2D between 1990 and 2018 (for example, reduced intake of harmful dietary factors, increased intake of protective dietary factors), while a positive absolute change in percentage burden indicates an increase in the diet-attributable burden of T2D during that time frame (for example, increased intake of harmful dietary factors, decreased intake of harmful dietary factors). Countries were delineated into world regions by the GDD. Data are presented as the central estimate (median) and the corresponding 95% UI, derived from the 2.5th and 97.5th percentiles of 1,000 multiway probabilistic Monte Carlo model simulations.

**Extended Data Fig. 4:**
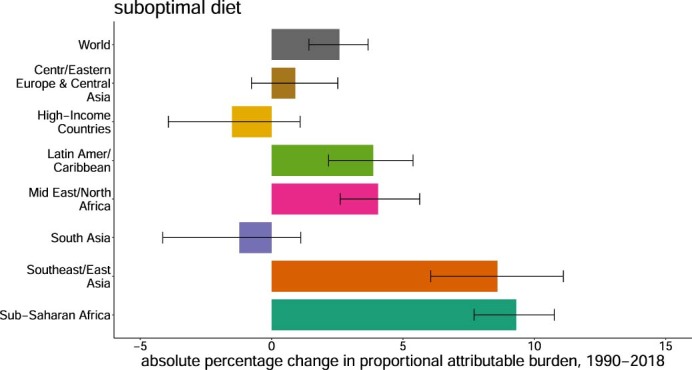
The absolute change in the proportional burden of T2D attributable to suboptimal diet by world region between 1990–2018. Bars represent the estimated absolute change between 1990 and 2018 by world region in proportional burden of T2D incidence attributable to suboptimal intake of 11 dietary factors jointly: insufficient intake of whole grains, yogurt, fruit, nuts and seeds, and non-starchy vegetables; and excess intake of refined rice and wheat, processed meats, unprocessed red meat, sugar-sweetened beverages, potatoes, and fruit juice. The burden due to suboptimal diet was estimated using proportional multiplication, assuming that half the benefit of whole grains intake is mediated through replacement of refined rice and wheat intake. A negative absolute change in proportional burden indicates a reduction in the diet-attributable burden of T2D between 1990 and 2018, while a positive absolute change in percentage burden indicates an increase in the diet-attributable burden of T2D during that time frame. Countries were delineated into world regions by the Global Dietary Database. Data are presented as the central estimate (median) and corresponding 95% uncertainty interval, derived from the 2.5th and 97.5th percentiles of 1000 multiway probabilistic Monte Carlo model simulations.

**Extended Data Fig. 5:**
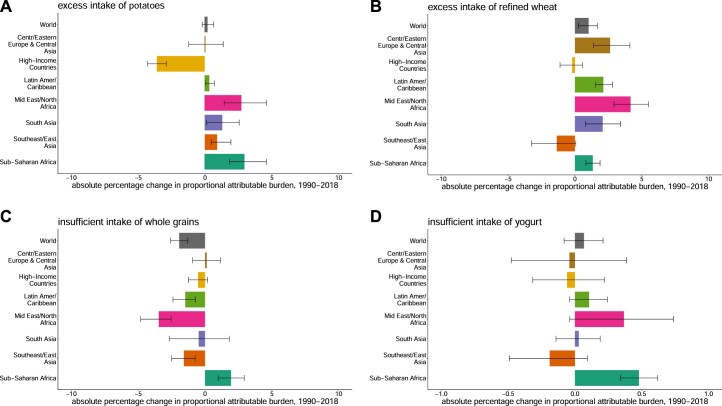
The absolute change in the proportional burden of T2D attributable to (A) excess potatoes, (B) excess refined wheat, (C) insufficient whole grains, and (D) insufficient yogurt by world region between 1990–2018. Bars represent the estimated absolute change in proportional burden of T2D incidence between 1990 and 2018 attributable to four dietary factors, globally and by world region – (**a**) excess intake of potatoes, (**b**) excess intake of refined wheat, (**c**) insufficient intake of whole grains, and (**d**) insufficient intake of yogurt. Note varying x-axis ranges across dietary factors. A negative absolute change in proportional burden indicates a reduction in the diet-attributable burden of T2D between 1990 and 2018 (for example, reduced intake of harmful dietary factors, increased intake of protective dietary factors), while a positive absolute change in percentage burden indicates an increase in the diet-attributable burden of T2D during that time frame (for example, increased intake of harmful dietary factors, decreased intake of harmful dietary factors). Countries were delineated into world regions by the Global Dietary Database. Data are presented as the central estimate (median) and corresponding 95% uncertainty interval, derived from the 2.5th and 97.5th percentiles of 1000 multiway probabilistic Monte Carlo model simulations.

**Extended Data Fig. 6:**
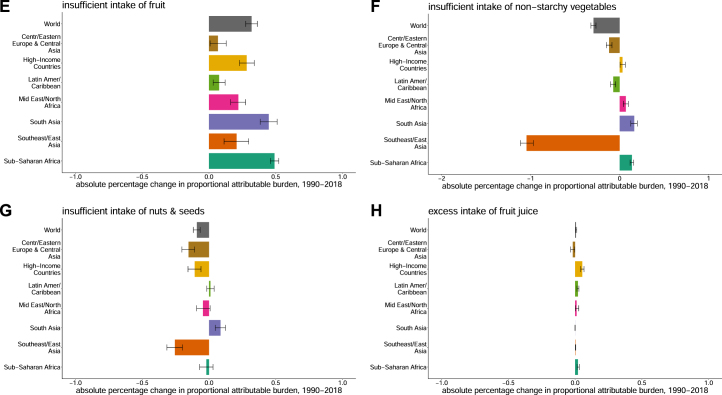
The absolute change in the proportional burden of T2D attributable to (E) insufficient fruit, (F) insufficient non-starchy vegetables, (G) insufficient nuts & seeds, and (H) excess fruit juice by world region between 1990–2018. Bars represent the estimated absolute change in proportional burden of T2D incidence between 1990 and 2018 attributable to eight dietary factors, globally and by world region – (**e**) insufficient intake of fruit, (**f**) insufficient intake of non-starchy vegetables, (**g**) insufficient intake of nuts & seeds, and (**h**) excess intake of fruit juice. Note varying x-axis ranges across dietary factors. A negative absolute change in proportional burden indicates a reduction in the diet-attributable burden of T2D between 1990 and 2018 (for example, reduced intake of harmful dietary factors, increased intake of protective dietary factors), while a positive absolute change in percentage burden indicates an increase in the diet-attributable burden of T2D during that time frame (for example, increased intake of harmful dietary factors, decreased intake of harmful dietary factors). Countries were delineated into world regions by the Global Dietary Database. Data are presented as the central estimate (median) and corresponding 95% uncertainty interval, derived from the 2.5th and 97.5th percentiles of 1000 multiway probabilistic Monte Carlo model simulations.

**Extended Data Fig. 7:**
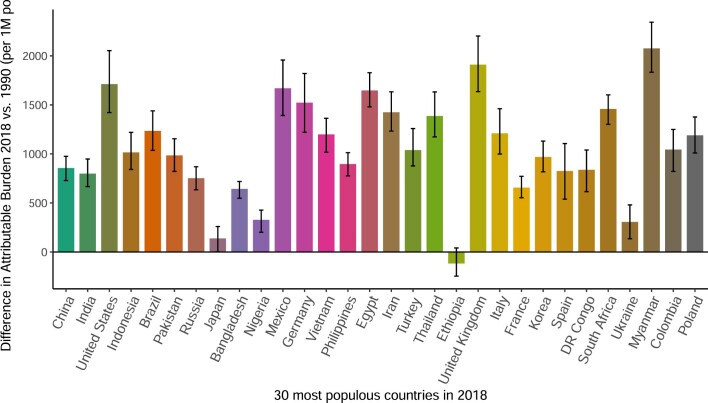
Difference in the absolute burden of T2D attributable to suboptimal diet between 1990–2018 in the top 30 most populous countries in 2018. Bars represent the estimated absolute change in the absolute burden per 1 M population of T2D incidence due to suboptimal intake of 11 dietary factors jointly: insufficient intake of whole grains, yogurt, fruit, nuts and seeds, and non-starchy vegetables; and excess intake of refined rice and wheat, processed meats, unprocessed red meat, sugar-sweetened beverages, potatoes, and fruit juice. The burden due to suboptimal diet was estimated using proportional multiplication, assuming that half the benefit of whole grains intake is mediated through replacement of refined rice and wheat intake. Countries are ordered based on population size in 2018, from highest to lowest. See Supplementary Table 1 for more details on the inputs for each dietary factor. Data are presented as the central estimate (median) and corresponding 95% uncertainty interval, derived from the 2.5th and 97.5th percentiles of 1000 multiway probabilistic Monte Carlo model simulations.

Global trends in diet-attributable T2D burden between 1990 and 2018 are described in Supplementary Note 1 and Fig. 5. Regionally, the largest increases in diet-attributable T2D burdens were in sub-Saharan Africa (+9.3 absolute percentage points (95% UI: 7.7–10.8%)) and southeast and East Asia (+8.6% (6.1–11.1%)), and the largest (although non-significant) declines were in South Asia (−1.2% (−4.1% to 1.1%)) and high-income countries (−1.5% (−3.9% to 1.1%)) (Extended Data Fig. 4). Certain dietary factors had considerable regional heterogeneity (Fig. 5 and Extended Data Figs. 5 and 6). The T2D burden attributable to excess unprocessed red meat increased by 21.3 absolute percentage points (18.1–24.1%) in southeast and East Asia but declined in central and eastern Europe and central Asia (−6.5% (−8.6% to −4.4%)), high-income countries (−3.8% (−6.4% to −0.7%) and the Middle East and North Africa (−2.8% (−4.2% to −1.4%)) (Fig. 5). T2D cases attributable to excess refined rice declined, but increased for excess refined wheat, in South Asia and central and eastern Europe and central Asia (Fig. 5 and Extended Data Fig. 5), while increasing T2D burdens for refined wheat and rice were observed in the Middle East and North Africa (+4.1% (2.9–5.5%) and +3.3% (2.4–4.4%), respectively) and sub-Saharan Africa (+1.3% (0.8–1.9%) and +1.8% (1.2–2.4%)). The T2D burden attributable to SSBs increased most in sub-Saharan Africa (+9.4% (7.1–11.8%)), with more modest changes in other world regions. The proportional T2D burden attributable to processed meat increased in all regions except South Asia. Trends in the 30 most populous countries are discussed in Supplementary Note 1 and shown in Extended Data Fig. 7.

### Findings by age, sex, education level and urbanicity

**Extended Data Fig. 8:**
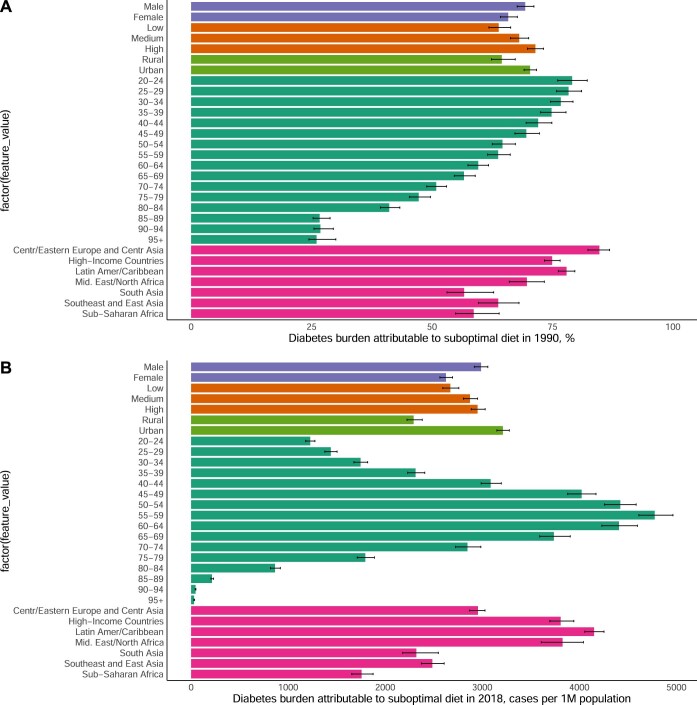
The burden of T2D attributable to suboptimal diet by key sociodemographic factors at the global level in 1990. Bars represent the estimated (**a**) percentage burden and (**b**) absolute burden per 1 M population of T2D incidence due to suboptimal intake of 11 dietary factors jointly: insufficient intake of whole grains, yogurt, fruit, nuts and seeds, and non-starchy vegetables; and excess intake of refined rice and wheat, processed meats, unprocessed red meat, sugar-sweetened beverages, potatoes, and fruit juice. The burden due to suboptimal diet was estimated using proportional multiplication, assuming that half the benefit of whole grains intake is mediated through replacement of refined rice and wheat intake. See Supplementary Table 1 for more details on the inputs for each dietary factor. Data are presented as the central estimate (median) and corresponding 95% uncertainty interval, derived from the 2.5th and 97.5th percentiles of 1000 multiway probabilistic Monte Carlo model simulations.

All findings were evaluated subnationally, jointly stratified by age, sex, educational attainment and urban or rural residence. Globally, the diet-attributable T2D burden was generally greater in males (proportional, 71.7% (95% UI: 70.2–73.4%); per million, 2,987 cases (95% UI: 2,918, 3,058)) versus females (68.6% (67.0–70.3%); 2,626 cases (2,564, 2,694)) (Fig. 2). Proportional burdens were higher in younger adults (aged 20–25 years, 83.5% (81.4–85.5%)) versus older adults (aged 95+ years, 27.7% (26.1–30.6%)), but middle-aged adults had the highest burden per million (for example, aged 55–59 years, 4,777 cases (4,613, 4,964)). These sex- and age-specific differences were generally similar in 1990 (Extended Data Fig. 8).

**Extended Data Fig. 9:**
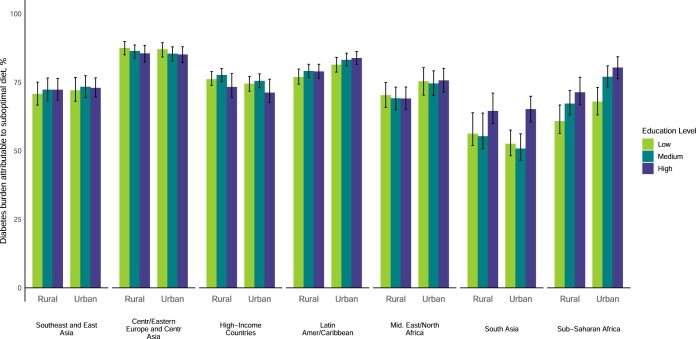
Proportional burden of T2D attributable to suboptimal diet by urbanicity and education level at the world region level in 2018. Bars represent the estimated proportional burden of T2D incidence due to suboptimal intake of 11 dietary factors jointly: insufficient intake of whole grains, yogurt, fruit, nuts and seeds, and non-starchy vegetables; and excess intake of refined rice and wheat, processed meats, unprocessed red meat, sugar-sweetened beverages, potatoes, and fruit juice. The burden due to suboptimal diet was estimated using proportional multiplication, assuming that half the benefit of whole grains intake is mediated through replacement of refined rice and wheat intake. Education level and urban/rural residence were defined previously by the Global Dietary Database Project. Uncertainty in the T2D incidence estimates by education level and urban/rural residence were not incorporated into the population attributable fraction calculation. See Methods for further details. Data are presented as the central estimate (median) and corresponding 95% uncertainty interval, derived from the 2.5th and 97.5th percentiles of 1000 multiway probabilistic Monte Carlo model simulations.

By education globally, estimated diet-attributable T2D burden was highest among individuals with high education (proportional, 73.6% (72.2–75.4%); per million, 2,952 cases (2,886, 3,030)) versus those with medium (70.7% (69.1–72.5%); 2,873 cases (2,807, 2,951)) or low (67.7% (65.8–69.8%); 2,670 cases (2,592, 2,759)) education (Fig. 2). This pattern was seen in all world regions except for in high-income countries and central and eastern Europe and central Asia, where populations with medium education and low education, respectively, had the largest diet-attributable proportional T2D burden in 2018 (Extended Data Fig. 9).

By residence globally, the estimated T2D burden attributable to suboptimal diet was higher among populations residing in urban (proportional, 72.5% (71.1–73.8%); per 1 million, 3,213 cases (3,150, 3,279)) versus rural (66.5% (64.5–69.1%); 2,293 cases (2,225, 2,381)) areas, with the largest regional differences by residence identified in the Middle East and North Africa and sub-Saharan Africa (Extended Data Fig. 9).

### Findings by national sociodemographic index

**Fig. 6:**
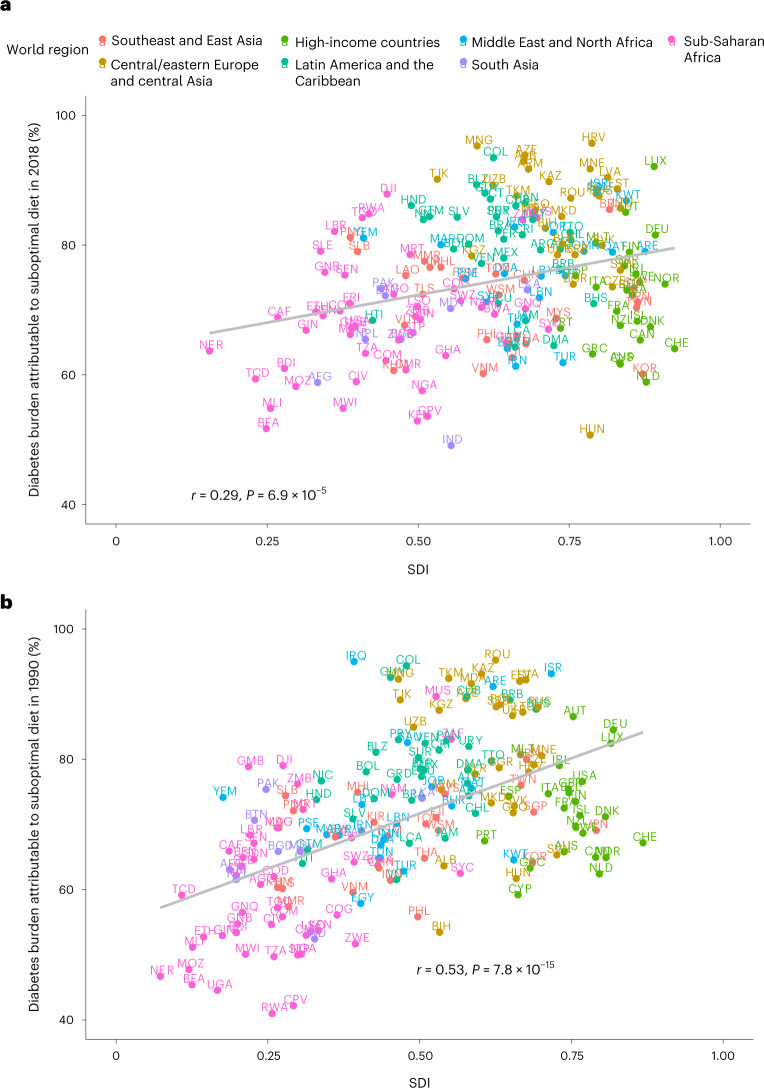
Correlation of national-level diet-attributable T2D burden and national SDI in 2018 and 1990. Points represent the 184 countries included in this analysis (labeled with their ISO3 code and colored based on world region) in 2018 (**a**) and 1990 (**b**). The gray line represents overall correlation, with Pearson correlation coefficient and associated *P* value (two-tailed) provided. No adjustments were made for multiple comparisons. The *y* axis is based on estimated proportional burden of T2D incidence due to suboptimal intake of 11 dietary factors jointly: insufficient intake of whole grains, yogurt, fruit, nuts and seeds, and non-starchy vegetables and excess intake of refined rice and wheat, processed meats, unprocessed red meat, SSBs, potatoes and fruit juice. The burden due to suboptimal diet was estimated using proportional multiplication, assuming that half the benefit of whole-grain intake is mediated through replacement of refined rice and wheat intake. SDI is a measure of a nation’s development expressed on a scale of 0 to 1 sourced from the Global Burden of Disease study, based on a compositive average of the rankings of income per capita, average educational attainment and fertility rates.

We also assessed national findings by sociodemographic index (SDI), a composite measure of a country’s development based on income per capita, educational attainment and fertility rates (Methods). In 2018, national diet-attributable T2D burdens were only modestly correlated with SDI (*r* = 0.29) (Fig. 6). This varied by world region, with a positive association among nations in sub-Saharan Africa, South Asia, the Middle East and North Africa, and high-income countries, but an inverse association among nations in central and eastern Europe and central Asia, Latin America and the Caribbean, and southeast and East Asia. In 1990, the association between national diet-attributable T2D burdens and SDI was stronger (*r* = 0.53) than in 2018, with similar trends by world region (Fig. 6). No bivariate outliers in the association between joint attributable T2D burden and SDI were detected based on statistical analysis.

## Discussion

Based on globally representative and stratified estimates of dietary intake and T2D incidence, our modeling investigation estimates that, in 2018, seven in ten (70.3% (95% UI: 68.8–71.8%)) or 14.1 million (95% UI: 13.8–14.4 million) new T2D cases globally are attributable to suboptimal intake of 11 dietary factors. Excess intake of harmful dietary factors contributed a greater percentage of this burden globally (60.8%) than did insufficient intake of protective dietary factors (39.2%). Among individual dietary factors, the largest number of estimated T2D cases globally were attributable to insufficient whole grains (26.1%), excess refined rice and wheat (24.6%), excess processed meat (20.3%) and excess unprocessed red meat (20.1%). Substantial heterogeneity in diet-attributable T2D burdens overall and for each dietary factor was found by world region and nation. The proportional diet-attributable T2D burden was inversely correlated with age and was generally greater in men versus women, urban versus rural residents and for individuals with higher versus lower education, except in high-income countries and central and eastern Europe and central Asia, where the reverse was true for urbanicity and education level. National diet-attributable T2D burdens were only modestly correlated with socio-economic development, with a weakening of this association since 1990.

Highest diet-attributable T2D burdens were observed in central and eastern Europe and central Asia, particularly in populous countries such as Poland and Russia, driven by larger burdens from excess intake of unprocessed red meat, processed meat and potatoes. These findings are consistent with the region’s culinary ‘meat and potatoes’ practices and associated cardiometabolic health risk for this dietary pattern in the eastern European context^13^. Latin America and the Caribbean had the second highest estimated diet-attributable T2D burden of all world regions, especially in Colombia and Mexico, related to excess SSBs, excess processed meats and insufficient whole grains. These results are consistent with the transition toward more highly processed diets in this region^14^, including increasing processed meat intake in Colombia, Argentina and Brazil^15^ and consistently low whole-grain intake across eight Latin American countries^16^. These results also build upon previous findings of excess SSB consumption and associated cardiometabolic disease burden in Latin America and the Caribbean, as well as evidence for the adverse effects of excess SSB and processed meat intake and insufficient whole-grain intake on T2D risk (Methods).

Disparities in diet, health and disease are a critical area of public health research and practice. We found differences in diet-attributable T2D burden by education level subnationally, as well as diverging effects in these differences by world region, providing evidence to inform nutrition-related disparities globally. In high-income countries and central and eastern Europe and central Asia, populations with lower education had higher diet-attributable T2D burdens than populations with high education, indicating that educational interventions and social safety net programs in these regions should include focus on nutrition and T2D to reduce health disparities. By contrast, in Latin America and the Caribbean, South Asia and sub-Saharan Africa, diet-attributable T2D burdens were highest among adults with high educational attainment. Improving education may therefore not reduce T2D in these regions, and alternative strategies such as front-of-package labeling, marketing standards, taxation and other financial incentive schemes may be more effective^17–19^. Financial markets can also drive health and equity in the food sector, based on the business case for investing in the production, sale and marketing of products aligned with these societal goals^20^.

Our finding of similar or higher diet-attributable T2D burden in urban areas compared to rural areas in most world regions (except high-income countries and central and eastern Europe and central Asia) is consistent with estimated urban–rural differences in animal source food intake^21^ and in age-standardized adiposity, particularly in the global south^22^. However, evidence from 1985 to 2017 suggests that BMI is rising at the same or faster rates in rural areas in low- and middle-income countries (LMICs)^22^, consistent with increased supply of highly processed foods in rural areas^14^, and indicating a growing need to focus on rural nutrition and lifestyle in low- and middle-income nations. Addressing these nutrition and health disparities will require clinical, policy and public health interventions and policies tailored to local circumstances.

The global T2D proportional burden attributable to suboptimal diet was inversely correlated with age, but, per million population, absolute burden was highest at middle age (45–60 years), indicating the interplay between differences in nutritional habits versus absolute risk for T2D at different ages^23^. Given these findings, multisectoral approaches to improving diet quality across the life course may be most effective, including among children and adolescents^24–26^, when lifelong dietary habits are often formed.

We did not find a strong relationship between SDI, an integrated measure of national sociodemographic development, and diet-attributable T2D risk. This is due to various reasons for suboptimal diet quality in different nations at different levels of SDI, such as often lower intake of protective foods in lower-SDI countries and higher intakes of protective foods but also harmful foods in higher-SDI countries. Our subnational findings by education and urbanicity provide additional insights in this regard, as we identified differing directions of association in high- versus low-income countries by subnational education and urbanicity, which are each associated with sociodemographic development, dietary habits and diet-attributable risk of T2D. Notably, the relationship between SDI and diet-attributable T2D risk weakened between 1990 and 2018, largely owing to increasing diet-attributable burdens in middle-SDI and especially lower-SDI nations. Our findings suggest that diet quality is worsening in lower-SDI nations but without relative improvements in sociodemographic development, related to growing industrialization and Westernization of food in the Global South over this time period.

Changes over time were also observed in specific diet-attributable T2D burdens between 1990 and 2018 at global, regional and national levels. The proportional burden increased by 2.6 percentage points, while the absolute burden increased by about 8.6 million new cases per year, with the latter also related to increases in population growth, aging and obesity^27–29^. Excess unprocessed red meat was estimated to contribute the largest global increase in proportional diet-related T2D of all dietary factors assessed. This was driven primarily by increases in southeast and East Asia (+21.3%), largely related to pork consumption^30^, which offset declines in unprocessed red meat-attributable T2D burdens in central and eastern Europe and central Asia (−6.5%), high-income countries (−3.8%) and the Middle East and North Africa (−2.8%). The findings in southeast and East Asia mirror economic development, population growth and increased urbanization in this region over the last 28 years^31,32^, although many of these same demographic changes occurred in other regions that did not experience increased unprocessed red meat-associated T2D burden, suggesting a region-specific increased demand for red meat. By contrast, growing awareness of the adverse human health impacts (for example, cardiovascular disease, T2D, colorectal cancer) and planetary health strains (for example, greenhouse gas emissions, water and land usage, eutrophication potential) of unprocessed red meats^33^ may be contributing to the decreasing unprocessed red meat-attributable T2D burden in several world regions, including central and eastern Europe and central Asia, high-income countries and the Middle East and North Africa. The T2D burden attributable to processed meat increased in all world regions except South Asia (−0.7%), indicating generally independent shifts in, and therefore need for potentially distinct interventions to address, the consumption of unprocessed red meat versus processed meat.

Our findings implicate poor carbohydrate quality (excess refined rice and wheat, insufficient whole grains) as a leading driver of diet-attributable T2D globally, although with varying trends over time and by world region. We found estimated burdens attributable to insufficient whole grains to decrease globally since 1990, except in sub-Saharan Africa, where it increased (+2.0%). The latter result, along with our finding of increasing T2D burdens in sub-Saharan Africa attributable to refined rice and wheat, quantifies some of the health harms occurring from the shift away from traditional whole grains toward more processed, refined staples^34^. T2D burdens attributable to excess refined wheat and rice increased even more in the Middle East and North Africa (+6.7%, jointly), consistent with commodity reports of increased availability and consumption of refined grains in this region^35^. In South Asia, we identified declining (but still high) T2D burdens attributable to refined rice but increasing burdens attributable to refined wheat, consistent with the growing popularity of processed, refined wheat breads, cakes and pastries in South Asia as part of globalization and convergence toward Western diets^36^. Our findings suggest that excess refined rice and wheat and insufficient whole grains may be the top two dietary drivers of T2D globally, highlighting carbohydrate quality as an area for urgent attention.

In prior work, we estimated T2D mortality attributable to SSBs globally in 2010 (ref. ^6^). This investigation expands and updates this work by evaluating T2D incidence, assessing 11 dietary factors and extending follow-up to 2018. We found the percentage of T2D attributable to SSBs to be highest in Latin American and the Caribbean (26.2%), with modest decline (−1.4%) over the last 28 years. These findings suggest that new public health interventions in the region, including national SSB taxes, restricted availability in schools, limits on marketing and front-of-package warning labels^37–39^, may be contributing to some reduction in SSB-related T2D. By contrast, SSB-attributable T2D has skyrocketed in sub-Saharan Africa (+9.4%) since 1990, suggesting success of multinational corporate strategies to make SSBs more available, affordable and attractive in sub-Saharan Africa^34^. South Africa recently introduced a national tax on SSBs, with observed reductions in SSB intakes^40^, but otherwise strategies for addressing this growing SSB-associated T2D burden in Africa are sparse.

Our assessment of BMI-mediated effects for dietary factors associated with weight gain acknowledges the role that caloric imbalance and excess weight gain play in the etiology of T2D. This risk assessment model incorporates energy imbalance via weight change, which cannot be achieved by considering total calorie intake, as the latter does not reflect energy imbalance but rather varies with age, sex, physical activity, metabolic efficiency, body size, muscle mass and gut microbial metabolism.

Incidence of T2D attributable to direct etiologic effects of dietary factors was generally higher than their separate BMI-mediated effects. Prospective observational studies and some controlled trials support BMI-independent dose–response associations with T2D of whole grains and yogurt (protective factors) as well as glycemic load, SSBs, unprocessed red meats and processed meats (harmful factors) (Methods). Several plausible mechanisms may underlie these associations. For example, fiber and phenolics in whole grains may benefit the gut microbiome, resting metabolic expenditure, fat mass, insulin sensitivity, blood lipids and systemic inflammation^41–44^. By contrast, refined grains, starches and sugars induce rapid blood glucose and insulin spikes, hepatic de novo lipogenesis, uric acid production and increased visceral adiposity and also can displace other healthier foods in people’s diets^44^. In controlled trials, active probiotics in yogurt improve glucose–insulin homeostasis^45,46^. Mechanisms for metabolic harms of unprocessed red and processed meats require further study and may include effects of heme iron and preservatives on insulin resistance, oxidative stress, visceral adiposity, intracellular lipid accumulation and chronic inflammation^47–52^. In sum, our findings of direct (rather than only BMI-mediated) diet-attributable T2D burdens suggest that public health, clinical and policy interventions should prioritize diet quality, rather than total calories or weight alone, in global efforts to address T2D. More research is needed to better understand the interplay of diet quality, energy balance, metabolism, obesity and T2D.

Previous studies estimated that between 35% and 41% of global burdens of diabetes and ~28% in the Americas were attributable to poor diet^1,53,54^. Compared with these prior studies, our investigation evaluated 11 dietary factors (versus only six) and separately assessed both direct and BMI-mediated dietary effects (versus direct only). In a prior study that aimed to catalog dietary and non-dietary risks for T2D, global attributable burdens were estimated to be 34.9% for poor diet, 45.8% for high BMI (including diet-mediated weight gain) and 6% for low physical activity^1^. Thus, the joint T2D burden attributable to the direct effects of six dietary factors plus high BMI in that analysis would be estimated to be ~64.7%. The joint T2D burden in that analysis attributable to all risk factors beyond diet and BMI (low physical activity, air pollution, smoking, second-hand smoke, alcohol) would be estimated at ~45.1%. Thus, our findings, which incorporate 11 dietary factors (including factors with major attributable estimates such as refined grains not evaluated in prior analyses) and include both direct and BMI-mediated effects, are broadly consistent and plausible in comparison to these prior estimates, particularly when accounting for differences in etiologic effects and optimal levels, uncertainty in each model and model assumptions.

These prior studies also relied on dietary estimates derived primarily from national per-capita food availability, rather than individual-level dietary surveys. Similar to our analysis, the two global studies identified low intake of whole grains as the leading dietary risk factor^1,53^. These studies did not have data on refined rice and wheat (the second leading risk factor in our analysis), yogurt, potatoes, non-starchy vegetables or fruit juice. Our findings suggest that, based on updated data on dietary habits, etiologic effects, weight-mediated effects and optimal intakes, a high proportion of T2D is attributable to poor diet. Our investigation also assesses burdens stratified by subnational educational status and urban–rural residence, potential determinants of disparities.

Our investigation has several strengths. This study extended prior global and national analyses of diet-related cardiometabolic disease with updated dietary, BMI and T2D data. We assessed global diabetes impacts of refined grains, potatoes, non-starchy vegetables and fruit juice, which had not previously been analyzed. We incorporated both direct and BMI-mediated etiologic effects for multiple protective and harmful dietary factors, stratified associated risk by education level and urbanicity and evaluated T2D incidence rather than only mortality of DALYs. The modeling design incorporated the available estimates of finely stratified global dietary habits, T2D incidence, underweight and overweight prevalence, and diet–T2D, diet–BMI and BMI–T2D relationships. This approach estimates attributable burdens from independent lines of evidence, rather than from an ecologic analysis of national diet–disease associations. Dietary etiologic effects were derived from meta-analysis of multivariable-adjusted prospective cohorts and controlled trials and pooled analyses of long-term changes in diet and weight gain, with additional age-adjusted associations of BMI and T2D risk (Methods). The modeling framework incorporated stratum-specific data by year, country, sex, age, educational attainment and urbanicity, increasing ability to assess disparities. Uncertainties were incorporated and quantified using probabilistic sensitivity analyses, allowing estimation of the bounds of plausible effects.

The limitations should also be considered. While results are based on the available evidence for etiologic effects of diet and adiposity, our modeling approach does not prove causation, and our findings should be considered as estimates of risk. Direct etiologic effects of refined grains were based on their glycemic potential. While refined grains represent a major contribution to dietary glycemic load, this approach may not be as robust as for the direct estimates obtained for other dietary exposures. By contrast, evidence for BMI-mediated effects of refined grains was based on the long-term relationship of refined grain intake with weight gain. The multivariable-adjusted relative risks used in the analysis may overestimate effects if confounded by other unmeasured factors and may underestimate effects due to random errors in the measurement of diet. Our estimated effects of dietary factors on BMI change were derived from prospective cohorts in high-income nations, potentially limiting generalizability to other populations, although these relationships were multivariable adjusted for major sociodemographic and lifestyle factors and represent the best available estimates of how dietary changes relate to long-term weight gain. BMI-mediated effects incorporated differences by normal weight versus overweight or greater but not potentially stronger effects in adults with obesity, which may underestimate BMI-mediated effects among individuals with obesity. Dietary relationships were based on models estimating a linear relationship between dietary intake and BMI and a log linear relationship between dietary intake and T2D risk (except for whole grains, for which we used a stepwise, log linear relationship). Future research should address whether more complex diet–T2D dose–response relationships exist. Certainty of evidence was formally graded in duplicate for diet–disease relationships but not for BMI-mediated effects beyond SSBs. We also did not account for other dietary influences on T2D or adiposity, which could lead to larger diet-attributable burdens. While we incorporated uncertainty in all the modeling parameters, we did not include uncertainty in the stratification of T2D cases by education and urbanicity, given lack of rigorous data to do so. We stratified estimates by sex, age, education and urban versus rural residence, but reliable global data on other social determinants of health are not yet available and could provide further insights into global disparities in diet-attributable T2D. We did not account for non-dietary risks for T2D in our analytical models, which could result in overestimates for the joint effects of suboptimal diet on incident T2D. On the other hand, we made several efforts to minimize overestimation of our joint effects, including use of proportional multiplication; modeling half of the health benefits of whole grains as mediated by replacement of refined grains, accounting for substitution effects; and incorporating only direct and BMI-mediated dietary pathways with strong evidence for an etiologic association with T2D risk. We have shown in prior validation analyses that, using these approaches, the magnitude of estimated joint etiologic effects across multiple individual dietary factors is similar to that seen in clinical trials, prospective cohorts and risk factor feeding trials of dietary patterns^12^, suggesting that this approach reasonably accounts for intercorrelations and substitution effects and does not meaningfully overestimate joint effects.

In conclusion, our model estimates that about seven in ten new T2D cases globally are attributable to suboptimal intake of 11 dietary factors in 2018, with heterogeneity by world region, nation and within-country demographics. These findings inform dietary priorities and clinical and public health planning to improve dietary quality and reduce T2D globally.

## Methods

### Ethics and inclusion statement

Data informing the GDD modeling estimates for this study, including from LMICs, were collected between 1980 and 2020 in the form of dietary intake surveys. If nationally representative surveys were not available for a country, we also considered national surveys without representative sampling, followed by regional, urban or rural surveys, and finally large local cohorts, provided that selection and measurement biases were not apparent limitations. For countries with no surveys identified, other sources of potential data were considered, including the WHO Infobase, the STEP database and household budget survey data. As of July 2021, we have identified and retrieved 1,634 eligible survey years of data from public and private sources. Of these, 1,220 have been checked, standardized and approved for GDD 2018 model inclusion. Most identified data were either privately held or not in a format appropriate for our modeling. We thus relied almost entirely on direct author contacts in each country to provide us with exposure data directly. Roles and responsibilities of GDD Consortium members were determined and agreed upon before data sharing as part of a standardized data-sharing agreement.

The draft manuscript was shared with all GDD consortium members before submission for peer review, and all members have been included as authors of this work. We endorse the Nature Portfolio journals’ guidance on LMIC authorship and inclusion and are committed to the inclusion of researchers from LMICs in publications from the GDD. We share the GDD data with the entire consortium, encourage authors from LMICs to take the lead on analyses and papers and can provide technical and writing support to LMIC authors. For more details on the collaborative GDD data-collection process, please visit our website at https://www.globaldietarydatabase.org/methods/summary-methods-and-data-collection.

This research is locally relevant to all countries included, given that it disaggregates findings nationally and subnationally by key demographic factors such as age, sex, education level and urbanicity and thus provides local decision makers with data on a range of dietary factors and corresponding T2D risk.

This modeling investigation was exempt from ethical review board approval because it was based on published data and nationally representative, de-identified datasets without personally identifiable information. Individual surveys underwent ethical review board approval required for the applicable local context.

### Study design

A comparative risk assessment model^55^ estimated the numbers, proportions and uncertainty of global T2D cases attributable to suboptimal intake of key dietary factors (Extended Data Fig. 1). Comparative risk assessment does not use ecologic correlations to estimate risk but incorporates independently derived inputs and parameters on demographics, risk factors, their etiologic effects and disease incidence to model attributable burdens^55^. For this investigation, we leveraged input data and corresponding uncertainty in 184 countries on (1) population dietary intake distributions based on individual-level survey data from the GDD (http://www.globaldietarydatabase.org/)^56^; (2) population overweight (BMI ≥ 25 kg m^−2^) and underweight (BMI < 18.5 kg m^−2^) distributions from the NCD-RisC^57^; (3) total T2D-incidence distributions from the Global Burden of Disease study^58,59^; (4) linear, BMI-stratified effects of dietary factors on weight gain or loss^60^; age-adjusted direct etiologic effects of these factors on T2D, adjusted for BMI, and of weight gain on T2D from previous meta-analyses and pooled analyses of prospective cohorts^23,61,62^; (5) optimal dietary intake levels from previous analyses^12^; and (6) population demographic data from the United Nations Population Division^63,64^ and the Baro and Lee 2013 dataset on educational attainment^65^ (Supplementary Table 6).

### Identification of relevant dietary factors

Dietary factors were selected based on the following principles: (1) probable or convincing evidence of an etiologic effect on T2D or weight gain based on meta-analyses or pooled cohort studies; (2) preference for foods over nutrients, when possible, to minimize double counting of similar nutrients and/or foods; and (3) global dietary data availability from the GDD. The methods and results for review, identification and assessment of evidence for direct etiologic diet–disease relationships have been described^12,66^. Briefly, evidence for each diet–disease relationship was first evaluated by grading the quality of evidence according to nine different Bradford Hill criteria for causation: strength, consistency, temporality, coherence, specificity, analogy, plausibility, biological gradient and experiment^67^. This evidence grading was completed independently and in duplicate by two expert investigators. Based on these assessments, probable or convincing evidence was determined independently and in duplicate, in accordance with the criteria of the FAO–World Health Organization^68^ and with consideration of consistency with the similar criteria of the World Cancer Research Fund–American Institute for Cancer Research^69^. See Miller et al.^61^ and Supplementary Table 7 for further details on the evidence grading criteria and results of this evaluation. In total, 11 dietary factors were identified with at least probable evidence for etiologic effects on weight gain, seven of which also had evidence for direct (BMI-independent) effects on T2D risk (Supplementary Table 1).

### Global distributions of diet

The GDD systematically searched for and compiled representative data on individual-level dietary intakes from national surveys and subnational surveys as previously described^70^. The GDD included 1,220 unique dietary surveys, covering 188 countries corresponding to 99.0% of the global population (Supplementary Table 8)^70^. For each dietary factor, a Bayesian hierarchical model estimated the mean intake levels for national subgroups within each of 264 strata within a country–year, jointly stratified by age (22 age categories from 0–6 months through 95+ years), sex (female, male), education (low, medium, high) and urbanicity (urban or rural residence) from 1990 through 2018 (ref. ^70^). Three countries of the 188 countries with survey data were dropped from the GDD prediction model due to unavailability of FAO food-availability data, a crucial covariate in the prediction model. A Markov chain Monte Carlo algorithm generated 4,000 samples of the posterior distributions of the model parameters, which were then used to generate predictive distributions of mean dietary intake for each stratum^71^. Stratum-specific values were combined and weighted to the stratum’s proportion of the population for global, regional, national or other subgroup analyses. Children and adolescents (aged <20 years) were excluded from the present analysis given the relatively low rates of T2D globally in this subgroup. Given serving size differences in refined rice versus refined wheat, GDD refined grain intake estimates were disaggregated into refined rice and wheat intake and further converted into glycemic load estimates to match available etiologic effects for T2D risk, detailed in the section of Conversion of GDD refined grain intake estimates to glycemic load estimates. For the present analysis, regression-based methods were used to estimate the standard deviation corresponding to each estimated, stratum-specific mean from the dietary survey input data. These mean–s.d. pairs were then used to generate gamma-distribution parameters for usual dietary intake, detailed in the section of Estimation of gamma parameters for the distribution of usual intake.

### Conversion of GDD refined grain intake estimates to glycemic load estimates

Refined grain serving sizes vary significantly by commodity, primarily due to water weight. We restricted our definition of refined grain intake to wheat and rice, based on GDD standardized dietary factor definitions (http://www.globaldietarydatabase.org/). To account for differences in the serving sizes of rice versus wheat, we first used FAO Food Balance Sheet data for the energy availability of ‘wheat and products’ and ‘rice and products’ (kcal per capita per d) from 1990 and 2018 to calculate the available wheat and rice servings for each country–year stratum^72^.

We estimated standardized serving sizes and caloric contents as follows: wheat, 160.2 kcal per 50-g standard serving; rice, 170.9 kcal per 150-g standard serving (Supplementary Table 9). Standard serving sizes reflect the average of serving sizes reported in international laboratory analyses, selected to represent the range of commonly consumed wheat and rice products globally^73^. Caloric content per 100 g was obtained from the USDA’s FNDDS 2017–2018 dataset for each food product and then converted to calories per standard serving sizes^74^. For each country–year stratum, we calculated the available wheat and rice servings for each country–year stratum as follows:

Standardized rice availability (in servings per capita per d) = (FAO ‘rice and products’ in kcal per capita per d) ÷ (170.9 kcal per 150 g)

Standardized wheat availability (in servings per capita per d) = (FAO ‘wheat and products’ in kcal per capita per d) ÷ (160.2 kcal per 50 g).

Standardized rice and wheat availability were then used to calculate the proportion of rice and wheat grams available for each country–year stratum.

Wheat intake (g per d) = (refined grain intake) × (servings wheat) ÷ (servings rice + wheat)

Rice intake (g per d) = (refined grain intake) × (serving rice) ÷ (servings rice + wheat)

For the purposes of the BMI-mediated CRA of refined grain intake, we modeled rice- and wheat-intake estimates separately. The monotonic effect of diet on BMI change for one serving per day of refined grains thus accounted for the differences in serving sizes (50 g for wheat, 150 g for rice) and relative consumption of rice versus wheat in each stratum.

In addition, given estimates for the direct association between glycemic load, but not refined grain intake, and T2D risk were available, we then further converted estimated rice- and wheat-intake estimates to refined grain-specific glycemic load (g per d in a diet of 2,000 kcal) to match available effect sizes. Because refined grains represent the largest contribution, by far, to total dietary glycemic load, which has been related to T2D risk with at least probable evidence, it is a reasonable extension to derive estimates of the attributable burden of refined grains from their glycemic load. Glycemic load was calculated per standard serving size for each product and averaged for wheat and rice separately. The average glycemic load for wheat was calculated as 13.1 g per 50-g serving and, for rice, 30.3 g per 150-g serving.

To convert rice- and wheat-intake estimates (g per d) into glycemic load (g per d), we summed the product of the estimated rice and wheat intake by its respective average glycemic load, as follows, repeating this calculation for the upper and lower UI estimates:

Glycemic load = ((wheat intake) × (13.1 g per 50 g)) + ((rice intake) × (30.3 g per 150 g)).

### Estimation of gamma parameters for the distribution of usual intake

Dietary intakes cannot be negative, and usual intake distributions tend to be skewed to the right^75,76^. Gamma distributions were shown to be more appropriate than normal distributions for each of the dietary factors based on the analysis of GDD input data (for example, NHANES data) in a previous study^9^ and other research on assessment of population dietary intake^77,78^, as they do not allow for negative intakes and include a wide range of shapes with varying degrees of skewness^79^. The standard deviation needed to be obtained to construct the gamma distribution of intakes for our dietary factors of interest, as the GDD prediction model only generates estimates of mean intake from which the standard deviation cannot be readily derived. Parameters for gamma distribution were generated using the mean estimate from the GDD prediction model and estimated s.d. for the mean estimate from 1,000 simulations.

#### Standard deviation estimate for the distribution of usual dietary intake

Stratum-level GDD input survey data were used to fit a linear regression of the standard deviation of intake on mean intake (both adjusted for energy). To determine the appropriate transformation of the input data used for fitting the linear regression, various scatterplots of energy-adjusted means versus energy-adjusted s.d. were created. Using this approach, we concluded that a natural log transformation for both mean and s.d. was most appropriate.

We also explored excluding Demographic and Health Surveys, household surveys and outlier data, due to potential unreliability for estimating s.d. Ultimately, it was determined that no one dietary-assessment method contributed unevenly to the observed linear trend, and thus all data were included. Doing so also allowed for the largest possible sample size.

Additional work was carried out to assess the usefulness of an interaction term by world region, which was ultimately excluded. The regression model below was used for each individual diet factor, where *i* refers to each survey stratum:

$$Y_i = \beta _0 + \beta _1x_i + \varepsilon _i,$$

in which *Y*_*i*_ is the natural log of the s.d. of stratum-specific intake, *x*_*i*_ is the natural log of the mean of stratum-specific intake, and *ε*_*I*_ is random error, follows *N*(0, *σ*^2^).

#### Monte Carlo simulations for generating standard deviation distributions

For each dietary factor, estimates for *β*_0_ and *β*_1_ were used to predict 1,000 ln (s.d.) values corresponding to 1,000 iterations (*k*) of the predicted mean intake for each population stratum (*j*) using Monte Carlo simulations:

$$\widehat {Y_{jk}} = \widehat {\beta _0} + \widehat {\beta _1}\widehat {X_{jk}},$$

in which $\widehat {X_{jk}}$ is the *k*th sample draw of the predictive distribution for mean intake for population stratum *j.*

We added error, propagating uncertainty from the model estimates as well as variation within the sampling data itself by randomly drawing from a t-distribution with *n* − 1 degrees of freedom using the following equation:

$$\ln \left( {\widehat {\textrm{s.d.}_{jk}}} \right) = \hat Y_{jk} + \hat \sigma \sqrt {1 + \left( {\frac{1}{n}} \right) \times } t_k^{n - 1},$$

in which $\hat \sigma$ is the estimate for *σ*, *n* is the number of survey strata, $t_k^{n - 1}$ is the *k*th sample drawn from a t-distribution with *n* − 1 degrees of freedom, and $\widehat {\textrm{s.d.}_{jk}}$ is the *k*th sample draw of the predicted s.d. distribution for population stratum *j*.

#### Estimation of gamma parameters for the distribution of usual intake

The predictive distributions for each stratum-specific s.d. were then used to generate 1,000 corresponding shape and rate gamma parameters for the distribution of usual intake, a primary input in the CRA model using the following equations:

$$\widehat {\textrm{Shape}_{jk}} = \left( {\widehat {X_{jk}}/\widehat {\textrm{s.d.}_{jk}}} \right)^2,$$

$$\widehat {\textrm{Rate}_{jk}} = \frac{{\widehat {X_{jk}}}}{{\widehat {\textrm{s.d.}_{jk}^2}}}.$$

### Global distributions of adiposity

Prevalence of overweight (BMI ≥ 25 kg m^−2^) and underweight (BMI < 18.5 kg m^−2^) in each country–year–age–sex–urbanicity stratum and their uncertainty was obtained from the NCD-RisC, based on 2,416 population-based studies of national, regional or global trends in mean BMI, with measurements of height and weight for 128.9 million people^57^. NCD-RisC further segregated the data by place of urban or rural residence from 1985 to 2017 and excluded surveys if based solely on self-report, on subsets of the population or on children or pregnancy. NCD-RisC used a Bayesian hierarchical model to estimate mean BMI by country, year, sex, age and urbanicity. A Markov chain Monte Carlo algorithm generated 4,000 samples of the posterior distributions of the model parameters, which were then used to generate predictive distributions of mean BMI for each stratum^57^. NCD-RisC then generated multivariable regression models to convert each stratum-specific mean BMI estimate to overweight and underweight prevalence and uncertainty by country, year, age and sex^27,80^. To further stratify the NCD-RisC estimates by education level and urbanicity, we assumed that overweight- and underweight-prevalence estimates did not vary across education levels and urban versus rural residence; did not change across GDD age groups of 85–89, 90–94 and 95+ years (as NCD-RisC reports estimates for 85+ years only); and did not change between 2017 and 2018 (as NCD-RisC only reports through 2017, but this CRA analysis assesses estimates for 2018).

### Estimated diet–disease relationships

The evidence for direct (BMI-adjusted) associations (relative risks) between dietary risk factors and T2D was obtained from published systematic reviews and evidence grading, based on meta-analyses of prospective cohort studies and randomized controlled trials including multivariable adjustment for age, sex, BMI and other risk factors to reduce bias from confounding (Supplementary Table 1)^61^. Because these studies generally adjusted for BMI, we separately assessed BMI-mediated effects of diet (BMI change in kg m^−2^) based on pooled analyses of changes in diet and changes in BMI in long-term prospective cohort studies (Supplementary Table 1)^60^. Specifically, we used the associations for diet and weight gain pooled from three separate prospective cohort studies, including 50,422 women in the Nurses’ Health Study (1986–2006), 47,898 women in the Nurses’ Health Study II (1991–2003) and 22,557 men in the Health Professionals Follow-up Study (1986–2006) who were free of obesity (BMI ≥ 30 kg m^−2^) or chronic diseases and with complete data on weight and lifestyle habits at baseline. Women who became pregnant during follow-up were excluded from the analysis. Independent relations of changes in dietary habits with BMI change were assessed in 4-year periods over 20 years in the Nurses’ Health Study, 12 years in the Nurses’ Health Study II and 20 years in the Health Professionals Follow-up Study, using linear regression with robust variance and accounting for within-person repeated measures.

Based on previous analyses demonstrating decreasing proportional effects of metabolic risk factors on T2D incidence at older ages, age-specific relative risks were calculated for each diet–T2D etiologic relationship^12,23^, based on the mean age at event and follow-up duration (see details below on incorporating Heterogeneity in diet–disease relationships using age-specific relative risks). Associations of dietary factors with BMI change were estimated separately for overweight (BMI ≥ 25 kg m^−2^) versus non-overweight adults (BMI < 25 kg m^−2^), given observed effect modification by baseline BMI status (Supplementary Table 10)^60^. Relationships of BMI with incident T2D were obtained from a pooled analysis of multiple cohort studies on the quantitative effects of metabolic risk factors on CVD and diabetes^23^, with age-specific relative risks (RRs) modified as described in Text S3.

### Heterogeneity in diet–disease relationships using age-specific relative risks

Consistent with previous investigations, we incorporated proportional effects of major risk factors on T2D varying by age, with a log linear age association^12^. Given limited evidence of significant effect modification by sex, we incorporated similar proportional effects of risk factors by sex^12^.

In previous work, the proportional differences in RRs for major diet-related cardiometabolic risk factors, including systolic blood pressure, BMI, fasting plasma glucose (FPG) and total cholesterol, across six 10-year age groups from 25–34 years to 75+ years were evaluated. Given similarities across these four risk factors, the mean proportional differences in RR across all risk factors were applied to the dietary relative risks. For the present analysis, these mean proportional differences were disaggregated into 16 5-year age groups from 20–24 years to 95+ years by linearly scaling between each 10-year mean proportional difference in log (RR).

To calculate de novo the average age at event for each diet–disease pair, we extracted the following data from each original study included in the respective diet–disease meta-analysis: average age at baseline (years), follow-up time (years), type of follow-up time reported (maximum, median or mean) and study weight for each meta-analysis. When baseline age range rather than average baseline age was reported, we calculated the average. Weights were corrected when specific studies were excluded from the meta-analysis due to study quality limitations to sum to 1. When study weights were not reported, log (incident cases) for each study were used as a proxy indication of each study’s weight within the meta-analysis.

The average age at event was estimated as the weighted average of the sum of the average baseline age and half the maximum follow-up time reported (or two-thirds of the mean or median follow-up time reported) for each original study included in the respective diet–disease meta-analysis. See Supplementary Table 2 for estimated average age at event for each risk factor.

To quantify and incorporate the previously observed effect modification by age, we calculated age-specific relative risk for each diet–disease pair by applying the mean proportional differences in RR by age across all diet–disease pairs; we anchored at the calculated mean age at event for each diet–disease pair (Supplementary Table 11). We used Monto Carlo simulations to estimate the uncertainty in the age-distributed log (RR), sampling from the distribution of log (RR) at the age at event. Based on 1,000 simulations, we used the 2.5th and 97.5th percentiles to derive the 95% UI. An example is presented for the average age-at-event calculation (Supplementary Table 12) and resulting age-adjusted risks for potato intake and T2D risk (Supplementary Fig. 1).

### Incorporating nonlinearity in the whole-grain–T2D risk association

Due to identified inconsistencies in the units of intake, portion size definitions, data extractions and inclusion criteria for whole-grain exposure in prior identified meta-analyses of whole grains and T2D^81,82^, we identified and used Reynolds et al.^62^ as the highest-quality meta-analysis for the association between whole grains and T2D risk. Reynold et al. suggest a potential nonlinear relationship between whole-grain intake and log (relative risk), with stronger protective effects for the first 40 g per d of intake and smaller protective effects thereafter. We approximated this nonlinear association by using two linear functions, visually estimated at between 0 and 40 g per d and between 41 and 90 g per d on the log (RR) scale. Specifically, we graphically determined the log (RR) corresponding to the first 40 g per d of whole-grain intake (and corresponding confidence intervals based on the spline curve confidence interval) and the log (RR) and confidence interval corresponding to the following intake of 50 g per d (for example, from 40 g per d to 90 g per d), standardizing these values to units of 30 g per d. Intake of 90 g per d was set as the optimal intake level, as it represents a conservative estimate of the intake level with lowest relative risk based on the estimated spline curve from the cohort study data points.

To estimate the burden of T2D attributable to suboptimal intake of whole grains, we modified the RR(*x*) input function for the standard population-attributable fraction (PAF), detailed below. In review, RR(*x*) is typically modeled as follows for protective dietary factors (that is, when there is no added benefit above the optimal intake level):

$$1:x - y\left(x\right) > 0$$

$${\textrm{exp}}\left( {\beta \left( {x - y\left( x \right)} \right)} \right):x - \left. {y\left( x \right)} \right) \le 0,$$

where *β* is the stratum-specific change in log relative risk per unit of exposure, *x* is the current exposure level, and *y*(*x*) is the optimal exposure level. *y*(*x*) is defined to be $F_{\textrm{optimal}}\left( {F_x^{ - 1}\left( x \right)} \right)$, where *F*_optimal_ is the cumulative distribution function of the optimal intake, and $F_x^{ - 1}$ is the inverse cumulative distribution function of the current exposure distribution. Implicit in how we characterize the relative risk function are some of the fundamental assumptions that we make about relative risk. Namely, that relative risk increases exponentially as distance from the optimal intake exposure level (*y*) increases, that there is no risk associated with exposure beyond the optimal intake exposure level and that both *x* and the optimal intake exposure level for an individual at exposure level *x* are the *q*th quantile of their respective distributions (the observed exposure distribution and the optimal intake distribution, respectively).

To account for the stepwise, nonlinear nature of the log relative risk for whole grains, we modified the RR(*x*) function so that intake between 40 and 90 g per d was evaluated based on the more conservative RR_90_ (0.92 (0.87, 0.94)) only. Intake between 0 and 40 g per d was evaluated based on RR_90_ (0.92 (0.87, 0.94)) for the intake difference of 50 g per d from the optimal intake level (90 g per d) and the further deviation beyond that using the stronger RR (0.81 (0.72, 0.90)), by summing the transformed RR_40_ and RR_90_ values. As previously, there is no risk associated with exposure beyond the optimal intake level of 90 g per d. The revised RR(*x*) for whole grains and T2D was instead modeled as:

$$\begin{array}{*{20}{l}} {\textrm{exp}\left( {\beta _{40}\left( {x - 40} \right) + \beta _{90}\left( {40 - y\left( x \right)} \right)} \right)} \hfill & {:x \le 40} \hfill \\ {\textrm{exp}\left( {\beta _{90}\left( {x - y\left( x \right)} \right)} \right)} \hfill & {:90 \ge x > 40} \hfill \\ 1 \hfill & {:\hat x > 90} \hfill \end{array}$$

### Characterization of optimal intakes

Optimal intake levels serve as the counterfactual in our comparative risk assessment modeling analysis, allowing for comparable quantification of impacts of dietary factors on disease risk at the population level. Optimal intake levels were determined primarily based on disease risk (observed consumption levels associated with lowest disease risk in meta-analyses) with further considerations of feasibility (observed national mean consumption levels in nationally representative surveys worldwide) and consistency with existing major food-based dietary guidelines. Because populations inevitably have a range of consumption levels, we used a normal distribution around each optimal intake level with s.d. equaling 10% of the mean, consistent with optimal distribution ranges of metabolic risk factors^83–85^. For each dietary factor, the comparative risk model assumed no additional health benefits beyond the optimal intake distribution within each stratum. For BMI-mediated effects, no further benefits of BMI reduction were estimated at or below a BMI of 18.5 kg m^−2^ (ref. ^86^).

The optimal intake levels used in this analysis are analogous to what has been termed a theoretical minimum risk exposure level in other analyses^1,53^, but we prefer the term ‘optimal intake’ as more relevant to dietary risk factors than ‘theoretical minimum risk exposure level’. These optimal intakes can be considered a benchmarking to quantifying disease risk and informing policy priorities in different nations. We determined optimal intake levels for dietary factors based on probable or convincing evidence for effects on cardiometabolic outcomes, and these levels were not developed as part of characterizing an overall ideal dietary pattern, which might also consider other factors.

Optimal intakes for whole grains, yogurt, processed meats, unprocessed red meats, SSBs, fruits, non-starchy vegetables, and nuts and seeds were previously calculated^66^; and optimal intakes for potatoes, refined rice and wheat, and fruit juices were estimated de novo using similar methods, detailed in Supplementary Tables 1 and 2^12^.

For potatoes, optimal intake was set at 0 g per d based on observed intake levels associated with lowest risk in studies included in meta-analyses (as low as 13 g per d^87,88^), national mean intakes in 2018 as low as 0 g per d (Laos) and less than 10 g per d for eight other countries (for example, Ghana, Philippines, etc. (http://www.globaldietarydatabase.org/)) and absence of specific recommendations for potatoes and/or grouping of potatoes with starchy staples rather than vegetables in food-based dietary guidelines^89^. For example, the US Dietary Guidelines for Americans, 2020, the Chinese Food Pagoda and the German Nutrition Circle all have general recommendations for total starchy vegetables or tubers for one serving per d or less (https://www.dietaryguidelines.gov)^90,91^. The optimal intake for refined rice and wheat was set at 0 g per d based on observed intake of <1 serving per d among individual of lowest risk in cohorts included in meta-analyses^92^ and national mean intakes of refined grains in 2018 <25 g per d in eight countries; and major dietary guidelines recommend limiting refined grain intake and choosing whole grains and tubers over refined grains (https://www.dietaryguidelines.gov)^89^. For fruit juice, the optimal intake was set at 0 g per d based on observed intake of ‘never’ or ‘rarely’ among individuals of lowest risk for T2D in cohorts included in meta-analyses, national mean intake of fruit juices in 2018 of less than one serving for more than ten countries and national food-based dietary guidelines that either include 100% fruit juice within the fruit category but state that it should not count for more than one serving per day for fruit or explicitly include negative messages about fruit juice and/or group fruit juice with SSBs^89^.

### Global distributions of T2D incidence

Global, regional and national data for T2D were derived from the Global Burden of Disease Study 2019, stratified by nation, age and sex in 1990 and 2018 (ref. ^58^). Overall diabetes was defined as FPG levels greater than 1.25 mg ml^−1^ (7 mML^1^) or being on diabetes medication^93^. T2D was defined as cases of overall diabetes not specified as type 1 (ref. ^94^). Data inputs included estimates of diabetes and mean FPG in a representative population, individual-level data on FPG measures from surveys and US MarketScan insurance claim data^94^. Data on T2D incidence were not available for South Sudan; thus, the entire country was excluded from the present analysis.

### Disaggregation of T2D incidence by education level and urbanicity

We further stratified these estimates of T2D incidence by education level (low, medium, high) and urbanicity (urban, rural) to align these with the demographic and GDD dietary data and enable assessment of heterogeneity in risk within education and urbanicity-based subpopulations (Supplementary Table 13), given evidence that these factors are known to influence both diet and T2D risk in region-specific ways^95–99^. We used the following additional data inputs to reconcile these stratification differences: (1) global population proportions, (2) effect estimates of educational attainment on T2D risk and (3) effect estimates of urban versus rural residence on T2D risk.

Global population proportions for each year were derived from the United Nations Population Division^63^, supplemented with data on education attainment from Barro and Lee^65^. We also further scanned the scientific literature for the latest meta-analysis, pooled analyses and large surveys evaluating the association between sociodemographic factors such as educational attainment and urbanicity with T2D risk. We hypothesized that country income level was a potential effect modifier for both educational attainment and urbanicity on T2D risk, and thus we collated risk estimates stratified by or specific to country income level. We limited our analysis to high-quality risk assessments adjusted for at least age and sex^95–97,100–102^.

For both educational attainment and urbanicity, we conducted fixed-effect meta-analysis of collated effect sizes, stratified by country income level. See Supplementary Table 14 for a full list of study characteristics and effect sizes used in each meta-analysis. Given inconsistent definitions across studies and limited data availability, medium education attainment was assumed to be neutral (that is, RR = 1). We distributed the central estimate of our meta-analyzed risk estimate equally for high versus low education (and urban versus rural residence) by taking the square root and inverse square root of the central estimate of the relative risk. See Supplementary Table 13 for final effect estimates for education level and urbanicity used in disaggregating the T2D-incidence estimates.

The total year–country–age–sex stratum-specific T2D-incidence estimates (mean and 95% UI) were then multiplied by their respective population proportion, education effect and urban effect for each of the six de novo strata to obtain raw, fully proportioned burden estimates and their uncertainty. These values were then scaled to the total stratum burden estimate to prevent underestimation or overestimation of the absolute number of T2D cases globally^103,104^. A fictitious, illustrative example is provided to illustrate how 1,000 T2D cases in a single age–sex population stratum (low-income country) in a given year were disaggregated into the six finer education–urbanicity strata using the central estimate of the meta-analyzed education and urban effects (Supplementary Table 15). The population-proportioned-only burden estimates are also provided as a comparison in the final column.

### Comparative risk assessment analysis, overview

The comparative risk assessment framework incorporated the data inputs and their uncertainty to estimate the absolute number, rate (per million adult population) and proportion of T2D cases attributable to suboptimal intake of each dietary factor in 1990 and 2018 (Supplementary Fig. 1). For each stratum, the model calculated the percentage (PAF) of T2D incidence associated with each dietary factor RR by comparing the present distribution of consumption with the optimal intake distribution. BMI-mediated effects were calculated based on the stratum-specific association of current dietary habits with BMI change, weighted by the prevalence of overweight, normal weight and underweight (no effect) in each stratum, combined with the RR for this BMI change associated with T2D using the same continuous PAF formula. A modified relative risk function, incorporating stepwise, nonlinear log relative risks, was used for the whole-grain direct-effect model given evidence of a nonlinear relationship between whole-grain intake and T2D risk^62^. See sections below for further details on each PAF calculation.

For dietary factors with both direct and BMI-mediated associations with T2D risk, the two stratum-level PAFs were combined into a single joint PAF for that dietary factor using proportional multiplication. The joint association of all 11 dietary factors was similarly estimated using proportional multiplication of each stratum-specific PAF. To consider plausible substitution effects and minimize the overestimation of attributable burdens, the model assumed that half the benefit of whole-grain intake was mediated by replacement of refined grains (rice and wheat). Stratum-level dietary factor and overall joint PAFs were then multiplied by the number of T2D cases in that stratum of the global population to estimate the attributable number of T2D cases in that stratum. Findings were evaluated globally, regionally and by nation and also in subgroups by age, sex, education and urbanicity and were reported as proportional attributable burden (percentage of cases) and attributable rate (cases per 1 million adults).

We also assessed national findings by SDI in 1990 and 2018, a measure of a nation’s development based on a composite average of the rankings of income per capita, average educational attainment and fertility rates^105^.

Uncertainty was quantified using 1,000 multiway probabilistic Monte Carlo simulations, jointly incorporating stratum-specific uncertainties in dietary exposures, underweight and overweight prevalence, and diet–T2D, diet–BMI and BMI–T2D etiologic effect estimates. Corresponding 95% UIs were derived from the 2.5th and 97.5th percentiles of 1,000 estimated models. For comparing trends between 1990 and 2018, we calculated differences for PAFs by subtracting the 1990 value from the corresponding 2018 value for each simulation, reporting the median and 95% UI for each difference. We did not formally standardize comparisons for age or sex over time, so that findings would reflect the actual population differences in attributable burdens that are relevant to policy decisions, but also performed analyses stratified by age and sex that account for changes in these demographics over time. All analyses were performed using R statistical software, R version 4.0.0 (ref. ^106^), and the Tufts High Performance Cluster.

### Direct-effect population attributable fraction

The population attributable fraction (PAF) formula is used to quantify the burden of disease attributable to the difference between a population’s observed exposure and a counterfactual, optimal intake distribution, given an etiologic exposure–disease risk relationship.

We aimed to estimate the burden of T2D incidence attributable to suboptimal intake of protective and harmful dietary factors (for example, lower intake of protective dietary factors and higher intake of harmful dietary factors than their respective optimal intake levels) with direct effects on T2D risk.

The standard PAF formula used is as follows:

$$\frac{{{\int}_{x = 0}^m {\textrm{RR}\left( x \right)P\left( x \right)dx - 1} }}{{{\int}_{x = 0}^m {\textrm{RR}\left( x \right)P\left( x \right)dx} }},$$

where *P*(*x*) is the usual dietary intake distribution in a specific population stratum, assumed to follow a gamma distribution for all dietary factors of interest, as used in previous analyses^9^; RR(*x*) is the age-specific relative risk function for T2D incidence; and *m* is the maximum exposure level.

RR(*x*) is defined as:

$$\left\{ {\begin{array}{*{20}{l}} {{\it{\textrm{exp}}}\left( {\beta \left( {x - y\left( x \right)} \right)} \right)} \hfill & {:x - y\left( x \right) \ge 0} \hfill \\ 1 \hfill & {:x - y\left( x \right) < 0} \hfill \end{array}} \right.,$$

where *β* is the stratum-specific change in log relative risk per unit of exposure, *x* is the current exposure level, and *y*(*x*) is the optimal exposure level. *y*(*x*) is defined to be $F_{\textrm{optimal}}\left( {F_x^{ - 1}\left( x \right)} \right)$, where *F*_optimal_ is the cumulative distribution function of the optimal intake, and $F_x^{ - 1}$ is the inverse cumulative distribution function of the current exposure distribution. Implicit in how we characterize the relative risk function are some of the fundamental assumptions that we make about relative risk. Namely, that relative risk increases exponentially as distance from optimal intake exposure level (*y*) increases, that there is no risk associated with exposure beyond the optimal intake exposure level and that both *x* and the optimal intake exposure level for an individual at exposure level *x* are the *q*th quantile of their respective distributions (the observed exposure distribution and the optimal intake distribution, respectively).

In practice, simple numerical integration using Riemann sums can be used to compute the integrals in the PAF formula, as described in detail in previous work^9^.

$${\textrm{PAF}} = \frac{{\mathop {\sum}\limits_{i = 1}^n {P_i\left( {\textrm{RR}_i - 1} \right)} }}{{\mathop {\sum}\limits_{i = 1}^n {P_i\left( {\textrm{RR}_i - 1} \right)} + 1}}$$

*n* categories are determined by dividing up the exposure range (chosen here to be 0, $F_x^{ - 1}\left( {\Phi \left( { - 6} \right)} \right.$)) into 121 intervals, each of length 0.1 when converted to the standard normal scale (except for the first one). Φ is defined as the cumulative distribution function of the standard normal distribution (N(0,1)). More precisely, the range of exposure groups *I* can be described as:

$$\begin{array}{*{20}{l}} {\left( {0,F_X^{ - 1}\left( {\Phi \left( { - 6} \right)} \right)} \right)} \hfill & {:i = 1} \hfill \\ {\left( {F_X^{ - 1}\left( {\Phi \left( { - 6 + 0.1\left( {i - 2} \right)} \right)} \right)} \right.,\left. {F_X^{ - 1}\left( {\Phi \left( { - 6 + 0.1\left( {i - 1} \right)} \right)} \right)} \right)} \hfill & {:i > 1} \hfill \end{array}.$$

### BMI-mediated effect population attributable fraction

The association of change in BMI with change in dietary intake was assessed using multivariate linear regression for within-person repeated measures, as described in an earlier work^60^. Separate linear relationships were then estimated for BMI < 18.5 kg m^−2^, 18.5–24.9 kg m^−2^ and ≥25 kg m^−2^, given observed effect modification by baseline BMI status, as described and reported in that same prior analysis^60^.

To assess the BMI-mediated effects of suboptimal dietary intake of 11 dietary factors on T2D incidence, we first calculated the monotonic effect of dietary intake on BMI change for each population stratum by weighting the baseline BMI-specific effect by the respective prevalence of underweight, normal weight and overweight within each stratum. We assumed that underweight individuals (BMI < 18.5 kg m^−2^) experienced no change (increase or decrease) in T2D risk with consumption of either risk or protective dietary factors. As such, the monotonic effect for this population segment was set at 0.

Df-to-BMI effect = *β*_BMI≥25_ × (overweight prevalence) + *β*_BMI18.5–25_ × (normal weight prevalence) + 0 × (underweight prevalence)

We then estimated log (RR) per unit-associated increase in exposure for each dietary factor by taking the log (RR) per unit-associated increase in exposure for BMI and multiplying it by the dietary Factor-to-BMI effect (associated increase in BMI per one-unit-associated increase in that dietary factor).

### Joint population-attributable fraction of suboptimal diet

Because summing would overestimate joint relationships, for each stratum, the joint PAF of suboptimal diet (overall, by direct effects and by BMI-mediated effects) was estimated by proportional multiplication as follows:

$${\textrm{PAF}}_{\textrm{joint}} = 1 - \mathop {\prod }\limits_{r = 1}^R \left( {1 - {\textrm{PAF}}_r} \right),$$

where *r* denotes each individual dietary factor, and *R* is the number of dietary factors. The analyses supported independent etiologic relationships of each dietary factor, and joint distributions were further determined within each stratum, maximizing validity of our joint PAFs. Joint distributions of exposure may be partly correlated among individuals, leading to overestimation of joint attributable fractions. Yet, separate prior validity analyses of dietary patterns, including interventional studies, suggested that the estimated etiologic relationships of individual components and their joint associations were reasonable^9^.

### Quantification of uncertainty using Monte Carlo simulations

Monte Carlo simulations were used to quantify uncertainty in the PAFs, incorporating stratum-specific uncertainty in usual dietary intake-distribution parameters, etiologic RR estimates and prevalence of overweight and normal weight. Specifically, for each diet–T2D pair and stratum, we drew randomly 1,000 times from the normal distribution of the estimate of T2D-specific changes in the log (RR) corresponding to a one-unit increase in intake, the predictive distributions for shape and rate parameters for usual dietary intake, and the normal distribution of the estimate of normal weight and overweight. Draws of proportions that were less than 0 or greater than 1 were changed to 0 or 1, respectively. Likewise, draws of mean intake that were zero or less were changed to 0.00001. Each set of random draws are used to calculate the PAFs and associated, absolute attributable T2D burden.

### Reporting summary

Further information on research design is available in the Nature Portfolio Reporting Summary linked to this article.

## Online content

Any methods, additional references, Nature Portfolio reporting summaries, source data, extended data, supplementary information, acknowledgements, peer review information; details of author contributions and competing interests; and statements of data and code availability are available at 10.1038/s41591-023-02278-8.

## Supplementary information

Supplementary Information Supplementary Note 1, Fig. 1 and Tables 1–15

Reporting Summary

## Data Availability

All data used in this analysis are publicly available from the following sources: (1) individual dietary intake estimate distribution data (GDD, Download 2018 Final Estimates: https://www.globaldietarydatabase.org/data-download); (2) stratum-specific global mean BMI, converted to overweight- and underweight-prevalence distribution data (NCD-RisC, Data Downloads: https://ncdrisc.org/data-downloads.html); (3) T2D burden-incidence-estimate distribution data (Global Health Data Exchange, Global Burden of Disease Study 2019 Results Tool: https://vizhub.healthdata.org/gbd-results/); (4) linear, BMI-stratified effects of dietary factors on weight gain or weight loss: ref. ^60^; (5) direct, proportional, age-adjusted effects of BMI on T2D: ref. ^23^; (6) direct, proportional, age-adjusted effects of diet on T2D: whole grains, ref. ^62^; all remaining dietary factors, ref. ^61^; (7) optimal intake levels for dietary factors: ref. ^12^; (8) population demographic data: UN Population Division (age, sex, urbanicity), ref. ^65^; (9) SDI data from Global Health Data Exchange: Global Burden of Disease Study 2019 SDI 1950–2019: https://ghdx.healthdata.org/record/ihme-data/gbd-2019-socio-demographic-index-sdi-1950-2019; (10) FAO Food Balance Sheet data for the energy availability of ‘wheat and products’ and ‘rice and products’ (kcal per capita per d): United Nations FAO: Food Availability Data: http://www.fao.org/faostat/en/#home; (11) global glycemic load estimates for wheat and rice products: ref. ^73^; (12) caloric content per 100 g for wheat and rice products: US Department of Agriculture Agricultural Research Service Food and Nutrient Database for Dietary Studies 2017–2018: https://www.ars.usda.gov/nea/bnrc/fsrg.

## Code availability

Custom code was developed using R (version 4.0.0) with two-tailed *α* = 0.05, for cleaning, merging and formatting of all data inputs; calculation of age-adjusted relative risks; comparative risk assessment modeling, including PAF calculations for each dietary factor separately and joint PAF calculations for all dietary factors; summary aggregation of stratum-level PAF estimates at the global, regional and national levels; and data visualization. Given their computational size and complexity, all comparative risk assessment modeling codes were run on the Tufts University High Performance Computing Cluster (https://it.tufts.edu/high-performance-computing), supported by the National Science Foundation (grant 2018149, https://www.nsf.gov/awardsearch/showAward?AWD_ID=2018149&HistoricalAwards=false) under active development by Research Technology (https://it.tufts.edu/researchtechnology.tufts.edu), Tufts Technology Services. The statistical code used for this analysis is not publicly available. The GDD can make the statistical code available to researchers upon request. Eligibility criteria for such requests include: utilization for nonprofit purposes only, for appropriate scientific use based on a robust research plan and by investigators from an academic institution. GDD will nominate co-authors to be included on any papers generated using GDD-generated statistical code. If you are interested in requesting access to the statistical code, please submit the following documents: (1) proposed research plan (please download and complete the proposed research plan form https://www.globaldietarydatabase.org/sites/default/files/manual_upload/research-proposal-template.pdf), (2) data-sharing agreement (please download this form https://www.globaldietarydatabase.org/sites/default/files/manual_upload/tufts-gdd-data-sharing-agreement.docx, complete the highlighted fields and have someone who is authorized to enter your institution into a binding legal agreement with outside institutions sign the document. Note that this agreement does not apply when protected health information or personally identifiable information are shared), (3) email items (1) and (2) to info@globaldietarydatabase.org. Please use the subject line ‘GDD Code Access Request’. Once all documents have been received, the GDD team will be in contact with you regarding subsequent steps.
