# Supplementary material for: Incident type 2 diabetes attributable to suboptimal diet in 184 countries
Source: Nat Med. 2023 Apr 17;29(4):982–95. doi: 10.1038/s41591-023-02278-8 (PMC10115653; doi:10.1038/s41591-023-02278-8)
Supplement: Supplementary file 1 — Supplementary Note 1, Fig. 1 and Tables 1–15 [file 41591_2023_2278_MOESM1_ESM.pdf]

nature medicine

Article https://doi.org/10.1038/s41591-023-02278-8

Incident type 2 diabetes attributable to suboptimal diet in 184 countries

In the format provided by the authors and unedited

Supplemental Materials

Supplementary Findings

Box S1. Trends in the global and country-specific estimated diet-attributable T2D cases burden, 1990-2018

Supplementary Figures

Figure S1. Example age-specific relative risks for the association between potato intake (g/d) and T2D incidence

Supplementary Tables

Table S1. Dietary Factors in the Analysis, Mean Consumption Levels Among Adults >20 years in 1990 and 2018, Optimal Consumption Levels, and Relative Risk for Diabetes at Age 50 and 70 years, and effect of dietary intake on BMI change
Table S2. Optimal intake levels and corresponding uncertainty for dietary factors with direct effects on T2D risk
Table S3 Absolute burden of T2D incidence attributable to suboptimal diet at the global, world region, and national level in 1990 and 2018
Table S4.Proportion of the diet-attributable T2D burden due to excess intake of harmful vs. insufficient intake of protective dietary factors globally and by world region in 1990 and 2018
Table S5. Proportional and absolute burden per 1M population of T2D incidence attributable to suboptimal diet and each individual dietary factor at the national level in 1990 and 2018
Table S6. Primary data inputs and associated data sources for comparative risk assessment analysis
Table S7. Grading of Evidence of the Association of Specific Dietary Factors with Type II Diabetes and Weight Gain
Table S8. Characteristics of the 1220 dietary surveys that inform the Global Dietary Database 2017
Table S9. Standard serving size, glycemic load per serving size, and standardized glycemic load and caloric content per standardized serving of common cooked/prepared wheat and rice products globally
Table S10. Pooled multivariable-adjusted relationships of changes in dietary intake with change in body mass index among 120,877 US women and men in three prospective cohorts with 12-20 Years follow-up, stratified by body mass index
Table S11. Multi-variable adjusted relative risks and average-age-at-event for the association between diet and lifestyle risk factors and T2D incidence from published meta-analyses
Table S12. Example age-at-event calculation for the association between potato intake (g/d) and T2D risk, based on the studies and associated relative risks included in Quan *et al.* 2020
Table S13. Effect estimates for the association between education level and urbanicity with T2D risk used in disaggregating the T2D incidence estimates
Table S14. Study characteristics and effect estimates from studies assessing the association between education level and urbanicity with T2D risk for disaggregating T2D incidence estimates by education level and urbanicity
Table S15. T2D burden disaggregation for a mock, a single national level age-sex T2D incidence estimate into six education level, urbanicity stratified estimates
.

Box S1. Trends in the global and country-specific estimated diet-attributable T2D cases burden, 1990-2018

The global proportion of estimated diet-attributable T2D increased by 2.6 percentage points, from 67.7% [66.2, 69.4] to 70.3% (68.8, 71.8) **(Figure 1, Figure 5A**), and the absolute number, from 5.5 million (5.3, 5.6) to 14.1 million (13.8, 14.4) (**Table S10**). For 4 dietary factors, improvements were estimated, such as lower burdens for insufficient whole grains (-1.9 [-2.6, -1.3] percentage point reduction in attributable burden) and insufficient non-starchy vegetables (-0.29 percentage points [-0.32, -0.27]) and nuts/seeds (-0.09 percentage points [-0.12, -0.07]). Worsening diet-attributable T2D burdens were estimated for 8 dietary factors, including excess unprocessed red meat (+4.4 percentage points [3.3, 5.5] and sugar-sweetened beverages (+1.2 percentage points [0.5, 1.8]) (**Figure 5A**).

Among the 30 most populous countries, the diet-attributable T2D burden per 1M population increased between 1990 and 2018 in 29 countries (exception: Ethiopia (-111 cases [-246, 41]), with largest increases in Myanmar (+2070 cases [1833, 2344], the United Kingdom (+1909 cases[1636, 2203), and the United States (+17105 cases [1421, 2054]) (**Figure E6**).

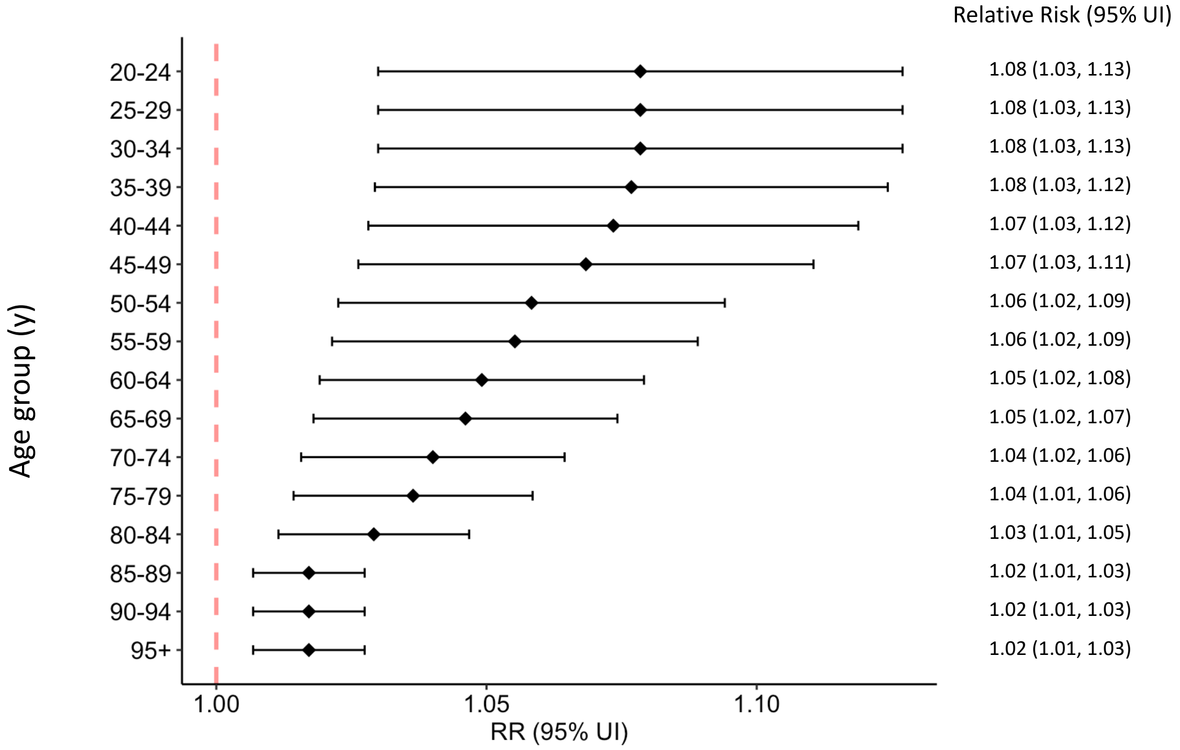

Figure S1. Example age-specific relative risks for the association between potato intake (g/d) and T2D incidence. Age-specific relative risks calculated based on previous evidence demonstrating decreasing proportional effects of metabolic risk factors (i.e., BMI, total cholesterol, etc.) for cardiometabolic disease. Relative risk estimates were interpolated and extrapolated into standard, 5-year age groups, assuming linear relationships between age and log(RR). While relative risks for dietary risk factors on T2D decline with age due to competing risk factors, absolute risk for T2D increase with age.

RR, relative risk; UI, uncertainty interval

Table S1. Dietary Factors in the Analysis, Mean Consumption Levels Among Adults >20 years in 1990 and 2018, Optimal Consumption Levels, and Relative Risk for Diabetes at Age 50 and 70 years, and effect of dietary intake on BMI change

	Global Consumption, g/d (mean, 95% UI)		Optimal Consumption, Mean (g/d)*	Unit of RR and effect estimate†	Diet- T2D Relative Risk (95% CI)		Diet- BMI Effect Estimate (95% CI), kg/m^2 ‡	
	1990	2018			Age 50 y	Age 70 y	Baseline BMI <25	Baseline BMI >25
Fruit	81.9 (78.8 - 85.2)	87.9 (84.9 - 90.8)	300	per 100 g/d	-	-	-0.06 (-0.08, -0.04)	-0.11 (-0.16, -0.06)
Non-starchy vegetables	133.3 (129.8 - 136.7)	209.8 (202.2 - 217.4)	300	per 100 g/d	--	-	-0.03 (-0.04, -0.02)	-0.06 (-0.10, -0.02)
Potatoes	45.5 (42.5 - 49.2)	47.8 (42.7 - 55.2)	0	per 100 g/d	1.06 (1.02 - 1.09)	1.04 (1.02 - 1.06)	0.08 (0.03, 0.12)	0.14 (0.08, 0.21)
Nuts and seeds	3.1 (2.8 - 3.5)	8.6 (7.7 - 9.7)	20.3	per 28.35 g/d			-0.1 (-0.15, -0.04)	-0.14 (-0.25, -0.03)
Refined rice and wheat§	304.8 (264.1 – 363.3)	302.9 (265.1, 354.8)	0	Per 1 serving/d§	1.31 (1.18 - 1.44)	1.21 (1.12 - 1.29)	0.05 (0.01, 0.09)	0.07 (0.03, 0.11)
Whole grains¶	44.9 (40.6 - 49.1)	50.1 (44.2 - 55.2)	90	30g/d	≤40g: 0.78 (0.68-0.88) 41-90g: 0.91(0.87-0.95)	≤40g: 0.84 (0.77-0.92) 41-90g: 0.93 (0.90-0.96)	-0.05 (-0.07, -0.03)	0.08 (-0.10, -0.06)
Processed meats	15.5 (13.5 - 18.4)	16.8 (14.7 - 19.9)	0	21.4 g/d	1.19 (1.17 - 1.22)	1.13 (1.11 - 1.15)	0.06 (0.03, 0.08)	0.07 (0.05, 0.09)
Unprocessed red meat	43.6 (41.8 - 45.4)	56.5 (53.3 - 59.9)	14.3	42.9 g/d	1.18 (1.17 - 1.19)	1.12 (1.11 - 1.13)	0.06 (0.03, 0.08)	0.10 (0.06, 0.14)
Yogurt	23.4 (20.0 - 28.5)	21.2 (18.3 - 25.1)	87.1	244 g/d	0.70 (0.56 - 0.85)	0.78 (0.67 - 0.90)	-0.09 (-0.12, -0.06)	-0.20 (-0.29, -0.11)
Sugar-sweetened beverages	82.9 (79.1 - 87.1)	95.6 (89.1 – 103.0)	0	244 g/d	1.20 (1.14 - 1.26)	1.14 (1.10 - 1.18)	0.09 (0.04, 0.13)	.23 (0.14, 0.31)
Fruit juice	17.7 (16.4 - 19.2)	15.1 (14.0 - 16.4)	0	248 g/d	-	-	0.04 (0.01, 0.07)	0.11 (0.03, 0.18)

* Optimal intake values were previously calculated using reported methods based on risk (observed levels associated with lowest mortality/morbidity in meta-analyses), feasibility (observed national consumption levels globally) and consistency (with other assessments in major dietary guidelines). For each dietary factor, the comparative risk modeling assumed no additional health benefits beyond the optimal intake value within each country, sex, age, education and urbanicity stratum. The population distribution around each optimal population mean was estimated as 10% of optimal intake mean.

† Units vary for each relative risk, but were generally obtains as risk per 1 serving/day and later converted into grams/day to align with dietary intake estimates. These relative risks are based on models estimating a linear relationship between dietary intake and BMI (indirect effects) and a log linear relationship between dietary intake and T2D risk (direct effects), except for whole grains for which we identified evidence for a step-wise, log linear association, with smaller protective effects above 40 g/d intake (direct effects).

‡ Based on pooled results from 3 separate prospective cohort studies who were free of obesity (BMI ≥30) or chronic diseases and with complete data on weight and lifestyle habits at baseline. Independent relations of changes in dietary habits with BMI change were assessed in 4-year periods, using linear regression with robust variance and accounting for within-person repeated measures. Linear BMI changes shown are for 1 serving/day increased consumption. When serving sizes differed for a given dietary factor for the diet-T2D RR and diet-weight gain effect estimate, serving size was standardized to the serving size reported for the diet-T2D RR.

§ Refined rice and wheat were evaluated separately in secondary analyses. Etiologic effects of refined rice and wheat were based on direct effects (per glycemic load) as well as effects on weight gain (per standard serving). Global refined grain intake estimates from the GDD were disaggregated into refined rice vs. wheat intake, based on the proportion of refined rice vs. wheat availabe in each country-year. Based on international tables of glycemic load values, average serving sizes were 50g for refined wheat and 150g for cooked refined rice (due to far higher water weight) and average glycemic load values were 13.1/50 g serving refined wheat intake and 30.3/150 g serving refined rice intake (see **Methods** for further details).

¶ Meta-analyses of the association between whole grain intake and diabetes risk suggest a non-linear, stepwise age-specific relative risk between whole grain intake and log(RR), with stronger protective effects for the first 40 g/d, and smaller protective effects thereafter. The nonlinear association was approximated by using 2 linear functions, visually estimated at between 0-40 g/d, and between 41-90 g/d from the dose-response association found in the literature.

BMI, body mass index; CI, confidence interval; RR, relative risk; T2D, type II diabetes

Table S2. Optimal intake levels and corresponding uncertainty for dietary factors with direct effects on T2D risk

Dietary factor	Optimal intake level*	Standard deviation†
**Dietary factors with direct effects**		
Whole grains	90.0 g/d	9.0 g/d
Yogurt	87.1 g/d	8.7 g/d
Processed meats	0.0 g/d	0.0 g/d
Unprocessed red meats	14.3 g/d	1.4 g/d
Sugar-sweetened beverages	0.0 g/d	0.0 g/d
Potatoes‡	0.0 g/d	0.0 g/d
Refined rice and wheat‡§	0.0 g/d	0.0 g/d
**Additional dietary factors with BMI-mediated effects**		
Fruits	300 g/d	30 g/d
Non-starchy vegetables	300 g/d	30 g/d
Nuts and seeds	20.3 g/d	2.0 g/d
Fruit juices‡	0.0 g/d	0.0 g/d

* Optimal intake values were previously calculated using reported methods for whole grains, yogurt, processed meats, unprocessed red meats, sugar-sweetened beverages, fruits, non-starchy vegetables, and nuts and seeds based on risk (observed levels associated with lowest mortality/morbidity in meta-analyses), feasibility (observed national consumption levels globally) and consistency (with other assessments in major dietary guidelines). For each dietary factor, the comparative risk modeling assumed no additional health benefits beyond the optimal intake value within each country, sex, age, education and urbanicity stratum.
† The population distribution around each optimal population mean was estimated as 10% of optimal intake mean.
‡ Optimal intake levels were defined *de novo* for potatoes, refined grains, and fruit juices based on risk, feasibility and consistency, as these were not reported in previous analyses. For potatoes, optimal intake was set at 0 g/d based on observed intake levels associated with lowest risk in studies included in meta-analyses as low as 13 g/d; national mean intakes in 2018 as low as 0 g/d (Laos) and less than 10 g/d for eight other countries (i.e., Ghana, Philippines, etc.); and absence of specific recommendations for potatoes in food-based dietary guidelines, or grouping potatoes with starchy staples rather than vegetables. For example, the US Dietary Guidelines for Americas 2020, Chinese Food Pagoda, and German Nutrition Circle all have general recommendations for starchy vegetables or tubers for 1 serving/d or less. The optimal intake for refined grains was set at 0 g/d based on observed intake of < 1 serving/d among individual of lowest risk in cohorts included in meta-analyses; national mean intakes of refined grains in 2018 < 25 g/d in eight countries (i.e., Gambia: 4.2 [2.3-8.0] g/d, Norway 13.5 [8.3-22.0], Greece 21.2 [12.8-35.2]); and major dietary guidelines recommend limiting refined grains intake and choosing whole grains and tubers over refined grains. For fruit juice, the optimal intake was set at 0 g/d based on: observed intake of "never" or "rarely" among individuals of lowest risk for T2D in cohorts included in meta-analyses; national mean intakes of fruit juices in 2018 < 1 serving for >10 countries (i.e., China: 0.4 [0.3-0.5] g/d, Nepal: 0.8 [0.4-1.4] g/d, Pakistan: 1.2 [0.7-2.0] g/d, etc.); and national food-based dietary guidelines, which typically include 100% fruit juice within the fruit category, but state it should not count for more than 1 serving/d fruit (and with some countries explicitly delivering negative messages about fruit juice or grouping it with sugar-sweetened beverages).
§ Refined grains intake was modelled as glycemic load for direct effects comparative risk assessment analyses, but as refined grains intake for BMI-mediated analyses. Optimal intake values were considered 0.0 g/d for both.

Table S3. Absolute burden of T2D incidence attributable to suboptimal diet at the global, world region, and national level in 1990 and 2018

	Absolute diet-attributable T2D cases, n (95% UI)	
	**1990**	**2018**
**World**	5471757 (5342213-5620619)	14110070 (13812334-14442328)
**World Region**		
Centr/Eastern Europe and Centr Asia	520887 (504631-536316)	927312 (899744-951383)
High-Income Countries	1218778 (1188049-1252841)	2402184 (2330751-2492579)
Latin Amer/Caribbean	632170 (616773-648984)	1772104 (1730727-1817436)
Mid. East/North Africa	275473 (260757-290556)	1274172 (1202552-1347024)
South Asia	834981 (778168-930735)	2622035 (2454426-2864097)
Southeast and East Asia	1745828 (1627352-1874889)	4208666 (4017428-4421714)
Sub-Saharan Africa	251398 (235302-274025)	878570 (828082-937012)
**Country**		
Afghanistan	11076 (9189-13126)	51903 (43923-60719)
Angola	6793 (6170-7449)	29692 (27329-31985)
Albania	1721 (1502-2002)	5116 (4891-5307)
United Arab Emirates	3605 (3305-3875)	46252 (40064-52505)
Argentina	41266 (39098-43697)	97963 (93053-103387)
Armenia	3682 (3285-4151)	9131 (8551-9630)
Antigua and Barbuda	120 (109-132)	355 (324-383)
Australia	16252 (15038-17537)	43059 (39367-47239)
Austria	10472 (9614-11366)	23528 (21469-25525)
Azerbaijan	6886 (6302-7369)	28212 (26946-29475)
Burundi	2391 (2075-2695)	6526 (5729-7328)
Belgium	14184 (13153-15253)	25021 (23363-26832)
Benin	2316 (1991-2785)	9967 (8591-11766)
Burkina Faso	3720 (3236-4298)	13295 (10902-16692)
Bangladesh	66565 (62877-70097)	202747 (193174-212786)
Bulgaria	16195 (15220-17257)	19368 (17876-20738)
Bahrain	945 (830-1062)	10283 (9060-11473)
The Bahamas	479 (447-512)	1131 (1015-1256)
Bosnia and Herzegovina	4864 (4204-5497)	15287 (13960-16461)
Belarus	10352 (9247-11389)	13083 (11913-14305)
Belize	179 (165-193)	974 (916-1024)
Bolivia	5688 (5380-6019)	20001 (18866-21278)
Brazil	193990 (184665-204552)	532972 (506449-560617)
Barbados	721 (690-751)	1218 (1132-1298)
Brunei	714 (649-773)	2513 (2307-2689)
Bhutan	377 (322-424)	1199 (1031-1337)
Botswana	936 (812-1087)	4089 (3633-4554)
Central African Republic	2122 (1940-2344)	6159 (5598-6767)
Canada	22978 (21759-24405)	62146 (58074-66748)
Switzerland	10187 (9468-11016)	17966 (16553-19496)

Table S3. Absolute burden of T2D incidence attributable to suboptimal diet at the global, world region, and national level in 1990 and 2018, continued

	Absolute diet-attributable T2D cases, n (95% UI)	
	1990	2018
Chile	15267 (13677-16887)	55487 (50529-60590)
China	1153593 (1045191-1272473)	2678203 (2525218-2852833)
Cote d'Ivoire	4716 (4143-5399)	19996 (17808-22519)
Cameroon	4127 (3691-4730)	20816 (18950-22852)
Democratic Republic of the Congo	24228 (21575-27930)	87415 (75158-101659)
Congo	1679 (1437-1982)	7557 (6668-8557)
Colombia	60115 (57679-62505)	149827 (143433-156532)
Comoros	202 (180-224)	556 (496-615)
Cape Verde	142 (121-166)	708 (623-795)
Costa Rica	4389 (4028-4727)	14094 (12934-15297)
Cuba	25916 (24520-27260)	44809 (41710-47663)
Cyprus	1600 (1362-1849)	3569 (3109-4108)
Czech Republic	24858 (22583-27188)	47964 (43005-52542)
Germany	221463 (210605-232619)	344254 (327321-364017)
Djibouti	240 (221-260)	1353 (1272-1438)
Dominica	160 (148-172)	253 (229-281)
Denmark	4745 (4268-5228)	10447 (9368-11631)
Dominican Republic	4446 (4115-4835)	17472 (16275-18832)
Algeria	21537 (19392-23951)	115150 (103204-126282)
Ecuador	8782 (8111-9378)	33628 (30814-36429)
Egypt	30095 (27666-32795)	154120 (142748-168407)
Eritrea	1337 (1203-1461)	5281 (4836-5730)
Spain	85094 (78228-92985)	145222 (131964-159462)
Estonia	2063 (1931-2172)	2759 (2616-2891)
Ethiopia	22678 (20511-26052)	50832 (44592-60203)
Finland	8903 (8290-9554)	16650 (15588-17708)
Fiji	1918 (1717-2103)	4463 (3977-4962)
France	47338 (44832-49999)	89241 (84688-94803)
Federated States of Micronesia	129 (115-142)	397 (355-436)
Gabon	1006 (897-1120)	2758 (2467-3068)
United Kingdom	101414 (95659-107875)	221793 (207579-235951)
Georgia	5940 (5131-6742)	11974 (10932-12860)
Ghana	7806 (7321-8326)	33052 (30541-35638)
Guinea	2592 (2067-3422)	9236 (8178-10412)
The Gambia	479 (428-526)	1656 (1377-1966)
Guinea-Bissau	450 (399-514)	1729 (1604-1855)
Equatorial Guinea	279 (245-313)	1434 (1299-1564)
Greece	12363 (11416-13430)	20855 (19278-22447)
Grenada	159 (149-169)	359 (330-392)
Guatemala	6834 (5978-7868)	46689 (43790-49614)

Table S3. Absolute burden of T2D incidence attributable to suboptimal diet at the global, world region, and national level in 1990 and 2018, continued

	Absolute diet-attributable T2D cases, n (95% UI)	
	1990	2018
Guyana	1736 (1616-1822)	3419 (3192-3611)
Honduras	5152 (4656-5723)	23376 (21720-24874)
Croatia	10057 (9054-11068)	17187 (16489-17770)
Haiti	7871 (7110-8956)	26809 (24081-29721)
Hungary	15942 (14387-17690)	20159 (18278-22424)
Indonesia	120646 (111293-134692)	394290 (355128-448738)
India	616374 (563770-708464)	1856878 (1705109-2093209)
Ireland	2426 (2229-2636)	10001 (9146-11011)
Iran	40940 (38914-43073)	172227 (160665-184905)
Iraq	27273 (25766-28632)	112410 (101794-122198)
Iceland	216 (200-234)	638 (586-692)
Israel	6993 (6660-7295)	22091 (21096-23065)
Italy	120161 (114035-127091)	197559 (187501-209368)
Jamaica	3294 (3069-3524)	8889 (8231-9588)
Jordan	4351 (4039-4719)	28695 (26011-31359)
Japan	172974 (164765-182611)	213126 (202548-224869)
Kazakhstan	21640 (20638-22678)	53085 (50327-55807)
Kenya	7054 (6628-7529)	25004 (23584-26642)
Kyrgyzstan	3009 (2829-3202)	6905 (6385-7476)
Cambodia	5004 (4605-5498)	24558 (22752-26791)
Kiribati	147 (133-162)	461 (416-503)
South Korea	47713 (45169-50457)	112531 (106912-118787)
Kuwait	2699 (2430-3050)	18490 (16905-20306)
Laos	3723 (3473-3966)	13780 (13077-14453)
Lebanon	4166 (3901-4435)	14030 (13062-15004)
Liberia	1544 (1364-1800)	6068 (5456-6695)
Libya	4351 (3901-4842)	23541 (21252-26067)
Saint Lucia	282 (260-305)	763 (699-838)
Sri Lanka	24182 (21874-26759)	96890 (87623-107000)
Lesotho	1150 (1044-1283)	3321 (3015-3627)
Lithuania	3941 (3688-4185)	4636 (4376-4893)
Luxembourg	411 (373-452)	2495 (2255-2642)
Latvia	3279 (3082-3458)	4294 (4027-4539)
Morocco	20672 (18004-23329)	99279 (89511-107763)
Moldova	6396 (5928-6788)	8348 (7591-8971)
Madagascar	5858 (5404-6377)	16744 (15443-18226)
Maldives	148 (134-160)	749 (673-853)
Mexico	179838 (172113-187138)	490341 (468860-511551)
Marshall Islands	98 (89-108)	317 (284-345)
Macedonia	3418 (3111-3755)	9486 (8788-10114)

Table S3. Absolute burden of T2D incidence attributable to suboptimal diet at the global, world region, and national level in 1990 and 2018, continued

	Absolute diet-attributable T2D cases, n (95% UI)	
	**1990**	**2018**
Mali	3448 (2850-4206)	11901 (10429-14015)
Malta	782 (716-850)	1634 (1494-1775)
Myanmar	38280 (33498-43246)	133728 (122310-143859)
Montenegro	1163 (1041-1290)	2497 (2319-2647)
Mongolia	855 (800-904)	3230 (3079-3377)
Mozambique	5127 (4575-5621)	17772 (16146-19767)
Mauritania	1014 (878-1176)	3045 (2675-3430)
Mauritius	2454 (2331-2571)	8460 (7858-9005)
Malawi	4134 (3749-4587)	11146 (9981-12572)
Malaysia	21298 (19025-23709)	69364 (61469-78743)
Namibia	1371 (1229-1532)	3079 (2779-3414)
Niger	1430 (1211-1816)	9455 (8384-10586)
Nigeria	34075 (29136-41863)	100828 (90609-113785)
Nicaragua	4188 (3811-4586)	16697 (15494-17907)
Netherlands	17026 (15894-18293)	26252 (24053-28915)
Norway	6192 (5564-6924)	12012 (10911-13119)
Nepal	10908 (10018-11830)	40724 (37305-44244)
New Zealand	3001 (2741-3273)	7055 (6459-7615)
Oman	1791 (1575-2011)	11495 (10291-12693)
Pakistan	96421 (88910-104136)	333617 (307748-363681)
Panama	3549 (3285-3825)	13012 (12245-13818)
Peru	13587 (12572-14644)	49989 (46554-53504)
Philippines	41280 (38938-43803)	143558 (136292-151336)
Papua New Guinea	6267 (5606-6923)	28449 (26279-30542)
Poland	76695 (73639-79969)	127361 (123460-131980)
Portugal	18505 (17573-19453)	35639 (33872-37569)
Paraguay	3749 (3455-4059)	13791 (12906-14673)
Palestine	1858 (1640-2075)	10907 (9651-12048)
Qatar	986 (854-1110)	17807 (15620-19926)
Romania	34105 (32797-35471)	44248 (42493-46186)
Russia	152675 (143343-160948)	250785 (236356-265288)
Rwanda	2306 (2023-2637)	11035 (10461-11574)
Saudi Arabia	21670 (19095-23872)	138727 (126419-149826)
Sudan	14116 (12644-15646)	64516 (57206-72854)
Senegal	5419 (4667-6331)	19773 (17766-21940)
Singapore	5840 (5163-6466)	14291 (12879-15681)
Solomon Islands	420 (378-464)	1873 (1718-2017)
Sierra Leone	1257 (1021-1542)	5187 (4456-5793)
El Salvador	4830 (4335-5422)	17026 (15767-18204)
Serbia	22737 (20885-24258)	31229 (27380-34757)

Table S3. Absolute burden of T2D incidence attributable to suboptimal diet at the global, world region, and national level in 1990 and 2018, continued

	Absolute diet-attributable T2D cases, n (95% UI)	
	**1990**	**2018**
Sao Tome and Principe	56 (48-67)	200 (178-230)
Suriname	727 (672-791)	2589 (2412-2769)
Slovakia	7224 (6658-7810)	13520 (12621-14326)
Slovenia	3038 (2683-3443)	5176 (4642-5705)
Sweden	11598 (10872-12411)	22577 (21187-24031)
Swaziland	582 (502-688)	1879 (1752-2027)
Seychelles	84 (77-90)	396 (366-428)
Syria	10557 (9445-11857)	34918 (31023-38776)
Chad	2793 (2507-3121)	9307 (8422-10289)
Togo	1035 (857-1272)	6392 (5989-6823)
Thailand	51633 (46189-57599)	155685 (138819-172867)
Tajikistan	3667 (3366-3919)	18850 (17419-20098)
Turkmenistan	2764 (2587-2923)	9299 (8526-9943)
Timor-Leste	363 (334-397)	1807 (1667-1951)
Tonga	156 (140-172)	320 (288-348)
Trinidad and Tobago	3623 (3349-3928)	7717 (7091-8401)
Tunisia	8705 (8060-9526)	36247 (33574-39274)
Turkey	54481 (51033-58464)	160999 (149492-175888)
Taiwan	28813 (26788-30624)	62714 (58735-67069)
Tanzania	7135 (6457-7874)	33554 (30877-36491)
Uganda	6083 (5568-6632)	29190 (26629-32210)
Ukraine	59451 (53540-64303)	67342 (60026-74070)
Uruguay	2234 (2063-2421)	6362 (5867-6835)
United States	498666 (473757-527992)	1096916 (1039351-1176370)
Uzbekistan	12576 (11511-13692)	73154 (68623-77280)
Saint Vincent and the Grenadines	179 (162-197)	566 (538-594)
Venezuela	27009 (24559-29698)	80684 (73382-88461)
Vietnam	43575 (39442-48438)	162189 (145308-178877)
Vanuatu	180 (166-196)	713 (653-776)
Samoa	242 (214-269)	624 (577-680)
Yemen	7163 (6435-8038)	36668 (33098-40172)
South Africa	43738 (41757-45658)	139267 (133121-145198)
Zambia	4659 (4274-5083)	12685 (11625-14056)
Zimbabwe	5213 (4668-5890)	18044 (16215-19923)

Table S4. Proportion of the diet-attributable T2D burden due to excess intake of harmful vs. insufficient intake of protective dietary factors globally and by world region in 1990 and 2018

World Region	dietary factors*	diet-attributable T2D burden, % (mean, 95% UI) 1990	2018
World	harmful	54.7 (52.7-57.1)	58.8 (56.9-60.9)
	protective	39.5 (38.8-40.2)	37.9 (36.9-38.7)
Southeast & East Asia	harmful	44.9 (38.6-51.4)	58.9 (54.4-63.7)
	protective	48.4 (47.4-49.5)	46.4 (45-47.7)
Centr/Eastern Europe & Central Asia	harmful	80.6 (77.5-83.2)	81.8 (78.8-84.3)
	protective	29.8 (27.4-32.4)	29.8 (27-32.5)
High-Income Countries	harmful	65.9 (63.6-68.3)	64 (61.2-67.1)
	protective	36.4 (35.5-37.4)	36.1 (35.1-37.1)
Latin Amer & Caribbean	harmful	65.1 (62.4-67.7)	72.4 (69.8-74.7)
	protective	46.8 (45.1-48.2)	45.7 (43.2-47.6)
Middle East & North Africa	harmful	58 (53.2-62.8)	64.1 (58.5-69.7)
	protective	41.9 (38.3-44.8)	39.3 (34.6-42.6)
South Asia	harmful	44 (39.4-51.8)	42.1 (38.1-49)
	protective	26.6 (24.5-29.1)	26.7 (24.7-29.5)
Sub-Saharan Africa	harmful	44.3 (40-51.6)	56 (51.1-62.3)
	protective	33.9 (30.7-36.7)	36.3 (32.6-39.4)

* Represent the estimated percentage of the diet-attributable T2D burden due to excess intake of six harmful dietary factors jointly (fruit juice, potatoes, processed meats, refined rice and wheat, sugar-sweetened beverages, and unprocessed red meat) and insufficient intake of five protective dietary factors jointly (fruit, non-starchy vegetables, nuts and seeds, whole grains, yogurt). The burden due to excessive intake of the six harmful dietary factors jointly and insufficient intake of the five protective dietary factors jointly were separately estimated using proportional multiplication, and thus should not add up to 100%.

Table S5. Proportional and absolute burden per 1M population of T2D incidence attributable to suboptimal diet and each individual dietary factor at the national level in 1990 and 2018.

Table at the end of the Supplementary Materials due to length.

Table S6. Primary data inputs and associated data sources for comparative risk assessment analysis

Data Inputs	Data source
Population dietary intake estimate distributions* (g/d)	Global Dietary Database (GDD) 2015
Overweight (BMI > 25 kg/m^2) and underweight (BMI <18.5 kg/m^2) prevalence distributions†	NCD-Risk Factor Collaboration
T2D burden incidence estimate distributions‡	Global Burden of Disease (GBD) Study
Linear, BMI-stratified effects of dietary factors on weight gain or weight loss§	Pooled analyses of cohort studies
Direct, proportional, age-adjusted effects of BMI on T2D‖	Pooled analyses of cohort studies
Direct, proportional, age-adjusted effects of diet on T2D¶	Meta-analyses of prospective cohort and RCTs
Optimal intake levels for dietary factors€	Systemic review of etiologic effects of diet on disease risk + de-novo analyses
Population demographic data**	UN Population Division; Baro and Lee 2013

* Dietary factors include: protective – fruits, non-starchy vegetables, nuts & seeds, whole grains, yogurt; harmful factors – fruit juices, potatoes, processed meats, refined grains, sugar-sweetened beverages, unprocessed red meats. The GDD systematically searched for and compiled representative data on individual-level dietary intakes from national surveys and sub-national surveys as previously described. Dietary intake values were adjusted for a 2000 kcal per day diet using the residual method to assess dietary composition, reduce measurement error within and across surveys (i.e. underreporting), and account for potential differences in body size, lean mass, level of physical activity, and metabolic efficiency. Units for each dietary factor were characterized to be consistent with studies providing evidence on etiologic diet-disease relationships. For each dietary factor, a Bayesian hierarchical model incorporating 1000 Monte Carlo simulations estimated the mean intake levels, with 95% uncertainty based on the 2.5^{th} and 97.5^{th} percentiles of the distribution, for each of the 264 strata within 185 countries from 1990 through 2018. Stratum-specific values were combined weighted to the stratum's proportion of the population for global, regional, national, or other subgroup analyses.

† Stratum-level mean BMI estimated based on a Bayesian hierarchical model incorporating more than 2,000 population-based studies of national, regional or global trends in mean BMI, with measurements of height and weight in more than 112 million adults. A Monte Carlo simulation algorithm was used to calculate posterior distributions of mean BMI and its uncertainty, and then converted into overweight and underweight prevalence using established multivariable regression models.

‡ Overall diabetes was defined by GBD as FPG greater than 125 mg/dL (7 mmol/L) or being on diabetes medication. T2D was defined as cases of overall diabetes not specified as type 1. GBD data inputs included estimates of diabetes and mean FPG in a representative population, individual level data on FPG measures from surveys, and US MarketScan insurance claims data.

§ Based on pooled results from 3 separate prospective cohort studies of individuals who were free of obesity (BMI ≥30) or chronic diseases and with complete data on weight and lifestyle habits at baseline. Independent relations of changes in dietary habits with BMI change were assessed in 4-year periods using linear regression with robust variance and accounting for within-person repeated measures. BMI changes shown are for 1 serving/day increased consumption, converted to standardized g/d servings.

‖ Relationships of BMI with incident T2D were obtained from a pooled analysis of multiple cohort studies on the quantitative, age-specific effects of metabolic risk factors on CVD and T2D

¶ Direct relationships of dietary risk factors and T2D were obtained from published systematic reviews and evidence grading, based on meta-analyses of prospective cohort studies and randomized controlled trials including multivariable adjustment for age, sex, BMI, and other risk factors. Age-specific relative risks were calculated for each diet-T2D etiologic relationship based on the mean age-at-event and follow-up duration.

€ Optimal intake levels for each dietary factor were derived from previously reported analyses or calculated de-novo, based on observed levels associated with mortality/morbidity in meta-analyses, feasibility based on observed national consumption levels globally, and consistency with major dietary guidelines

**The GDD obtained annual, national level age, sex, and urbanicity population demographics from the UN population division; and annual national level educational attainment from Baro and Lee 2013 dataset. These values were used to determine the population proportion of each population stratum for informing population weights used in mean dietary intake estimates as well as for reporting summary T2D burden results in the present analysis.

BMI, body mass index; CVD, cardiovascular disease; FPG, fasting plasma glucose; GBD, Global Burden of Disease Study; GDD, Global Dietary Database; NCD, non-communicable disease; RCT, randomized controlled trials; T2D, type II diabetes; UN, United Nations

Table S7. Grading of Evidence of the Association of Specific Dietary Factors with Type II Diabetes and Weight Gain*

Dietary Factor	Outcome	Strength	Consistency	Temporality	Coherence	Specificity	Analogy	Plausibility	Biological Gradient	Experiment
Potatoes	T2D	+	++	+++	+++	+++	++‡	++	+++	+
Refined rice and wheat	T2D	+++	+++	+++	+++	++	+++§	+++	+++	+++
Whole grains	T2D	+++	+++	+++	+++	++	+++¶	+++	+++	+++
Processed meats	T2D	+++	+++	+++	+++	+++	+++**	++	+++	++
Unprocessed red meat	T2D	+++	++	+++	+++	+++	+++**	++	+++	++
Yogurt	T2D	+++	++	+++	+++	+++	++††	++	+++	++
Sugar-sweetened beverages	T2D	++	+++	+++	+++	+++	+++§	+++	+++	+++
	Weight gain†	+++	+++	+++	+++	+++	+++§	+++	+++	+++

*Reproduced from evidence grading conducted in by Miller *et al.* in previous analyses. The 9 Bradford-Hill criteria for grading the evidence were strength, consistency, temporality, coherence, specificity, analogy, plausibility, biological gradient, and experiment. To score each Bradford-Hill criterion, the following general principles were utilized, focusing on evidence from meta-analyses of prospective cohort studies and/or randomized controlled trials: +++ Consistent evidence from several well-designed studies with relatively few limitations; ++ Consistent evidence from several studies but with some important limitations; + Emerging evidence from a few studies or conflicting results from several studies; – criterion not met. Definitions for each of the nine criteria and adaptations to the general scoring system were as follows: **Strength:** magnitude of association, including RRs for protective factors of >0.9 (+), 0.8±0.89 (++), or <0.8 (+++); and for harmful factors, of <1.11 (+), 1.25 (++), and >1.25 (+++). Since magnitude is directly dependent on both the selected serving size and frequency of consumption, we utilized serving sizes most similar to standard dietary guidelines and frequencies of consumption representing modest, standardized differences in intake (e.g., 1 serving/d of fruit) that are easily communicated and could be feasibly achieved by an intervention. **Consistency:** association is repeatedly observed in different populations and circumstances, including ≥80% of included study-specific estimates being in the expected direction (+++); ≥60 - <80% (++); ≥40 - <60% (+); and <40% (not meeting criteria). **Temporality:** exposure precedes outcome. Because all evidence was based on longitudinal studies, this was a necessary criterion (+++); when relatively few overall studies were available (<5), we graded this criterion conservatively as ++. **Coherence:** interpretation of association does not conflict with known natural history and biology of the disease, for example based on pathways of disease occurrence and laboratory findings on the dietary factor. **Specificity:** exposure linked to a specific outcome. Because many nutritional factors can plausibly have diverse effects and influence multiple outcomes, scoring was based on three principles: 1) dietary factor influences a mechanism/pathways known to cause the outcome; 2) dietary factor not associated with multiple other, unrelated non-communicable diseases (e.g., multiple cancers, chronic obstructive pulmonary disease (COPD)); 3) dietary association has additional specificity within the set of cardiometabolic outcomes (coronary heart disease (CHD), stroke, diabetes mellitus). **Analogy:** based on the effects of similar factors on the disease outcome; see detailed footnotes below. **Plausibility:** association supported by one or more credible biological mechanisms. **Biological gradient:** exposure and outcome are related by a monotonic dose-response curve. **Experiment:** association is also supported by evidence from randomized controlled trials on intermediate risk factors (or, less commonly, disease outcomes) plus supportive laboratory studies.
†The strength of evidence for weight gain and SSBs was formally evaluated, but was not completed for other dietary factors in this analysis.
‡Based on analogies to other higher glycemic load carbohydrates in relation to both diabetes mellitus and weight gain (don't want to refer to potatoes as poor-quality carbohydrates)
§ Based on analogies to other poor-quality carbohydrates in relation to both diabetes mellitus and weight gain
¶ Based on analogies with other less-processed foods, dietary fiber, and glycemic load
**Based on analogies to processed meats (or unprocessed red meats), blood ferritin levels, and hemochromatosis
††Based on analogies to probiotics in relation to weight gain
NS, non-starchy; T2D, type II diabetes

Table S8. Characteristics of the 1220 dietary surveys that inform the Global Dietary Database, 2017*

Survey characteristics	Overall	Public surveys†	Private surveys†
Surveys included‡, n	1220	864	356
No. of countries represented, n	188	174	128
Percent of the global population represented in 2015, %	99.0	98.1	87.4
Demographic characteristics by surveys, %§			
Adults (20+ y), %	64.5	57.2	82.3
Age 20-44 y	62.5	56.4	77.2
Age 45-69 y	61.7	56.3	75.0
Age 70+ y	32.8	25.6	50.3
Level of educational attainment, %	30.2	24.7	43.5
Urban vs. rural residence, %			
Both urban and rural	52.2	62.3	27.8
Urban only	4.7	0.7	14.3
Rural only	1.4	0.0	4.8
Information not available	41.7	37.0	53.1
Year of Data collection, %			
1980-1999	36.1	42.0	21.6
2000-2015	63.9	58.0	78.4
Representativeness			
National, No. survey (% of total)	880 (72.1)	664 (76.9)	216 (60.7)
No. countries (% global pop represented) ‖	185 (99.6)	172 (99.6)	95 (84.7)
Sub-national, No. survey (% of total)	207 (17.0)	146 (16.9)	61 (17.1)
No. countries (% global pop represented)	75 (81.6)	45 (67.8)	46 (46.2)
Community, No. survey (% of total)	133 (10.9)	54 (6.3)	79 (22.2)
No. countries (% global pop represented)	48 (70.2)	19 (35.1)	36 (48.2)
Response rate, %			
60-100%	37.8	24.8	69.4
20-59%	3.2	1.2	8.1
<20%	5.7	8.0	0.0
Information not available	53.4	66.1	22.5
Sampling methodology, %			
Probability sampling, w/ survey weights	38.4	39.5	36.0
Probability sampling, no survey weights	32.7	32.9	32.3
Non-probability sampling	4.2	0.7	12.6
Information not available	24.7	27.0	19.0
Median (5, 95 pctl) no. of GDD dietary factors/survey	6.0 (1.0, 30.0)	3.0 (1.0, 14.0)	11.0 (2.0, 47.0)
Dietary assessment method¶			
Single or multiple recall/records			
No. surveys (% total)	286 (23.4)	96 (11.1)	190 (53.4)
No. countries (% global pop represented)	68 (78.0)	90 (40.4)	64 (84.7)
Food Frequency Questionnaire			
No. surveys (% total)	503 (41.2)	346 (40.0)	157 (44.1)
No. countries (% global pop represented)	164 (94.3)	141 (89.50	89 (57.2)
DHS questionnaire			
No. surveys (% total)	193 (15.8)	190 (22.0)	3 (0.8)
No. countries (% global pop represented)	70 (53.9)	69 (53.4)	2 (1.7)
Household survey			
No. surveys (% total)	78 (6.4)	78 (9.0)	0 (0.0)
No. countries (% global pop represented)	27 (15.1)	27 (15.3)	0 (0.0)

Biomarker (urine sodium, hemoglobin)			
No. surveys (% total)	160 (13.1)	154 (17.8)	6 (1.7)
No. countries (% global pop represented)	60 (70.7)	59 (71.3)	5 (2.5)

* Table adapted from Miller *et al.* 2021. Number of surveys for each dietary factor varied – this table presents the overall data available

† Public surveys are those retrieved from publicly available databases; private surveys are non-publicly available surveys that are contributed by a data owner (corresponding member)

‡ Each survey count represents a country-specific survey year. When data collection for a single survey was performed over multiple years, the median survey year was used (or first year if 2 years)

§ Because data on urban/rural residence, education level and response rate were not collected in GDD 2010 (41.7% of total surveys), these percentages may underestimate available data in these surveys. Values are shown for surveys including data on that subgroup and may sum to greater than 100% because a survey can include multiple subgroups

‖ While 188 countries had survey data available, 3 countries were dropped from the analysis because FAO food availability data was not available, a crucial covariate in the modeling estimates

¶ Based on the food groups collected in GDD 2010 (up to 21, 41.7% of surveys) and GDD 2017 (up to 54, 58.3% of surveys), not including biomarker surveys

DHS, Demographic Health Survey; GDD, Global Dietary Database

Table S9. Standard serving size, glycemic load per serving size, and standardized glycemic load and caloric content* per standardized serving of common cooked/prepared wheat and rice products globally

Food product	Serving size (g)	GL (g) per serving size	GL (g) per standard serving†	Energy (kcal) per standard serving†
WHEAT				
enriched white bread, Wonder (USA)	30	11	18.3	135
digestive cookies (UK)	25	8	16.0	203
plain crackers (UK)	25	8	16.0	255
spaghetti classici (Voiello, Italy)	180	20	5.6	68.5
wheat tortilla (Mexico) ‡	50	8	8.0	153
bagel, white (USA)‡	70	24	17.1	132
traditional baguette (France)	30	10	*16.7*	136
pita bread, white, mini (UK)	30	10	16.7	137.5
instant 2 min Maggi noodles (New Zealand)‡	180	12	3.3	221.5
		**AVERAGE:**	**13.1**	**160.2**
RICE				
medium grain white rice, boiled 12 min (Australia)	150	34	34.0	193.5
basmati , white, boiled (Australia)	150	26	26.0	193.5
basmati, parboiled long grain rice (India)	150	23	23.0	193.5
white rice, Surti Kolam variety, cooked in rice cooker for 32 min (India)	150	35	35.0	193.5
sticky rice, Thai, cooked for 10 min (UK)	150	41	41.0	144
rice noodles, freshly made, boiled (Australia)	180	16	13.3	160.5
Arborio, risotto rice (Italy)‡	150	16	16.0	195
Rice porridge, NS (Australia)	150	40	40.0	49.5
jasmine rice (Double FP Thai Hom Mali premium quality fragrant rice, Thailand)	150	41	41.0	193.5
basmati rice (Singapore)	150	28	28.0	181.5
basmati, easy cook (heat treated white, polished basmati rice), cooked 15 min (UK)	150	36	36.0	181.5
		**AVERAGE:**	**30.3**	**170.9**

* glycemic load (g per 2000 kcal diet) and serving sizes (g) sourced from Atkinson *et al.* Wheat and rice products were selected to represent the range of commonly consumed wheat and rice products globally. Caloric content (kcal per 100 g) were sourced from USDA FNDDS 2017-18

†standard serving size for wheat assumed as 50 g, and for rice as 150 g, based on reviewing the range of available wheat and rice products

‡ food product serving size and corresponding glycemic load values sourced from Atkinson *et al.* 2008, as they were not provded in the updated Atkinson *et al.* 2021 glycemic load tables.

GL, glycemic load

Table S10. Pooled multivariable-adjusted relationships of changes in dietary intake with change in body mass index among 120,877 US women and men in three prospective cohorts with 12-20 Years follow-up, stratified by body mass index*

Dietary factor†	Body mass index 18-25 kg/m^2	Body mass index ≥25 kg/m^2
Fruits, 100 g/day	-0.06 (-0.08, -0.04)	-0.11 (-0.16, -0.06)
Non-starchy vegetables, 100 g/day	-0.03 (-0.04, -0.01)	-0.06 (-0.09, -0.02)
Nuts and seeds, 28.35 g/day	-0.10 (-0.16, -0.05)	-0.14 (-0.25, -0.03)
Yogurt, 245 g/day §	-0.09 (-0.12, -0.06)	-0.20 (-0.29, -0.11)
Potatoes/fries, 186 g/day	0.14 (0.05, 0.22)	0.27 (0.15, 0.38)
Whole grains, 30 g/day ¶	-0.05 (-0.07, -0.03)	-0.08 (-0.10, -0.06)
Refined grains, 1 serving/day‡	0.05 (0.01, 0.08)	0.07 (0.03, 0.11)
Sugar sweetened beverages, 248 g/day	0.09 (0.05, 0.14)	0.23 (0.14, 0.32)
100% fruit juice, 248 g/day	0.04 (0.01, 0.07)	0.11 (0.03, 0.18)
Processed meats, 50 g/day	0.13 (0.07, 0.19)	0.16 (0.11, 0.21)
Unprocessed red meats, 100 g/day	0.13 (0.07, 0.20)	0.23 (0.14, 0.32)

*Based on pooled results from 3 separate prospective cohort studies, including 50,422 women in the Nurses Health Study (1986-2006), 47,898 women in the Nurses Health Study2 (1991-2003), and 22,557 men in the Health Professionals Follow-up Study (1986-2006) who were free of obesity (BMI ≥30) or chronic diseases and with complete data on weight and lifestyle habits at baseline. Women who became pregnant during follow-up were excluded from the analysis. Independent relations of changes in dietary habits with BMI change were assessed in 4-year periods over 20 years in the Nurses Health Study, 12 years in the Nurses Health Study2, and 20 years in the Health Professionals Follow-up Study, using linear regression with robust variance and accounting for within-person repeated measures.

†BMI changes shown are for 1 serving/day increased consumption. Decreased consumption would be associated with the inverse BMI change. Serving sizes have all be converted to standardized grams per day servings. All results are adjusted for all of the dietary factors in the table simultaneously as well as for other dietary factors (whole-fat dairy foods, low-fat dairy foods, potato chips, diet soda, sweets/desserts, trans fats, fried foods at home, fried foods away from home), age, baseline body mass index at the beginning of each 4-year period, sleep duration, and changes in physical activity, alcohol use, television watching, and smoking.

‡Refined grains average serving size vary by rice (150 g) vs. wheat (50 g), primarily due to water weight. To account for these differences, refined grains intake was disaggregated into rice and wheat intake based on the availability rice vs. wheat as a proportion of total refined grains, based on FAO food balance sheet estimates (kcal/capita/day). The estimated rice and wheat intakes were then assessed separately for the BMI-mediated CRA. See Methods for further methodological details.

§Whole-fat dairy and low-fat dairy were also evaluated in the full multivariable-adjusted model in place of the disaggregated sub-types (e.g., butter, cheese, yogurt). Separate information on fat content or flavoring of yogurt was not available in most years.

¶Findings were similar when either total dietary fiber or cereal fiber were evaluated in the analysis instead of whole grains

Table S11. Multi-variable adjusted* relative risks and average-age-at-event for the association between diet and lifestyle risk factors and T2D incidence from published meta-analyses

Risk factor	Relative risk unit	Relative risk	Average age-at-event (y)†	Data source
potatoes	100 g/d	1.05 (1.02, 1.08)	61.07	Quan 2020
whole grains‡	30 g/d	≤40 g: 0.81 (0.72, 0.90)<br>41-90g: 0.92 (0.87, 0.94)	60.05	Reynolds 2019
yogurt	244 g/d	0.74 (0.60, 0.86)	62.07	Gijsbers 2016
glycemic load§	80 g/d in 2000 kcal	1.26 (1.15, 1.37)	62.25	Livsey 2019
processed meat	150 g/week	1.16 (1.14, 1.18)	63.14	Zeraatkar 2019
unprocessed red meat	300 g/week	1.15 (1.14, 1.16)	60.86	Zeraatkar 2019
sugar-sweetened beverages	244 g/d	1.19 (1.13, 1.24)	55.23	Qin 2020
BMI	1 kg/m^2	1.18 (1.15, 1.21)	57.00	Singh 2013

*The majority of reported relative risks in meta-analyses included adjustments for BMI. Thus, we separately analyzed the BMI-mediated effect of dietary intake on T2D risk.

†Average age-at-event calculated *de novo* from calculated a weighted average of the sum of the average baseline age and ½ the maximum follow-up time (or 2/3 the mean or median follow-up time was maximum not reported) for each study included in meta-analysis. Average age-at-event used for calculating age-specific risks to account for effect modification by age for each diet-disease pair

‡Dose-response meta-analysis of the association between whole grains suggests a non-linear, step-wise relative risk with stronger protective effects for the first 40 g/d of intake, and diminishing protective effects for intakes greater than 40 g/d. Risk assessed relative to optimal intake level See Methods for further details.

§Refined grains intake estimates from GDD were converted to glycemic load estimates to match available relative risk estimates. See Methods for detailed conversion methodology.

‖ RR units were 5 kg/m^2 in original publication, and transformed (logRR divided by 5) to better reflect a 1 unit change in BMI for the present analysis.

Table S12. Example age-at-event calculation for the association between potato intake (g/d) and T2D risk, based on the studies and associated relative risks included in Quan *et al.* 2020

study	weighting*	total weight	weighting fraction	age at baseline (y)	follow-up time (y)	follow-up time reported	0.5(FU max); 0.67(FU mean)	age at event (y)	weighted average age at event (y)
Halton 2006	15.6	18.16	0.18	46.5	20	max	10.00	56.5	10.26
Montonen 2005†	6.8	7.92	0.08	54.5	23	max	11.50	66	5.22
Farhadnejad 2018	2.7	3.14	0.03	44.5	6	max	3.00	47.5	1.49
Muraki 2016	11.4	13.27	0.13	52.5	26	max	13.00	65.5	8.69
Muraki 2016	9.5	11.06	0.11	33.5	20	max	10.00	43.5	4.81
Muraki 2016	9.2	10.71	0.11	57.5	24	max	12.00	69.5	7.44
Liu 2004†	12.8	14.90	0.15	67.5	8.8	mean	5.87	76.3	11.37
Hodge 2004	17.9	20.84	0.21	54.5	4	max	2.00	56.5	11.77
Total	85.9	100	1						**61.07**

*von Ruesten *et al.* 2013 removed from dose-response total potatoes meta-analysis, so weighting needed to be rescaled

†Where discrepancies exist between follow-up time reported in original article versus meta-analysis, value reported in original article was used. Montonen *et al.* 2005 reports maximum follow-up time of 23 y, whereas Quan *et al.* 2020 meta-analysis reports 6 y follow-up. Similarly, Liu *et al.* 2004 reports mean follow-up time of 8.8 y, whereas Quan *et al.* 2020 meta-analysis reports 10 y follow-up.

Table S13. Effect estimates for the association between education level and urbanicity with T2D risk used in disaggregating the T2D incidence estimates*

	EDUCATION LEVEL EFFECTS‡				URBANICITY EFFECTS		
**Country income level†**	**RR (high v. low education)**	***High***	***Medium***	***Low***	**RR (urban v. rural)**	**Urban**	**Rural**
low	1.50 (1.32, 1.71)	*1.23*	*1*	*0.82*	1.65 (1.28, 2.12)	1.29	0.78
lower middle	1.39 (1.32, 1.47)	*1.18*	*1*	*0.85*	1.20 (1.10, 1.30)	1.09	0.91
upper middle	0.99 (0.88, 1.12)	*1.00*	*1.00*	*1.00*	1.31 (1.11, 1.55)	1.15	0.87
high	0.71 (0.64, 0.79)	0.84	1	1.19	1.06 (1.01, 1.12)	1.03	0.97

* We identified meta-analyses, pooled analyses, and large surveys evaluating the association between sociodemographic factors such as educational attainment and urbanicity with T2D risk. We limited our analysis to high quality risk assessments adjusted for at least age and sex, to avoid the attenuating effects of adjusting for additional covariates. Fixed effects meta-analyses of collated effect sizes were conducted, stratified by country income level. Given inconsistent definitions across studies and limited data availability, medium education attainment was assumed to be neutral (i.e., RR = 1). We distributed the central estimate of our meta-analyzed risk estimate for high vs low education (and urban vs. rural residence) by taking the square root and inverse square root of the central estimate of the relative risk, thus assuming equidistance from the high to medium level and from medium to low level (ref). We tested distributing the central estimate by incorporating information on the actual distance from high to medium education and medium to low education, when available. As the effects did not differ, we used the square root and inverse square root to keep consistency across studies.

† Based on World Bank Country Income group classification

‡ GDD definition of education level definition as follows- Low (Primary) : 0 - < 6 years education completed; Medium (Secondary): 7-11 years education completed; High (Tertiary): ≥12 years education completed.

RR, relative risk; T2D, type II diabetes

Table S14. Study characteristics and effect estimates from studies assessing the association between education level and urbanicity with T2D risk for disaggregating T2D incidence estimates by education level and urbanicity

Study	Study design	T2D outcome*	T2D outcome ascertainment	Exposure definition†	Covariates¶	Country income**	Sample size	Effect estimate (RR (95% CI))
**EDUCATION LEVEL**								**High vs. Low education**
Dagenais et al 2016	PURE (Prospective Urban and Rural Epidemiological Study): cross- sectional surveys across World Bank income group countries	prevalence	blood glucose or self-report	primary completed vs. college/university completed; primary completed vs. secondary completed‡	age, sex, BMI, waist-hip ratio, phys. Activity level, diet quality (aHEI), smoking, education level, family history of diabetes	high income	3 countries; n = 14757	0.78 (0.63, 0.99)
						upper middle income	7 countries; n = 26088	0.83, (0.71, 0.96)
						lower middle income	4 countries; n = 55430	0.96 (0.85, 1.08)
						low income	4 countries; n = 23391	1.02 (0.83, 1.27)
Seiglie et al. 2020	pooled 29 nationally representative surveys	prevalence	blood glucose or medication usage	primary completed vs. no formal education; secondary completed vs. no formal education§	age and sex only	upper middle income	11 countries; n = 36417	1.34 (1.10, 1.62)
						lower middle income	8 countries; n = 524983	1.54 (1.45, 1.63)
						low income	10 countries; n = 27174	1.86 (1.59, 2.17)
Agardh et al. 2011	meta-analysis	incidence	varied by study (self-report, blood glucose, or medical records)	primary school completed to university degree (high) vs. primary not completed (low)	at min. age + sex, residence; >50% of studies also adjusted for well-established outcome related risk factors	high income	18 studies	0.69 (0.61, 0.78)
**URBANICITY**								**Urban vs. Rural**
Dagenais et al 2016	PURE (Prospective Urban and Rural Epidemiological Study): long-term cross sectional survey World Bank income group countries	prevalence	blood glucose or self-report	urban vs. rural	age, sex, BMI, waist-hip ratio, phys. Activity level, diet quality (aHEI), smoking, education level, family history of diabetes	high income	3 countries; n = 14757	1.07 (0.81, 1.40)
						upper middle income	7 countries; n = 26088	1.20 (0.97, 1.49)
						lower middle income	4 countries; n = 55430	1.20 (1.02, 1.41)
						low income	4 countries; n = 23391	1.65 (1.28, 2.12)
Den Braver et al. 2018	meta-analysis	prevalence or incidence	blood glucose or self-report	urban vs. rural	all age-sex adjusted ; some studies also adjusted for BMI, phys. Activity, etc.	upper middle income	n=8 studies	1.49 (1.16, 1.92)
						lower middle income	n=7 studies	1.45 (1.20, 1.74)
O'Connor et al. 2012	United States Behavioral Risk Factor Surveillance System (BRFSS) study	prevalence	self-report	rural vs. urban ‖	HH income, education, age, sex, BMI, race/ethnicity	high income	n=214698	1.06 (1.01, 1.12)

Corsi et al. 2019	India National Family Health Survey 4	prevalence	blood glucose or medication usage	urban vs. rural	age, sex, smoking and alcohol, wealth, education, social caste	lower middle income	n=718597	1.12 (1.01, 1.25)

*Relative risk difference estimates based on T2D prevalence are assumed to be generalizable to diabetes incidence, the outcome of interest in this comparative risk assessment analysis.

†GDD definition of education level definition as follows- Low(Primary) : 0 - < 6 years completed; Medium (Secondary): 7-11 years completed; High (Tertiary): ≥12 years completed.

‡ effect estimates reported by Dagenais *et al.* were inverted to assess high vs. low education. The education level definitions were otherwise the same as that for GDD.

§ The categories "no formal education" and "primary education" of Seiglie *et al.* fall into the GDD's definition of low education. To combine these categories, we first calculated the ratio of "secondary education vs. no formal education" to "primary education vs. no formal education" to obtain the effect of "secondary education vs. primary education". Then, we pooled the later with the effect of "secondary education vs. no education" and obtained the effect of "secondary education vs. primary + no formal education" that most closely matches or GDD definition of high vs. low education. The ratio of the effect estimates was calculated using the following equation, where ES_1 is the effect estimate for primary vs. no formal education; ES_2 is the effect estimate for secondary vs. no formal education; and $Var(ES_1)$ and $Var(ES_2)$ are the respective standard errors for each effect estimate: $ES_1/ES_2 = exp(ln(ES_1) - ln(ES_2)$ and $SE(ES_1/ES_2)$ and $Var(ES_1/ES_2) = ES_1/ES_2 \sqrt{Var(ES_1)^2/ES_1^2 + Var(ES_2)^2/ES_2^2}$.

‖ Effect estimate was inverted to standardize to urban vs. rural comparison

¶ The aim of collating these studies was to partition Global Burden of Disease study age-sex stratified T2D incidence rates further into education level and urban/rural residence finer stratifications (not to determine the causal association between education level and urban rural residence with T2D risk). As such, the ideal effect estimates for this analysis were only age and sex adjusted, to avoid the attenuating effects of adjusting for additional covariates.

**Based on World Bank Country Income group classification

Table S15. T2D burden disaggregation for a mock, a single national level age-sex T2D incidence estimate (low-income country) into six education level, urbanicity stratified estimates

Educational level†	Urbanicity	total stratum T2D burden estimate	mock population proportion	Education effect	Urban effect	Raw, fully proportioned burden estimate	Scaled, fully proportioned burden estimate	***Population proportional only cases***
Low	Urban	1000	0.2	0.82	1.29	212	229.9	*200*
Medium	Urban	1000	0.1	1.00	1.29	129	140.2	*100*
High	Urban	1000	0.1	1.23	1.29	159	172.5	*100*
Low	Rural	1000	0.4	0.82	0.78	256	278.1	*400*
Medium	Rural	1000	0.15	1.00	0.78	117	127.2	*150*
High	Rural	1000	0.05	1.23	0.78	48	52.1	*50*
	Total:		*1*			*920*	1000	*1000*

The total year-country-age-sex stratum-specific T2D incidence estimates were then multiplied by their respective population proportion, education level effect, and urbanicity effect for each of the six, de-novo* strata to obtain raw, fully proportioned burden estimates. These values were then scaled to the total stratum T2D burden estimate to prevent under- or overestimation of the absolute number of T2D cases globally.

†GDD definition of education level definition as follows- Low(Primary) : 0 - < 6 years completed; Medium (Secondary): 7-11 years completed; High (Tertiary): ≥12 years completed.

T2D, type II diabetes

Table S5. Proportional and absolute burden per 1M population of T2D incidence attributable to suboptimal diet* and each individual dietary factor† at the national level in 1990 and 2018

Country	Dietary factor	Proportional burden, %		Rate, per 1M population‡	
		1990	2018	1990	2018
Afghanistan	Suboptimal diet	63.1 (52.5-74)	60 (51.1-70.1)	2169 (1799-2570)	3101 (2624-3627)
	Whole grains	4.8 (2-11.4)	27.4 (15.3-37.7)	164 (70-390)	1425 (782-1951)
	Refined grains (total)	38.2 (24.1-54.3)	28.5 (17.5-43.8)	1315 (843-1869)	1470 (913-2277)
	Refined rice	9.4 (5.3-15.2)	3.9 (2.2-7)	323 (186-523)	199 (113-359)
	Refined wheat	32.5 (20.2-47.5)	25.8 (15.8-40.5)	1123 (703-1633)	1335 (822-2104)
	Processed meats	3.5 (0.7-17)	2.9 (0.5-14.9)	122 (23-574)	148 (28-770)
	Unprocessed red meats	16.7 (9.6-26.6)	3.2 (1.5-8)	575 (334-909)	167 (76-417)
	Yogurt	12 (10.7-13.2)	13.4 (12-14.6)	413 (363-454)	692 (615-763)
	Sugar-sweetened beverages	0.6 (0.3-1.8)	10 (5-19.2)	22 (10-60)	520 (259-995)
	Potatoes	5.5 (2.7-12.2)	2.1 (1-5.2)	190 (91-419)	109 (51-264)
	Fruit	2.8 (2.4-3)	3.7 (3.3-4)	95 (83-105)	189 (169-211)
	Nuts and seeds	1 (0.7-1.2)	0.8 (0.5-1.1)	34 (24-41)	40 (27-56)
	Non-starchy vegetables	0.8 (0.5-1.1)	1.6 (1.2-1.9)	28 (18-39)	81 (62-98)
	Fruit juice	0 (0-0)	0 (0-0)	0 (0-1)	1 (0-2)
Albania	Suboptimal diet	63.6 (55.5-73.8)	94.1 (91.2-96.3)	911 (795-1060)	2371 (2267-2459)
	Whole grains	4.5 (2.7-7.9)	28.5 (22-33.7)	64 (39-112)	718 (555-849)
	Refined grains (total)	26 (21.2-31.2)	25.6 (20.8-30.7)	374 (304-449)	644 (524-776)
	Refined rice	1.6 (1.3-2.3)	3 (2.3-4)	24 (19-33)	76 (59-101)
	Refined wheat	24.8 (20.1-29.7)	23.3 (18.9-28.1)	356 (289-429)	587 (475-710)
	Processed meats	33.8 (20.5-51.2)	63.2 (48.5-75.2)	486 (298-729)	1593 (1213-1889)
	Unprocessed red meats	2.3 (1.3-4.6)	26.5 (21.8-31.8)	33 (19-66)	669 (551-805)
	Yogurt	7.2 (5-9.1)	6.6 (4.3-8.4)	103 (71-131)	165 (109-213)
	Sugar-sweetened beverages	5.2 (3.4-8.3)	53.1 (44.5-62.9)	75 (49-119)	1342 (1120-1578)
	Potatoes	4.1 (2.4-7)	15.5 (9.7-23)	58 (35-102)	392 (243-579)
	Fruit	3.7 (3.5-3.8)	2.3 (1.9-2.6)	53 (50-56)	57 (49-66)
	Nuts and seeds	1.3 (1.3-1.4)	1.1 (0.9-1.2)	19 (18-21)	27 (23-31)
	Non-starchy vegetables	1.4 (1.3-1.6)	1.1 (0.9-1.3)	21 (19-23)	28 (23-33)
	Fruit juice	0.2 (0.1-0.3)	0.1 (0.1-0.2)	2 (1-4)	3 (2-5)
Algeria	Suboptimal diet	66.8 (60.6-74.3)	76.7 (68.9-82.6)	1838 (1655-2044)	4319 (3871-4737)
	Whole grains	33 (25.9-38)	29.4 (20-36.4)	907 (707-1056)	1650 (1127-2065)
	Refined grains (total)	18.3 (11.1-27.5)	39.5 (27.2-51.9)	503 (310-750)	2239 (1556-2945)
	Refined rice	0.2 (0.1-0.4)	1.6 (1-2.5)	6 (4-11)	91 (58-140)
	Refined wheat	18.2 (11-27.3)	38.6 (26.6-50.9)	498 (307-744)	2191 (1520-2889)
	Processed meats	15.1 (6.3-31.3)	13.2 (5.4-28.3)	415 (173-863)	744 (307-1584)
	Unprocessed red meats	9.4 (5.8-14.2)	15.1 (9.7-22.2)	260 (158-395)	850 (550-1266)

Table S5. Proportional and absolute burden per 1M population of T2D incidence attributable to suboptimal diet* and each individual dietary factor† at the national level in 1990 and 2018

Country	Dietary factor	Proportional burden, %		Rate, per 1M population‡	
		1990	2018	1990	2018
	Yogurt	9.5 (6.8-11.3)	8.9 (6.2-11)	260 (185-312)	501 (345-620)
	Sugar-sweetened beverages	20.8 (14.4-29.7)	20.1 (13.3-28.9)	570 (397-815)	1127 (742-1644)
	Potatoes	2.4 (1.3-4.1)	7.8 (4.4-13.1)	65 (37-114)	441 (248-748)
	Fruit	2.9 (2.6-3.1)	3.1 (2.8-3.4)	80 (72-86)	176 (156-195)
	Nuts and seeds	1.3 (1.3-1.4)	1.2 (1-1.4)	37 (34-39)	68 (57-77)
	Non-starchy vegetables	1 (0.8-1.2)	0.6 (0.4-0.9)	27 (22-32)	36 (23-49)
	Fruit juice	0.1 (0.1-0.3)	0.1 (0.1-0.3)	4 (2-7)	7 (4-14)
Angola	Suboptimal diet	60.8 (55.6-66.7)	73.6 (67.7-78.9)	1373 (1247-1506)	2262 (2082-2437)
	Whole grains	29.5 (21.6-35.7)	23.3 (14.6-31.2)	666 (486-802)	713 (446-959)
	Refined grains (total)	21 (16.4-26.1)	17.5 (12.9-22.9)	474 (372-594)	537 (397-701)
	Refined rice	12.9 (9.9-16.4)	5 (3.6-7.1)	291 (224-373)	153 (111-217)
	Refined wheat	9.8 (7.5-12.7)	13.4 (9.8-18)	222 (169-287)	413 (302-552)
	Processed meats	5.6 (1.8-16)	2.3 (0.7-8)	126 (41-362)	71 (21-242)
	Unprocessed red meats	7.3 (5.1-10.3)	8.4 (5.9-12.6)	164 (115-234)	259 (180-388)
	Yogurt	12.3 (11.1-13.4)	12.4 (11.2-13.5)	278 (250-303)	383 (342-418)
	Sugar-sweetened beverages	15.7 (11-22.5)	29.5 (20.7-38.8)	356 (245-507)	907 (638-1193)
	Potatoes	2.3 (1.3-4.3)	29.8 (20.1-38.9)	52 (29-96)	923 (626-1198)
	Fruit	3.3 (3.1-3.5)	2.6 (2.4-2.9)	74 (70-79)	81 (72-89)
	Nuts and seeds	1.3 (1.1-1.4)	1 (0.9-1.2)	28 (26-31)	32 (26-38)
	Non-starchy vegetables	1 (0.9-1.2)	0.3 (0.2-0.5)	23 (20-27)	10 (6-15)
	Fruit juice	0.1 (0.1-0.2)	0.1 (0.1-0.2)	2 (1-4)	3 (2-6)
Antigua and Barbuda	Suboptimal diet	76.3 (69.8-83.2)	80.5 (73.9-86.5)	3187 (2897-3491)	5238 (4789-5661)
	Whole grains	26.1 (18.8-32.2)	34.4 (28.3-38.2)	1089 (783-1353)	2245 (1851-2502)
	Refined grains (total)	14.9 (10.5-20.4)	14.8 (10.5-20.6)	625 (439-855)	961 (679-1351)
	Refined rice	3.2 (2.2-4.6)	3.3 (2.2-5)	132 (91-194)	214 (147-326)
	Refined wheat	12.3 (8.6-17)	12 (8.4-17.1)	514 (360-707)	782 (549-1108)
	Processed meats	36.5 (23-52)	39.1 (24.3-56.4)	1519 (961-2168)	2538 (1574-3687)
	Unprocessed red meats	13.8 (9.9-19.2)	11.7 (7.7-17.2)	574 (417-810)	764 (495-1112)
	Yogurt	11.2 (10-12.1)	11.5 (10.2-12.6)	470 (418-512)	750 (661-828)
	Sugar-sweetened beverages	28 (19.8-38.2)	31.8 (22.5-43.6)	1171 (824-1602)	2073 (1458-2859)
	Potatoes	1.5 (0.8-2.8)	2.8 (1.5-5.3)	63 (34-120)	183 (97-348)
	Fruit	2.6 (2.2-2.9)	3 (2.5-3.5)	109 (91-124)	197 (164-227)
	Nuts and seeds	1.2 (1.2-1.3)	1.3 (1.2-1.4)	52 (48-55)	86 (77-94)
	Non-starchy vegetables	0.6 (0.3-0.9)	0.6 (0.2-1)	26 (12-40)	39 (16-66)
	Fruit juice	0.1 (0.1-0.2)	0.1 (0.1-0.2)	4 (3-7)	7 (4-12)

Table S5. Proportional and absolute burden per 1M population of T2D incidence attributable to suboptimal diet* and each individual dietary factor† at the national level in 1990 and 2018

Country	Dietary factor	Proportional burden, %		Rate, per 1M population‡	
		1990	2018	1990	2018
Argentina	Suboptimal diet	74.4 (71.5-77.6)	80.4 (77.5-83.3)	2088 (1978-2211)	3280 (3116-3462)
	Whole grains	38.2 (36.7-39.5)	35.3 (33.3-37.1)	1073 (1021-1124)	1442 (1350-1530)
	Refined grains (total)	8.8 (7.5-11.1)	16.8 (14.2-20.2)	248 (208-312)	684 (576-825)
	Refined rice	0.8 (0.7-1)	2.3 (1.9-3.1)	22 (19-27)	92 (77-126)
	Refined wheat	8.1 (6.8-10.2)	14.8 (12.5-18)	228 (191-286)	604 (509-731)
	Processed meats	17.8 (13-25.1)	30.8 (24-39)	498 (367-706)	1255 (977-1598)
	Unprocessed red meats	30 (25.3-36.3)	33.3 (27.5-40)	841 (705-1025)	1359 (1123-1636)
	Yogurt	9.4 (8.5-10.4)	9.7 (8.6-10.6)	265 (236-293)	394 (349-438)
	Sugar-sweetened beverages	12.3 (9.7-16.4)	19.6 (15.8-24.3)	345 (269-463)	801 (635-1001)
	Potatoes	5.7 (4.7-7.8)	4.1 (3.4-6.1)	161 (131-218)	168 (137-247)
	Fruit	2.6 (2.4-2.8)	3 (2.8-3.3)	73 (67-80)	124 (114-136)
	Nuts and seeds	1.3 (1.2-1.4)	1.4 (1.2-1.5)	36 (33-39)	55 (50-61)
	Non-starchy vegetables	1.1 (0.9-1.2)	1.3 (1.2-1.5)	30 (26-34)	54 (47-60)
	Fruit juice	0.1 (0-0.1)	0.1 (0-0.1)	2 (1-2)	3 (2-4)
Armenia	Suboptimal diet	75.3 (68.2-83.7)	92.8 (88.2-95.6)	1676 (1496-1890)	4200 (3933-4429)
	Whole grains	39.4 (36-41.7)	32.2 (25.6-37)	875 (790-948)	1458 (1158-1683)
	Refined grains (total)	29.8 (21.8-38.7)	24.2 (17.4-32.3)	663 (487-873)	1097 (790-1484)
	Refined rice	2.9 (2-4.4)	1 (0.7-1.6)	64 (45-95)	46 (32-71)
	Refined wheat	27.7 (20.2-36.2)	23.4 (16.8-31.3)	616 (451-816)	1061 (763-1439)
	Processed meats	34.1 (18.3-54.6)	80.1 (66.8-87.5)	762 (403-1242)	3624 (3015-4002)
	Unprocessed red meats	10.1 (6.8-15.7)	27.2 (20.5-35.1)	224 (151-348)	1227 (931-1596)
	Yogurt	8 (5.3-10.2)	6.3 (3.9-8.6)	178 (119-226)	286 (176-393)
	Sugar-sweetened beverages	2.1 (1.3-4.5)	6 (3.8-10.1)	46 (28-98)	272 (170-460)
	Potatoes	9.7 (5.6-15.7)	4.3 (2.7-8.3)	215 (127-349)	197 (121-371)
	Fruit	3.5 (3.3-3.8)	3.1 (2.8-3.4)	79 (72-86)	141 (124-157)
	Nuts and seeds	1.2 (1.1-1.4)	0.9 (0.7-1.1)	27 (24-31)	41 (31-51)
	Non-starchy vegetables	1.3 (1.1-1.5)	1.5 (1.3-1.7)	29 (25-34)	67 (56-79)
	Fruit juice	0.1 (0.1-0.2)	0.1 (0.1-0.2)	3 (2-5)	6 (4-9)
Australia	Suboptimal diet	65.8 (61.4-70.6)	63 (58-68.5)	1374 (1272-1483)	2311 (2113-2536)
	Whole grains	11.8 (8.7-15.1)	13 (9.8-16.3)	247 (182-315)	476 (359-597)
	Refined grains (total)	6.7 (4.6-9.8)	9.8 (6.8-13.8)	140 (95-204)	358 (250-508)
	Refined rice	1.3 (0.8-1.9)	2.6 (1.8-3.8)	26 (18-40)	94 (65-140)
	Refined wheat	5.5 (3.8-8.2)	7.4 (5.2-10.7)	115 (78-169)	273 (190-395)
	Processed meats	16.1 (11.7-23.7)	21.3 (15.4-29.8)	337 (244-493)	784 (569-1094)
	Unprocessed red meats	33 (28.2-38.9)	23.3 (19.3-29.7)	691 (588-814)	856 (710-1087)
	Yogurt	7.1 (5.1-8.8)	7.2 (5.1-9)	147 (107-185)	264 (185-332)

Table S5. Proportional and absolute burden per 1M population of T2D incidence attributable to suboptimal diet* and each individual dietary factor† at the national level in 1990 and 2018

Country	Dietary factor	Proportional burden, %		Rate, per 1M population‡	
		1990	2018	1990	2018
	Sugar-sweetened beverages	12.8 (10-17.4)	9.4 (7.3-13.5)	268 (208-365)	343 (265-496)
	Potatoes	9.1 (6.2-14.2)	7.6 (5-12)	189 (127-295)	277 (181-444)
	Fruit	1.7 (1.4-2)	2.2 (1.8-2.5)	36 (30-42)	80 (67-91)
	Nuts and seeds	1.1 (1-1.1)	1 (0.9-1.1)	22 (20-24)	36 (31-41)
	Non-starchy vegetables	1 (0.8-1.1)	1.2 (1-1.4)	21 (18-24)	43 (36-50)
	Fruit juice	0.2 (0.1-0.3)	0.2 (0.1-0.4)	4 (2-6)	9 (5-14)
Austria	Suboptimal diet	86.6 (79.7-92.9)	86.2 (78.6-93.4)	1774 (1629-1926)	3283 (2996-3562)
	Whole grains	26 (23.8-28.2)	26.2 (23.8-28.4)	534 (486-583)	995 (905-1084)
	Refined grains (total)	4.2 (2.7-6.8)	2.6 (1.7-4.3)	86 (55-139)	98 (64-165)
	Refined rice	0.6 (0.4-1.1)	0.2 (0.2-0.4)	13 (9-22)	9 (6-14)
	Refined wheat	3.6 (2.3-5.8)	2.3 (1.5-3.9)	73 (47-120)	89 (58-150)
	Processed meats	54.7 (34-76.9)	58.7 (37.1-80.2)	1123 (698-1585)	2233 (1416-3032)
	Unprocessed red meats	46.4 (39.8-53.1)	40.5 (34.5-47.2)	951 (815-1097)	1541 (1307-1796)
	Yogurt	7.6 (5.7-9.2)	7.3 (5.3-9.1)	155 (116-189)	278 (202-347)
	Sugar-sweetened beverages	8.7 (7-11.5)	9.1 (7.4-11.8)	178 (146-232)	343 (281-452)
	Potatoes	6.5 (5.5-8.5)	5.9 (5.1-8)	135 (114-173)	224 (191-306)
	Fruit	2.3 (2.1-2.4)	2.6 (2.4-2.8)	46 (42-50)	98 (89-107)
	Nuts and seeds	1 (0.9-1.1)	1.1 (1-1.2)	21 (19-22)	41 (38-45)
	Non-starchy vegetables	1.3 (1.2-1.4)	1.2 (1.1-1.3)	27 (25-29)	46 (42-51)
	Fruit juice	0.1 (0.1-0.1)	0.1 (0.1-0.2)	2 (2-3)	5 (3-6)
Azerbaijan	Suboptimal diet	89.3 (83.1-93.7)	95 (91.6-96.9)	1654 (1514-1770)	4047 (3866-4229)
	Whole grains	20.1 (13.1-27.3)	25.6 (18.1-32.7)	370 (240-508)	1089 (774-1397)
	Refined grains (total)	32.2 (25.9-38.1)	32.8 (26.6-39.2)	596 (477-708)	1405 (1130-1678)
	Refined rice	0.9 (0.7-1.4)	1.9 (1.4-2.5)	17 (13-26)	80 (61-108)
	Refined wheat	31.6 (25.4-37.5)	31.6 (25.5-37.8)	584 (467-695)	1350 (1084-1618)
	Processed meats	72.8 (56.6-83.8)	79.8 (66.7-87)	1350 (1034-1570)	3401 (2846-3745)
	Unprocessed red meats	18.3 (14.3-23.6)	40.4 (34.2-47.4)	340 (263-439)	1723 (1457-2028)
	Yogurt	10.3 (8.8-11.5)	10.3 (8.8-11.5)	191 (163-214)	438 (374-493)
	Sugar-sweetened beverages	3.8 (2.4-6.6)	5 (3.2-8.1)	71 (44-123)	214 (139-347)
	Potatoes	1.9 (1.1-3.3)	9.9 (6.1-16.4)	34 (21-62)	426 (257-693)
	Fruit	2.6 (2.3-2.9)	2.9 (2.6-3.2)	48 (42-54)	125 (110-140)
	Nuts and seeds	1.1 (1-1.3)	0.9 (0.7-1)	21 (18-23)	36 (29-44)
	Non-starchy vegetables	1.7 (1.5-1.8)	1.7 (1.5-1.9)	31 (28-34)	72 (64-80)
	Fruit juice	0.2 (0.1-0.3)	0.2 (0.1-0.3)	3 (2-5)	7 (5-12)
Bahrain	Suboptimal diet	72.8 (64.5-80.5)	76.4 (68.1-84.9)	3189 (2801-3585)	8665 (7635-9668)

Table S5. Proportional and absolute burden per 1M population of T2D incidence attributable to suboptimal diet* and each individual dietary factor† at the national level in 1990 and 2018

Country	Dietary factor	Proportional burden, %		Rate, per 1M population‡	
		1990	2018	1990	2018
	Whole grains	34.5 (26.2-39.7)	33.8 (25.3-39.3)	1506 (1146-1742)	3843 (2888-4486)
	Refined grains (total)	27 (16.2-40)	30.5 (18.3-43.8)	1181 (713-1778)	3453 (2086-4997)
	Refined rice	27 (16.2-40)	30.5 (18.3-43.8)	1181 (713-1778)	3453 (2086-4997)
	Refined wheat	0 (0-0)	0 (0-0)	0 (0-0)	0 (0-0)
	Processed meats	24 (11.2-43)	27.9 (12.5-48.3)	1052 (494-1904)	3140 (1410-5486)
	Unprocessed red meats	14.5 (9-22.2)	13.9 (8.3-22.1)	636 (393-979)	1584 (952-2514)
	Yogurt	7.8 (5.1-10)	7.7 (4.9-10.2)	342 (220-440)	873 (556-1161)
	Sugar-sweetened beverages	16 (11-23.2)	18.1 (12.2-27)	696 (482-1016)	2047 (1409-3107)
	Potatoes	2.9 (1.7-5.5)	5 (2.6-9.8)	128 (73-239)	574 (299-1132)
	Fruit	2.6 (2.3-2.8)	2.7 (2.3-3.1)	112 (98-126)	308 (263-353)
	Nuts and seeds	1.1 (1-1.3)	0.9 (0.6-1.1)	49 (42-57)	98 (73-126)
	Non-starchy vegetables	0.8 (0.6-1)	0.9 (0.6-1.2)	34 (24-45)	101 (68-135)
	Fruit juice	0.1 (0.1-0.2)	0.1 (0.1-0.3)	6 (3-11)	16 (8-31)
Bangladesh	Suboptimal diet	65.9 (62.9-68.8)	66.6 (63.6-69.6)	1373 (1297-1445)	2008 (1913-2107)
	Whole grains	27.8 (23.6-30.9)	25.5 (20.5-29.4)	578 (490-647)	768 (619-891)
	Refined grains (total)	46.2 (42.2-50.5)	45.8 (41.8-50.1)	965 (870-1056)	1382 (1259-1510)
	Refined rice	44.1 (40.1-48.3)	44.1 (40.2-48.3)	921 (829-1010)	1331 (1212-1458)
	Refined wheat	3.8 (3.4-5)	3 (2.7-3.9)	80 (70-106)	91 (81-119)
	Processed meats	0.5 (0.3-1.3)	0.4 (0.3-0.8)	10 (7-27)	11 (8-24)
	Unprocessed red meats	2.2 (1.4-4.6)	2.3 (1.5-4.6)	47 (30-97)	69 (46-138)
	Yogurt	13.4 (12.7-14.2)	13.3 (12.6-13.9)	279 (264-298)	399 (378-424)
	Sugar-sweetened beverages	0.9 (0.6-1.8)	0.9 (0.6-2)	19 (12-39)	28 (17-59)
	Potatoes	5.4 (4.3-7.4)	8.9 (7.4-12.1)	113 (90-155)	270 (222-364)
	Fruit	3.3 (3.1-3.5)	3.6 (3.5-3.8)	69 (65-73)	109 (104-116)
	Nuts and seeds	1.4 (1.3-1.5)	1.5 (1.4-1.6)	29 (27-31)	44 (41-47)
	Non-starchy vegetables	0.5 (0.4-0.6)	0.6 (0.5-0.7)	10 (8-12)	18 (15-20)
	Fruit juice	0 (0-0)	0 (0-0)	0 (0-0)	0 (0-0)
Barbados	Suboptimal diet	89.1 (86.2-91.1)	77.4 (71.8-81.8)	4171 (3992-4343)	5591 (5195-5958)
	Whole grains	10.1 (6.4-13.9)	10.5 (6.5-14.8)	472 (297-652)	761 (469-1076)
	Refined grains (total)	26.6 (19.9-34)	19.9 (14.2-26.7)	1249 (933-1587)	1439 (1017-1968)
	Refined rice	11.1 (7.9-14.6)	6.5 (4.4-9.5)	519 (373-687)	472 (323-692)
	Refined wheat	18.2 (13.3-23.8)	14.6 (10.2-20)	856 (627-1111)	1056 (736-1468)
	Processed meats	17.2 (10.6-25.2)	15.5 (9.9-24)	806 (498-1181)	1128 (711-1723)
	Unprocessed red meats	66.5 (60.1-69.6)	23.9 (17.5-32.4)	3110 (2808-3299)	1732 (1266-2331)
	Yogurt	10.2 (8.9-11.2)	10.7 (9.3-11.8)	480 (415-527)	775 (677-856)

Table S5. Proportional and absolute burden per 1M population of T2D incidence attributable to suboptimal diet* and each individual dietary factor† at the national level in 1990 and 2018

Country	Dietary factor	Proportional burden, %		Rate, per 1M population‡	
		1990	2018	1990	2018
	Sugar-sweetened beverages	32.9 (26.6-40.2)	42.4 (34.4-50.8)	1537 (1236-1904)	3074 (2475-3686)
	Potatoes	2 (1.1-3.7)	3.5 (1.8-6.6)	93 (50-175)	252 (133-470)
	Fruit	2.1 (1.8-2.3)	2.5 (2.2-2.8)	97 (84-110)	181 (155-203)
	Nuts and seeds	1 (0.9-1.1)	1 (0.8-1.1)	47 (42-52)	71 (59-81)
	Non-starchy vegetables	0.9 (0.8-1.1)	1.2 (1-1.3)	44 (37-51)	84 (71-97)
	Fruit juice	0 (0-0)	0 (0-0)	1 (1-2)	2 (1-3)
Belarus	Suboptimal diet	77.8 (70-85.1)	81.3 (74.2-87.8)	1458 (1302-1604)	1760 (1603-1925)
	Whole grains	16.7 (10.2-24.4)	28.3 (21.1-34.2)	312 (194-459)	613 (453-748)
	Refined grains (total)	17.4 (12.3-24.1)	20.1 (14.2-27.3)	325 (229-446)	434 (307-599)
	Refined rice	1.5 (1-2.4)	2.1 (1.4-3.2)	28 (19-45)	46 (31-70)
	Refined wheat	16.2 (11.4-22.5)	18.5 (13.1-25.2)	303 (213-418)	399 (281-552)
	Processed meats	36.1 (22.1-51.9)	43.5 (28.1-58.5)	677 (413-958)	946 (625-1275)
	Unprocessed red meats	32.7 (19.2-50.2)	30.4 (16.9-46.8)	609 (358-938)	659 (367-1018)
	Yogurt	6.6 (4.2-8.4)	7.4 (5-9.3)	123 (79-158)	160 (108-203)
	Sugar-sweetened beverages	8.2 (5.5-12.5)	4.7 (3.1-7.8)	154 (104-234)	103 (67-169)
	Potatoes	13.4 (8.2-20.5)	14.7 (9.3-22.3)	250 (154-383)	319 (201-483)
	Fruit	2.7 (1.8-3.3)	1.2 (0.4-2.5)	50 (33-61)	25 (8-54)
	Nuts and seeds	1.4 (1.3-1.5)	1.1 (0.9-1.3)	25 (24-28)	24 (20-28)
	Non-starchy vegetables	1.4 (0.9-1.7)	1.6 (1-2)	26 (16-33)	34 (21-44)
	Fruit juice	0.2 (0.1-0.3)	0.2 (0.1-0.3)	3 (2-5)	4 (2-6)
Belgium	Suboptimal diet	74.9 (70.8-79.5)	74.2 (69.8-78.5)	1879 (1742-2021)	2814 (2627-3018)
	Whole grains	16.9 (13.5-20.3)	14 (9.3-18.7)	423 (343-506)	531 (354-714)
	Refined grains (total)	5 (3.1-8)	6.2 (3.9-10.3)	125 (77-199)	235 (146-392)
	Refined rice	5 (3.1-8)	0.9 (0.6-1.6)	125 (77-199)	35 (22-61)
	Refined wheat	0 (0-0)	5.3 (3.3-8.9)	0 (0-0)	202 (125-340)
	Processed meats	36.7 (28.6-46.9)	36.1 (28.9-45.5)	918 (715-1176)	1379 (1098-1727)
	Unprocessed red meats	27.3 (22.3-33.5)	23.7 (19.2-30)	681 (558-844)	901 (726-1138)
	Yogurt	10 (8-11.3)	10.1 (8.1-11.4)	250 (201-288)	383 (304-437)
	Sugar-sweetened beverages	13.8 (11.6-18.3)	16.7 (14-21.2)	346 (289-459)	636 (529-800)
	Potatoes	10.7 (8.8-14.6)	11.5 (9.5-15.2)	269 (219-366)	440 (360-581)
	Fruit	2.2 (2-2.4)	2.6 (2.4-2.8)	56 (51-62)	99 (90-109)
	Nuts and seeds	1.1 (1-1.2)	1.2 (1.1-1.3)	27 (25-30)	45 (40-50)
	Non-starchy vegetables	1.3 (1.2-1.4)	1.3 (1.1-1.4)	32 (29-35)	48 (42-53)
	Fruit juice	0.2 (0.2-0.3)	0.3 (0.2-0.4)	5 (4-7)	12 (9-15)
Belize	Suboptimal diet	81.1 (75.5-86.9)	90.4 (86.2-94.1)	2099 (1935-2267)	4284 (4031-4503)

Table S5. Proportional and absolute burden per 1M population of T2D incidence attributable to suboptimal diet* and each individual dietary factor† at the national level in 1990 and 2018

Country	Dietary factor	Proportional burden, %		Rate, per 1M population‡	
		1990	2018	1990	2018
	Whole grains	31.9 (24.6-36.5)	29.6 (21.1-35.8)	827 (632-956)	1398 (994-1700)
	Refined grains (total)	17.5 (12.3-23.8)	20.4 (14.7-27.7)	451 (321-614)	967 (692-1310)
	Refined rice	7.7 (5.3-11.1)	11.4 (8.1-16.1)	200 (137-287)	539 (380-760)
	Refined wheat	10.8 (7.5-15.3)	10.6 (7.5-15.1)	280 (195-395)	502 (353-708)
	Processed meats	37.5 (22.4-55.9)	52.2 (36.1-69.2)	971 (582-1451)	2471 (1709-3257)
	Unprocessed red meats	28.6 (21.1-36.3)	39.2 (30.1-48)	742 (546-948)	1855 (1411-2292)
	Yogurt	11.4 (9.9-12.5)	12.1 (10.6-13.2)	295 (258-327)	572 (497-630)
	Sugar-sweetened beverages	22.3 (15.7-30.1)	37.3 (27.2-49.2)	577 (403-785)	1767 (1291-2298)
	Potatoes	2.9 (1.5-5.3)	3.6 (1.9-6.8)	75 (41-135)	171 (89-320)
	Fruit	2.4 (1.8-2.9)	3.2 (2.7-3.7)	63 (48-75)	153 (126-175)
	Nuts and seeds	1.4 (1.3-1.4)	1.4 (1.2-1.5)	35 (32-38)	65 (58-72)
	Non-starchy vegetables	1.4 (1.1-1.6)	1.1 (0.7-1.5)	36 (29-42)	54 (31-73)
	Fruit juice	0.1 (0.1-0.2)	0.1 (0.1-0.2)	3 (2-5)	7 (4-12)
Benin	Suboptimal diet	64.9 (56-77.8)	70.2 (60.9-82.8)	1058 (910-1273)	1851 (1595-2185)
	Whole grains	21.8 (14.2-28.5)	28.8 (21.1-34.4)	354 (232-462)	757 (558-913)
	Refined grains (total)	12 (9.7-14.9)	12.5 (10.3-15.6)	195 (156-244)	329 (272-411)
	Refined rice	9.4 (7.6-11.9)	11.2 (9.2-14)	152 (122-195)	294 (241-369)
	Refined wheat	2.9 (2.3-3.8)	1.5 (1.2-1.9)	47 (37-62)	39 (32-52)
	Processed meats	24.1 (9.2-50.9)	27.9 (10.4-55.4)	389 (149-821)	734 (273-1478)
	Unprocessed red meats	15.2 (11.4-20.3)	8.3 (5.8-12)	248 (185-329)	219 (154-321)
	Yogurt	11.3 (10.2-12.3)	12 (11-13.1)	183 (165-201)	317 (287-346)
	Sugar-sweetened beverages	14.7 (9.4-22.3)	23.6 (15.5-34.4)	238 (152-362)	624 (407-921)
	Potatoes	0.7 (0.4-1.5)	1 (0.5-2.1)	12 (6-25)	25 (13-54)
	Fruit	2.8 (2.7-3)	3.1 (2.9-3.3)	46 (43-48)	82 (76-88)
	Nuts and seeds	1 (0.8-1.1)	1 (0.8-1.1)	16 (14-18)	25 (20-30)
	Non-starchy vegetables	1.2 (1.1-1.3)	1.4 (1.3-1.5)	20 (18-21)	37 (34-41)
	Fruit juice	0.1 (0-0.1)	0.1 (0.1-0.1)	1 (1-2)	2 (1-3)
Bhutan	Suboptimal diet	70.6 (60.3-78.9)	73.3 (63.3-81.3)	1542 (1317-1734)	2456 (2112-2739)
	Whole grains	27.9 (14.1-37)	26.2 (13.4-36.1)	608 (310-809)	880 (447-1210)
	Refined grains (total)	48.3 (31.9-62)	48.5 (31.6-61.5)	1053 (706-1356)	1622 (1064-2077)
	Refined rice	48.3 (31.9-62)	48.5 (31.6-61.5)	1053 (706-1356)	1622 (1064-2077)
	Refined wheat	0 (0-0)	0 (0-0)	0 (0-0)	0 (0-0)
	Processed meats	2.8 (0.6-13.6)	2.4 (0.5-11.4)	62 (12-292)	81 (15-385)
	Unprocessed red meats	10.2 (5.7-17.3)	7.4 (3.8-12.6)	222 (125-382)	247 (129-423)
	Yogurt	12.9 (11.7-14)	12.9 (11.8-13.8)	282 (255-308)	432 (393-468)

Table S5. Proportional and absolute burden per 1M population of T2D incidence attributable to suboptimal diet* and each individual dietary factor† at the national level in 1990 and 2018

Country	Dietary factor	Proportional burden, %		Rate, per 1M population‡	
		1990	2018	1990	2018
	Sugar-sweetened beverages	4.7 (2.4-9)	10.7 (5.6-19)	101 (51-196)	357 (189-634)
	Potatoes	1.8 (0.9-4.1)	8.4 (4.1-17.3)	39 (19-90)	280 (138-578)
	Fruit	3 (2.6-3.3)	3.1 (2.7-3.5)	65 (56-73)	105 (89-119)
	Nuts and seeds	1.3 (1.2-1.5)	1.2 (0.9-1.4)	29 (25-32)	41 (31-48)
	Non-starchy vegetables	0.2 (0.1-0.6)	0.3 (0.1-0.7)	5 (2-14)	9 (3-24)
	Fruit juice	0 (0-0)	0 (0-0)	0 (0-1)	0 (0-1)
Bolivia	Suboptimal diet	78.1 (74.4-81.9)	80.5 (76.7-84.6)	1658 (1568-1754)	2983 (2814-3174)
	Whole grains	28.8 (21.2-34)	27.1 (19.3-33.2)	609 (448-724)	1003 (714-1232)
	Refined grains (total)	18.8 (16.1-21.8)	18.8 (16.2-22)	399 (342-463)	694 (597-820)
	Refined rice	8.8 (7.4-10.4)	9.6 (8.2-11.5)	187 (157-223)	354 (301-429)
	Refined wheat	11 (9.3-12.9)	10.2 (8.8-12.2)	234 (197-275)	378 (323-456)
	Processed meats	11.1 (5.3-21.6)	19.3 (10-33.6)	236 (113-462)	712 (367-1242)
	Unprocessed red meats	29.6 (26.1-33.6)	36.9 (32.9-41.7)	630 (554-712)	1366 (1212-1550)
	Yogurt	11.6 (10.4-12.4)	11.7 (10.4-12.5)	245 (219-264)	433 (385-467)
	Sugar-sweetened beverages	36.1 (28.6-44.7)	30.5 (23.2-38.4)	767 (609-954)	1129 (853-1424)
	Potatoes	4 (2.2-7.4)	4.1 (2.2-7.6)	86 (46-157)	154 (80-279)
	Fruit	2.7 (2.6-2.9)	3.1 (2.9-3.3)	58 (54-61)	116 (108-124)
	Nuts and seeds	1.1 (1-1.2)	1 (0.8-1.2)	24 (22-26)	37 (30-43)
	Non-starchy vegetables	1.2 (1.1-1.3)	1.4 (1.3-1.5)	25 (23-27)	51 (46-56)
	Fruit juice	0.1 (0.1-0.1)	0.1 (0.1-0.2)	2 (2-3)	5 (4-7)
Bosnia and Herzegovina	Suboptimal diet	53.5 (46.4-60.2)	83.7 (77-89.3)	1619 (1399-1830)	5778 (5277-6222)
	Whole grains	22.7 (15.3-29.3)	24.5 (17.6-30.8)	689 (469-885)	1691 (1207-2129)
	Refined grains (total)	3.4 (2.3-5.4)	15.3 (10.6-21.8)	104 (70-162)	1059 (725-1504)
	Refined rice	0 (0-0)	0.6 (0.4-1)	1 (1-1)	43 (29-66)
	Refined wheat	3.4 (2.3-5.3)	14.8 (10.2-21.1)	103 (69-161)	1022 (700-1456)
	Processed meats	14 (8.6-21.8)	36.9 (25.9-48.9)	425 (260-660)	2555 (1797-3390)
	Unprocessed red meats	12.9 (7.6-20.9)	51.3 (36.7-66.4)	391 (231-639)	3534 (2496-4569)
	Yogurt	6.9 (4.6-8.7)	6.9 (4.7-8.9)	207 (140-268)	477 (322-614)
	Sugar-sweetened beverages	1.5 (0.9-2.9)	5.9 (4-9.1)	44 (29-88)	404 (278-634)
	Potatoes	6.1 (3.7-10.5)	14.2 (8.9-21.8)	185 (110-320)	976 (607-1505)
	Fruit	3.3 (3.1-3.5)	0.3 (0.2-0.4)	99 (93-106)	18 (12-25)
	Nuts and seeds	1.3 (1.3-1.4)	1.1 (1-1.3)	41 (38-44)	79 (68-87)
	Non-starchy vegetables	1 (0.9-1.2)	0.1 (0-0.1)	31 (26-35)	4 (3-6)
	Fruit juice	0.2 (0.1-0.3)	0.1 (0.1-0.2)	5 (3-8)	7 (5-12)
Botswana	Suboptimal diet	63.7 (55.2-73.7)	70.5 (63.2-78.1)	1670 (1448-1938)	3219 (2860-3585)

Table S5. Proportional and absolute burden per 1M population of T2D incidence attributable to suboptimal diet* and each individual dietary factor† at the national level in 1990 and 2018

Country	Dietary factor	Proportional burden, %		Rate, per 1M population‡	
		1990	2018	1990	2018
	Whole grains	17.5 (10.4-25)	19.5 (11.8-27.5)	462 (273-653)	887 (540-1254)
	Refined grains (total)	8 (6-10.9)	11 (8.1-14.6)	210 (155-285)	500 (369-665)
	Refined rice	2.1 (1.6-3)	2.8 (2-4.1)	56 (41-79)	126 (91-186)
	Refined wheat	6.1 (4.5-8.3)	8.5 (6.2-11.5)	158 (116-216)	388 (284-531)
	Processed meats	21.2 (8.7-42.7)	12.3 (4.2-27.8)	554 (226-1127)	559 (194-1266)
	Unprocessed red meats	16.6 (12.5-21.2)	12.9 (9.2-17.7)	435 (328-552)	587 (420-807)
	Yogurt	10.1 (8.5-11.4)	10.5 (8.8-11.8)	264 (223-299)	480 (405-542)
	Sugar-sweetened beverages	19.3 (13.3-26.2)	39.8 (29.4-50.8)	503 (349-690)	1817 (1326-2310)
	Potatoes	1.3 (0.7-2.5)	2.8 (1.6-5.4)	33 (19-64)	125 (71-248)
	Fruit	3.1 (2.9-3.3)	3.6 (3.3-3.8)	81 (76-87)	163 (149-175)
	Nuts and seeds	0.9 (0.8-1.1)	0.7 (0.5-0.9)	25 (21-29)	31 (23-39)
	Non-starchy vegetables	1.1 (1-1.3)	1.4 (1.2-1.6)	30 (25-34)	63 (53-72)
	Fruit juice	0.1 (0.1-0.2)	0.1 (0.1-0.2)	3 (2-5)	6 (3-11)
Brazil	Suboptimal diet	73.7 (71-76.4)	83.3 (80.1-86.5)	2372 (2258-2501)	3600 (3421-3787)
	Whole grains	39.1 (37.7-40.4)	37.9 (36.3-39.3)	1258 (1199-1321)	1641 (1551-1731)
	Refined grains (total)	17.7 (14.9-20.8)	17.3 (14.7-20.5)	568 (478-675)	746 (630-888)
	Refined rice	11.9 (9.9-14.3)	9.7 (8.2-11.9)	380 (317-462)	418 (351-511)
	Refined wheat	6.6 (5.5-8.2)	8.4 (7.1-10.3)	211 (176-265)	362 (305-449)
	Processed meats	19.8 (14.8-26.6)	46.5 (37.9-55.5)	640 (472-859)	2016 (1634-2408)
	Unprocessed red meats	14.2 (11.3-18.3)	27.7 (23.9-32.9)	455 (363-593)	1196 (1026-1432)
	Yogurt	12.4 (11.5-13.2)	12.2 (11.3-13)	398 (369-428)	526 (487-566)
	Sugar-sweetened beverages	25.3 (21.6-30)	15.5 (13-19.7)	815 (692-967)	671 (560-853)
	Potatoes	1 (0.8-1.2)	1.7 (1.4-2.4)	33 (27-40)	75 (61-108)
	Fruit	2.8 (2.6-3)	3.1 (2.9-3.3)	90 (84-97)	135 (125-145)
	Nuts and seeds	1.3 (1.2-1.4)	1.4 (1.3-1.6)	43 (40-46)	63 (57-68)
	Non-starchy vegetables	1.4 (1.3-1.5)	1.5 (1.4-1.7)	44 (41-47)	67 (61-73)
	Fruit juice	0.3 (0.2-0.3)	0.3 (0.3-0.4)	9 (8-11)	15 (12-18)
Brunei	Suboptimal diet	80 (73.3-86.1)	86.6 (80-91.6)	4905 (4456-5312)	8504 (7806-9101)
	Whole grains	30.6 (23-36.2)	43.7 (42.3-45.1)	1871 (1398-2218)	4297 (4106-4488)
	Refined grains (total)	26.6 (17.7-36.8)	36.3 (23-50.2)	1629 (1091-2259)	3567 (2253-4990)
	Refined rice	21.3 (13.9-30)	36.3 (23-50.2)	1298 (854-1844)	3567 (2253-4990)
	Refined wheat	7.5 (4.7-11.3)	0 (0-0)	459 (288-691)	0 (0-0)
	Processed meats	31.5 (19.4-49.3)	38 (22-57.8)	1932 (1180-3033)	3731 (2142-5705)
	Unprocessed red meats	36.6 (28.1-45.7)	34.1 (25.1-42.8)	2242 (1723-2770)	3352 (2474-4238)
	Yogurt	13.1 (12-13.9)	12.8 (11.6-13.8)	798 (731-862)	1259 (1133-1366)

Table S5. Proportional and absolute burden per 1M population of T2D incidence attributable to suboptimal diet* and each individual dietary factor† at the national level in 1990 and 2018

Country	Dietary factor	Proportional burden, %		Rate, per 1M population‡	
		1990	2018	1990	2018
	Sugar-sweetened beverages	7.4 (4.8-11.1)	14.6 (9.9-20.6)	450 (295-678)	1440 (973-2017)
	Potatoes	0.3 (0.2-0.7)	1.1 (0.6-2.2)	21 (11-42)	108 (55-216)
	Fruit	3 (2.6-3.4)	3.4 (2.9-3.8)	186 (159-208)	334 (285-372)
	Nuts and seeds	1.2 (1.1-1.3)	1.3 (1.1-1.4)	73 (64-82)	127 (110-143)
	Non-starchy vegetables	1.1 (0.8-1.4)	1.1 (0.7-1.4)	67 (47-84)	105 (68-138)
	Fruit juice	0 (0-0.1)	0 (0-0.1)	2 (1-4)	4 (3-7)
Bulgaria	Suboptimal diet	78.7 (74.8-82.9)	75.1 (70.1-79.5)	2530 (2378-2696)	3385 (3124-3624)
	Whole grains	29.3 (25.7-32.2)	28.5 (25.1-31.7)	943 (826-1046)	1283 (1119-1430)
	Refined grains (total)	17.8 (11.9-26.3)	6.9 (4.6-10.7)	572 (384-855)	313 (207-479)
	Refined rice	1 (0.6-1.5)	0.2 (0.2-0.4)	31 (20-49)	10 (7-17)
	Refined wheat	17 (11.4-25.2)	6.7 (4.4-10.4)	546 (366-818)	302 (200-464)
	Processed meats	37.8 (29.8-47.3)	42.8 (34.1-52.5)	1215 (958-1510)	1930 (1530-2371)
	Unprocessed red meats	41.9 (36.4-47.8)	27.5 (22.5-32.7)	1347 (1165-1545)	1238 (1013-1482)
	Yogurt	0.2 (0.1-0.7)	0.2 (0.1-0.8)	7 (2-22)	11 (3-35)
	Sugar-sweetened beverages	4.2 (3-6.5)	4.2 (3.1-7)	135 (98-209)	191 (142-316)
	Potatoes	5.8 (4.1-8.8)	7.6 (5.3-11.3)	187 (130-284)	345 (239-511)
	Fruit	2.5 (2.3-2.8)	2.9 (2.6-3.1)	81 (72-89)	129 (114-143)
	Nuts and seeds	1 (0.9-1.2)	1 (0.8-1.1)	34 (30-37)	44 (38-51)
	Non-starchy vegetables	0 (0-0.1)	0.1 (0-0.1)	1 (1-2)	4 (2-6)
	Fruit juice	0.2 (0.1-0.3)	0.2 (0.1-0.3)	6 (4-9)	9 (6-14)
Burkina Faso	Suboptimal diet	45.4 (39.7-52.3)	52.9 (43.8-66.2)	1002 (872-1158)	1526 (1251-1916)
	Whole grains	22.2 (14.5-29.1)	21.4 (13.4-28.6)	488 (318-645)	615 (389-828)
	Refined grains (total)	10.7 (8.5-13.6)	11 (8.9-13.4)	237 (188-299)	315 (256-388)
	Refined rice	9.2 (7.3-11.7)	7.8 (6.3-9.7)	205 (162-260)	225 (182-279)
	Refined wheat	1.6 (1.3-2.2)	3.4 (2.8-4.4)	36 (28-47)	98 (79-127)
	Processed meats	3.1 (0.8-11.1)	9.4 (2.2-29.5)	68 (17-244)	270 (63-837)
	Unprocessed red meats	1.8 (1.1-4.1)	0.8 (0.4-2.7)	40 (24-94)	22 (12-79)
	Yogurt	12 (11.1-12.9)	12.7 (11.9-13.6)	265 (242-286)	366 (341-393)
	Sugar-sweetened beverages	2.3 (0.7-6.9)	13.7 (4.9-32.9)	51 (16-153)	396 (142-953)
	Potatoes	7.9 (4.1-14.3)	1.7 (0.9-3.7)	174 (92-315)	49 (26-106)
	Fruit	3.1 (3-3.3)	3.8 (3.6-4)	69 (65-73)	108 (102-114)
	Nuts and seeds	0.7 (0.4-1)	0.7 (0.4-1.1)	15 (10-22)	20 (13-31)
	Non-starchy vegetables	1.4 (1.3-1.5)	2.1 (2-2.2)	31 (29-34)	60 (56-64)
	Fruit juice	0 (0-0.1)	0.1 (0-0.1)	1 (1-1)	2 (1-2)
Burundi	Suboptimal diet	53.4 (46.3-59.8)	62.1 (54.8-69.2)	1030 (894-1161)	1317 (1156-1479)

Table S5. Proportional and absolute burden per 1M population of T2D incidence attributable to suboptimal diet* and each individual dietary factor† at the national level in 1990 and 2018

Country	Dietary factor	Proportional burden, %		Rate, per 1M population‡	
		1990	2018	1990	2018
	Whole grains	28 (18.8-34.8)	29.2 (19.9-36.5)	540 (362-675)	620 (419-773)
	Refined grains (total)	8.3 (6.3-11.6)	9.2 (7.2-12.5)	159 (122-225)	196 (153-266)
	Refined rice	8.3 (6.3-11.6)	9.2 (7.2-12.5)	159 (122-225)	196 (153-266)
	Refined wheat	0 (0-0)	0 (0-0)	0 (0-0)	0 (0-0)
	Processed meats	3.9 (1.2-12.1)	7.3 (2.2-18.2)	74 (23-232)	155 (46-387)
	Unprocessed red meats	4.2 (2.6-8.3)	4.9 (3-9.6)	81 (51-161)	104 (64-201)
	Yogurt	12.9 (11.9-13.9)	13.5 (12.5-14.5)	250 (227-272)	287 (264-309)
	Sugar-sweetened beverages	14.9 (9.7-22)	23.5 (16-32.9)	286 (186-426)	498 (339-699)
	Potatoes	0.9 (0.5-1.8)	3.6 (2.1-7.3)	18 (10-35)	76 (44-156)
	Fruit	2.7 (2.4-2.9)	3.2 (2.9-3.5)	52 (46-57)	68 (61-75)
	Nuts and seeds	1.2 (1.1-1.3)	1.2 (1-1.3)	23 (20-26)	25 (20-29)
	Non-starchy vegetables	0.6 (0.4-0.8)	0.7 (0.5-1)	12 (8-15)	16 (11-21)
	Fruit juice	0 (0-0.1)	0.1 (0-0.1)	1 (1-1)	1 (1-2)
Cambodia	Suboptimal diet	60.3 (55.6-66.1)	61.8 (58.1-66.9)	1219 (1122-1340)	2531 (2345-2761)
	Whole grains	36.5 (31.8-39.1)	33.6 (27.5-37.5)	735 (642-794)	1374 (1117-1547)
	Refined grains (total)	27 (22-32.7)	21.9 (19-25.6)	544 (444-659)	896 (780-1046)
	Refined rice	27 (22-32.7)	21.7 (18.9-25.4)	544 (444-659)	889 (774-1039)
	Refined wheat	0 (0-0)	0.2 (0.2-0.2)	0 (0-0)	8 (7-10)
	Processed meats	7.9 (2.3-21)	7.2 (2.8-19.3)	160 (45-431)	297 (112-782)
	Unprocessed red meats	9 (6.7-13.1)	8.3 (6.2-12.2)	182 (136-263)	341 (256-501)
	Yogurt	13.2 (12.5-14.1)	13.3 (12.5-14.2)	267 (249-286)	545 (509-587)
	Sugar-sweetened beverages	0.3 (0.2-0.8)	12.3 (9.2-16.8)	6 (4-16)	507 (376-692)
	Potatoes	0.3 (0.2-0.8)	0.3 (0.2-0.8)	7 (3-15)	14 (7-32)
	Fruit	3.1 (3-3.3)	3.6 (3.4-3.8)	63 (59-67)	146 (137-157)
	Nuts and seeds	1.3 (1.2-1.4)	1.3 (1.2-1.4)	26 (25-29)	55 (50-60)
	Non-starchy vegetables	1.1 (1-1.2)	1.3 (1.2-1.4)	23 (20-25)	53 (47-59)
	Fruit juice	0 (0-0)	0 (0-0)	0 (0-1)	1 (1-1)
Cameroon	Suboptimal diet	53 (47.6-60.9)	61.9 (56.5-68.1)	809 (724-927)	1767 (1608-1940)
	Whole grains	21.8 (14.6-28.5)	19.5 (11.8-27.2)	334 (222-437)	555 (338-780)
	Refined grains (total)	11.5 (9.4-14.2)	20 (16.6-24.2)	174 (142-217)	570 (477-696)
	Refined rice	7.3 (5.9-9.1)	14 (11.6-17.2)	111 (90-140)	400 (332-494)
	Refined wheat	4.5 (3.6-5.7)	7 (5.7-8.7)	68 (55-87)	198 (163-249)
	Processed meats	8.8 (2.9-22.6)	3.6 (1-11.3)	135 (45-343)	104 (30-323)
	Unprocessed red meats	14.3 (11-18.5)	5.6 (4-8.3)	218 (167-283)	160 (112-237)
	Yogurt	11.8 (10.8-12.5)	12.4 (11.5-13.1)	180 (164-192)	353 (324-377)

Table S5. Proportional and absolute burden per 1M population of T2D incidence attributable to suboptimal diet* and each individual dietary factor† at the national level in 1990 and 2018

Country	Dietary factor	Proportional burden, %		Rate, per 1M population‡	
		1990	2018	1990	2018
	Sugar-sweetened beverages	6.8 (4.4-10.7)	25.1 (18-33.6)	104 (67-162)	716 (512-955)
	Potatoes	0.2 (0.1-0.4)	4.6 (2.6-8.4)	3 (2-5)	132 (73-244)
	Fruit	2.7 (2.5-2.8)	3 (2.9-3.3)	40 (38-43)	87 (81-93)
	Nuts and seeds	1.1 (1-1.2)	0.9 (0.8-1.1)	17 (16-19)	27 (22-32)
	Non-starchy vegetables	1 (0.9-1.1)	1.2 (1.1-1.4)	15 (14-17)	35 (31-39)
	Fruit juice	0 (0-0.1)	0.1 (0-0.1)	1 (0-1)	1 (1-2)
Canada	Suboptimal diet	65 (62.1-68.2)	66.5 (62.9-70.8)	1154 (1093-1225)	2131 (1992-2289)
	Whole grains	25.1 (23.2-27)	15.5 (13.2-17.7)	445 (407-483)	496 (421-573)
	Refined grains (total)	10.6 (9-12.7)	11.2 (9.6-13.6)	188 (158-227)	360 (307-439)
	Refined rice	1.2 (1-1.5)	2.4 (2.1-3.2)	21 (18-26)	78 (66-101)
	Refined wheat	9.5 (8-11.4)	9 (7.7-10.9)	168 (141-202)	289 (245-352)
	Processed meats	20.4 (15.8-26.9)	31 (25.1-38.9)	362 (281-480)	996 (797-1255)
	Unprocessed red meats	20.8 (18-25)	18.1 (15.4-22.7)	370 (319-446)	584 (494-729)
	Yogurt	9.2 (8.3-9.9)	9.5 (8.6-10.3)	163 (148-177)	305 (274-332)
	Sugar-sweetened beverages	10.1 (8.7-13)	11.7 (10-15.7)	180 (154-235)	377 (320-505)
	Potatoes	4.3 (3.8-5.5)	3.5 (3.1-5.2)	77 (68-98)	113 (100-167)
	Fruit	2.3 (2.2-2.4)	2.2 (1.9-2.4)	41 (38-44)	69 (62-76)
	Nuts and seeds	0.9 (0.8-1)	0.7 (0.6-0.8)	16 (15-18)	23 (20-27)
	Non-starchy vegetables	0.9 (0.8-1)	1.1 (1-1.2)	16 (15-18)	35 (31-39)
	Fruit juice	0.3 (0.3-0.4)	0.4 (0.3-0.5)	6 (5-7)	13 (11-15)
Cape Verde	Suboptimal diet	42.2 (36.3-49.2)	54.7 (48.4-62.1)	972 (829-1134)	2094 (1841-2350)
	Whole grains	12.3 (6.7-20.1)	20.9 (13.1-28.8)	282 (154-466)	795 (496-1103)
	Refined grains (total)	8.2 (5.9-11.2)	14.8 (11.1-19.8)	190 (136-259)	569 (420-763)
	Refined rice	4.9 (3.5-6.9)	10.9 (8.1-14.8)	113 (80-158)	419 (307-568)
	Refined wheat	3.5 (2.5-5)	4.5 (3.3-6.7)	81 (58-115)	174 (125-255)
	Processed meats	6.5 (2-17)	8.2 (2.9-18.9)	151 (47-393)	313 (111-725)
	Unprocessed red meats	6.5 (4.3-9.8)	9.4 (6.7-14.8)	148 (100-226)	361 (254-558)
	Yogurt	10.3 (8.9-11.4)	11.1 (9.8-12.3)	238 (206-263)	424 (373-474)
	Sugar-sweetened beverages	7.6 (5-11.3)	12.2 (8-18.5)	174 (113-262)	464 (304-707)
	Potatoes	0.8 (0.4-1.4)	3.2 (1.8-6.1)	18 (10-33)	123 (69-233)
	Fruit	2.5 (2.2-2.8)	1.3 (0.7-2)	58 (50-64)	50 (28-75)
	Nuts and seeds	0.9 (0.8-1)	0.9 (0.7-1.1)	21 (17-24)	35 (28-43)
	Non-starchy vegetables	1.4 (1.3-1.6)	0.8 (0.4-1.1)	33 (30-36)	29 (17-42)
	Fruit juice	0.1 (0-0.1)	0.1 (0.1-0.1)	2 (1-3)	3 (2-6)
Central African Republic	Suboptimal diet	65.9 (60.6-72.6)	70 (64-76.6)	1643 (1502-1815)	3025 (2750-3324)

Table S5. Proportional and absolute burden per 1M population of T2D incidence attributable to suboptimal diet* and each individual dietary factor† at the national level in 1990 and 2018

Country	Dietary factor	Proportional burden, %		Rate, per 1M population‡	
		1990	2018	1990	2018
	Whole grains	30.1 (22.9-35.9)	26.2 (17.7-33.6)	751 (564-901)	1126 (763-1450)
	Refined grains (total)	10.1 (7.5-13.6)	9.9 (7.3-13.9)	251 (187-343)	428 (314-610)
	Refined rice	3.6 (2.6-5.3)	7.7 (5.6-11)	89 (66-132)	333 (244-480)
	Refined wheat	6.8 (5.1-9.5)	2.5 (1.8-3.7)	170 (126-238)	106 (77-161)
	Processed meats	7.3 (1.9-22.5)	7.7 (2.1-24.6)	181 (48-560)	336 (90-1055)
	Unprocessed red meats	30.3 (24.9-36.9)	39.4 (32.6-46.6)	756 (614-927)	1705 (1406-2008)
	Yogurt	12.4 (11.3-13.4)	13 (11.9-14)	310 (280-336)	561 (508-611)
	Sugar-sweetened beverages	7.8 (4.9-11.8)	9.2 (5.9-14)	194 (121-298)	399 (253-607)
	Potatoes	1.4 (0.7-2.8)	1.3 (0.7-2.9)	34 (17-70)	57 (29-127)
	Fruit	2.8 (2.6-3)	3 (2.8-3.3)	71 (65-76)	131 (119-143)
	Nuts and seeds	1 (0.9-1.2)	1.2 (1-1.4)	26 (21-30)	51 (42-59)
	Non-starchy vegetables	1.1 (1-1.3)	1.5 (1.3-1.7)	29 (25-33)	66 (57-74)
	Fruit juice	0.1 (0.1-0.2)	0.1 (0.1-0.2)	3 (2-5)	6 (4-11)
Chad	Suboptimal diet	59.2 (53.3-65.7)	60.5 (55-66.7)	1105 (992-1234)	1444 (1306-1596)
	Whole grains	28.1 (20.9-33.4)	21.6 (13.4-29.1)	524 (389-628)	517 (319-699)
	Refined grains (total)	11.2 (9-14.1)	11.5 (9.5-14.3)	209 (168-262)	274 (226-341)
	Refined rice	9.4 (7.6-11.9)	8.8 (7.3-11.3)	176 (140-220)	210 (174-270)
	Refined wheat	2 (1.6-2.7)	2.9 (2.4-3.7)	37 (29-50)	69 (56-90)
	Processed meats	7.1 (2.2-21.1)	5.5 (1.6-16.1)	133 (41-398)	132 (38-381)
	Unprocessed red meats	18 (14-23.1)	16.8 (13.1-21.9)	336 (264-433)	402 (314-521)
	Yogurt	11.2 (10.1-12.2)	12.2 (11.2-13.2)	209 (187-229)	293 (265-318)
	Sugar-sweetened beverages	12 (7.9-17.5)	18.8 (12.9-26)	225 (148-329)	448 (310-626)
	Potatoes	1.2 (0.7-2.4)	1.5 (0.8-2.8)	23 (13-45)	35 (19-68)
	Fruit	1 (0.8-1.3)	3.7 (3.5-3.9)	19 (15-24)	89 (84-95)
	Nuts and seeds	1.1 (1-1.2)	1.1 (0.9-1.2)	20 (18-22)	26 (21-30)
	Non-starchy vegetables	1 (0.9-1.1)	2 (1.9-2.2)	18 (16-21)	49 (45-52)
	Fruit juice	0 (0-0)	0 (0-0.1)	0 (0-1)	1 (1-1)
Chile	Suboptimal diet	71.7 (65.2-78.4)	81.9 (75.1-88.4)	1900 (1702-2102)	4036 (3675-4407)
	Whole grains	35.5 (29.8-39)	25 (16.4-32.3)	939 (773-1038)	1235 (810-1589)
	Refined grains (total)	22.3 (13.9-34.8)	22.6 (14-35)	589 (365-929)	1116 (687-1733)
	Refined rice	2.5 (1.5-4.3)	3.5 (2.1-6.1)	65 (39-113)	174 (104-304)
	Refined wheat	20.2 (12.5-32)	19.7 (12.2-30.9)	536 (330-855)	975 (596-1531)
	Processed meats	29.8 (19-44)	53.3 (38.4-69)	790 (499-1174)	2636 (1888-3426)
	Unprocessed red meats	5 (1.8-10.6)	6 (2.4-12.8)	132 (47-276)	297 (121-631)
	Yogurt	9.9 (8-11.3)	9.5 (7.4-11)	261 (210-303)	467 (364-546)

Table S5. Proportional and absolute burden per 1M population of T2D incidence attributable to suboptimal diet* and each individual dietary factor† at the national level in 1990 and 2018

Country	Dietary factor	Proportional burden, %		Rate, per 1M population‡	
		1990	2018	1990	2018
	Sugar-sweetened beverages	18.6 (14.1-25)	26.1 (19.7-33.6)	493 (373-661)	1288 (962-1656)
	Potatoes	1.1 (0.7-2)	1.6 (0.9-3.1)	30 (17-54)	78 (45-152)
	Fruit	2.5 (2.2-2.8)	2.9 (2.5-3.2)	67 (58-75)	141 (123-159)
	Nuts and seeds	1.3 (1.2-1.4)	0.9 (0.7-1.1)	35 (32-38)	45 (35-54)
	Non-starchy vegetables	1.1 (0.9-1.2)	1.2 (1-1.4)	28 (23-33)	60 (50-71)
	Fruit juice	0 (0-0.1)	0 (0-0.1)	1 (0-2)	1 (0-4)
China	Suboptimal diet	63.3 (57.7-69.4)	75.7 (71.6-80)	1613 (1461-1779)	2460 (2319-2620)
	Whole grains	38.6 (37.2-40.1)	39.5 (38.2-40.7)	983 (937-1035)	1283 (1227-1338)
	Refined grains (total)	31.8 (21.9-42.9)	30.5 (21.1-41.4)	811 (567-1098)	987 (685-1358)
	Refined rice	23.2 (15.7-32.4)	23 (15.5-32.2)	593 (402-826)	745 (506-1050)
	Refined wheat	12.3 (8-17.8)	10.6 (6.9-15.7)	314 (206-459)	344 (226-508)
	Processed meats	4 (2.4-6.8)	4.4 (3-6.9)	102 (61-175)	144 (96-226)
	Unprocessed red meats	11 (8.5-14.4)	42.5 (37.4-47.1)	281 (214-368)	1380 (1205-1536)
	Yogurt	13.3 (12.4-14.1)	13 (12.1-13.7)	338 (314-361)	420 (389-449)
	Sugar-sweetened beverages	1.2 (0.9-2.5)	0.9 (0.7-1.9)	31 (23-65)	29 (22-63)
	Potatoes	0.3 (0.2-0.7)	1.6 (0.9-3.4)	8 (4-17)	53 (28-111)
	Fruit	3.1 (2.9-3.2)	3.2 (3.1-3.4)	78 (73-82)	105 (99-111)
	Nuts and seeds	1.4 (1.3-1.5)	1 (0.9-1.1)	36 (34-38)	33 (30-36)
	Non-starchy vegetables	2 (1.9-2.1)	0.3 (0.2-0.4)	51 (48-53)	9 (7-12)
	Fruit juice	0 (0-0)	0 (0-0)	0 (0-0)	0 (0-0)
Colombia	Suboptimal diet	94.4 (92.1-96)	94.6 (92.4-96.4)	3379 (3242-3513)	4421 (4232-4619)
	Whole grains	29.7 (22.1-35.3)	28.5 (20.2-35)	1064 (789-1270)	1333 (926-1632)
	Refined grains (total)	19.4 (16.9-22.8)	19 (16.5-22.3)	696 (601-822)	892 (769-1046)
	Refined rice	14.8 (12.8-17.7)	13.3 (11.5-15.9)	532 (457-632)	624 (534-745)
	Refined wheat	5.3 (4.6-6.6)	6.6 (5.6-8.1)	192 (164-239)	308 (262-378)
	Processed meats	68.8 (59-76.8)	67.4 (57.7-76.6)	2461 (2109-2767)	3152 (2684-3591)
	Unprocessed red meats	40.7 (36.6-45.4)	37 (32.9-41.6)	1458 (1309-1640)	1729 (1538-1962)
	Yogurt	10.5 (9.5-11.4)	10.7 (9.7-11.6)	376 (339-408)	499 (452-547)
	Sugar-sweetened beverages	41.7 (34-50.2)	52 (42.6-61.3)	1494 (1212-1814)	2433 (2005-2872)
	Potatoes	7.5 (4-13)	3.9 (2-7.2)	268 (142-466)	181 (95-334)
	Fruit	2.6 (2.5-2.8)	3 (2.8-3.2)	94 (88-101)	140 (130-150)
	Nuts and seeds	1.3 (1.2-1.4)	0.6 (0.5-0.8)	46 (42-50)	29 (22-37)
	Non-starchy vegetables	1.3 (1.3-1.4)	1.6 (1.4-1.7)	48 (45-52)	73 (67-80)
	Fruit juice	0.3 (0.2-0.4)	0.3 (0.2-0.4)	11 (9-13)	14 (11-18)
Comoros	Suboptimal diet	55.8 (50.2-61.3)	63.3 (56.9-69.8)	1122 (999-1241)	1332 (1190-1475)

Table S5. Proportional and absolute burden per 1M population of T2D incidence attributable to suboptimal diet* and each individual dietary factor† at the national level in 1990 and 2018

Country	Dietary factor	Proportional burden, %		Rate, per 1M population‡	
		1990	2018	1990	2018
	Whole grains	25.9 (17-32.7)	27.2 (18.5-34)	521 (342-661)	573 (385-721)
	Refined grains (total)	12.8 (10.2-16.1)	12.5 (10-15.9)	256 (205-325)	262 (210-335)
	Refined rice	12.8 (10.2-16.1)	12.5 (10-15.9)	256 (205-325)	262 (210-335)
	Refined wheat	0 (0-0)	0 (0-0)	0 (0-0)	0 (0-0)
	Processed meats	4.1 (1.2-11.1)	7 (2.4-17.8)	83 (25-222)	148 (51-377)
	Unprocessed red meats	11.4 (8.4-15.2)	11.9 (8.7-16.1)	229 (170-308)	250 (183-336)
	Yogurt	9.6 (7.6-11)	10.2 (8.4-11.6)	192 (153-222)	216 (176-244)
	Sugar-sweetened beverages	17 (11.8-23.1)	24.3 (17.2-33.5)	343 (236-467)	510 (364-703)
	Potatoes	1 (0.6-2)	3.6 (2-6.7)	20 (12-40)	76 (42-141)
	Fruit	2.5 (2.3-2.6)	3 (2.7-3.2)	49 (45-53)	62 (57-68)
	Nuts and seeds	1.1 (1-1.2)	1.1 (0.9-1.2)	22 (20-24)	23 (19-26)
	Non-starchy vegetables	1.2 (1.1-1.3)	1.4 (1.3-1.6)	24 (22-26)	30 (27-33)
	Fruit juice	0.1 (0.1-0.2)	0.1 (0.1-0.2)	2 (1-3)	3 (2-4)
Congo	Suboptimal diet	56.1 (48.4-66.8)	74.4 (65.7-83.4)	1611 (1379-1902)	2999 (2646-3395)
	Whole grains	28.9 (20.5-35.1)	19.9 (11.8-28.6)	827 (588-1008)	796 (473-1159)
	Refined grains (total)	14.6 (11.8-18.2)	14.1 (11.5-17.3)	419 (341-522)	569 (462-698)
	Refined rice	3.6 (2.8-4.8)	5.4 (4.4-6.9)	103 (81-138)	219 (176-280)
	Refined wheat	11.5 (9.2-14.4)	9.2 (7.4-11.5)	328 (265-410)	369 (298-466)
	Processed meats	16.5 (6.3-35.6)	31.3 (13.9-54.5)	475 (182-1019)	1262 (559-2197)
	Unprocessed red meats	4 (2.5-6.6)	10.5 (7.5-14.6)	114 (71-191)	424 (303-601)
	Yogurt	9.7 (8.1-11.1)	10 (8.4-11.3)	279 (232-320)	405 (335-462)
	Sugar-sweetened beverages	9 (5.9-13.5)	32.9 (23.7-42.4)	259 (169-391)	1322 (965-1728)
	Potatoes	1.1 (0.6-2.2)	3.6 (1.9-7.2)	31 (17-62)	145 (79-290)
	Fruit	2.7 (2.5-2.9)	3.1 (2.9-3.3)	77 (72-83)	125 (116-135)
	Nuts and seeds	0.7 (0.6-0.9)	1.1 (0.9-1.3)	20 (16-25)	45 (37-52)
	Non-starchy vegetables	1 (0.8-1.1)	1.1 (1-1.3)	27 (24-31)	46 (41-52)
	Fruit juice	0.1 (0.1-0.2)	0.1 (0.1-0.2)	3 (2-5)	6 (4-9)
Costa Rica	Suboptimal diet	81.4 (75.2-87.2)	82.7 (76.5-88.7)	2535 (2326-2730)	3962 (3636-4300)
	Whole grains	24.1 (15.7-30.9)	26.7 (18.1-33.5)	751 (489-965)	1273 (887-1599)
	Refined grains (total)	18.3 (12.9-24.5)	18.4 (12.7-25)	569 (403-756)	883 (607-1197)
	Refined rice	13.3 (9.2-18)	13.5 (9.1-18.7)	413 (288-560)	648 (439-892)
	Refined wheat	6.1 (4.1-8.5)	6 (4-8.5)	189 (128-265)	287 (190-409)
	Processed meats	38.8 (24.5-55)	44.7 (29.5-62.6)	1209 (761-1708)	2144 (1398-2999)
	Unprocessed red meats	26.2 (20-33.8)	29.3 (22.1-37.6)	815 (621-1049)	1409 (1046-1833)
	Yogurt	11.5 (10.3-12.4)	11.6 (10.2-12.7)	357 (318-388)	557 (486-611)

Table S5. Proportional and absolute burden per 1M population of T2D incidence attributable to suboptimal diet* and each individual dietary factor† at the national level in 1990 and 2018

Country	Dietary factor	Proportional burden, %		Rate, per 1M population‡	
		1990	2018	1990	2018
	Sugar-sweetened beverages	28.9 (20.2-38.6)	22.1 (14.9-30.8)	899 (631-1215)	1065 (710-1484)
	Potatoes	1.6 (0.9-3.1)	2.3 (1.2-4.4)	51 (27-96)	110 (57-213)
	Fruit	2.4 (1.8-2.9)	2.6 (1.9-3.2)	76 (57-90)	125 (89-152)
	Nuts and seeds	1.2 (1.1-1.3)	1.1 (0.9-1.3)	39 (35-42)	53 (44-61)
	Non-starchy vegetables	1.5 (1.2-1.7)	1 (0.6-1.4)	46 (39-52)	50 (31-68)
	Fruit juice	0.1 (0.1-0.2)	0.2 (0.1-0.3)	4 (2-6)	8 (4-13)
Cote d'Ivoire	Suboptimal diet	54.6 (48.3-62.3)	60.1 (53.7-67.2)	878 (772-1006)	1699 (1513-1913)
	Whole grains	27.9 (18.9-35)	28.1 (18.6-35.4)	446 (302-560)	798 (522-1003)
	Refined grains (total)	10.2 (8-13.8)	11 (8.5-14.5)	164 (128-220)	313 (240-411)
	Refined rice	10.2 (8-13.8)	11 (8.5-14.5)	164 (128-220)	313 (240-411)
	Refined wheat	0 (0-0)	0 (0-0)	0 (0-0)	0 (0-0)
	Processed meats	6.9 (2.1-19)	6.8 (2.3-18.7)	111 (34-310)	193 (66-525)
	Unprocessed red meats	6.6 (4.5-10.3)	5.6 (3.5-8.9)	106 (71-167)	157 (100-252)
	Yogurt	11.7 (10.4-12.7)	11.7 (10.5-12.8)	188 (167-206)	332 (295-365)
	Sugar-sweetened beverages	13.4 (9-19.3)	22 (15.2-30.1)	216 (145-313)	623 (426-849)
	Potatoes	0.5 (0.3-1)	2.6 (1.4-5.3)	8 (5-16)	74 (40-150)
	Fruit	3 (2.8-3.2)	3.4 (3.2-3.7)	48 (45-52)	98 (91-104)
	Nuts and seeds	1.1 (1-1.2)	1.1 (0.9-1.2)	18 (16-20)	30 (25-35)
	Non-starchy vegetables	1.3 (1.2-1.4)	1.5 (1.4-1.7)	21 (19-23)	43 (39-48)
	Fruit juice	0.1 (0-0.1)	0.1 (0.1-0.1)	1 (1-2)	2 (2-4)
Croatia	Suboptimal diet	78.1 (70.5-85.2)	96.8 (94.1-97.8)	2876 (2589-3165)	5144 (4935-5318)
	Whole grains	29 (23.4-33.9)	21.2 (14.5-27.8)	1072 (866-1252)	1126 (772-1479)
	Refined grains (total)	6.5 (4.4-9.5)	17.3 (12.5-23.7)	240 (162-354)	919 (658-1268)
	Refined rice	0.1 (0.1-0.2)	1 (0.7-1.6)	5 (3-7)	53 (37-83)
	Refined wheat	6.4 (4.4-9.3)	16.5 (11.9-22.7)	236 (159-349)	877 (627-1213)
	Processed meats	40.5 (24.5-58.3)	81.5 (66-87)	1487 (902-2142)	4329 (3506-4675)
	Unprocessed red meats	27.7 (17.3-40.1)	70.2 (65.8-72.9)	1018 (639-1480)	3737 (3494-3920)
	Yogurt	6.8 (4.6-8.5)	6.2 (3.9-8.2)	248 (169-314)	331 (211-437)
	Sugar-sweetened beverages	6 (4.4-8.6)	6.5 (4.8-9.5)	221 (162-320)	347 (257-497)
	Potatoes	12.4 (7.8-19.3)	6.5 (4.1-11)	460 (289-720)	347 (215-584)
	Fruit	2.8 (2.7-3)	3.2 (3-3.4)	105 (99-111)	169 (158-180)
	Nuts and seeds	1.2 (1.1-1.3)	1 (0.8-1.2)	45 (42-49)	55 (41-66)
	Non-starchy vegetables	1.3 (1.2-1.4)	0 (0-0.1)	47 (43-51)	3 (2-4)
	Fruit juice	0.1 (0-0.3)	0.1 (0-0.2)	4 (2-9)	5 (2-12)
Cuba	Suboptimal diet	89.6 (85.8-93.1)	87.2 (82.1-91.7)	3688 (3489-3879)	5077 (4726-5400)

Table S5. Proportional and absolute burden per 1M population of T2D incidence attributable to suboptimal diet* and each individual dietary factor† at the national level in 1990 and 2018

Country	Dietary factor	Proportional burden, %		Rate, per 1M population‡	
		1990	2018	1990	2018
	Whole grains	41.5 (40.4-42.7)	26.5 (18.5-32.6)	1710 (1636-1784)	1546 (1074-1916)
	Refined grains (total)	16.5 (11.7-22.5)	17.2 (12.2-23.7)	678 (477-922)	1002 (712-1382)
	Refined rice	9.7 (6.7-13.4)	12.1 (8.5-17.2)	399 (275-557)	705 (494-999)
	Refined wheat	7.8 (5.4-10.9)	6 (4.2-8.7)	323 (221-452)	353 (242-511)
	Processed meats	46.2 (30.1-62.9)	39.7 (23.6-58)	1905 (1240-2586)	2315 (1366-3390)
	Unprocessed red meats	36.6 (28.5-44.8)	48.4 (38.6-57.2)	1508 (1175-1861)	2823 (2217-3355)
	Yogurt	11.2 (9.9-12.2)	11.2 (9.9-12.3)	462 (409-509)	653 (570-719)
	Sugar-sweetened beverages	38.5 (31-46.3)	28.9 (22.2-37.4)	1583 (1283-1914)	1675 (1307-2176)
	Potatoes	1.2 (0.7-2.3)	1 (0.5-2)	49 (27-96)	57 (31-116)
	Fruit	2.9 (2.7-3.1)	2.5 (2.3-2.8)	119 (110-128)	148 (129-166)
	Nuts and seeds	1.3 (1.2-1.4)	1.4 (1.3-1.5)	53 (49-57)	82 (76-89)
	Non-starchy vegetables	1.8 (1.7-1.9)	1.4 (1.2-1.6)	73 (68-78)	80 (70-91)
	Fruit juice	0.2 (0.1-0.3)	0.2 (0.1-0.2)	6 (4-11)	9 (5-15)
Cyprus	Suboptimal diet	59.2 (50.1-68.4)	62.8 (54.6-71.5)	3114 (2650-3599)	3907 (3403-4496)
	Whole grains	14.4 (9.8-19.8)	10.9 (7-15.9)	756 (518-1038)	679 (433-984)
	Refined grains (total)	5.1 (3.7-7.4)	9.6 (6.8-13.3)	271 (194-395)	599 (421-822)
	Refined rice	0.3 (0.2-0.4)	1 (0.7-1.5)	16 (11-24)	64 (45-94)
	Refined wheat	4.9 (3.5-7)	8.7 (6.1-12.1)	257 (184-373)	541 (380-747)
	Processed meats	10.5 (5-19.8)	13.3 (6.5-23.4)	549 (264-1048)	824 (400-1458)
	Unprocessed red meats	29.5 (18.4-42.8)	29 (18.2-42.6)	1546 (966-2259)	1796 (1122-2645)
	Yogurt	7.4 (5.7-9)	8.2 (6.4-9.8)	387 (294-472)	512 (398-610)
	Sugar-sweetened beverages	6.8 (3.5-13.4)	9.1 (4.7-17.5)	356 (181-706)	567 (293-1096)
	Potatoes	8.1 (5.6-12.7)	8.2 (5.6-12.6)	427 (287-672)	510 (350-782)
	Fruit	1.1 (0.5-1.9)	3.2 (2.6-3.5)	60 (24-100)	196 (163-223)
	Nuts and seeds	0.9 (0.7-1.1)	1.1 (0.9-1.3)	49 (38-58)	69 (54-81)
	Non-starchy vegetables	0.7 (0.3-1.1)	1.2 (0.6-1.6)	37 (13-60)	73 (39-99)
	Fruit juice	0.1 (0.1-0.3)	0.2 (0.1-0.4)	7 (3-15)	12 (5-27)
Czech Republic	Suboptimal diet	79.2 (72.9-85.2)	74.7 (67.7-81.6)	3420 (3107-3740)	5613 (5033-6149)
	Whole grains	24.7 (20.9-28.1)	24.8 (20.8-28.2)	1067 (894-1226)	1860 (1560-2123)
	Refined grains (total)	0.9 (0.6-1.5)	1.9 (1.2-3.2)	39 (24-64)	145 (91-239)
	Refined rice	0.1 (0-0.1)	0.2 (0.1-0.3)	2 (1-3)	15 (10-24)
	Refined wheat	0.9 (0.5-1.4)	1.7 (1.1-2.8)	37 (23-60)	130 (82-214)
	Processed meats	42.9 (28.8-58.2)	43.8 (29.7-58.4)	1848 (1234-2530)	3301 (2224-4367)
	Unprocessed red meats	37.7 (30.4-44.9)	24.5 (18.7-30.7)	1623 (1311-1945)	1834 (1415-2328)
	Yogurt	6 (3.7-8)	5.9 (3.7-7.9)	258 (161-350)	444 (278-596)

Table S5. Proportional and absolute burden per 1M population of T2D incidence attributable to suboptimal diet* and each individual dietary factor† at the national level in 1990 and 2018

Country	Dietary factor	Proportional burden, %		Rate, per 1M population‡	
		1990	2018	1990	2018
	Sugar-sweetened beverages	5.6 (4.3-8.4)	4.7 (3.6-7)	244 (186-360)	352 (274-525)
	Potatoes	7.3 (5.7-10)	5.9 (4.6-8)	314 (244-434)	443 (347-601)
	Fruit	2.8 (2.6-2.9)	3.1 (2.9-3.3)	120 (110-129)	237 (220-252)
	Nuts and seeds	1.2 (1.1-1.3)	1.3 (1.2-1.4)	52 (47-56)	97 (89-105)
	Non-starchy vegetables	1.2 (1.1-1.3)	1.3 (1.2-1.5)	50 (45-55)	100 (91-110)
	Fruit juice	0.1 (0-0.3)	0.1 (0-0.3)	4 (1-12)	7 (3-22)
Democratic Republic of the Congo	Suboptimal diet	62 (55.6-71.7)	71 (61.3-81.9)	1569 (1397-1809)	2395 (2059-2786)
	Whole grains	39.4 (35.8-41.5)	37.2 (29.1-41.3)	996 (896-1058)	1258 (983-1408)
	Refined grains (total)	1.1 (0.9-1.4)	10.2 (8.4-12.7)	27 (22-35)	348 (284-433)
	Refined rice	0.3 (0.2-0.3)	3.9 (3.2-5)	6 (5-8)	132 (108-170)
	Refined wheat	0.8 (0.7-1.1)	6.6 (5.4-8.2)	21 (17-27)	224 (183-278)
	Processed meats	18 (6.5-37.9)	33.7 (16-57.5)	458 (165-964)	1139 (540-1941)
	Unprocessed red meats	1.8 (1.1-4.1)	5 (3.4-8.3)	46 (28-104)	168 (113-280)
	Yogurt	12.2 (11.1-13.1)	12.8 (11.7-13.8)	308 (280-336)	433 (393-472)
	Sugar-sweetened beverages	6.5 (2.3-16.9)	9.2 (3.3-23.9)	165 (59-438)	313 (110-795)
	Potatoes	1 (0.5-1.9)	3.4 (1.9-6.7)	24 (13-47)	113 (63-231)
	Fruit	2.5 (2.3-2.7)	3 (2.7-3.2)	64 (59-69)	100 (92-109)
	Nuts and seeds	0.5 (0.4-0.8)	0.7 (0.5-1)	14 (10-20)	23 (16-33)
	Non-starchy vegetables	0.5 (0.4-0.6)	0.6 (0.4-0.7)	12 (9-15)	20 (15-25)
	Fruit juice	0.1 (0-0.1)	0.1 (0.1-0.2)	2 (1-3)	3 (2-5)
Denmark	Suboptimal diet	71.2 (65-77.9)	68.5 (61.4-75.7)	1217 (1095-1341)	2342 (2100-2608)
	Whole grains	13.5 (9.8-17.1)	20.1 (16.7-23.2)	230 (167-293)	684 (570-803)
	Refined grains (total)	0.5 (0.3-0.7)	1.9 (1.2-3)	8 (5-13)	64 (40-101)
	Refined rice	0 (0-0)	0.1 (0.1-0.2)	0 (0-1)	5 (3-8)
	Refined wheat	0.4 (0.3-0.7)	1.7 (1.1-2.7)	8 (5-12)	59 (37-93)
	Processed meats	35.3 (24.8-48.2)	36.6 (25.2-50.1)	604 (419-816)	1251 (867-1719)
	Unprocessed red meats	34.7 (27.3-42.7)	21.4 (15.8-28.6)	591 (464-725)	731 (542-973)
	Yogurt	7.6 (5.5-9.3)	7.3 (5.3-9.1)	129 (93-158)	250 (183-313)
	Sugar-sweetened beverages	5.2 (4.1-8)	6.3 (5-9.5)	88 (71-135)	217 (171-326)
	Potatoes	4.6 (3.8-6.7)	3.7 (3.1-5.3)	79 (64-113)	127 (105-183)
	Fruit	2.4 (2.2-2.6)	2.7 (2.5-3)	41 (37-45)	94 (84-103)
	Nuts and seeds	1.2 (1.1-1.3)	1 (0.8-1.1)	20 (18-22)	33 (28-37)
	Non-starchy vegetables	1.3 (1.2-1.5)	1.2 (1-1.3)	23 (21-25)	40 (34-45)
	Fruit juice	0.2 (0.1-0.3)	0.2 (0.1-0.3)	3 (2-5)	7 (4-11)
Djibouti	Suboptimal diet	79 (73.3-84.1)	89 (84.4-92.9)	915 (840-989)	2316 (2177-2462)

Table S5. Proportional and absolute burden per 1M population of T2D incidence attributable to suboptimal diet* and each individual dietary factor† at the national level in 1990 and 2018

Country	Dietary factor	Proportional burden, %		Rate, per 1M population‡	
		1990	2018	1990	2018
	Whole grains	8 (4-14.9)	18.2 (10.2-27.1)	92 (46-173)	469 (263-700)
	Refined grains (total)	15.8 (11.9-21.2)	18 (12.9-24.2)	183 (138-247)	467 (336-630)
	Refined rice	9.9 (7.2-13.6)	8.1 (5.7-11.5)	114 (85-159)	211 (148-302)
	Refined wheat	6.9 (5.1-9.8)	11 (7.8-15.2)	80 (59-114)	286 (202-394)
	Processed meats	11.3 (3.9-26)	24.5 (9.6-46.4)	130 (45-304)	637 (252-1211)
	Unprocessed red meats	7 (4.7-10.9)	9.7 (6.8-14.5)	81 (54-125)	252 (176-382)
	Yogurt	12 (10.8-13.1)	12 (10.6-13.2)	139 (124-153)	312 (275-346)
	Sugar-sweetened beverages	35.9 (25.3-46.4)	52 (40.3-63.5)	416 (293-533)	1345 (1043-1658)
	Potatoes	39.3 (32-44.7)	43.9 (40.3-48.9)	456 (368-521)	1141 (1036-1288)
	Fruit	3.4 (3.2-3.6)	2.4 (2-2.7)	39 (37-42)	61 (53-69)
	Nuts and seeds	1.4 (1.3-1.5)	1.5 (1.4-1.6)	16 (15-17)	39 (36-43)
	Non-starchy vegetables	1.2 (1.1-1.4)	1.5 (1.3-1.6)	14 (12-16)	38 (33-43)
	Fruit juice	0.2 (0.1-0.3)	0.1 (0.1-0.2)	2 (1-3)	3 (2-6)
Dominica	Suboptimal diet	78.4 (72.6-83.8)	65.6 (59.9-72.1)	3957 (3658-4269)	5237 (4730-5808)
	Whole grains	33.8 (28.7-36.8)	28.1 (20-34.2)	1703 (1452-1870)	2237 (1606-2740)
	Refined grains (total)	27.7 (20.5-35.6)	17.5 (12.1-24.1)	1395 (1044-1806)	1390 (962-1932)
	Refined rice	7.2 (5-9.8)	3 (2-4.5)	360 (255-499)	240 (160-359)
	Refined wheat	22.7 (16.6-29.5)	15.1 (10.3-20.9)	1142 (848-1494)	1200 (825-1679)
	Processed meats	30.5 (16.8-45.8)	15.4 (7.4-28.9)	1542 (850-2317)	1226 (589-2312)
	Unprocessed red meats	15 (10.7-21)	8.8 (5.6-13)	754 (543-1060)	698 (441-1051)
	Yogurt	10.7 (9.4-11.6)	11.5 (10.2-12.6)	538 (474-589)	916 (807-1012)
	Sugar-sweetened beverages	26.1 (18.7-35.5)	19.1 (13-26.9)	1321 (941-1803)	1515 (1041-2159)
	Potatoes	0.6 (0.3-1)	4.4 (2.3-8.5)	28 (15-51)	347 (179-678)
	Fruit	2.4 (2.2-2.7)	2.9 (2.5-3.2)	123 (108-135)	229 (200-258)
	Nuts and seeds	1.3 (1.2-1.3)	1.2 (1.1-1.4)	64 (60-68)	98 (85-110)
	Non-starchy vegetables	1.2 (1-1.3)	1.5 (1.3-1.7)	60 (52-67)	118 (102-133)
	Fruit juice	0.1 (0.1-0.2)	0.1 (0.1-0.2)	5 (3-8)	9 (5-15)
Dominican Republic	Suboptimal diet	74 (69.1-79.8)	81.3 (76.1-86.7)	1233 (1141-1341)	2611 (2432-2814)
	Whole grains	34.8 (28.3-38.3)	33.8 (26.6-38.1)	580 (469-641)	1084 (855-1229)
	Refined grains (total)	22.7 (19.7-26)	22.2 (19.2-25.8)	379 (328-434)	714 (614-828)
	Refined rice	19 (16.5-22)	17.6 (15.1-20.7)	317 (273-366)	565 (483-665)
	Refined wheat	4.6 (3.9-5.6)	5.6 (4.8-7.1)	76 (65-94)	180 (152-229)
	Processed meats	27 (14.6-42.9)	37.3 (22.3-54.4)	449 (242-718)	1202 (725-1756)
	Unprocessed red meats	19 (15.7-22.7)	20.5 (16.9-24.5)	316 (261-379)	657 (541-788)
	Yogurt	13 (12.3-13.6)	13 (12.2-13.7)	216 (203-228)	417 (387-448)

Table S5. Proportional and absolute burden per 1M population of T2D incidence attributable to suboptimal diet* and each individual dietary factor† at the national level in 1990 and 2018

Country	Dietary factor	Proportional burden, %		Rate, per 1M population‡	
		1990	2018	1990	2018
	Sugar-sweetened beverages	13.7 (10-18.5)	24.4 (18.6-31.4)	228 (168-310)	782 (597-1007)
	Potatoes	0.8 (0.4-1.6)	3.9 (2-7.3)	14 (7-27)	124 (63-238)
	Fruit	2.6 (2.5-2.7)	2.4 (2.2-2.6)	43 (41-46)	77 (70-85)
	Nuts and seeds	1.3 (1.2-1.3)	1.4 (1.3-1.5)	21 (19-22)	45 (41-49)
	Non-starchy vegetables	1.9 (1.8-2)	1.5 (1.4-1.6)	32 (30-34)	47 (43-52)
	Fruit juice	0.3 (0.2-0.4)	0.3 (0.2-0.4)	5 (4-6)	10 (8-13)
Ecuador	Suboptimal diet	77.4 (71.8-82.1)	72.1 (66.3-77.7)	1686 (1557-1801)	3133 (2871-3394)
	Whole grains	25.6 (17.9-31.9)	26.2 (18.4-32.5)	557 (387-696)	1142 (804-1419)
	Refined grains (total)	15.8 (10.3-23.3)	15.7 (10-22.8)	344 (225-506)	680 (438-987)
	Refined rice	11.2 (7.2-16.9)	9.3 (5.9-13.8)	244 (157-366)	404 (257-601)
	Refined wheat	5.2 (3.2-7.9)	6.9 (4.4-10.4)	112 (71-172)	302 (191-453)
	Processed meats	4.4 (2-9)	4.9 (2.3-10.6)	95 (44-196)	214 (102-462)
	Unprocessed red meats	19.4 (11-30.5)	20.1 (11.4-32.2)	422 (239-667)	875 (495-1400)
	Yogurt	11.2 (9.9-12.1)	10.9 (9.5-11.9)	244 (216-264)	474 (413-520)
	Sugar-sweetened beverages	47.9 (39.9-55.6)	35.5 (28.5-44.1)	1044 (862-1218)	1545 (1231-1912)
	Potatoes	6 (3.9-9.8)	3 (1.9-5.2)	132 (84-216)	130 (83-225)
	Fruit	2.4 (2.1-2.6)	3.9 (3.7-4)	51 (46-56)	168 (159-177)
	Nuts and seeds	1.3 (1.2-1.3)	1.2 (1.1-1.4)	28 (25-29)	54 (46-61)
	Non-starchy vegetables	1.2 (1.1-1.3)	1.4 (1.2-1.5)	26 (24-29)	60 (54-66)
	Fruit juice	0.2 (0.2-0.3)	0.3 (0.2-0.5)	5 (3-7)	14 (10-20)
Egypt	Suboptimal diet	57.9 (53.4-63.2)	65.2 (60.5-70.9)	1083 (996-1180)	2723 (2522-2976)
	Whole grains	30.6 (22.9-36.7)	31.3 (22-37.3)	573 (427-687)	1303 (918-1568)
	Refined grains (total)	20.6 (17.5-23.9)	21 (17.8-24.6)	384 (327-448)	879 (742-1030)
	Refined rice	6.4 (5.4-7.9)	7 (5.8-8.6)	120 (100-147)	294 (242-355)
	Refined wheat	15.1 (12.9-17.8)	15 (12.6-17.8)	283 (239-333)	628 (527-747)
	Processed meats	7.2 (2.5-16.9)	10.8 (4.6-23)	136 (47-318)	454 (186-965)
	Unprocessed red meats	5.3 (3.7-8.1)	11.9 (9.3-15.8)	100 (69-152)	495 (389-660)
	Yogurt	11.6 (10.8-12.4)	11.9 (11.1-12.7)	216 (200-233)	498 (462-536)
	Sugar-sweetened beverages	10.8 (8.3-14)	10.6 (8-14)	201 (156-264)	442 (335-583)
	Potatoes	3.1 (1.7-5.6)	8.6 (4.9-14.5)	58 (32-103)	359 (208-608)
	Fruit	2.5 (2.4-2.7)	3 (2.8-3.2)	47 (44-50)	125 (116-134)
	Nuts and seeds	1.3 (1.2-1.4)	1.4 (1.3-1.5)	24 (23-26)	58 (53-62)
	Non-starchy vegetables	0.8 (0.7-0.9)	1 (0.8-1.1)	15 (12-17)	40 (34-46)
	Fruit juice	0.1 (0.1-0.1)	0.1 (0.1-0.2)	2 (1-3)	5 (4-6)
El Salvador	Suboptimal diet	70.8 (63.5-79.3)	85.4 (79.6-90.7)	1862 (1671-2090)	4198 (3887-4488)

Table S5. Proportional and absolute burden per 1M population of T2D incidence attributable to suboptimal diet* and each individual dietary factor† at the national level in 1990 and 2018

Country	Dietary factor	Proportional burden, %		Rate, per 1M population‡	
		1990	2018	1990	2018
	Whole grains	28.9 (20.9-34.6)	31 (22.3-36.6)	757 (555-914)	1521 (1097-1801)
	Refined grains (total)	17.9 (12.6-24.2)	22.1 (15.9-29.5)	471 (330-644)	1084 (787-1457)
	Refined rice	6.8 (4.6-9.7)	9.7 (6.9-13.6)	178 (120-254)	477 (337-667)
	Refined wheat	12.3 (8.5-17)	14.2 (10.1-19.5)	322 (222-448)	696 (494-958)
	Processed meats	32.2 (18.1-50)	47.4 (31.3-65.1)	847 (476-1328)	2329 (1540-3202)
	Unprocessed red meats	7.9 (5-12.1)	19.9 (14.1-27.5)	210 (130-319)	983 (694-1356)
	Yogurt	12 (10.9-12.9)	12 (10.5-13)	316 (286-342)	588 (520-644)
	Sugar-sweetened beverages	17.7 (12-25.8)	32.1 (23-43.7)	468 (317-676)	1576 (1131-2158)
	Potatoes	0.4 (0.2-0.7)	4.1 (2.2-7.9)	10 (5-19)	202 (107-385)
	Fruit	2.6 (2-3)	2.7 (1.9-3.3)	67 (52-78)	134 (96-161)
	Nuts and seeds	1.2 (1.1-1.3)	1.3 (1.2-1.4)	32 (29-35)	65 (58-71)
	Non-starchy vegetables	1.5 (1.3-1.7)	1.3 (0.9-1.6)	40 (35-45)	64 (44-81)
	Fruit juice	0.1 (0.1-0.2)	0.2 (0.1-0.3)	4 (2-6)	8 (4-14)
Equatorial Guinea	Suboptimal diet	56.5 (49.7-63.2)	71.3 (65.3-77.2)	1377 (1205-1544)	2034 (1843-2218)
	Whole grains	27.8 (18.7-34.7)	28.8 (19.5-36.7)	676 (456-855)	818 (552-1052)
	Refined grains (total)	9.7 (6.5-14.2)	20.2 (14.3-27.6)	235 (157-347)	576 (405-795)
	Refined rice	9.7 (6.5-14.2)	20.2 (14.3-27.6)	235 (157-347)	576 (405-795)
	Refined wheat	0 (0-0)	0 (0-0)	0 (0-0)	0 (0-0)
	Processed meats	7.1 (2.6-19.5)	6.6 (2.1-17.3)	174 (62-472)	187 (59-500)
	Unprocessed red meats	9.5 (6.7-13.1)	12.8 (8.9-17.6)	231 (163-319)	362 (253-500)
	Yogurt	11.9 (10.7-12.9)	12.6 (11.4-13.8)	289 (260-316)	358 (322-396)
	Sugar-sweetened beverages	13.5 (9.4-19.3)	25.8 (18.1-36)	329 (229-471)	736 (515-1037)
	Potatoes	1.3 (0.7-2.6)	13.1 (7.4-21.4)	33 (18-63)	373 (209-607)
	Fruit	2.8 (2.6-3)	3.1 (2.9-3.4)	68 (64-73)	89 (81-97)
	Nuts and seeds	0.9 (0.7-1)	1.1 (0.9-1.3)	21 (17-25)	31 (25-36)
	Non-starchy vegetables	0.9 (0.8-1.1)	1.2 (0.9-1.4)	23 (19-27)	33 (26-40)
	Fruit juice	0.1 (0-0.1)	0.1 (0.1-0.2)	2 (1-3)	3 (2-5)
Eritrea	Suboptimal diet	62.2 (56.4-68)	72.2 (66.2-77.7)	1326 (1193-1450)	3162 (2895-3430)
	Whole grains	29 (19.2-36.2)	28.9 (19.4-36.2)	615 (410-772)	1261 (847-1585)
	Refined grains (total)	19 (13.7-26)	21.5 (15.8-28.6)	407 (289-554)	941 (688-1261)
	Refined rice	19 (13.7-26)	21.5 (15.8-28.6)	407 (289-554)	941 (688-1261)
	Refined wheat	0 (0-0)	0 (0-0)	0 (0-0)	0 (0-0)
	Processed meats	4 (1.2-11.8)	7.4 (2.4-17.7)	86 (26-253)	325 (106-773)
	Unprocessed red meats	8.8 (6.1-13)	11.6 (8.4-15.9)	187 (130-276)	510 (362-692)
	Yogurt	12.6 (11.6-13.6)	12.4 (11.3-13.4)	269 (244-294)	542 (493-592)

Table S5. Proportional and absolute burden per 1M population of T2D incidence attributable to suboptimal diet* and each individual dietary factor† at the national level in 1990 and 2018

Country	Dietary factor	Proportional burden, %		Rate, per 1M population‡	
		1990	2018	1990	2018
	Sugar-sweetened beverages	21.2 (15-29.1)	33.2 (24.5-43.4)	452 (324-621)	1456 (1059-1892)
	Potatoes	1.2 (0.6-2.4)	4.9 (2.7-9.2)	25 (14-50)	213 (120-399)
	Fruit	3.1 (2.8-3.4)	3.5 (3.2-3.8)	66 (59-72)	155 (138-169)
	Nuts and seeds	1.2 (1.1-1.3)	1.1 (0.9-1.2)	26 (23-28)	47 (39-55)
	Non-starchy vegetables	1.5 (1.4-1.7)	1.8 (1.6-1.9)	32 (29-36)	77 (69-85)
	Fruit juice	0.1 (0-0.1)	0.1 (0.1-0.1)	1 (1-2)	4 (2-6)
Estonia	Suboptimal diet	92 (87.2-94.9)	89.8 (86.2-92.6)	1860 (1741-1958)	2632 (2496-2758)
	Whole grains	7.6 (5.1-10.6)	7.9 (5.3-10.6)	155 (103-214)	230 (159-310)
	Refined grains (total)	1 (0.8-1.4)	16.2 (13.2-19.8)	21 (16-29)	476 (388-584)
	Refined rice	0 (0-0)	0.9 (0.7-1.1)	0 (0-0)	25 (21-33)
	Refined wheat	1 (0.8-1.4)	15.5 (12.6-18.9)	21 (16-29)	454 (370-558)
	Processed meats	79.8 (68.4-87)	76.3 (68.2-82.3)	1615 (1373-1778)	2235 (1999-2435)
	Unprocessed red meats	43.7 (39-48.8)	30.9 (26.8-35.8)	886 (783-987)	905 (786-1048)
	Yogurt	2.4 (1.5-3.7)	2.6 (1.6-3.8)	48 (30-74)	77 (48-114)
	Sugar-sweetened beverages	4.8 (3.7-7.3)	5.2 (4.2-7.6)	97 (74-146)	153 (122-225)
	Potatoes	12.1 (10.2-15.3)	11.5 (9.7-14.5)	245 (206-311)	337 (284-427)
	Fruit	2.5 (2.3-2.7)	2.8 (2.6-3)	51 (47-56)	82 (75-89)
	Nuts and seeds	1.3 (1.2-1.4)	1.1 (1-1.2)	26 (24-28)	33 (29-36)
	Non-starchy vegetables	1.1 (1-1.2)	0.2 (0.2-0.3)	22 (20-25)	7 (5-10)
	Fruit juice	0.8 (0.6-1.1)	0.6 (0.5-0.9)	16 (11-23)	18 (13-25)
Ethiopia	Suboptimal diet	52.7 (47.9-60.3)	70.8 (62.3-84.1)	1089 (985-1251)	976 (856-1156)
	Whole grains	2.9 (1.8-4.5)	3 (1.8-4.4)	60 (37-93)	41 (24-61)
	Refined grains (total)	17.5 (15-20.9)	26.2 (22.3-30.8)	360 (307-431)	362 (306-426)
	Refined rice	0.6 (0.5-0.8)	4.6 (3.8-6)	13 (11-16)	64 (53-83)
	Refined wheat	17 (14.5-20.3)	22.7 (19.2-26.8)	350 (298-418)	313 (263-370)
	Processed meats	8.8 (2.9-22.5)	26.1 (7.8-59.1)	182 (59-466)	359 (108-819)
	Unprocessed red meats	4.3 (2.9-7.4)	4.3 (3-7.3)	88 (60-155)	60 (42-101)
	Yogurt	12.1 (10.8-13.2)	12 (10.7-13)	249 (222-274)	165 (146-181)
	Sugar-sweetened beverages	14.3 (10.9-19.2)	25.9 (20.4-32.2)	297 (224-398)	357 (282-448)
	Potatoes	5 (3.7-7.5)	5.7 (4.3-8.1)	103 (76-155)	79 (58-111)
	Fruit	3.4 (3.2-3.5)	3.7 (3.6-3.9)	69 (66-74)	51 (49-54)
	Nuts and seeds	1.3 (1.2-1.4)	1.4 (1.3-1.5)	26 (24-28)	19 (17-20)
	Non-starchy vegetables	1.4 (1.3-1.5)	1.5 (1.4-1.7)	28 (26-30)	21 (20-23)
	Fruit juice	0 (0-0)	0 (0-0)	0 (0-1)	0 (0-1)
Federated States of Micronesia	Suboptimal diet	68.2 (61.2-75.1)	76.1 (68.4-83.2)	3018 (2684-3333)	6100 (5445-6691)

Table S5. Proportional and absolute burden per 1M population of T2D incidence attributable to suboptimal diet* and each individual dietary factor† at the national level in 1990 and 2018

Country	Dietary factor	Proportional burden, %		Rate, per 1M population‡	
		1990	2018	1990	2018
	Whole grains	38.1 (33.1-41.4)	30.2 (22.1-36.3)	1686 (1458-1835)	2413 (1776-2900)
	Refined grains (total)	34.3 (23.5-44.9)	37.7 (26-48.9)	1518 (1039-2007)	3024 (2072-3927)
	Refined rice	25.7 (17-34.5)	28 (18.6-37.7)	1133 (753-1539)	2246 (1489-3004)
	Refined wheat	13.1 (8.4-18.2)	15 (9.7-21.2)	579 (372-816)	1203 (767-1684)
	Processed meats	13.7 (6.1-26.9)	23.2 (11.3-42.4)	605 (267-1178)	1865 (887-3426)
	Unprocessed red meats	9.1 (6-13.4)	14.5 (9.5-20.5)	402 (264-591)	1164 (755-1636)
	Yogurt	13.4 (12.4-14.3)	13.7 (12.7-14.6)	591 (546-635)	1098 (1010-1181)
	Sugar-sweetened beverages	4.8 (3.2-7.6)	13 (9-18.7)	211 (139-336)	1044 (725-1487)
	Potatoes	0.2 (0.1-0.4)	0.1 (0-0.2)	9 (4-18)	7 (3-15)
	Fruit	2.7 (2.2-3.1)	2.6 (1.9-3.2)	121 (96-139)	213 (153-260)
	Nuts and seeds	1.2 (1.1-1.4)	1.4 (1.3-1.6)	54 (48-61)	114 (101-126)
	Non-starchy vegetables	0.8 (0.3-1.2)	1.3 (0.7-1.7)	36 (14-54)	103 (56-136)
	Fruit juice	0 (0-0.1)	0 (0-0.1)	2 (1-3)	4 (2-6)
Fiji	Suboptimal diet	71.1 (64-77.7)	63.8 (57-70.6)	5104 (4571-5597)	8121 (7237-9031)
	Whole grains	25.1 (16.9-32)	24.6 (17.4-31.3)	1810 (1213-2308)	3125 (2203-4014)
	Refined grains (total)	29 (19.5-39.2)	29.6 (19.8-40.6)	2082 (1396-2830)	3763 (2514-5159)
	Refined rice	16.8 (10.7-23.7)	17.2 (11-24.8)	1208 (769-1707)	2190 (1399-3156)
	Refined wheat	15.9 (10-22.6)	16 (10.2-23.3)	1142 (724-1626)	2034 (1298-2957)
	Processed meats	17.3 (9.2-32)	9.3 (4.4-18.6)	1249 (659-2289)	1180 (562-2395)
	Unprocessed red meats	25.6 (19-33.2)	7.8 (5.1-11.8)	1840 (1367-2388)	997 (653-1508)
	Yogurt	13.1 (12.1-14)	13 (11.9-13.8)	946 (871-1012)	1651 (1510-1777)
	Sugar-sweetened beverages	4.8 (3.3-7.4)	11.5 (8.2-16.3)	344 (236-531)	1468 (1060-2073)
	Potatoes	0.8 (0.4-1.6)	1.8 (0.9-3.6)	56 (29-117)	228 (115-466)
	Fruit	2.7 (2.1-3.1)	3 (2.3-3.5)	193 (149-227)	383 (294-449)
	Nuts and seeds	1.4 (1.3-1.5)	1.3 (1.1-1.4)	102 (96-109)	163 (142-183)
	Non-starchy vegetables	0.9 (0.6-1.3)	0.7 (0.3-1.1)	67 (41-91)	87 (39-144)
	Fruit juice	0.1 (0-0.1)	0.1 (0-0.1)	4 (3-8)	7 (4-12)
Finland	Suboptimal diet	73.5 (69.3-78.1)	80 (75.6-84.3)	2388 (2223-2562)	3841 (3596-4085)
	Whole grains	17.3 (14.9-19.6)	16.4 (13.8-19.1)	563 (482-640)	790 (662-919)
	Refined grains (total)	9.4 (5.8-14.4)	10.2 (6.3-15.6)	304 (187-467)	489 (298-752)
	Refined rice	1 (0.6-1.6)	1.1 (0.6-1.7)	31 (19-52)	51 (31-83)
	Refined wheat	8.5 (5.2-13.1)	9.2 (5.6-14.2)	276 (169-424)	443 (269-682)
	Processed meats	45.9 (38.8-53.7)	59.3 (51.5-67.2)	1492 (1254-1754)	2845 (2453-3248)
	Unprocessed red meats	23.2 (19.3-27.9)	25 (21.1-29.9)	754 (629-912)	1200 (1010-1452)
	Yogurt	6.8 (4.4-8.6)	6.4 (4.1-8.2)	220 (144-278)	305 (193-395)

Table S5. Proportional and absolute burden per 1M population of T2D incidence attributable to suboptimal diet* and each individual dietary factor† at the national level in 1990 and 2018

Country	Dietary factor	Proportional burden, %		Rate, per 1M population‡	
		1990	2018	1990	2018
	Sugar-sweetened beverages	6.4 (5.3-9.2)	5.5 (4.5-8.3)	208 (170-301)	262 (216-404)
	Potatoes	5 (4.2-6.7)	4.4 (3.7-6)	161 (135-217)	212 (178-290)
	Fruit	2.3 (2.1-2.5)	2 (1.8-2.2)	74 (68-80)	96 (84-108)
	Nuts and seeds	1.1 (1-1.2)	1.2 (1.1-1.3)	37 (34-39)	57 (52-62)
	Non-starchy vegetables	1.2 (1.2-1.3)	1.1 (1-1.3)	40 (37-44)	55 (49-61)
	Fruit juice	0.2 (0.1-0.2)	0.2 (0.2-0.2)	6 (4-7)	9 (7-12)
France	Suboptimal diet	72.5 (69.6-76)	70.9 (67.6-74.6)	1153 (1092-1218)	1804 (1712-1916)
	Whole grains	26.9 (25.3-28.4)	23.9 (22.2-25.8)	426 (399-455)	609 (562-662)
	Refined grains (total)	10.1 (8.5-12.3)	14.5 (12.2-17.1)	161 (135-197)	369 (311-439)
	Refined rice	0.8 (0.6-1)	1.3 (1.1-1.7)	12 (10-16)	33 (27-42)
	Refined wheat	9.4 (8-11.5)	13.4 (11.3-15.9)	150 (125-184)	341 (286-407)
	Processed meats	35.5 (29.5-42.3)	38.6 (32.5-45.9)	564 (467-678)	984 (832-1170)
	Unprocessed red meats	25.3 (22.2-29.2)	19.8 (17-23.6)	402 (352-467)	504 (431-607)
	Yogurt	5.5 (4.7-6.3)	5.5 (4.7-6.3)	88 (75-100)	141 (119-161)
	Sugar-sweetened beverages	6.4 (5.4-8.7)	9.4 (7.8-12.3)	101 (85-138)	238 (199-313)
	Potatoes	7.3 (6.5-9.3)	2.8 (2.5-3.5)	116 (102-148)	71 (64-89)
	Fruit	2.8 (2.7-2.9)	1.9 (1.7-2.1)	45 (43-47)	48 (43-53)
	Nuts and seeds	1.1 (1-1.2)	1.2 (1.1-1.3)	18 (16-19)	31 (28-33)
	Non-starchy vegetables	0.8 (0.7-0.8)	0.9 (0.8-1)	12 (11-13)	22 (20-25)
	Fruit juice	0.1 (0.1-0.1)	0.1 (0.1-0.1)	1 (1-1)	2 (2-2)
Gabon	Suboptimal diet	68 (60.8-75.5)	66.1 (59.6-72.8)	2182 (1944-2429)	2400 (2146-2670)
	Whole grains	23 (15.1-30.2)	25 (16.8-32.6)	738 (480-965)	905 (607-1185)
	Refined grains (total)	4.7 (3.6-6.2)	12 (9.7-15)	152 (117-199)	435 (349-547)
	Refined rice	2.2 (1.7-3.2)	6.8 (5.5-8.9)	72 (56-101)	248 (197-320)
	Refined wheat	2.5 (1.9-3.5)	5.5 (4.4-7.2)	81 (62-110)	199 (159-259)
	Processed meats	10.8 (3.7-26.3)	9.6 (3.2-24.5)	347 (118-833)	349 (115-882)
	Unprocessed red meats	16.8 (12.6-21.8)	14.7 (11.1-20.4)	537 (406-706)	533 (401-742)
	Yogurt	11.9 (10.8-12.8)	12.5 (11.4-13.7)	381 (345-414)	453 (407-502)
	Sugar-sweetened beverages	32.4 (23.5-42.1)	23.9 (16.2-32.6)	1035 (753-1349)	867 (586-1195)
	Potatoes	1.1 (0.6-2.1)	5 (2.7-9.1)	34 (19-67)	181 (99-332)
	Fruit	2.5 (2.3-2.7)	3.3 (3.1-3.6)	80 (73-88)	120 (110-131)
	Nuts and seeds	0.8 (0.6-0.9)	0.9 (0.7-1.1)	24 (20-29)	32 (25-39)
	Non-starchy vegetables	1.1 (0.9-1.2)	1.3 (1.1-1.4)	34 (30-38)	47 (41-53)
	Fruit juice	0.1 (0.1-0.1)	0.1 (0.1-0.2)	3 (2-4)	5 (3-7)
Georgia	Suboptimal diet	71.8 (62.5-81.3)	86.6 (79.6-92.1)	1626 (1405-1846)	4005 (3656-4301)

Table S5. Proportional and absolute burden per 1M population of T2D incidence attributable to suboptimal diet* and each individual dietary factor† at the national level in 1990 and 2018

Country	Dietary factor	Proportional burden, %		Rate, per 1M population‡	
		1990	2018	1990	2018
	Whole grains	28.8 (21.9-34.5)	26.3 (19.2-32.9)	655 (500-778)	1212 (883-1524)
	Refined grains (total)	6.6 (4.5-10.1)	37.5 (28.4-46.8)	149 (101-229)	1740 (1318-2180)
	Refined rice	0 (0-0.1)	1.7 (1.2-2.3)	1 (1-2)	77 (54-107)
	Refined wheat	6.5 (4.4-10)	36.6 (27.7-45.7)	148 (101-228)	1698 (1284-2130)
	Processed meats	44 (28.4-61.1)	62.6 (45-77.6)	1000 (629-1398)	2898 (2076-3604)
	Unprocessed red meats	9.5 (4.1-18.5)	5.4 (2.3-11.1)	216 (93-413)	248 (106-518)
	Yogurt	8.5 (6-10.4)	8.4 (5.7-10.3)	192 (135-239)	388 (264-485)
	Sugar-sweetened beverages	0.3 (0.2-0.9)	6.8 (4.7-10.5)	7 (4-20)	314 (215-486)
	Potatoes	9.1 (5.6-15.1)	10.2 (6.2-16.7)	206 (128-344)	469 (286-772)
	Fruit	3 (2.8-3.2)	3.4 (3.2-3.6)	67 (62-72)	157 (145-169)
	Nuts and seeds	1 (0.9-1.2)	1.3 (1.1-1.4)	23 (20-27)	59 (51-65)
	Non-starchy vegetables	1.4 (1.3-1.5)	1.6 (1.4-1.7)	32 (29-35)	72 (65-80)
	Fruit juice	0.1 (0.1-0.2)	0.2 (0.1-0.3)	3 (2-5)	9 (5-15)
Germany	Suboptimal diet	84.5 (82-87.3)	82.7 (79.4-86.3)	3574 (3399-3754)	5091 (4841-5383)
	Whole grains	11.8 (9.5-14)	11.8 (9.6-14.1)	497 (404-596)	726 (589-868)
	Refined grains (total)	22.3 (19.6-25.1)	22.6 (19.8-25.7)	945 (828-1062)	1392 (1214-1592)
	Refined rice	1.5 (1.3-2)	1.8 (1.5-2.4)	64 (56-86)	110 (95-143)
	Refined wheat	21.1 (18.5-23.8)	21.2 (18.5-24.1)	894 (783-1011)	1306 (1137-1502)
	Processed meats	59.5 (53.4-66.6)	60.9 (53.8-68.3)	2526 (2254-2826)	3742 (3300-4250)
	Unprocessed red meats	25.7 (22.8-29.7)	19 (16.4-23.2)	1086 (956-1260)	1174 (1010-1424)
	Yogurt	7.7 (6.8-8.5)	7.6 (6.7-8.5)	324 (287-360)	467 (411-527)
	Sugar-sweetened beverages	8.5 (7-11.4)	9.3 (7.9-12.4)	359 (295-487)	571 (482-764)
	Potatoes	11.8 (10.4-14.8)	5.5 (4.8-7.4)	501 (436-630)	336 (296-458)
	Fruit	1.7 (1.5-1.9)	2.1 (1.9-2.3)	72 (64-79)	130 (117-143)
	Nuts and seeds	1.1 (1-1.2)	1.2 (1.1-1.3)	47 (43-51)	73 (67-80)
	Non-starchy vegetables	1.2 (1.1-1.3)	0.7 (0.6-0.8)	50 (46-54)	42 (36-48)
	Fruit juice	0.1 (0.1-0.2)	0.2 (0.1-0.2)	5 (4-7)	10 (8-12)
Ghana	Suboptimal diet	61.6 (58.1-65.4)	64.1 (60-69)	1184 (1110-1263)	2127 (1966-2294)
	Whole grains	35.8 (32.7-37.9)	36.5 (33.4-38.7)	687 (626-734)	1209 (1106-1291)
	Refined grains (total)	11.9 (9.7-14.6)	14.8 (12.2-17.7)	228 (186-280)	489 (405-589)
	Refined rice	7.6 (6.2-9.4)	11.7 (9.6-14.1)	145 (118-182)	387 (318-470)
	Refined wheat	4.7 (3.8-5.8)	3.5 (2.8-4.5)	90 (72-113)	115 (94-149)
	Processed meats	1.4 (0.3-5.6)	2.9 (0.7-10.9)	26 (6-108)	97 (23-359)
	Unprocessed red meats	12.5 (9.8-16.1)	6.4 (4.5-9.2)	241 (187-309)	212 (147-305)
	Yogurt	12.7 (12-13.4)	13 (12.4-13.7)	244 (229-259)	433 (407-457)

Table S5. Proportional and absolute burden per 1M population of T2D incidence attributable to suboptimal diet* and each individual dietary factor† at the national level in 1990 and 2018

Country	Dietary factor	Proportional burden, %		Rate, per 1M population‡	
		1990	2018	1990	2018
	Sugar-sweetened beverages	18.5 (12.5-25.5)	26 (19.1-34.9)	356 (239-490)	862 (631-1161)
	Potatoes	0.1 (0.1-0.3)	0.2 (0.1-0.4)	3 (1-5)	6 (3-12)
	Fruit	2.7 (2.5-2.8)	3 (2.9-3.2)	51 (48-54)	100 (94-105)
	Nuts and seeds	0.7 (0.5-0.8)	0.6 (0.5-0.8)	13 (10-16)	21 (16-27)
	Non-starchy vegetables	1 (0.9-1)	1.2 (1.1-1.2)	18 (17-20)	38 (35-41)
	Fruit juice	0.1 (0.1-0.1)	0.1 (0-0.1)	1 (1-2)	2 (2-4)
Greece	Suboptimal diet	63.3 (59-67.9)	64.3 (59.9-68.8)	1663 (1536-1807)	2450 (2265-2637)
	Whole grains	27.8 (23.6-31.4)	24.8 (19.4-29.5)	731 (613-827)	948 (738-1115)
	Refined grains (total)	1.3 (0.8-2.4)	2.5 (1.5-4.4)	35 (21-63)	94 (56-170)
	Refined rice	0.1 (0.1-0.1)	0.2 (0.1-0.4)	2 (1-4)	9 (5-16)
	Refined wheat	1.2 (0.7-2.3)	2.2 (1.3-4)	33 (19-59)	85 (51-154)
	Processed meats	4.7 (3.2-8.5)	7.3 (4.8-12.2)	122 (84-221)	276 (181-466)
	Unprocessed red meats	28.9 (23.3-35.9)	32 (25.8-39.2)	760 (610-950)	1218 (979-1500)
	Yogurt	7.1 (5.1-8.7)	7.4 (5.5-9.2)	187 (133-231)	282 (209-351)
	Sugar-sweetened beverages	6.3 (5-9)	7.4 (5.9-10.3)	165 (130-237)	280 (225-393)
	Potatoes	10 (7.4-13.7)	6.7 (5-9.3)	264 (194-361)	257 (190-357)
	Fruit	1.8 (1.6-2.1)	3 (2.8-3.2)	48 (42-55)	115 (107-124)
	Nuts and seeds	1 (0.9-1.1)	1 (0.9-1.1)	26 (23-28)	39 (34-43)
	Non-starchy vegetables	1 (0.9-1.1)	1.5 (1.3-1.6)	26 (23-29)	55 (50-60)
	Fruit juice	0.1 (0-0.1)	0.1 (0.1-0.1)	2 (1-2)	3 (2-4)
Grenada	Suboptimal diet	76.9 (71.9-81.1)	65.4 (60.1-71.2)	3266 (3057-3472)	4642 (4272-5074)
	Whole grains	33 (26.8-36.8)	30.6 (22.8-35.8)	1400 (1149-1567)	2167 (1615-2544)
	Refined grains (total)	35.5 (27.8-42.9)	21.9 (15.9-29.9)	1507 (1170-1833)	1552 (1136-2124)
	Refined rice	15.3 (11.5-19.3)	5.9 (4.1-8.6)	652 (489-825)	420 (292-607)
	Refined wheat	24.8 (19-30.6)	17.3 (12.5-24.1)	1053 (806-1303)	1229 (888-1713)
	Processed meats	10.7 (4.9-20.6)	12.2 (5.7-23.9)	451 (211-883)	864 (402-1702)
	Unprocessed red meats	8.7 (5.8-12.6)	8.6 (5.6-12.7)	370 (247-539)	608 (398-897)
	Yogurt	11 (9.7-11.9)	11.6 (10.4-12.6)	467 (411-508)	825 (730-900)
	Sugar-sweetened beverages	36.3 (29.4-43.9)	19.6 (14.9-25.7)	1541 (1250-1872)	1387 (1051-1824)
	Potatoes	0.7 (0.4-1.3)	1.3 (0.7-2.5)	29 (16-55)	93 (50-183)
	Fruit	2 (1.4-2.6)	2.6 (1.9-3.2)	86 (59-108)	187 (138-226)
	Nuts and seeds	1.2 (1.2-1.3)	1.5 (1.4-1.6)	53 (49-56)	105 (98-112)
	Non-starchy vegetables	1.1 (0.8-1.4)	0.5 (0.2-0.9)	48 (36-58)	34 (13-64)
	Fruit juice	0.1 (0.1-0.2)	0.1 (0.1-0.2)	5 (3-7)	9 (5-15)
Guatemala	Suboptimal diet	66.1 (58.2-76.2)	85.5 (80.8-90.5)	1710 (1496-1969)	4969 (4661-5280)

Table S5. Proportional and absolute burden per 1M population of T2D incidence attributable to suboptimal diet* and each individual dietary factor† at the national level in 1990 and 2018

Country	Dietary factor	Proportional burden, %		Rate, per 1M population‡	
		1990	2018	1990	2018
	Whole grains	26.9 (19.1-33.6)	31.9 (23.6-37.6)	696 (492-865)	1853 (1364-2185)
	Refined grains (total)	12.2 (10.2-14.6)	28 (24.1-32.3)	316 (262-378)	1623 (1397-1883)
	Refined rice	2.3 (1.9-3.2)	6.5 (5.4-8.3)	59 (49-81)	376 (316-484)
	Refined wheat	10.1 (8.4-12.3)	23 (19.8-26.8)	262 (217-317)	1337 (1142-1567)
	Processed meats	32.8 (17.5-51.6)	44.3 (28.1-62.4)	849 (447-1333)	2574 (1604-3615)
	Unprocessed red meats	7 (5.3-9.9)	25.5 (21.7-29.7)	181 (136-257)	1471 (1261-1732)
	Yogurt	12 (11.3-12.8)	12.7 (11.9-13.6)	311 (289-333)	740 (688-798)
	Sugar-sweetened beverages	10.3 (7.7-13.6)	26.9 (21.7-33)	267 (197-352)	1558 (1259-1919)
	Potatoes	0.4 (0.2-0.7)	2.4 (1.2-4.6)	9 (5-18)	137 (71-271)
	Fruit	3 (2.9-3.2)	3.4 (3.2-3.6)	79 (74-83)	197 (184-210)
	Nuts and seeds	1.3 (1.2-1.4)	1.4 (1.2-1.5)	33 (31-36)	79 (70-87)
	Non-starchy vegetables	1.3 (1.2-1.4)	1.6 (1.5-1.7)	34 (32-37)	93 (86-101)
	Fruit juice	0.1 (0.1-0.1)	0.1 (0.1-0.1)	2 (2-3)	6 (5-8)
Guinea	Suboptimal diet	52.9 (42-69.7)	68 (61-76.9)	909 (725-1200)	1670 (1479-1882)
	Whole grains	23.3 (15.9-29.7)	26 (18.2-32.2)	399 (274-512)	635 (444-791)
	Refined grains (total)	9.7 (7.5-12.3)	9.9 (7.9-12.4)	165 (127-212)	244 (194-304)
	Refined rice	8.9 (6.9-11.4)	9.2 (7.3-11.6)	153 (117-194)	226 (180-284)
	Refined wheat	0.8 (0.6-1.1)	0.8 (0.6-1.1)	14 (11-18)	19 (15-25)
	Processed meats	22.5 (7.7-50)	18.9 (7.8-38.7)	383 (135-857)	463 (192-936)
	Unprocessed red meats	0.5 (0.3-2.2)	6.1 (4-9.8)	9 (5-38)	151 (96-239)
	Yogurt	11.5 (10.4-12.3)	12.3 (11.3-13.1)	196 (177-211)	301 (275-322)
	Sugar-sweetened beverages	4.7 (2.8-7.6)	8.8 (5.6-13)	80 (49-131)	214 (137-321)
	Potatoes	0.1 (0-0.1)	23.7 (15.5-33)	1 (1-2)	582 (379-808)
	Fruit	2.9 (2.7-3.1)	3.9 (3.7-4.1)	49 (46-52)	96 (91-102)
	Nuts and seeds	1.2 (1.1-1.3)	1 (0.8-1.2)	20 (18-22)	24 (20-29)
	Non-starchy vegetables	1.7 (1.6-1.8)	2.1 (2-2.2)	30 (28-32)	52 (49-55)
	Fruit juice	0 (0-0)	0 (0-0.1)	0 (0-1)	1 (1-1)
Guinea-Bissau	Suboptimal diet	54.8 (48.8-62.6)	76.9 (72-81.7)	1057 (939-1209)	1956 (1814-2099)
	Whole grains	27.7 (20.3-33.7)	34.6 (28.4-38.7)	533 (392-653)	882 (720-989)
	Refined grains (total)	21.9 (16.7-28.1)	20 (15.1-25.7)	421 (321-541)	508 (384-655)
	Refined rice	21.6 (16.4-27.7)	18.7 (14.1-24.2)	416 (317-534)	475 (358-615)
	Refined wheat	0.4 (0.3-0.5)	1.7 (1.2-2.5)	7 (6-10)	43 (31-64)
	Processed meats	7 (1.9-21.4)	6.4 (1.9-17.7)	135 (37-411)	163 (50-450)
	Unprocessed red meats	10.9 (7.7-15.3)	10.8 (7.8-15.1)	211 (151-294)	276 (199-381)
	Yogurt	11.7 (10.6-12.6)	12.3 (11.3-13.3)	225 (204-246)	313 (284-341)

Table S5. Proportional and absolute burden per 1M population of T2D incidence attributable to suboptimal diet* and each individual dietary factor† at the national level in 1990 and 2018

Country	Dietary factor	Proportional burden, %		Rate, per 1M population‡	
		1990	2018	1990	2018
	Sugar-sweetened beverages	0.2 (0.1-0.4)	26.9 (18.6-35.4)	3 (2-9)	683 (473-901)
	Potatoes	1.3 (0.7-2.6)	28.8 (19.2-37.3)	25 (13-51)	731 (485-954)
	Fruit	2.8 (2.6-3)	3.3 (3.1-3.5)	54 (50-58)	84 (78-90)
	Nuts and seeds	0.8 (0.6-1)	1 (0.8-1.2)	15 (12-18)	25 (20-30)
	Non-starchy vegetables	1.1 (0.9-1.2)	1.2 (1-1.3)	21 (18-24)	29 (25-34)
	Fruit juice	0.1 (0-0.1)	0.1 (0.1-0.2)	1 (1-2)	3 (2-4)
Guyana	Suboptimal diet	92.6 (86.6-96.1)	89.1 (83.6-93.2)	4459 (4150-4679)	7115 (6643-7515)
	Whole grains	42.7 (41.5-43.9)	40.4 (36.9-42.4)	2058 (1971-2141)	3220 (2908-3418)
	Refined grains (total)	23.5 (19.6-28.3)	20.8 (17.3-25.1)	1134 (944-1356)	1664 (1376-2025)
	Refined rice	18.6 (15.4-22.7)	15.4 (12.6-18.9)	896 (742-1084)	1230 (1007-1517)
	Refined wheat	6.1 (4.9-7.7)	6.4 (5.2-8.2)	293 (238-374)	512 (412-652)
	Processed meats	69.1 (46.8-83.1)	61.9 (44.4-76.4)	3330 (2246-4029)	4937 (3540-6116)
	Unprocessed red meats	18.8 (15.2-23.1)	18.8 (15.1-23.9)	906 (732-1108)	1499 (1202-1915)
	Yogurt	12.6 (11.6-13.5)	12.3 (11.1-13.3)	609 (556-658)	986 (887-1069)
	Sugar-sweetened beverages	38.6 (27.5-50.5)	28.9 (19.8-39.6)	1863 (1306-2440)	2299 (1577-3158)
	Potatoes	0 (0-0)	2.7 (1.5-5.1)	0 (0-0)	218 (117-403)
	Fruit	2.6 (2.4-2.8)	1.8 (1.4-2.1)	125 (114-136)	142 (114-171)
	Nuts and seeds	1.3 (1.1-1.4)	1.1 (0.9-1.3)	61 (55-65)	90 (76-103)
	Non-starchy vegetables	1.4 (1.3-1.6)	1.2 (1-1.4)	69 (63-75)	95 (80-109)
	Fruit juice	0.1 (0.1-0.2)	0.1 (0.1-0.2)	7 (4-12)	10 (6-17)
Haiti	Suboptimal diet	64 (58.2-72.2)	69.5 (63.2-77.1)	2373 (2144-2700)	4258 (3825-4721)
	Whole grains	31.9 (24.3-37.1)	30.6 (22.1-36.9)	1180 (899-1377)	1867 (1340-2267)
	Refined grains (total)	20.1 (17.2-23.7)	20.5 (17.4-24.3)	742 (632-877)	1256 (1060-1488)
	Refined rice	13.9 (11.8-16.7)	17 (14.4-20.3)	514 (434-621)	1039 (876-1244)
	Refined wheat	7.2 (6.1-9)	4.2 (3.6-5.4)	265 (225-336)	259 (217-330)
	Processed meats	18.7 (7.8-36.6)	23.8 (10.6-41)	693 (284-1346)	1456 (640-2528)
	Unprocessed red meats	5.1 (3.5-9)	5.8 (4-10.4)	189 (130-327)	353 (245-640)
	Yogurt	13.6 (12.8-14.4)	14 (13.1-14.8)	504 (470-539)	855 (794-923)
	Sugar-sweetened beverages	12.6 (8.5-18.8)	13.5 (9-20.3)	465 (315-691)	828 (550-1239)
	Potatoes	0.6 (0.3-1.3)	6.9 (3.4-12.9)	24 (11-50)	420 (209-795)
	Fruit	3 (2.8-3.2)	3.5 (3.3-3.7)	111 (104-119)	213 (198-229)
	Nuts and seeds	1 (0.8-1.1)	1.2 (1-1.3)	36 (30-41)	72 (63-81)
	Non-starchy vegetables	1.4 (1.3-1.5)	1.7 (1.6-1.8)	53 (48-57)	105 (95-114)
	Fruit juice	0.1 (0.1-0.1)	0.1 (0.1-0.2)	3 (3-4)	8 (6-11)
Honduras	Suboptimal diet	73.8 (67.2-81.7)	87.2 (82-91.7)	2410 (2177-2677)	4250 (3949-4522)

Table S5. Proportional and absolute burden per 1M population of T2D incidence attributable to suboptimal diet* and each individual dietary factor† at the national level in 1990 and 2018

Country	Dietary factor	Proportional burden, %		Rate, per 1M population‡	
		1990	2018	1990	2018
	Whole grains	27.5 (19.2-33.7)	31.4 (23.3-37.1)	897 (629-1108)	1526 (1131-1814)
	Refined grains (total)	21.1 (17.7-24.8)	21.5 (18.3-25.3)	691 (580-818)	1045 (888-1229)
	Refined rice	7.4 (6.1-9.1)	10 (8.3-11.9)	244 (199-300)	484 (404-578)
	Refined wheat	14.8 (12.3-17.6)	12.9 (10.8-15.3)	485 (403-578)	626 (525-745)
	Processed meats	36.7 (21.4-54.2)	55.3 (37.6-70.7)	1200 (707-1773)	2696 (1827-3461)
	Unprocessed red meats	4.7 (3.2-7.2)	13.2 (10.4-16.7)	153 (105-237)	642 (501-817)
	Yogurt	13.3 (12.7-13.9)	13.9 (13.2-14.6)	435 (410-460)	675 (636-717)
	Sugar-sweetened beverages	20.1 (15.7-25.5)	34.8 (28-41.7)	655 (515-836)	1693 (1362-2046)
	Potatoes	0.8 (0.4-1.6)	3.6 (1.8-6.9)	26 (13-52)	174 (87-335)
	Fruit	2.8 (2.6-2.9)	3.2 (3-3.5)	90 (84-96)	158 (146-169)
	Nuts and seeds	1.2 (1.1-1.3)	1.2 (1.1-1.3)	39 (36-42)	60 (53-66)
	Non-starchy vegetables	1.8 (1.8-1.9)	2.2 (2.1-2.3)	60 (57-63)	107 (101-113)
	Fruit juice	0.1 (0.1-0.2)	0.2 (0.1-0.2)	5 (4-6)	8 (6-10)
Hungary	Suboptimal diet	61.7 (55.9-67.8)	51.9 (47.4-57.1)	2130 (1922-2364)	2578 (2337-2868)
	Whole grains	17.2 (13.3-21.1)	21.3 (17.2-24.8)	593 (459-725)	1059 (853-1235)
	Refined grains (total)	3.6 (2.1-6.1)	5.5 (3.2-9)	123 (72-210)	272 (157-449)
	Refined rice	0.2 (0.1-0.3)	0.4 (0.2-0.7)	6 (3-9)	19 (11-33)
	Refined wheat	3.4 (2-5.9)	5.1 (2.9-8.4)	118 (69-201)	253 (145-419)
	Processed meats	31.3 (22.3-41.7)	18.3 (12.4-26.1)	1085 (776-1440)	908 (615-1292)
	Unprocessed red meats	12.8 (9.1-18.6)	7.3 (4.9-11.1)	443 (320-637)	362 (245-555)
	Yogurt	6.5 (4.2-8.3)	6.4 (4.1-8.4)	224 (145-289)	316 (203-415)
	Sugar-sweetened beverages	7.9 (6.2-10.9)	6.3 (4.9-9)	272 (213-376)	316 (244-450)
	Potatoes	4.7 (3.8-6.7)	1.5 (1.2-2)	162 (132-234)	75 (62-99)
	Fruit	2.7 (2.5-2.8)	3.2 (3.1-3.4)	93 (86-99)	160 (151-170)
	Nuts and seeds	1.2 (1.1-1.2)	1.3 (1.2-1.4)	41 (38-43)	65 (61-70)
	Non-starchy vegetables	1.4 (1.3-1.4)	1.6 (1.6-1.7)	47 (44-50)	82 (77-87)
	Fruit juice	0 (0-0.1)	0.1 (0-0.2)	2 (1-5)	3 (1-9)
Iceland	Suboptimal diet	71.4 (67.2-76.1)	69.4 (64.2-74.8)	1271 (1173-1376)	2564 (2356-2782)
	Whole grains	10.4 (7.4-13.4)	11.9 (8.7-15.2)	185 (131-238)	437 (323-560)
	Refined grains (total)	5.2 (3.5-7.8)	10.8 (7.5-15.2)	93 (63-139)	402 (277-568)
	Refined rice	0.3 (0.2-0.4)	1.1 (0.7-1.6)	5 (4-8)	39 (27-59)
	Refined wheat	4.9 (3.3-7.4)	9.9 (6.8-14)	88 (60-132)	367 (253-521)
	Processed meats	41.2 (34.1-49.7)	32.8 (23.7-43.8)	734 (606-890)	1213 (882-1611)
	Unprocessed red meats	22.6 (19.1-27.4)	25.6 (22-31.6)	402 (339-491)	947 (802-1175)
	Yogurt	8.1 (6-9.7)	7.5 (5.4-9.3)	143 (108-175)	276 (202-347)

Table S5. Proportional and absolute burden per 1M population of T2D incidence attributable to suboptimal diet* and each individual dietary factor† at the national level in 1990 and 2018

Country	Dietary factor	Proportional burden, %		Rate, per 1M population‡	
		1990	2018	1990	2018
	Sugar-sweetened beverages	11.1 (9-14.9)	12.8 (10.3-17.2)	199 (158-268)	475 (380-638)
	Potatoes	7.8 (5.3-11.7)	2.2 (1.5-3.8)	138 (93-208)	83 (54-143)
	Fruit	2.6 (2.4-2.7)	2.9 (2.7-3.2)	46 (42-50)	109 (100-118)
	Nuts and seeds	1.2 (1.1-1.3)	1.3 (1.2-1.4)	22 (20-23)	48 (44-52)
	Non-starchy vegetables	1.3 (1.2-1.4)	1.6 (1.5-1.7)	24 (22-26)	58 (53-64)
	Fruit juice	0.2 (0.2-0.2)	0.2 (0.2-0.3)	3 (3-4)	7 (6-9)
India	Suboptimal diet	52.4 (48.3-60.2)	50.2 (46.5-56.9)	1359 (1243-1562)	2156 (1979-2430)
	Whole grains	6.6 (4.2-9.8)	6.7 (4.5-10.1)	171 (109-257)	288 (188-437)
	Refined grains (total)	32.7 (28.5-37.6)	29.2 (25.4-33.8)	847 (735-970)	1254 (1088-1449)
	Refined rice	26.8 (23.2-31)	20.8 (17.9-24.6)	696 (599-804)	891 (765-1053)
	Refined wheat	8 (6.8-9.7)	10.7 (9.1-12.9)	208 (176-252)	459 (390-554)
	Processed meats	5 (1-19.5)	3.8 (0.9-15.3)	129 (25-498)	164 (36-648)
	Unprocessed red meats	0.8 (0.5-3)	0.7 (0.4-2.7)	21 (13-77)	32 (19-116)
	Yogurt	13.2 (12.3-14)	13.2 (12.3-14)	342 (317-363)	564 (526-605)
	Sugar-sweetened beverages	0.7 (0.4-1.9)	0.8 (0.5-2)	19 (11-50)	35 (21-84)
	Potatoes	3.3 (2.7-4.9)	4.1 (3.4-5.8)	86 (71-126)	178 (147-248)
	Fruit	3.5 (3.4-3.7)	4 (3.8-4.2)	92 (87-97)	171 (162-181)
	Nuts and seeds	1.2 (1.1-1.3)	1.3 (1.2-1.4)	31 (29-34)	56 (52-62)
	Non-starchy vegetables	0.8 (0.8-0.9)	1 (0.9-1.1)	22 (20-24)	42 (38-47)
	Fruit juice	0 (0-0)	0 (0-0)	0 (0-0)	0 (0-0)
Indonesia	Suboptimal diet	61.4 (57-68.3)	66 (59.6-75)	1265 (1167-1412)	2275 (2049-2589)
	Whole grains	33.3 (27.3-37.6)	29.7 (22.6-35.1)	685 (562-776)	1024 (779-1211)
	Refined grains (total)	24 (21.2-27.2)	22.4 (19.8-25.2)	494 (435-560)	771 (678-876)
	Refined rice	23.3 (20.6-26.4)	20.2 (17.8-22.9)	479 (422-544)	696 (611-793)
	Refined wheat	0.9 (0.8-1.1)	2.7 (2.4-3.6)	19 (16-23)	94 (82-124)
	Processed meats	13.9 (6.1-28.3)	25.7 (12.1-45.4)	287 (125-584)	887 (414-1559)
	Unprocessed red meats	9.4 (7.3-12.3)	9 (7-12.2)	193 (150-253)	311 (242-422)
	Yogurt	13.4 (12.8-14)	13.4 (12.7-14.1)	276 (260-292)	462 (435-489)
	Sugar-sweetened beverages	1.6 (1.1-3.1)	3.2 (2.3-5.3)	33 (23-63)	111 (78-182)
	Potatoes	1.8 (1.5-2.4)	2.3 (1.9-3.1)	36 (30-49)	78 (65-107)
	Fruit	3.1 (2.9-3.2)	3.4 (3.3-3.6)	63 (60-66)	117 (112-123)
	Nuts and seeds	1.1 (1-1.2)	1.2 (1.1-1.2)	22 (21-24)	40 (37-43)
	Non-starchy vegetables	0.9 (0.8-1)	1 (0.9-1.1)	18 (16-20)	35 (31-39)
	Fruit juice	0 (0-0)	0 (0-0)	1 (1-1)	1 (1-1)
Iran	Suboptimal diet	69 (66.4-71.8)	62.4 (58.9-66)	1638 (1557-1724)	3057 (2852-3282)

Table S5. Proportional and absolute burden per 1M population of T2D incidence attributable to suboptimal diet* and each individual dietary factor† at the national level in 1990 and 2018

Country	Dietary factor	Proportional burden, %		Rate, per 1M population‡	
		1990	2018	1990	2018
	Whole grains	40.1 (38.9-41.1)	16.8 (14.2-19.6)	951 (913-988)	824 (693-967)
	Refined grains (total)	39.3 (35.5-43.4)	39.1 (35.2-43.6)	934 (833-1033)	1917 (1719-2137)
	Refined rice	12.6 (11-14.7)	13.5 (11.9-15.8)	299 (259-348)	662 (578-778)
	Refined wheat	30.9 (27.6-34.4)	29.8 (26.6-33.4)	733 (649-816)	1461 (1297-1648)
	Processed meats	7.5 (5.1-11.1)	9.7 (6-15.7)	177 (119-262)	476 (294-774)
	Unprocessed red meats	12.3 (9.9-15.6)	5 (3.5-8.4)	291 (235-371)	244 (174-409)
	Yogurt	5.7 (4.8-6.5)	6.1 (5.1-7)	134 (114-156)	297 (251-348)
	Sugar-sweetened beverages	10.3 (8.5-13.1)	9.9 (8.3-13.8)	245 (202-314)	487 (407-674)
	Potatoes	1.1 (1-1.4)	2.2 (1.9-2.7)	27 (23-34)	106 (95-135)
	Fruit	1.8 (1.6-1.9)	1.9 (1.8-2.1)	42 (38-46)	95 (86-105)
	Nuts and seeds	1.2 (1.1-1.3)	1.1 (1-1.2)	28 (26-30)	54 (49-59)
	Non-starchy vegetables	0.8 (0.7-0.9)	0.5 (0.4-0.6)	19 (17-21)	25 (20-30)
	Fruit juice	0 (0-0)	0 (0-0)	1 (1-1)	1 (1-2)
Iraq	Suboptimal diet	95 (91-97.4)	83.9 (76.6-90.2)	3681 (3478-3864)	5720 (5180-6218)
	Whole grains	16.2 (8-25.6)	38.1 (31.7-41.8)	628 (315-1004)	2595 (2157-2865)
	Refined grains (total)	31.1 (20.7-43.3)	31.8 (21.5-43.1)	1212 (800-1682)	2169 (1432-2936)
	Refined rice	10.6 (6.6-15.9)	13.1 (8.3-18.6)	412 (257-619)	892 (562-1275)
	Refined wheat	23.8 (15.5-34.1)	22.5 (14.8-31.3)	928 (605-1326)	1537 (989-2144)
	Processed meats	70.4 (53.1-82.9)	42.4 (23.9-64.3)	2727 (2046-3237)	2874 (1631-4320)
	Unprocessed red meats	60.6 (49.5-68.8)	22.1 (14.8-30.7)	2347 (1913-2705)	1507 (1002-2092)
	Yogurt	8.2 (5.5-10.5)	8.9 (6.2-11.2)	319 (216-411)	607 (424-762)
	Sugar-sweetened beverages	21.7 (14.8-30.9)	19.4 (12.9-27.8)	844 (569-1203)	1322 (875-1907)
	Potatoes	0.8 (0.4-1.5)	3.3 (1.9-6.5)	30 (17-55)	227 (131-443)
	Fruit	2.4 (1.8-2.9)	2.8 (2.1-3.4)	93 (69-113)	191 (140-231)
	Nuts and seeds	0.6 (0.4-0.7)	1 (0.8-1.2)	22 (16-29)	68 (54-82)
	Non-starchy vegetables	0.6 (0.3-1)	1.2 (0.9-1.6)	24 (12-38)	85 (58-108)
	Fruit juice	0.1 (0.1-0.2)	0.2 (0.1-0.3)	5 (3-9)	11 (6-20)
Ireland	Suboptimal diet	79 (73.3-85.4)	76.7 (70.4-83.9)	1103 (1013-1198)	2866 (2621-3155)
	Whole grains	27.2 (23.1-30.9)	30.5 (27.1-33.5)	381 (319-432)	1142 (1011-1259)
	Refined grains (total)	2.2 (1.3-3.6)	6.1 (3.8-10.1)	30 (18-51)	230 (143-373)
	Refined rice	0.1 (0-0.1)	0.4 (0.2-0.7)	1 (1-1)	15 (9-25)
	Refined wheat	2.1 (1.3-3.6)	5.8 (3.6-9.5)	29 (18-50)	215 (135-350)
	Processed meats	44.6 (31.4-60.9)	43.5 (30.1-59.6)	625 (437-845)	1629 (1141-2246)
	Unprocessed red meats	16.9 (10.7-25.3)	15.6 (9.8-23.7)	235 (149-355)	583 (364-893)
	Yogurt	8.1 (6.3-9.6)	7.8 (5.8-9.4)	113 (87-135)	291 (217-354)

Table S5. Proportional and absolute burden per 1M population of T2D incidence attributable to suboptimal diet* and each individual dietary factor† at the national level in 1990 and 2018

Country	Dietary factor	Proportional burden, %		Rate, per 1M population‡	
		1990	2018	1990	2018
	Sugar-sweetened beverages	12.1 (9.6-15.2)	10.8 (8.7-14.1)	169 (132-214)	403 (327-525)
	Potatoes	18.2 (14-23.7)	9.3 (7-12.8)	254 (197-333)	347 (260-476)
	Fruit	2.5 (2.3-2.7)	2.9 (2.6-3.1)	35 (32-38)	108 (98-117)
	Nuts and seeds	1.1 (1-1.2)	0.8 (0.6-1)	16 (14-17)	30 (22-39)
	Non-starchy vegetables	1 (0.9-1.1)	1.2 (1-1.3)	14 (12-16)	44 (38-50)
	Fruit juice	0.1 (0.1-0.3)	0.1 (0.1-0.3)	2 (1-4)	5 (3-9)
Israel	Suboptimal diet	93.1 (91.1-94.9)	89.8 (86.6-92.5)	2656 (2529-2770)	4095 (3911-4276)
	Whole grains	12.9 (10-16)	13.8 (10.8-17.1)	371 (282-462)	632 (495-777)
	Refined grains (total)	19 (16.2-22.3)	19.4 (16.7-23.2)	542 (464-640)	885 (760-1063)
	Refined rice	2.2 (1.9-3)	5.2 (4.5-6.6)	64 (54-85)	239 (204-306)
	Refined wheat	17.1 (14.6-20.3)	14.9 (12.8-18.1)	489 (418-580)	681 (583-827)
	Processed meats	78.4 (72.6-83.5)	67 (58.2-75.2)	2231 (2047-2411)	3061 (2660-3451)
	Unprocessed red meats	39.4 (34.6-45.2)	42.8 (37.5-48.7)	1126 (976-1291)	1952 (1712-2230)
	Yogurt	2.1 (1.4-3)	2 (1.3-3)	60 (38-85)	93 (59-135)
	Sugar-sweetened beverages	19.2 (16.1-23.8)	13.2 (11-17.5)	547 (454-681)	603 (500-803)
	Potatoes	9 (7.8-11.9)	9.4 (8.1-12.5)	255 (222-336)	430 (372-576)
	Fruit	1.2 (1-1.5)	2.1 (1.9-2.3)	36 (29-42)	97 (86-107)
	Nuts and seeds	0.6 (0.5-0.7)	0.6 (0.5-0.7)	17 (14-20)	26 (21-31)
	Non-starchy vegetables	1 (0.9-1.1)	1.4 (1.3-1.5)	27 (24-30)	65 (59-70)
	Fruit juice	0.1 (0.1-0.1)	0.1 (0.1-0.2)	3 (2-3)	5 (4-7)
Italy	Suboptimal diet	74.7 (72-77.7)	74.7 (72-77.9)	2773 (2632-2933)	3976 (3774-4214)
	Whole grains	35.2 (33.7-36.5)	34.8 (33.3-36.3)	1307 (1239-1374)	1853 (1747-1959)
	Refined grains (total)	31.9 (28.1-35.3)	32.1 (28.3-35.3)	1183 (1045-1324)	1711 (1490-1902)
	Refined rice	2.4 (2.1-3.1)	3.5 (3-4.4)	89 (77-115)	186 (160-236)
	Refined wheat	30.2 (26.7-33.5)	29.6 (26.1-32.7)	1122 (989-1258)	1580 (1373-1762)
	Processed meats	27.2 (22-35)	26.5 (21.6-34.5)	1010 (811-1289)	1413 (1147-1854)
	Unprocessed red meats	20.4 (17.5-24.7)	21.1 (18.2-25.9)	757 (647-915)	1122 (962-1376)
	Yogurt	10.5 (9.7-11.3)	10.4 (9.7-11.3)	389 (359-423)	556 (510-605)
	Sugar-sweetened beverages	5 (4.2-7.7)	5 (4.3-8)	186 (155-287)	270 (227-421)
	Potatoes	3.2 (2.8-4.2)	2.6 (2.3-3.3)	119 (102-158)	140 (119-178)
	Fruit	1.3 (1.2-1.5)	1.4 (1.2-1.6)	50 (43-56)	75 (65-86)
	Nuts and seeds	1.2 (1.1-1.3)	1.1 (1.1-1.2)	45 (41-48)	61 (56-67)
	Non-starchy vegetables	0.8 (0.7-0.9)	0.9 (0.8-1.1)	31 (28-35)	50 (44-57)
	Fruit juice	0.1 (0-0.1)	0.1 (0-0.1)	2 (2-2)	3 (3-4)
Jamaica	Suboptimal diet	67.9 (63.5-72.1)	69.5 (64.9-74.4)	2503 (2332-2677)	4479 (4147-4831)

Table S5. Proportional and absolute burden per 1M population of T2D incidence attributable to suboptimal diet* and each individual dietary factor† at the national level in 1990 and 2018

Country	Dietary factor	Proportional burden, %		Rate, per 1M population‡	
		1990	2018	1990	2018
	Whole grains	27.6 (23.2-31.3)	29.2 (24.5-32.9)	1023 (854-1162)	1876 (1574-2125)
	Refined grains (total)	21.2 (15.5-28.4)	20.5 (15-27.5)	783 (574-1048)	1319 (963-1761)
	Refined rice	10.9 (7.8-14.9)	9.1 (6.4-12.8)	403 (288-552)	588 (415-826)
	Refined wheat	12.1 (8.6-16.5)	12.9 (9.2-17.7)	448 (319-612)	832 (594-1148)
	Processed meats	12.9 (8.9-18.1)	18.7 (12.6-27.1)	478 (328-675)	1204 (804-1753)
	Unprocessed red meats	6.1 (3.9-9.3)	3.6 (2.1-6.3)	224 (146-345)	231 (137-402)
	Yogurt	11 (9.8-11.9)	11.7 (10.5-12.5)	408 (360-443)	751 (670-813)
	Sugar-sweetened beverages	30.5 (24.7-37.5)	29.6 (23.3-36.2)	1129 (909-1388)	1911 (1499-2347)
	Potatoes	0.7 (0.4-1.3)	1.6 (0.8-3.1)	25 (13-50)	104 (54-200)
	Fruit	1.7 (1.3-2.1)	2 (1.4-2.4)	63 (47-77)	126 (94-157)
	Nuts and seeds	1.2 (1.2-1.3)	1.2 (1.1-1.3)	45 (43-48)	80 (73-87)
	Non-starchy vegetables	0.8 (0.6-1)	1.1 (0.9-1.3)	31 (24-38)	71 (56-84)
	Fruit juice	0.1 (0.1-0.1)	0.1 (0.1-0.2)	4 (2-5)	8 (5-11)
Japan	Suboptimal diet	69.1 (66.5-72.3)	72.3 (70-75.2)	1892 (1802-1998)	2025 (1925-2137)
	Whole grains	38.3 (36.5-39.8)	36.4 (34.8-37.9)	1048 (998-1104)	1018 (962-1075)
	Refined grains (total)	33.3 (29.4-37.9)	30 (26.5-34.1)	912 (805-1037)	839 (732-964)
	Refined rice	26.2 (22.9-30.1)	22.6 (19.8-26.2)	717 (626-827)	633 (548-739)
	Refined wheat	9.8 (8.4-12)	9.6 (8.3-11.5)	267 (229-328)	267 (230-321)
	Processed meats	12.8 (9.7-18.1)	12.1 (8.9-17.6)	349 (264-498)	338 (250-499)
	Unprocessed red meats	14.6 (12.5-18.9)	30.9 (28-34.8)	399 (341-518)	863 (778-982)
	Yogurt	8.6 (7.3-9.7)	7.5 (6.4-8.6)	234 (201-267)	211 (178-243)
	Sugar-sweetened beverages	8.4 (6.8-11.7)	6.7 (5.4-9.9)	230 (185-321)	186 (151-273)
	Potatoes	1.8 (1.5-2.4)	1.5 (1.2-1.9)	50 (41-64)	41 (34-52)
	Fruit	2.6 (2.5-2.7)	2.7 (2.6-2.9)	72 (67-76)	76 (71-82)
	Nuts and seeds	1.2 (1.1-1.3)	1.2 (1.1-1.3)	33 (30-35)	34 (31-37)
	Non-starchy vegetables	0.3 (0.3-0.4)	0.4 (0.3-0.4)	9 (8-11)	10 (8-12)
	Fruit juice	0.1 (0-0.1)	0.1 (0.1-0.1)	2 (1-2)	2 (1-2)
Jordan	Suboptimal diet	75.8 (70.9-81.4)	83.1 (76.2-89.5)	2889 (2682-3133)	5177 (4692-5657)
	Whole grains	37.3 (31.7-40.4)	30.6 (21.1-37.1)	1419 (1208-1554)	1901 (1340-2330)
	Refined grains (total)	29.6 (25.2-34.5)	30.6 (25.7-35.6)	1128 (952-1325)	1908 (1596-2229)
	Refined rice	8.1 (6.7-10)	7.1 (5.7-9)	306 (253-381)	442 (358-559)
	Refined wheat	23.5 (19.9-27.6)	25.4 (21.2-29.8)	897 (753-1058)	1581 (1315-1862)
	Processed meats	18.1 (8.2-35.8)	42.1 (21.9-63.9)	688 (313-1363)	2630 (1370-3958)
	Unprocessed red meats	20.4 (16.8-25)	21.3 (17.2-27)	778 (636-965)	1321 (1073-1689)
	Yogurt	9.7 (8.2-10.8)	9.6 (8-10.9)	368 (313-415)	596 (494-687)

Table S5. Proportional and absolute burden per 1M population of T2D incidence attributable to suboptimal diet* and each individual dietary factor† at the national level in 1990 and 2018

Country	Dietary factor	Proportional burden, %		Rate, per 1M population‡	
		1990	2018	1990	2018
	Sugar-sweetened beverages	20.7 (15.6-26.3)	24.1 (18-30.8)	789 (597-1002)	1499 (1124-1898)
	Potatoes	3.1 (1.7-5.7)	1.9 (1-3.5)	117 (64-215)	117 (65-221)
	Fruit	1.6 (1.4-1.8)	1.9 (1.6-2.2)	61 (53-71)	116 (99-135)
	Nuts and seeds	0.9 (0.8-1.1)	0.9 (0.8-1.1)	36 (31-41)	59 (48-69)
	Non-starchy vegetables	1.1 (0.9-1.2)	1.3 (1.1-1.4)	40 (36-45)	78 (69-88)
	Fruit juice	0.2 (0.1-0.2)	0.2 (0.2-0.3)	6 (5-8)	13 (10-17)
Kazakhstan	Suboptimal diet	93.1 (90.7-95.3)	90.9 (87.3-94.1)	2206 (2104-2312)	4404 (4175-4630)
	Whole grains	13.8 (8-21.2)	28.1 (21.1-34.3)	327 (188-502)	1359 (1016-1662)
	Refined grains (total)	64.7 (62.8-67)	47.7 (39.4-57.1)	1534 (1454-1617)	2313 (1897-2781)
	Refined rice	18.8 (17.8-20.8)	8.4 (6.5-11.2)	446 (416-499)	407 (312-542)
	Refined wheat	56.9 (55-59.2)	43 (35.2-52.1)	1350 (1276-1428)	2086 (1695-2540)
	Processed meats	45.6 (30.4-61.6)	49.7 (35.1-64)	1082 (721-1460)	2397 (1676-3105)
	Unprocessed red meats	47.6 (40.6-55.7)	41.8 (34.1-49.9)	1127 (951-1330)	2023 (1648-2434)
	Yogurt	8 (5.5-10)	7.8 (5.2-9.9)	190 (131-240)	375 (248-481)
	Sugar-sweetened beverages	3.1 (2-5.5)	5.3 (3.6-8.6)	74 (47-130)	256 (173-413)
	Potatoes	10.4 (6.6-16.7)	14.6 (9.2-22)	247 (153-400)	705 (442-1073)
	Fruit	3.3 (3.1-3.4)	3.6 (3.3-3.8)	77 (72-82)	172 (160-185)
	Nuts and seeds	1.4 (1.3-1.5)	0.5 (0.4-0.7)	33 (30-36)	27 (20-34)
	Non-starchy vegetables	1.1 (1-1.3)	0.8 (0.7-1)	27 (24-30)	40 (32-48)
	Fruit juice	0.1 (0-0.1)	0 (0-0.1)	1 (1-2)	2 (1-3)
Kenya	Suboptimal diet	53.7 (50.7-56.8)	54 (51.4-56.8)	748 (703-798)	991 (935-1056)
	Whole grains	34.7 (31.8-36.9)	36.7 (33.8-39)	484 (440-516)	674 (614-723)
	Refined grains (total)	18.8 (15.5-23.1)	18.8 (15.9-22.2)	263 (215-322)	344 (292-408)
	Refined rice	2.8 (2.3-3.7)	7.7 (6.4-9.6)	39 (31-52)	141 (118-178)
	Refined wheat	16.5 (13.5-20.3)	12 (10.1-14.5)	230 (189-283)	221 (186-267)
	Processed meats	0.9 (0.4-2.6)	0.9 (0.4-2.5)	12 (6-36)	16 (8-48)
	Unprocessed red meats	6.4 (4.3-9.3)	6.2 (4.2-9.7)	90 (59-130)	113 (78-179)
	Yogurt	12.1 (11.1-12.8)	12.9 (12-13.7)	168 (154-180)	236 (218-254)
	Sugar-sweetened beverages	6.5 (3.5-11.2)	4 (2.1-7.6)	90 (49-159)	73 (39-140)
	Potatoes	1.6 (1.1-2.5)	2.7 (1.8-4.2)	22 (16-35)	49 (34-77)
	Fruit	2.4 (2.2-2.5)	2.8 (2.6-3)	33 (31-36)	52 (48-56)
	Nuts and seeds	1.3 (1.2-1.4)	1.5 (1.4-1.6)	18 (17-20)	27 (25-29)
	Non-starchy vegetables	1.5 (1.4-1.6)	0.4 (0.3-0.5)	21 (20-23)	8 (6-10)
	Fruit juice	0.1 (0-0.1)	0.1 (0.1-0.1)	1 (1-1)	2 (1-3)
Kiribati	Suboptimal diet	70.3 (63.9-77.3)	77.7 (70.1-84.8)	3993 (3602-4392)	7179 (6481-7840)

Table S5. Proportional and absolute burden per 1M population of T2D incidence attributable to suboptimal diet* and each individual dietary factor† at the national level in 1990 and 2018

Country	Dietary factor	Proportional burden, %		Rate, per 1M population‡	
		1990	2018	1990	2018
	Whole grains	39.6 (34.1-42.6)	30.6 (22.2-37)	2249 (1932-2436)	2812 (2034-3420)
	Refined grains (total)	35.6 (24.6-46.9)	39.3 (27.3-51.2)	2017 (1391-2655)	3624 (2527-4726)
	Refined rice	26.7 (17.9-36.2)	29.3 (19.7-39.6)	1513 (1019-2043)	2708 (1823-3631)
	Refined wheat	13.7 (8.8-19.3)	15.7 (10.1-22)	779 (495-1103)	1458 (939-2031)
	Processed meats	15 (6.6-28.7)	26.5 (12.5-45.9)	853 (372-1611)	2446 (1162-4210)
	Unprocessed red meats	10.1 (6.6-14.9)	16.9 (11.8-22.7)	574 (377-841)	1567 (1084-2092)
	Yogurt	13.8 (12.8-14.7)	14 (13-14.9)	785 (723-837)	1293 (1183-1383)
	Sugar-sweetened beverages	4.8 (2.8-8.5)	9.4 (5.8-14.6)	272 (160-482)	872 (530-1371)
	Potatoes	0.2 (0.1-0.4)	0.1 (0-0.2)	12 (6-24)	9 (4-18)
	Fruit	3.3 (2.8-3.6)	3.3 (2.7-3.8)	185 (158-206)	304 (247-355)
	Nuts and seeds	1.2 (1.1-1.4)	1.4 (1.3-1.6)	71 (62-79)	130 (116-144)
	Non-starchy vegetables	1.3 (1-1.6)	1.7 (1.4-2)	76 (55-94)	159 (127-184)
	Fruit juice	0 (0-0.1)	0 (0-0.1)	3 (2-5)	5 (3-8)
Kuwait	Suboptimal diet	64.6 (59-72.3)	88 (81-93.6)	2282 (2055-2579)	6102 (5579-6702)
	Whole grains	43.4 (40.6-45.5)	34.5 (25.8-40)	1532 (1403-1646)	2390 (1789-2826)
	Refined grains (total)	22.9 (14.4-34)	25.4 (16.2-37.5)	809 (509-1215)	1772 (1123-2640)
	Refined rice	13 (8-20.6)	13.5 (8.3-21.4)	459 (279-722)	946 (576-1491)
	Refined wheat	12 (7.3-19)	14.3 (8.8-22.4)	423 (256-669)	994 (606-1565)
	Processed meats	8.9 (2.3-25.1)	42.1 (18.8-66.8)	312 (81-900)	2928 (1310-4706)
	Unprocessed red meats	3.2 (1.9-8.5)	9.9 (6.9-16.2)	115 (66-299)	687 (475-1124)
	Yogurt	8.2 (5.2-10.8)	7.5 (4.6-10.1)	287 (187-381)	524 (319-707)
	Sugar-sweetened beverages	15.5 (10-23.3)	35.9 (25.3-47.7)	546 (354-828)	2488 (1753-3324)
	Potatoes	2.6 (1.4-5.8)	29.8 (19.4-39.6)	92 (49-204)	2071 (1356-2799)
	Fruit	3.3 (3-3.5)	3.7 (3.4-3.9)	116 (105-127)	255 (232-280)
	Nuts and seeds	1.1 (0.9-1.3)	0.8 (0.6-1.1)	39 (32-46)	57 (39-73)
	Non-starchy vegetables	1.5 (1.4-1.7)	1.5 (1.3-1.6)	54 (48-61)	101 (87-114)
	Fruit juice	0.1 (0.1-0.3)	0.2 (0.1-0.3)	5 (3-10)	11 (6-21)
Kyrgyzstan	Suboptimal diet	87.5 (83.6-91.6)	79.5 (73.7-85.2)	1298 (1220-1381)	1832 (1694-1984)
	Whole grains	43.2 (41.9-44.5)	25.4 (17.5-32.2)	642 (610-669)	586 (408-751)
	Refined grains (total)	24.6 (19.5-30.9)	27 (21.6-33.3)	365 (291-461)	623 (497-778)
	Refined rice	0.6 (0.4-0.7)	2.4 (1.9-3.4)	8 (6-11)	56 (43-78)
	Refined wheat	24.2 (19.2-30.4)	25.1 (20-31.1)	359 (286-454)	581 (462-727)
	Processed meats	46.3 (31.2-64.1)	35.7 (21.7-53.7)	687 (455-942)	826 (501-1230)
	Unprocessed red meats	41.9 (35.6-48.6)	19.2 (15-24.5)	622 (527-723)	444 (348-568)
	Yogurt	9.2 (7.2-10.7)	9.4 (7.3-11)	136 (105-158)	217 (168-255)

Table S5. Proportional and absolute burden per 1M population of T2D incidence attributable to suboptimal diet* and each individual dietary factor† at the national level in 1990 and 2018

Country	Dietary factor	Proportional burden, %		Rate, per 1M population‡	
		1990	2018	1990	2018
	Sugar-sweetened beverages	13.1 (9-18.6)	17.7 (12.5-24.4)	193 (133-277)	408 (289-569)
	Potatoes	4.7 (2.8-8)	8.6 (5.3-14.3)	70 (41-118)	198 (121-332)
	Fruit	3 (2.7-3.2)	3.4 (3.1-3.7)	44 (40-48)	78 (71-86)
	Nuts and seeds	1.2 (1.1-1.4)	0.8 (0.6-1)	18 (16-20)	19 (15-23)
	Non-starchy vegetables	1.8 (1.6-2)	2.1 (1.9-2.3)	27 (24-29)	48 (43-53)
	Fruit juice	0.1 (0-0.1)	0.1 (0-0.1)	1 (1-1)	1 (1-2)
Laos	Suboptimal diet	69.5 (65.4-73.5)	76.4 (73.3-79.8)	1948 (1817-2075)	3399 (3226-3565)
	Whole grains	40.6 (39.2-41.9)	41.4 (40.1-42.7)	1138 (1088-1186)	1843 (1760-1916)
	Refined grains (total)	49.9 (43.4-55.9)	46.9 (41.4-52.4)	1396 (1207-1578)	2080 (1821-2320)
	Refined rice	49.8 (43.3-55.8)	46.6 (41.2-52.1)	1395 (1205-1576)	2067 (1809-2306)
	Refined wheat	0.1 (0.1-0.2)	0.6 (0.5-0.7)	4 (3-4)	25 (21-30)
	Processed meats	0.7 (0.4-1.9)	13.9 (8.5-22.3)	20 (11-53)	622 (377-990)
	Unprocessed red meats	4.2 (2.7-6.9)	15.5 (12.3-19.7)	117 (78-193)	686 (548-870)
	Yogurt	12.4 (11.6-13.2)	12.7 (11.9-13.6)	348 (323-374)	565 (528-606)
	Sugar-sweetened beverages	1.5 (1-3.1)	5 (3.5-7.4)	41 (28-88)	222 (157-331)
	Potatoes	0.5 (0.2-0.9)	0 (0-0)	13 (7-25)	0 (0-0)
	Fruit	2.4 (2.2-2.6)	2.7 (2.4-2.9)	67 (61-73)	119 (108-130)
	Nuts and seeds	1.3 (1.2-1.4)	1.4 (1.3-1.5)	37 (35-40)	64 (59-68)
	Non-starchy vegetables	0.9 (0.8-1)	1.1 (1-1.3)	26 (22-29)	50 (45-56)
	Fruit juice	0 (0-0)	0 (0-0)	0 (0-0)	0 (0-0)
Latvia	Suboptimal diet	92.2 (88-95.8)	91.6 (86.5-95.3)	1719 (1615-1812)	2789 (2616-2948)
	Whole grains	13.1 (8.9-17.3)	14.2 (9.7-18.3)	243 (168-324)	433 (299-558)
	Refined grains (total)	0.3 (0.2-0.5)	4.1 (2.5-6.6)	6 (3-9)	125 (77-200)
	Refined rice	0 (0-0)	0.3 (0.2-0.5)	0 (0-0)	9 (5-14)
	Refined wheat	0.3 (0.2-0.5)	3.8 (2.3-6.2)	6 (3-9)	116 (72-186)
	Processed meats	59.2 (40.9-77.6)	64.1 (46.3-80)	1104 (765-1444)	1956 (1409-2455)
	Unprocessed red meats	70.7 (64.9-74.3)	64.2 (56.5-70.9)	1317 (1193-1405)	1960 (1718-2171)
	Yogurt	6.8 (4.4-8.9)	6.2 (3.8-8.3)	127 (82-167)	187 (117-254)
	Sugar-sweetened beverages	4.6 (3.6-7.2)	4.1 (3.1-6)	86 (66-136)	124 (96-185)
	Potatoes	11.5 (9.1-14.7)	9.1 (7.3-12.1)	214 (169-276)	276 (222-375)
	Fruit	3.2 (3.1-3.4)	3.3 (3.2-3.5)	60 (56-64)	102 (96-109)
	Nuts and seeds	1.3 (1.2-1.4)	1.1 (1-1.2)	24 (22-26)	34 (30-38)
	Non-starchy vegetables	0.8 (0.7-0.9)	0.4 (0.3-0.6)	15 (13-18)	13 (10-17)
	Fruit juice	0.1 (0.1-0.2)	0.1 (0-0.2)	2 (1-4)	3 (1-7)
Lebanon	Suboptimal diet	70.2 (66.5-74)	73 (69.1-77)	2897 (2713-3084)	3140 (2923-3358)

Table S5. Proportional and absolute burden per 1M population of T2D incidence attributable to suboptimal diet* and each individual dietary factor† at the national level in 1990 and 2018

Country	Dietary factor	Proportional burden, %		Rate, per 1M population‡	
		1990	2018	1990	2018
	Whole grains	29.5 (26.9-32.2)	27.7 (24.3-30.7)	1220 (1099-1337)	1191 (1039-1339)
	Refined grains (total)	39.1 (33.4-45.3)	39.9 (33.9-46.3)	1611 (1366-1883)	1716 (1463-2003)
	Refined rice	6.4 (5.3-8.2)	5.9 (4.7-7.6)	264 (215-337)	252 (205-327)
	Refined wheat	35 (29.9-40.9)	36.2 (30.6-42.3)	1445 (1217-1697)	1557 (1323-1830)
	Processed meats	10.6 (6.7-15.9)	15.9 (11.3-23.5)	438 (280-658)	688 (480-1025)
	Unprocessed red meats	5.8 (4.2-9.3)	8.2 (5.9-12.4)	239 (175-389)	350 (254-534)
	Yogurt	5.5 (4.2-6.8)	5.5 (4.2-7)	228 (175-282)	238 (180-298)
	Sugar-sweetened beverages	21.1 (17.3-25.2)	21.5 (17.8-27)	869 (717-1043)	923 (764-1164)
	Potatoes	5.7 (4.8-7.7)	4.8 (4.1-6.7)	236 (199-316)	206 (174-286)
	Fruit	0.2 (0.2-0.3)	1.3 (0.9-1.6)	9 (7-13)	54 (41-67)
	Nuts and seeds	0.7 (0.5-0.8)	0.5 (0.4-0.7)	27 (22-31)	23 (17-29)
	Non-starchy vegetables	0.1 (0.1-0.2)	0.1 (0.1-0.2)	4 (3-7)	5 (3-8)
	Fruit juice	0.1 (0-0.1)	0.1 (0.1-0.1)	2 (2-3)	4 (3-5)
Lesotho	Suboptimal diet	53.6 (48.7-59.7)	71.7 (65.6-78.1)	1558 (1414-1737)	2763 (2508-3017)
	Whole grains	23.1 (15.3-29.3)	21.8 (13.4-29.1)	675 (447-856)	839 (528-1125)
	Refined grains (total)	15 (11.8-18.9)	15.7 (12.8-19.5)	435 (346-554)	605 (492-760)
	Refined rice	0.8 (0.7-1.2)	6.2 (5-8)	24 (19-34)	238 (190-311)
	Refined wheat	14.3 (11.2-18)	10.1 (8.2-12.8)	414 (328-527)	391 (316-496)
	Processed meats	4.8 (1.5-12.1)	6 (1.9-15.5)	140 (44-355)	228 (72-596)
	Unprocessed red meats	6.8 (4.9-10.2)	11.8 (8.7-16)	199 (142-294)	453 (338-617)
	Yogurt	10.7 (9.2-11.8)	11.7 (10.4-12.7)	311 (268-343)	451 (399-492)
	Sugar-sweetened beverages	16 (10.9-23)	41.1 (30.8-52.2)	463 (316-669)	1584 (1172-2020)
	Potatoes	1.5 (0.8-2.9)	5.7 (3.3-10.8)	44 (23-85)	219 (127-418)
	Fruit	2.7 (2.5-2.9)	3.2 (3-3.4)	79 (73-84)	124 (115-134)
	Nuts and seeds	1.2 (1.1-1.3)	1.3 (1.2-1.5)	34 (31-37)	52 (47-57)
	Non-starchy vegetables	0.1 (0-0.1)	0.8 (0.6-0.9)	1 (1-2)	29 (23-35)
	Fruit juice	0.1 (0-0.1)	0.1 (0.1-0.1)	2 (1-3)	3 (2-5)
Liberia	Suboptimal diet	68.4 (60.7-78.7)	83.2 (75.6-91)	1664 (1471-1941)	2619 (2355-2890)
	Whole grains	36.9 (33.8-38.8)	37.5 (32.4-40.6)	900 (818-957)	1179 (1011-1291)
	Refined grains (total)	23.2 (17.5-29.7)	21.5 (17.6-26.1)	564 (430-720)	677 (547-820)
	Refined rice	22.9 (17.4-29.4)	19.1 (15.5-23.2)	558 (426-712)	600 (481-732)
	Refined wheat	0.3 (0.2-0.4)	3 (2.4-4)	8 (6-11)	96 (76-127)
	Processed meats	22.8 (8.1-47.9)	43.6 (20.3-69.7)	553 (196-1171)	1363 (638-2192)
	Unprocessed red meats	13.2 (9.8-18)	12.1 (9.1-16.3)	323 (238-440)	380 (286-512)
	Yogurt	11.2 (10.2-12.1)	12.6 (11.8-13.5)	273 (245-298)	398 (368-428)

Table S5. Proportional and absolute burden per 1M population of T2D incidence attributable to suboptimal diet* and each individual dietary factor† at the national level in 1990 and 2018

Country	Dietary factor	Proportional burden, %		Rate, per 1M population‡	
		1990	2018	1990	2018
	Sugar-sweetened beverages	8.2 (5.1-12.9)	33.1 (24.2-44)	200 (125-317)	1039 (749-1391)
	Potatoes	0.3 (0.1-0.6)	0.4 (0.2-0.8)	7 (3-14)	12 (6-24)
	Fruit	2.8 (2.6-3)	3.5 (3.3-3.8)	68 (63-73)	112 (103-120)
	Nuts and seeds	1 (0.8-1.1)	1.3 (1.2-1.4)	24 (20-27)	41 (37-45)
	Non-starchy vegetables	1 (0.9-1.1)	1.3 (1.1-1.4)	25 (21-28)	40 (34-45)
	Fruit juice	0.1 (0-0.1)	0.1 (0.1-0.2)	2 (1-3)	4 (2-6)
Libya	Suboptimal diet	73 (65.9-80.2)	76.4 (68.9-83.9)	2110 (1892-2348)	5554 (5014-6150)
	Whole grains	35.1 (26.9-39.7)	35.6 (27.6-40.8)	1015 (773-1167)	2596 (1997-2992)
	Refined grains (total)	27.5 (16.3-40.5)	31.5 (19.7-44.1)	798 (467-1173)	2285 (1410-3240)
	Refined rice	27.5 (16.3-40.5)	31.5 (19.7-44.1)	798 (467-1173)	2285 (1410-3240)
	Refined wheat	0 (0-0)	0 (0-0)	0 (0-0)	0 (0-0)
	Processed meats	20.4 (9.2-39)	21.8 (9.8-41.2)	594 (266-1121)	1590 (714-3016)
	Unprocessed red meats	14.5 (9.3-21.6)	14.4 (9-21.7)	422 (269-625)	1047 (648-1572)
	Yogurt	8 (5.5-10.2)	8.1 (5.3-10.5)	232 (161-299)	591 (386-775)
	Sugar-sweetened beverages	17.8 (12.7-24.8)	20.1 (14.6-28.5)	516 (367-713)	1459 (1047-2081)
	Potatoes	3.2 (1.8-5.9)	5.5 (3.1-10)	94 (53-170)	397 (225-735)
	Fruit	3.2 (2.9-3.4)	3.6 (3.3-3.9)	91 (84-98)	263 (241-287)
	Nuts and seeds	1.1 (0.9-1.2)	0.9 (0.7-1.1)	32 (27-36)	64 (48-80)
	Non-starchy vegetables	1.2 (1-1.3)	1.3 (1.1-1.5)	33 (28-38)	98 (82-113)
	Fruit juice	0.1 (0.1-0.2)	0.1 (0.1-0.3)	3 (2-6)	10 (5-18)
Lithuania	Suboptimal diet	87.2 (82.8-91.1)	86.8 (82.3-90.7)	1524 (1426-1618)	2059 (1944-2173)
	Whole grains	35.1 (32.9-37.1)	34.9 (33-36.8)	613 (569-655)	829 (775-881)
	Refined grains (total)	0.2 (0.1-0.3)	2.7 (1.7-4.6)	3 (2-5)	64 (39-109)
	Refined rice	0 (0-0)	0.1 (0.1-0.2)	0 (0-0)	2 (1-4)
	Refined wheat	0.2 (0.1-0.3)	2.6 (1.6-4.4)	3 (2-5)	62 (38-104)
	Processed meats	38.5 (24.1-55.4)	47.5 (33.5-61)	673 (416-960)	1129 (804-1456)
	Unprocessed red meats	58.6 (49.1-67.1)	51.6 (41.9-60.7)	1022 (849-1174)	1225 (992-1450)
	Yogurt	6.7 (4.5-8.7)	6 (3.8-8)	117 (77-153)	142 (90-190)
	Sugar-sweetened beverages	4.3 (3.2-6.9)	4.9 (3.7-7.2)	74 (55-119)	116 (88-169)
	Potatoes	7.7 (6.3-10.2)	5 (4.1-6.6)	135 (110-180)	119 (96-158)
	Fruit	3.1 (2.9-3.3)	3.3 (3.1-3.5)	54 (51-59)	78 (73-83)
	Nuts and seeds	1.3 (1.2-1.4)	1.1 (0.9-1.2)	22 (20-24)	25 (22-29)
	Non-starchy vegetables	1.5 (1.4-1.6)	1.2 (1-1.3)	26 (24-28)	28 (24-32)
	Fruit juice	0.2 (0.1-0.3)	0.2 (0.1-0.3)	3 (2-6)	4 (2-6)
Luxembourg	Suboptimal diet	82.5 (74.6-90.1)	93.2 (85.2-97.1)	1402 (1272-1541)	5271 (4764-5580)

Table S5. Proportional and absolute burden per 1M population of T2D incidence attributable to suboptimal diet* and each individual dietary factor† at the national level in 1990 and 2018

Country	Dietary factor	Proportional burden, %		Rate, per 1M population‡	
		1990	2018	1990	2018
	Whole grains	38.9 (37.6-40.1)	28.4 (22.9-32.7)	663 (631-694)	1604 (1277-1871)
	Refined grains (total)	15.6 (10.6-20.8)	15.8 (11.6-21.5)	265 (182-356)	895 (647-1210)
	Refined rice	15.6 (10.6-20.8)	1.3 (0.9-1.9)	265 (182-356)	75 (53-107)
	Refined wheat	0 (0-0)	14.8 (10.8-20.1)	0 (0-0)	834 (601-1133)
	Processed meats	40.2 (21.3-62.7)	76.6 (51.4-87.7)	681 (365-1068)	4332 (2870-4999)
	Unprocessed red meats	28.9 (16.2-46.3)	39.6 (23.2-59.7)	492 (271-787)	2251 (1318-3394)
	Yogurt	7.5 (5.7-9.2)	7.9 (5.9-9.8)	128 (96-158)	447 (328-564)
	Sugar-sweetened beverages	9.1 (6.5-12.7)	11.2 (8.3-16.3)	154 (109-218)	636 (471-919)
	Potatoes	17.2 (12.5-23)	9.6 (6.4-15.3)	292 (213-394)	545 (364-874)
	Fruit	2.4 (2.1-2.7)	2.8 (2.5-3.2)	41 (36-46)	159 (139-179)
	Nuts and seeds	1 (0.8-1.2)	1.4 (1.3-1.5)	18 (14-20)	78 (70-86)
	Non-starchy vegetables	1.1 (0.8-1.2)	1.4 (1.1-1.6)	18 (14-21)	78 (65-89)
	Fruit juice	0.2 (0.1-0.5)	0.2 (0.1-0.5)	3 (1-8)	10 (4-29)
Macedonia	Suboptimal diet	73.3 (66.8-79.8)	85.5 (79.9-90.5)	2618 (2382-2876)	5876 (5443-6264)
	Whole grains	38.8 (36.5-40.5)	23.4 (16.7-29.8)	1387 (1295-1472)	1610 (1145-2059)
	Refined grains (total)	18.8 (13.7-25.4)	22.1 (16.2-29.6)	671 (490-906)	1520 (1097-2055)
	Refined rice	0.5 (0.3-0.7)	1 (0.7-1.5)	17 (12-26)	70 (48-105)
	Refined wheat	18.4 (13.4-24.9)	21.4 (15.6-28.7)	658 (480-889)	1468 (1060-1992)
	Processed meats	38.2 (24.9-53.1)	57.8 (43.3-71.3)	1370 (881-1903)	3965 (2960-4879)
	Unprocessed red meats	12.9 (9.1-17.8)	19.6 (14.6-25.7)	462 (323-639)	1337 (1005-1763)
	Yogurt	6.8 (4.7-8.8)	6.7 (4.6-8.7)	243 (166-315)	463 (315-599)
	Sugar-sweetened beverages	3 (2.2-5)	18.6 (14.6-24.2)	108 (78-180)	1273 (999-1665)
	Potatoes	5.5 (3.4-9.4)	12.3 (7.8-19.1)	197 (120-338)	848 (540-1295)
	Fruit	2.9 (2.8-3.2)	2.9 (2.6-3.2)	105 (97-114)	197 (177-218)
	Nuts and seeds	1.2 (1.2-1.3)	1.1 (0.9-1.2)	44 (41-48)	72 (62-82)
	Non-starchy vegetables	0.8 (0.7-1)	0.9 (0.7-1.1)	30 (23-36)	64 (50-79)
	Fruit juice	0.1 (0.1-0.2)	0.1 (0.1-0.2)	4 (3-7)	8 (5-13)
Madagascar	Suboptimal diet	69.5 (64.6-75.2)	67.3 (62.4-73)	1155 (1066-1258)	1320 (1217-1437)
	Whole grains	31.3 (24.5-36)	33 (25.7-37.6)	521 (407-598)	647 (502-741)
	Refined grains (total)	20.9 (17.8-24.6)	20.9 (17.5-24.5)	347 (293-413)	410 (343-484)
	Refined rice	20.3 (17.2-23.9)	19.7 (16.5-23.2)	337 (285-401)	387 (323-458)
	Refined wheat	0.7 (0.6-0.9)	1.4 (1.2-1.9)	12 (10-15)	28 (23-36)
	Processed meats	9.9 (3.5-25.2)	8.7 (3-21.8)	165 (57-419)	171 (58-430)
	Unprocessed red meats	14.6 (11.4-18.4)	13.9 (10.7-18.1)	241 (189-305)	271 (211-351)
	Yogurt	12.1 (11.2-13)	12.9 (12-13.7)	202 (186-217)	253 (235-271)

Table S5. Proportional and absolute burden per 1M population of T2D incidence attributable to suboptimal diet* and each individual dietary factor† at the national level in 1990 and 2018

Country	Dietary factor	Proportional burden, %		Rate, per 1M population‡	
		1990	2018	1990	2018
	Sugar-sweetened beverages	20.4 (13.7-28.2)	19.9 (13.5-28.1)	341 (230-467)	387 (265-548)
	Potatoes	8.7 (5.1-15.1)	2.1 (1.2-4.2)	145 (85-252)	42 (24-84)
	Fruit	2.5 (2.4-2.7)	3 (2.8-3.2)	42 (39-45)	58 (54-63)
	Nuts and seeds	1.2 (1.1-1.3)	1.4 (1.3-1.6)	20 (18-21)	28 (26-31)
	Non-starchy vegetables	0.8 (0.7-0.9)	0.9 (0.8-1.1)	13 (11-15)	18 (15-21)
	Fruit juice	0 (0-0)	0 (0-0.1)	1 (0-1)	1 (1-1)
Malawi	Suboptimal diet	50.1 (45.6-55.8)	56 (50.8-63)	978 (886-1085)	1370 (1227-1545)
	Whole grains	11.9 (7.4-16.6)	16.1 (11.2-20.7)	231 (145-327)	393 (271-506)
	Refined grains (total)	21.5 (17.9-25.6)	22 (18.6-26.1)	418 (349-506)	538 (454-646)
	Refined rice	13.8 (11.4-16.7)	9.7 (8.1-12)	268 (221-326)	238 (198-293)
	Refined wheat	9 (7.4-11.2)	13.6 (11.5-16.5)	175 (143-219)	333 (279-406)
	Processed meats	4.4 (1.3-12.8)	9.3 (2.8-23.5)	86 (25-250)	226 (67-565)
	Unprocessed red meats	4.9 (3.3-8.7)	6 (4.2-10.1)	95 (64-170)	147 (103-245)
	Yogurt	12.5 (11.3-13.4)	13.1 (12-14)	243 (220-266)	319 (292-345)
	Sugar-sweetened beverages	9.6 (6.2-14.6)	5.3 (3.5-8.5)	186 (120-283)	129 (85-208)
	Potatoes	0.7 (0.4-1.3)	6.3 (3.4-11.9)	13 (7-25)	154 (84-294)
	Fruit	2.3 (2.1-2.5)	2.7 (2.4-2.9)	45 (41-50)	65 (60-72)
	Nuts and seeds	1.3 (1.2-1.4)	1.1 (0.9-1.3)	25 (23-28)	27 (23-32)
	Non-starchy vegetables	0.4 (0.3-0.5)	0.9 (0.8-1)	8 (6-10)	21 (18-24)
	Fruit juice	0.1 (0-0.1)	0.1 (0-0.1)	1 (1-1)	1 (1-2)
Malaysia	Suboptimal diet	74.7 (67.4-82.6)	69.9 (62.2-78.5)	2237 (1998-2490)	3270 (2898-3712)
	Whole grains	39.9 (37.3-41.5)	33.1 (27.2-37.1)	1194 (1110-1258)	1549 (1267-1749)
	Refined grains (total)	43.4 (28.4-61.1)	39.7 (25.9-57.6)	1303 (856-1844)	1855 (1209-2679)
	Refined rice	38.3 (24.5-55.2)	30.5 (19.3-46)	1149 (742-1670)	1423 (900-2143)
	Refined wheat	8.6 (5-14.2)	13.5 (8.1-22.1)	256 (154-429)	636 (384-1037)
	Processed meats	18.8 (13.4-24.9)	12.9 (8.7-18.9)	561 (402-745)	604 (404-894)
	Unprocessed red meats	8.3 (6.1-11.6)	6.6 (4.8-9.7)	248 (183-346)	309 (222-452)
	Yogurt	12.8 (11.9-13.6)	12.4 (11.4-13.2)	384 (357-406)	581 (527-622)
	Sugar-sweetened beverages	7.4 (5.5-10.2)	7.1 (5.2-10.1)	222 (163-303)	334 (242-470)
	Potatoes	0.3 (0.2-0.7)	3.1 (1.6-6.4)	10 (5-21)	144 (74-301)
	Fruit	2.6 (2.4-2.7)	2.8 (2.6-3)	77 (73-82)	132 (122-142)
	Nuts and seeds	0.5 (0.4-0.6)	0.5 (0.4-0.6)	14 (12-17)	22 (18-27)
	Non-starchy vegetables	1.1 (1-1.2)	1 (0.9-1.1)	33 (30-36)	46 (40-52)
	Fruit juice	0.1 (0.1-0.1)	0.1 (0.1-0.1)	2 (2-3)	4 (3-5)
Maldives	Suboptimal diet	65.9 (60-71.4)	71.4 (64.5-80.9)	1566 (1421-1697)	1964 (1765-2236)

Table S5. Proportional and absolute burden per 1M population of T2D incidence attributable to suboptimal diet* and each individual dietary factor† at the national level in 1990 and 2018

Country	Dietary factor	Proportional burden, %		Rate, per 1M population‡	
		1990	2018	1990	2018
	Whole grains	2.9 (1.9-4.2)	7.7 (3.2-17.2)	69 (44-98)	211 (88-470)
	Refined grains (total)	39 (33.4-45.2)	40.3 (34.4-46.4)	929 (795-1076)	1107 (945-1281)
	Refined rice	27.3 (23-32.2)	26.9 (22.5-31.5)	649 (548-765)	737 (617-873)
	Refined wheat	16.4 (13.6-19.6)	18.7 (15.5-22.2)	390 (323-463)	513 (424-614)
	Processed meats	1.1 (0.2-6.2)	18.3 (4.8-46.7)	26 (5-148)	505 (132-1281)
	Unprocessed red meats	1.9 (1.1-3.7)	12 (8.7-16.3)	45 (26-89)	329 (238-445)
	Yogurt	11.3 (10.5-12.1)	11.5 (10.3-12.4)	270 (249-289)	314 (283-342)
	Sugar-sweetened beverages	28 (18.3-38.3)	12.4 (7.3-20.1)	662 (434-912)	339 (200-553)
	Potatoes	0.8 (0.4-1.8)	4.6 (2.2-10)	20 (10-43)	126 (61-273)
	Fruit	3.5 (3.4-3.6)	1.6 (1.2-1.9)	84 (80-88)	43 (33-52)
	Nuts and seeds	1.1 (0.8-1.2)	1 (0.7-1.2)	25 (20-29)	27 (19-34)
	Non-starchy vegetables	1.7 (1.6-1.8)	0.9 (0.7-1.1)	41 (39-44)	26 (20-31)
	Fruit juice	0 (0-0)	0 (0-0)	0 (0-1)	0 (0-1)
Mali	Suboptimal diet	51.2 (41.9-62.3)	56 (49.2-66)	961 (794-1172)	1499 (1313-1765)
	Whole grains	21.1 (13.1-28.5)	21.9 (14-29.5)	395 (246-534)	588 (371-796)
	Refined grains (total)	7.7 (6.2-10)	11.7 (9.6-14.6)	145 (115-187)	313 (258-392)
	Refined rice	7.1 (5.7-9.2)	10.2 (8.4-12.9)	133 (105-173)	273 (225-347)
	Refined wheat	0.7 (0.6-0.9)	1.6 (1.4-2)	13 (11-17)	44 (36-55)
	Processed meats	14.9 (5.5-34.2)	13.1 (4.7-31)	282 (102-625)	353 (125-824)
	Unprocessed red meats	6.8 (4.7-11.1)	10.2 (7.4-14.8)	127 (88-209)	274 (198-394)
	Yogurt	11.5 (10.4-12.7)	12 (10.9-13.1)	217 (194-240)	322 (290-353)
	Sugar-sweetened beverages	5.9 (3.7-10)	9.2 (5.7-14.7)	112 (69-188)	247 (155-395)
	Potatoes	0.7 (0.4-1.4)	2 (1.1-4.4)	14 (8-27)	55 (31-116)
	Fruit	2.8 (2.6-2.9)	3.1 (2.9-3.4)	52 (48-56)	85 (78-91)
	Nuts and seeds	0.7 (0.4-1)	0.8 (0.6-1.2)	13 (8-19)	22 (15-31)
	Non-starchy vegetables	1.2 (1.1-1.4)	1.5 (1.3-1.6)	23 (21-26)	39 (36-43)
	Fruit juice	0.1 (0.1-0.1)	0.1 (0.1-0.2)	2 (1-2)	3 (2-4)
Malta	Suboptimal diet	80.7 (74.8-86.7)	81.5 (75-87.4)	3123 (2858-3394)	4604 (4211-5004)
	Whole grains	23.2 (17.7-28.2)	15.2 (10.4-20.6)	899 (689-1092)	856 (591-1168)
	Refined grains (total)	33.5 (19.9-37.7)	32.9 (19.9-36.7)	1288 (768-1476)	1856 (1116-2102)
	Refined rice	1.8 (1-2.3)	2.4 (1.3-3.1)	71 (39-89)	136 (75-179)
	Refined wheat	32.2 (19.1-36.5)	31.3 (18.8-35)	1241 (737-1424)	1764 (1053-2001)
	Processed meats	38.3 (25.4-55)	43.6 (29.6-60.1)	1477 (981-2126)	2472 (1667-3414)
	Unprocessed red meats	30.4 (22.1-40.5)	24.7 (17.2-34.3)	1175 (849-1560)	1394 (964-1949)
	Yogurt	7.7 (5.7-9.5)	7.5 (5.6-9.2)	298 (221-371)	424 (314-528)

Table S5. Proportional and absolute burden per 1M population of T2D incidence attributable to suboptimal diet* and each individual dietary factor† at the national level in 1990 and 2018

Country	Dietary factor	Proportional burden, %		Rate, per 1M population‡	
		1990	2018	1990	2018
	Sugar-sweetened beverages	11.9 (8.7-16.9)	18.5 (13.9-24.8)	461 (332-655)	1045 (778-1411)
	Potatoes	2.9 (1.6-5.6)	2.8 (1.6-5.3)	111 (62-220)	159 (90-301)
	Fruit	1.9 (1.7-2.2)	2.1 (1.8-2.4)	75 (64-85)	120 (103-135)
	Nuts and seeds	0.9 (0.7-1.1)	1 (0.8-1.2)	36 (27-43)	56 (43-66)
	Non-starchy vegetables	1.2 (1.1-1.3)	1.4 (1.3-1.5)	47 (42-52)	78 (70-87)
	Fruit juice	0.3 (0.2-0.4)	0.4 (0.3-0.5)	13 (9-17)	20 (15-27)
Marshall Islands	Suboptimal diet	74.9 (67.7-81.6)	77.7 (70.6-83.8)	3900 (3519-4290)	8909 (7963-9684)
	Whole grains	40.2 (34.8-43.3)	40.8 (34.5-44.3)	2090 (1804-2278)	4657 (3946-5101)
	Refined grains (total)	36.5 (23.1-50.4)	41.6 (26.7-56.2)	1908 (1207-2643)	4776 (3015-6398)
	Refined rice	36.5 (23.1-50.4)	41.6 (26.7-56.2)	1908 (1207-2643)	4776 (3015-6398)
	Refined wheat	0 (0-0)	0 (0-0)	0 (0-0)	0 (0-0)
	Processed meats	12.7 (6-26.2)	13.3 (6.3-25.7)	654 (316-1379)	1516 (720-2966)
	Unprocessed red meats	23.4 (16.9-31)	16.3 (10.8-23.3)	1219 (879-1617)	1863 (1235-2658)
	Yogurt	14 (12.9-15.1)	14.3 (13-15.4)	731 (664-795)	1627 (1466-1790)
	Sugar-sweetened beverages	5.5 (3.6-9.1)	14.2 (9.9-20.3)	286 (183-482)	1617 (1119-2320)
	Potatoes	0.2 (0.1-0.5)	1.5 (0.8-3.3)	13 (7-26)	172 (86-375)
	Fruit	3.1 (2.8-3.3)	3.2 (2.9-3.5)	159 (145-175)	363 (324-408)
	Nuts and seeds	1.3 (1.2-1.4)	1.4 (1.3-1.6)	68 (60-76)	162 (142-182)
	Non-starchy vegetables	0.9 (0.6-1.1)	1 (0.7-1.3)	45 (33-56)	113 (83-145)
	Fruit juice	0 (0-0.1)	0.1 (0-0.1)	2 (2-4)	6 (4-10)
Mauritania	Suboptimal diet	72.3 (62.5-83.6)	79.7 (69.5-89.1)	1127 (976-1307)	1390 (1221-1566)
	Whole grains	31.8 (25.9-35.7)	29.8 (22.8-34.8)	495 (400-559)	519 (401-607)
	Refined grains (total)	21.7 (15.9-28)	21.8 (16.3-28.3)	338 (247-434)	379 (283-496)
	Refined rice	12.9 (9.3-17.1)	10.8 (7.9-14.8)	202 (145-267)	187 (136-255)
	Refined wheat	10.5 (7.5-13.9)	12.8 (9.4-17.2)	163 (117-215)	223 (163-302)
	Processed meats	33.5 (12.7-60.5)	38.8 (15.1-67.2)	520 (199-952)	676 (264-1165)
	Unprocessed red meats	12.1 (8.8-16.1)	8.9 (6-13.4)	189 (136-252)	155 (104-233)
	Yogurt	10.5 (9-11.6)	10.7 (9.3-11.9)	163 (141-182)	187 (161-209)
	Sugar-sweetened beverages	12.6 (8-18.4)	31.7 (22.3-43.9)	197 (124-287)	554 (389-768)
	Potatoes	0.3 (0.2-0.6)	1.8 (1-3.5)	5 (3-10)	32 (18-61)
	Fruit	3.3 (3.2-3.4)	3.6 (3.4-3.8)	51 (49-54)	63 (59-66)
	Nuts and seeds	1.1 (1-1.2)	1.3 (1.2-1.4)	18 (16-19)	22 (20-24)
	Non-starchy vegetables	1.4 (1.3-1.5)	0.5 (0.3-0.7)	22 (20-24)	9 (6-12)
	Fruit juice	0.1 (0.1-0.2)	0.1 (0.1-0.2)	2 (1-3)	2 (1-3)
Mauritius	Suboptimal diet	89.6 (86-93)	85.4 (80.1-90.1)	3844 (3651-4028)	8936 (8300-9512)

Table S5. Proportional and absolute burden per 1M population of T2D incidence attributable to suboptimal diet* and each individual dietary factor† at the national level in 1990 and 2018

Country	Dietary factor	Proportional burden, %		Rate, per 1M population‡	
		1990	2018	1990	2018
	Whole grains	32.8 (25.7-37.7)	18.5 (11.1-26.3)	1403 (1096-1616)	1945 (1154-2761)
	Refined grains (total)	20.8 (15.7-26.2)	12.9 (9.2-17.1)	893 (677-1123)	1348 (962-1803)
	Refined rice	13.9 (10.3-17.7)	5.2 (3.6-7.4)	595 (444-760)	548 (384-778)
	Refined wheat	8.6 (6.3-11.2)	8.2 (5.8-11.3)	367 (271-485)	858 (604-1187)
	Processed meats	28.9 (20.1-39)	39.8 (29.2-50.8)	1235 (856-1678)	4150 (3061-5312)
	Unprocessed red meats	20.7 (8.6-40.4)	18.9 (7.7-38.1)	889 (370-1745)	1984 (803-4007)
	Yogurt	11.3 (9.9-12.5)	10.3 (8.7-11.5)	484 (423-536)	1072 (907-1218)
	Sugar-sweetened beverages	65.1 (57-72.1)	52.2 (42.6-61.6)	2796 (2462-3108)	5460 (4464-6472)
	Potatoes	3.1 (1.7-5.9)	3.8 (2.1-7.4)	133 (74-254)	393 (215-766)
	Fruit	3 (2.8-3.1)	3.2 (3-3.4)	127 (119-135)	332 (308-356)
	Nuts and seeds	1.2 (1-1.3)	1.1 (0.9-1.2)	49 (43-55)	110 (93-128)
	Non-starchy vegetables	0.8 (0.7-0.9)	0.9 (0.8-1.1)	35 (29-40)	96 (81-111)
	Fruit juice	0.1 (0.1-0.3)	0.1 (0.1-0.2)	6 (4-11)	10 (6-18)
Mexico	Suboptimal diet	78.3 (75.8-80.6)	79.1 (76.9-81.8)	4352 (4165-4528)	6015 (5751-6275)
	Whole grains	39.3 (38.1-40.5)	40.3 (38.9-41.7)	2188 (2097-2279)	3064 (2924-3201)
	Refined grains (total)	4.1 (3.7-5.3)	8.2 (7.1-10)	230 (202-293)	619 (543-765)
	Refined rice	0.7 (0.6-0.8)	2.2 (1.9-2.9)	38 (33-47)	165 (144-222)
	Refined wheat	3.5 (3.1-4.5)	6.1 (5.3-7.7)	193 (170-249)	462 (404-592)
	Processed meats	30.5 (24.9-36.6)	26.1 (20.9-33.2)	1695 (1377-2052)	1984 (1575-2520)
	Unprocessed red meats	11.3 (9.3-14.6)	18.8 (16-23.2)	627 (513-807)	1429 (1217-1767)
	Yogurt	11.7 (11.1-12.5)	12 (11.3-12.9)	652 (611-697)	914 (850-986)
	Sugar-sweetened beverages	30.4 (26.9-34.3)	30.9 (27.2-35.9)	1693 (1487-1916)	2345 (2046-2721)
	Potatoes	1 (0.9-1.1)	1.4 (1.2-1.7)	53 (47-62)	106 (93-127)
	Fruit	2.8 (2.6-2.9)	2.3 (2.2-2.5)	154 (145-163)	177 (163-193)
	Nuts and seeds	1.3 (1.2-1.3)	1.4 (1.3-1.5)	70 (65-75)	103 (95-111)
	Non-starchy vegetables	1.4 (1.3-1.4)	0.8 (0.7-0.9)	76 (71-81)	62 (54-70)
	Fruit juice	0 (0-0.1)	0.1 (0-0.1)	3 (2-3)	4 (3-5)
Moldova	Suboptimal diet	91.6 (85.6-95.3)	85.9 (78.8-91.8)	2279 (2112-2419)	2610 (2374-2805)
	Whole grains	27.5 (20.4-33.6)	21.9 (14.8-29.3)	685 (508-842)	664 (452-894)
	Refined grains (total)	1.1 (0.7-1.7)	13.7 (9.6-19.3)	27 (18-42)	417 (290-594)
	Refined rice	0 (0-0)	1.8 (1.2-2.9)	0 (0-0)	55 (37-88)
	Refined wheat	1.1 (0.7-1.7)	12.2 (8.5-17.3)	27 (18-42)	371 (257-529)
	Processed meats	58.2 (42.8-71.6)	61.9 (48-74.3)	1447 (1054-1798)	1884 (1457-2263)
	Unprocessed red meats	62.1 (42.7-74.3)	38 (21.9-58.1)	1549 (1065-1875)	1156 (663-1774)
	Yogurt	7.8 (5.3-9.8)	7.2 (5-9.2)	195 (133-242)	220 (152-280)

Table S5. Proportional and absolute burden per 1M population of T2D incidence attributable to suboptimal diet* and each individual dietary factor† at the national level in 1990 and 2018

Country	Dietary factor	Proportional burden, %		Rate, per 1M population‡	
		1990	2018	1990	2018
	Sugar-sweetened beverages	6.1 (4.1-9.5)	2.4 (1.6-4.4)	151 (101-232)	74 (48-136)
	Potatoes	10.1 (6.4-16.3)	6.2 (3.9-11.1)	253 (160-401)	191 (116-335)
	Fruit	3.1 (2.4-3.5)	3 (2-3.7)	76 (58-89)	91 (59-112)
	Nuts and seeds	0.8 (0.7-1)	1 (0.8-1.2)	20 (16-24)	31 (26-36)
	Non-starchy vegetables	1.4 (0.8-1.8)	1.6 (0.9-2)	35 (21-45)	47 (27-60)
	Fruit juice	0.2 (0.1-0.3)	0.2 (0.1-0.3)	4 (2-6)	5 (3-8)
Mongolia	Suboptimal diet	92.3 (87.3-95.9)	96.4 (93.3-97.6)	809 (757-856)	1623 (1547-1696)
	Whole grains	16.6 (10.4-24.5)	13.2 (7.5-20.9)	146 (91-217)	221 (125-352)
	Refined grains (total)	23.1 (16.6-30.7)	24.1 (17.4-32.1)	203 (146-270)	406 (291-542)
	Refined rice	3.3 (2.3-4.8)	3.8 (2.5-5.7)	29 (20-42)	63 (43-96)
	Refined wheat	20.7 (14.8-27.7)	21.4 (15.3-28.7)	182 (130-245)	361 (256-486)
	Processed meats	67.2 (47.7-82.3)	87.4 (77.8-90.9)	591 (416-728)	1470 (1320-1559)
	Unprocessed red meats	51 (43.7-58.6)	36.7 (29.7-44.8)	447 (382-519)	620 (497-759)
	Yogurt	8.6 (5.9-10.6)	8.2 (5.6-10.3)	75 (52-93)	138 (95-174)
	Sugar-sweetened beverages	14.7 (10.1-20.7)	12.6 (8.6-18.4)	130 (88-184)	213 (144-313)
	Potatoes	5.6 (3.4-9.8)	14.9 (9.5-22.9)	49 (30-86)	252 (158-389)
	Fruit	3.4 (3.2-3.6)	3.8 (3.6-4.1)	30 (28-32)	65 (60-70)
	Nuts and seeds	1.5 (1.4-1.6)	1.5 (1.3-1.6)	13 (12-14)	25 (22-28)
	Non-starchy vegetables	0.6 (0.5-0.8)	0.7 (0.5-0.9)	6 (4-7)	12 (9-16)
	Fruit juice	0.3 (0.2-0.6)	0.2 (0.1-0.4)	3 (2-5)	4 (2-7)
Montenegro	Suboptimal diet	80.6 (72.5-88.7)	92.9 (86.6-96.9)	2863 (2561-3176)	5272 (4896-5589)
	Whole grains	27.9 (20.5-33.7)	2.9 (1.8-4.4)	989 (730-1203)	167 (99-254)
	Refined grains (total)	15.6 (9.5-24.7)	11.2 (7.9-16.2)	555 (339-877)	631 (447-934)
	Refined rice	15.6 (9.5-24.7)	0.4 (0.3-0.6)	555 (339-877)	23 (16-35)
	Refined wheat	0 (0-0)	10.8 (7.7-15.7)	0 (0-0)	611 (433-906)
	Processed meats	44.3 (26.8-66.5)	65.6 (41.1-84.3)	1583 (953-2364)	3734 (2352-4783)
	Unprocessed red meats	33 (20.9-48.8)	62.7 (46.9-72.2)	1177 (750-1725)	3546 (2642-4137)
	Yogurt	6.6 (4.4-8.5)	5.8 (3.7-7.9)	234 (154-301)	332 (208-451)
	Sugar-sweetened beverages	4.1 (1.6-8.9)	6.4 (2.6-13.8)	145 (58-314)	366 (145-782)
	Potatoes	11.3 (7-17.9)	25.4 (17.4-33.3)	404 (250-647)	1438 (981-1908)
	Fruit	3 (2.5-3.3)	1.1 (0.4-2.1)	106 (89-119)	62 (22-121)
	Nuts and seeds	1.1 (0.9-1.2)	1 (0.7-1.2)	38 (30-44)	56 (41-71)
	Non-starchy vegetables	1 (0.4-1.4)	1 (0.4-1.5)	36 (14-50)	59 (22-88)
	Fruit juice	0.1 (0-0.3)	0.1 (0-0.2)	4 (1-9)	5 (2-13)
Morocco	Suboptimal diet	68.4 (59.8-76.8)	81.2 (73.4-87.4)	1683 (1466-1899)	4263 (3843-4627)

Table S5. Proportional and absolute burden per 1M population of T2D incidence attributable to suboptimal diet* and each individual dietary factor† at the national level in 1990 and 2018

Country	Dietary factor	Proportional burden, %		Rate, per 1M population‡	
		1990	2018	1990	2018
	Whole grains	30.7 (21.8-37.1)	30.4 (21.6-37.2)	757 (535-915)	1598 (1119-1956)
	Refined grains (total)	29.5 (19.1-41.4)	47.8 (35.5-57.7)	728 (475-1026)	2506 (1845-3030)
	Refined rice	0.5 (0.3-0.8)	1.9 (1.3-2.7)	12 (7-19)	101 (67-145)
	Refined wheat	29.2 (18.8-41.1)	46.9 (34.7-56.8)	721 (470-1016)	2458 (1808-2978)
	Processed meats	18.4 (8.4-34.4)	29.8 (15.5-49.1)	452 (205-848)	1569 (813-2619)
	Unprocessed red meats	7.1 (3-15.3)	9.6 (4.2-19.9)	174 (74-375)	504 (216-1031)
	Yogurt	8.3 (4.8-10.7)	7.5 (3.9-10.1)	204 (117-265)	394 (200-531)
	Sugar-sweetened beverages	14.7 (10.4-22)	17.1 (11.7-24.5)	364 (254-540)	901 (610-1303)
	Potatoes	2.4 (1.3-4.7)	4.7 (2.6-9.2)	60 (33-117)	250 (135-493)
	Fruit	3.1 (2.9-3.2)	2.9 (2.6-3.1)	76 (70-81)	151 (134-166)
	Nuts and seeds	1.1 (1-1.3)	0.9 (0.7-1.1)	28 (24-31)	48 (38-59)
	Non-starchy vegetables	0.8 (0.6-1)	0.9 (0.7-1.1)	20 (16-24)	46 (35-56)
	Fruit juice	0.1 (0.1-0.2)	0.1 (0.1-0.2)	3 (2-6)	7 (4-13)
Mozambique	Suboptimal diet	47.8 (43.2-51.9)	59.3 (54.4-65.8)	932 (832-1022)	1365 (1241-1519)
	Whole grains	32.4 (27-35.9)	34.1 (28.4-37.8)	630 (518-704)	787 (652-878)
	Refined grains (total)	6.6 (5.1-8.7)	16.5 (13.3-20.3)	128 (100-170)	379 (305-474)
	Refined rice	4.2 (3.3-5.7)	12.9 (10.4-16.2)	82 (64-111)	298 (238-376)
	Refined wheat	2.4 (1.9-3.4)	4 (3.3-5.7)	47 (37-66)	93 (74-131)
	Processed meats	2 (0.6-7.2)	6.8 (2.2-19.2)	39 (11-142)	156 (50-440)
	Unprocessed red meats	1.3 (0.8-3.6)	9.5 (6.6-14.1)	25 (15-69)	218 (151-323)
	Yogurt	12.1 (11.3-13)	13 (12.1-14)	237 (217-257)	300 (276-324)
	Sugar-sweetened beverages	5.5 (3.1-9.7)	11.1 (6.4-18.6)	108 (60-188)	255 (148-430)
	Potatoes	0.2 (0.1-0.4)	0.9 (0.5-1.6)	4 (3-8)	20 (12-37)
	Fruit	2.4 (2.2-2.6)	2.8 (2.6-3)	47 (42-51)	64 (59-71)
	Nuts and seeds	0.8 (0.7-1)	0.9 (0.8-1.1)	16 (13-20)	22 (18-26)
	Non-starchy vegetables	0.9 (0.8-1)	1.1 (1-1.3)	18 (16-20)	26 (22-29)
	Fruit juice	0 (0-0.1)	0.1 (0-0.1)	1 (1-1)	1 (1-2)
Myanmar	Suboptimal diet	57.4 (50.3-64.4)	78.7 (72.2-84.4)	1802 (1577-2036)	3877 (3546-4171)
	Whole grains	35.8 (30.9-38.8)	30.8 (24.4-35.4)	1121 (974-1233)	1521 (1198-1756)
	Refined grains (total)	31.2 (21-41.6)	30 (20.8-40.2)	982 (659-1299)	1485 (1050-1996)
	Refined rice	31 (20.8-41.3)	28.4 (19.7-38.3)	975 (653-1290)	1409 (992-1903)
	Refined wheat	0.4 (0.3-0.6)	2.5 (1.6-3.9)	13 (8-19)	122 (79-191)
	Processed meats	3.9 (1.1-11.6)	25.7 (14.1-41.5)	124 (35-367)	1266 (695-2046)
	Unprocessed red meats	1.2 (0.5-3.3)	34.1 (26.2-41.8)	37 (17-104)	1676 (1284-2075)
	Yogurt	12.5 (11.6-13.3)	12.3 (11.3-13.1)	392 (362-420)	604 (557-646)

Table S5. Proportional and absolute burden per 1M population of T2D incidence attributable to suboptimal diet* and each individual dietary factor† at the national level in 1990 and 2018

Country	Dietary factor	Proportional burden, %		Rate, per 1M population‡	
		1990	2018	1990	2018
	Sugar-sweetened beverages	1.4 (0.9-2.8)	8.9 (6.3-12.7)	43 (28-88)	435 (310-622)
	Potatoes	0.2 (0.1-0.3)	0.6 (0.3-1.3)	6 (3-11)	31 (16-65)
	Fruit	2.8 (2.6-3)	3.1 (2.9-3.3)	88 (82-94)	152 (141-163)
	Nuts and seeds	1.3 (1.2-1.4)	1.2 (1-1.3)	40 (37-43)	57 (50-64)
	Non-starchy vegetables	1.3 (1.2-1.4)	0.9 (0.7-1)	40 (36-43)	43 (36-50)
	Fruit juice	0 (0-0.1)	0 (0-0.1)	1 (1-2)	2 (1-3)
Namibia	Suboptimal diet	74.6 (66.8-83.2)	71.5 (65-78.8)	2167 (1943-2422)	2368 (2137-2624)
	Whole grains	18.8 (11.7-26.3)	23.1 (15.3-29.9)	547 (336-765)	764 (500-992)
	Refined grains (total)	13.6 (10.2-17.6)	14.9 (12.1-18)	395 (293-515)	492 (400-598)
	Refined rice	0 (0-0)	4.2 (3.4-5.4)	0 (0-0)	138 (111-178)
	Refined wheat	13.6 (10.2-17.6)	11.2 (9-13.6)	395 (293-515)	369 (299-451)
	Processed meats	17.7 (5.9-40.9)	9.4 (3.1-24)	515 (171-1181)	310 (101-797)
	Unprocessed red meats	18.1 (14.5-22.4)	11.4 (8.8-14.8)	526 (423-652)	377 (291-492)
	Yogurt	11.3 (10.1-12.1)	11.5 (10.3-12.4)	327 (292-354)	381 (338-414)
	Sugar-sweetened beverages	41.7 (30.7-54.3)	41.7 (30.2-54.2)	1211 (893-1585)	1379 (1004-1801)
	Potatoes	0 (0-0)	3.3 (1.9-6.3)	0 (0-0)	110 (63-208)
	Fruit	2.6 (2.4-2.7)	1.2 (1-1.4)	74 (70-79)	39 (33-45)
	Nuts and seeds	1 (0.9-1.1)	1.1 (0.9-1.2)	29 (25-33)	36 (31-41)
	Non-starchy vegetables	0.8 (0.8-0.9)	1 (0.9-1.2)	25 (22-27)	35 (31-38)
	Fruit juice	0.1 (0.1-0.1)	0.1 (0.1-0.1)	2 (2-4)	3 (2-5)
Nepal	Suboptimal diet	61.6 (56.9-66.5)	66.6 (61.4-71.8)	1219 (1119-1322)	2501 (2291-2717)
	Whole grains	22.8 (11.7-33.7)	21.3 (10.3-32.5)	449 (232-671)	797 (384-1228)
	Refined grains (total)	41.2 (36.2-47.4)	40.3 (35.5-46.1)	815 (718-935)	1509 (1318-1721)
	Refined rice	36.2 (31.6-42)	32.9 (28.7-38)	717 (627-827)	1233 (1066-1429)
	Refined wheat	7.9 (6.8-9.9)	11.1 (9.5-13.6)	157 (133-197)	414 (353-510)
	Processed meats	0.8 (0.2-5.5)	0.9 (0.2-5.6)	16 (4-109)	34 (7-208)
	Unprocessed red meats	3.6 (2.4-7.7)	4.3 (3-7.5)	72 (48-153)	162 (112-283)
	Yogurt	13.3 (12.4-14.3)	13.3 (12.5-14.2)	264 (243-285)	500 (466-536)
	Sugar-sweetened beverages	1.4 (0.6-3.1)	6.9 (3.6-12.4)	27 (13-62)	258 (134-474)
	Potatoes	1.8 (0.9-4.3)	10.6 (5-20.3)	36 (18-84)	398 (186-760)
	Fruit	3.9 (3.7-4.1)	4 (3.8-4.2)	77 (72-82)	150 (142-160)
	Nuts and seeds	1.4 (1.3-1.5)	1.3 (1-1.5)	28 (25-30)	47 (37-55)
	Non-starchy vegetables	0.7 (0.6-0.8)	0.8 (0.7-0.9)	13 (11-15)	29 (25-34)
	Fruit juice	0 (0-0)	0 (0-0)	0 (0-0)	0 (0-0)
Netherlands	Suboptimal diet	62.4 (58.7-66.3)	60 (55.4-65.6)	1525 (1424-1638)	1975 (1809-2175)

Table S5. Proportional and absolute burden per 1M population of T2D incidence attributable to suboptimal diet* and each individual dietary factor† at the national level in 1990 and 2018

Country	Dietary factor	Proportional burden, %		Rate, per 1M population‡	
		1990	2018	1990	2018
	Whole grains	10.9 (8.8-13.4)	9.6 (6.9-12.8)	266 (214-326)	313 (225-421)
	Refined grains (total)	6.6 (5.7-8)	9.1 (7.9-11.1)	160 (139-195)	299 (258-366)
	Refined rice	0.6 (0.5-0.7)	1 (0.9-1.2)	14 (12-16)	32 (28-40)
	Refined wheat	6 (5.3-7.4)	8.2 (7.1-10.1)	147 (127-180)	269 (232-331)
	Processed meats	28.9 (23.5-35.5)	29.7 (23-38.2)	705 (571-871)	973 (754-1265)
	Unprocessed red meats	15.4 (13-19)	18.2 (15.7-23)	375 (318-463)	597 (516-767)
	Yogurt	2.3 (1.7-3.3)	2.2 (1.5-3.2)	57 (41-80)	73 (48-107)
	Sugar-sweetened beverages	11.9 (10.1-14.9)	9.6 (8.2-13)	290 (245-364)	315 (265-427)
	Potatoes	12.8 (11.3-16)	3.3 (2.9-4.5)	313 (275-391)	107 (95-145)
	Fruit	2.2 (2.1-2.4)	2.5 (2.3-2.7)	55 (51-59)	82 (75-89)
	Nuts and seeds	1.1 (1-1.1)	1.1 (1-1.2)	26 (24-28)	36 (33-40)
	Non-starchy vegetables	1 (1-1.1)	1.2 (1.1-1.3)	26 (23-28)	39 (35-43)
	Fruit juice	0.2 (0.2-0.2)	0.2 (0.2-0.3)	5 (4-6)	8 (7-9)
New Zealand	Suboptimal diet	69.7 (64.2-75.5)	68.7 (63.6-73.7)	1296 (1184-1414)	2013 (1843-2172)
	Whole grains	14.6 (4.1-26.6)	14.1 (3.8-25.2)	272 (75-488)	411 (111-739)
	Refined grains (total)	4.6 (3.3-6.7)	9.1 (6.5-12.7)	85 (60-124)	267 (190-371)
	Refined rice	0.5 (0.4-0.8)	1.9 (1.3-2.8)	9 (7-14)	54 (38-82)
	Refined wheat	4.1 (2.9-6.1)	7.5 (5.2-10.5)	76 (54-111)	218 (154-309)
	Processed meats	36.9 (29.8-45.4)	38.1 (31-46.3)	685 (547-849)	1113 (902-1364)
	Unprocessed red meats	27.2 (22.9-32.1)	20.6 (17.1-24.9)	505 (424-599)	602 (496-733)
	Yogurt	7.7 (5.7-9.4)	7.3 (5.4-9)	143 (103-174)	214 (158-264)
	Sugar-sweetened beverages	3.9 (3-6.1)	5.6 (4.3-7.8)	71 (55-113)	164 (126-228)
	Potatoes	8.2 (5.7-12.6)	8 (5.4-11.9)	151 (104-233)	232 (158-348)
	Fruit	1.7 (1.4-2)	1.8 (1.5-2.2)	31 (25-38)	53 (43-64)
	Nuts and seeds	1.2 (1.1-1.3)	1.2 (1.1-1.3)	22 (20-24)	35 (32-38)
	Non-starchy vegetables	1 (0.8-1.1)	1 (0.9-1.2)	18 (15-21)	31 (26-35)
	Fruit juice	0.2 (0.1-0.3)	0.2 (0.1-0.3)	3 (2-5)	6 (4-10)
Nicaragua	Suboptimal diet	76.7 (70.2-83.3)	85.1 (78.9-90.5)	2326 (2117-2547)	4269 (3961-4578)
	Whole grains	32.4 (24.6-37.3)	29.3 (20.7-35.9)	982 (746-1131)	1464 (1038-1816)
	Refined grains (total)	12.5 (8.6-17.2)	18.6 (13.1-25.3)	379 (262-525)	936 (660-1267)
	Refined rice	9.6 (6.6-13.4)	14.1 (9.8-19.5)	293 (200-409)	709 (491-985)
	Refined wheat	3.3 (2.2-4.8)	5.6 (3.8-8.1)	100 (67-148)	280 (189-407)
	Processed meats	31.9 (17.3-50.1)	49.5 (32.7-66.9)	967 (526-1532)	2488 (1635-3400)
	Unprocessed red meats	29.8 (22.9-38.3)	15.8 (10.9-22.8)	901 (689-1168)	791 (550-1148)
	Yogurt	12.2 (11.1-13.1)	12.5 (11.2-13.5)	369 (333-398)	625 (557-679)

Table S5. Proportional and absolute burden per 1M population of T2D incidence attributable to suboptimal diet* and each individual dietary factor† at the national level in 1990 and 2018

Country	Dietary factor	Proportional burden, %		Rate, per 1M population‡	
		1990	2018	1990	2018
	Sugar-sweetened beverages	13.1 (9.4-18.1)	36.6 (29.4-45.1)	398 (289-550)	1832 (1467-2265)
	Potatoes	1.4 (0.7-2.8)	1.7 (0.9-3.4)	42 (22-84)	87 (47-172)
	Fruit	2.6 (2.3-2.8)	2.4 (2.1-2.7)	78 (70-86)	120 (103-137)
	Nuts and seeds	1 (0.9-1.2)	1.2 (1.1-1.4)	32 (27-36)	62 (53-69)
	Non-starchy vegetables	1.2 (1.1-1.4)	0.5 (0.3-0.7)	37 (32-42)	26 (18-35)
	Fruit juice	0.2 (0.1-0.3)	0.2 (0.1-0.3)	5 (3-8)	9 (5-16)
Niger	Suboptimal diet	46.7 (39.4-59.4)	64.8 (57.7-72.2)	426 (360-540)	1073 (951-1201)
	Whole grains	19.4 (11.6-27.1)	22.7 (14.6-30.1)	177 (106-249)	378 (239-501)
	Refined grains (total)	12.9 (10-16.6)	32.6 (26.7-39.3)	118 (92-152)	539 (441-655)
	Refined rice	9.2 (7.1-12.2)	30.4 (24.7-36.7)	84 (65-112)	503 (409-613)
	Refined wheat	4.1 (3.1-5.6)	3.2 (2.6-4.1)	37 (29-52)	53 (42-69)
	Processed meats	10 (2.9-30.7)	5 (1.3-18.7)	92 (26-282)	82 (21-312)
	Unprocessed red meats	2.8 (1.7-6.3)	1.1 (0.6-3.5)	25 (15-57)	18 (11-59)
	Yogurt	10.4 (8.7-11.7)	11 (9.4-12.3)	95 (79-107)	182 (155-206)
	Sugar-sweetened beverages	6.3 (3.9-11)	0 (0-0)	58 (35-101)	0 (0-0)
	Potatoes	0.2 (0.1-0.5)	23.3 (14.5-33.2)	2 (1-4)	388 (239-551)
	Fruit	2.8 (2.6-3.1)	3.2 (3-3.5)	26 (24-28)	54 (49-58)
	Nuts and seeds	1.3 (1.2-1.4)	0.9 (0.7-1.1)	12 (10-13)	15 (11-18)
	Non-starchy vegetables	1.2 (1-1.3)	1.4 (1.2-1.5)	11 (10-12)	23 (20-26)
	Fruit juice	0.1 (0.1-0.1)	0.1 (0-0.1)	1 (0-1)	1 (1-2)
Nigeria	Suboptimal diet	50.1 (43.1-62)	58.7 (53.3-65.9)	801 (685-985)	1127 (1013-1272)
	Whole grains	18.9 (11.6-26.1)	21.6 (13.7-29.1)	302 (183-418)	414 (262-558)
	Refined grains (total)	13.5 (10.8-17.2)	13.1 (10.9-16.2)	217 (172-276)	252 (210-309)
	Refined rice	12.6 (10.1-16.1)	9.4 (7.8-11.9)	203 (161-260)	180 (149-228)
	Refined wheat	1 (0.8-1.3)	4.1 (3.4-5.5)	16 (13-22)	79 (65-105)
	Processed meats	12.7 (3.9-33)	7.8 (2.5-20.1)	204 (61-528)	151 (47-385)
	Unprocessed red meats	9.4 (7.2-13.2)	9.7 (7.2-13.6)	151 (114-210)	186 (139-258)
	Yogurt	11.4 (10.4-12.2)	12.1 (11.1-13)	182 (166-197)	233 (213-251)
	Sugar-sweetened beverages	1.7 (1-3.4)	17.1 (11.7-24.6)	28 (17-55)	328 (222-469)
	Potatoes	0.1 (0-0.1)	3.7 (2.1-7.6)	1 (1-2)	71 (40-144)
	Fruit	3.2 (3-3.3)	3.7 (3.6-3.9)	51 (48-54)	72 (68-76)
	Nuts and seeds	1.2 (1.1-1.3)	1 (0.8-1.2)	20 (18-21)	19 (16-23)
	Non-starchy vegetables	1.2 (1.1-1.3)	1.5 (1.4-1.6)	20 (18-21)	28 (26-31)
	Fruit juice	0 (0-0)	0 (0-0.1)	0 (0-1)	1 (1-1)
Norway	Suboptimal diet	64.9 (58.7-72.4)	75.1 (69.3-81.5)	1980 (1779-2214)	2946 (2676-3218)

Table S5. Proportional and absolute burden per 1M population of T2D incidence attributable to suboptimal diet* and each individual dietary factor† at the national level in 1990 and 2018

Country	Dietary factor	Proportional burden, %		Rate, per 1M population‡	
		1990	2018	1990	2018
	Whole grains	10 (7.3-13.1)	25.3 (22.8-27.8)	305 (224-404)	991 (887-1098)
	Refined grains (total)	0.6 (0.4-1)	1.6 (1-2.6)	18 (11-31)	62 (38-103)
	Refined rice	0 (0-0.1)	0.1 (0.1-0.2)	1 (1-2)	5 (3-9)
	Refined wheat	0.5 (0.3-0.9)	1.4 (0.9-2.4)	17 (10-29)	56 (35-95)
	Processed meats	30.2 (19.8-44)	35.2 (22.9-51.9)	922 (604-1340)	1375 (891-2039)
	Unprocessed red meats	27.7 (23.3-32.8)	34.3 (28.9-40.7)	847 (708-1001)	1343 (1125-1601)
	Yogurt	7.5 (5.6-9.1)	7.6 (5.6-9.3)	230 (169-279)	298 (220-368)
	Sugar-sweetened beverages	7.2 (5.7-10.2)	6.9 (5.6-10)	219 (172-312)	270 (219-396)
	Potatoes	4.5 (3.8-6.2)	3.1 (2.7-4.4)	137 (116-191)	122 (104-173)
	Fruit	2.5 (2.3-2.7)	2.8 (2.6-3.1)	77 (71-83)	111 (102-121)
	Nuts and seeds	1.1 (1-1.2)	1.2 (1.1-1.3)	34 (31-37)	45 (41-50)
	Non-starchy vegetables	1.3 (1.2-1.4)	1.1 (0.9-1.2)	40 (37-44)	42 (36-48)
	Fruit juice	0.1 (0.1-0.1)	0.1 (0.1-0.1)	2 (2-3)	3 (2-5)
Oman	Suboptimal diet	67.7 (59.6-76)	80.2 (72.7-87.4)	2127 (1871-2389)	3256 (2915-3595)
	Whole grains	35.5 (27.9-40.4)	30.9 (21.4-38.1)	1114 (878-1280)	1252 (865-1558)
	Refined grains (total)	26.1 (17-37.3)	28.3 (18.6-40.1)	822 (534-1180)	1145 (750-1633)
	Refined rice	16.3 (10.3-24.3)	19 (12-27.9)	514 (325-766)	768 (487-1146)
	Refined wheat	12.5 (7.7-18.9)	12.3 (7.6-18.6)	391 (243-591)	498 (309-765)
	Processed meats	20.5 (9.1-38.3)	36.9 (19.8-56.8)	644 (288-1202)	1499 (811-2323)
	Unprocessed red meats	10.5 (6.3-16.6)	17.9 (11.6-26.3)	328 (196-521)	725 (469-1086)
	Yogurt	8.7 (6-10.9)	8.2 (5.4-10.6)	273 (188-349)	333 (217-436)
	Sugar-sweetened beverages	6.9 (4.3-11.3)	17.5 (11.4-26.5)	218 (135-359)	709 (455-1072)
	Potatoes	3.7 (2-7.1)	8 (4.4-14.8)	117 (64-220)	321 (182-612)
	Fruit	2.7 (2.3-3.2)	2.8 (2.2-3.4)	86 (71-99)	116 (88-139)
	Nuts and seeds	1.5 (1.4-1.6)	1 (0.7-1.2)	46 (43-49)	39 (30-50)
	Non-starchy vegetables	0.9 (0.6-1.2)	1.1 (0.8-1.4)	29 (20-37)	45 (31-58)
	Fruit juice	0.1 (0.1-0.2)	0.1 (0.1-0.2)	4 (2-7)	5 (3-9)
Pakistan	Suboptimal diet	75.4 (70.1-80.7)	74.4 (69-81.1)	1905 (1757-2057)	2881 (2658-3141)
	Whole grains	28.3 (17.3-37)	17.8 (8.1-29.6)	715 (435-931)	686 (316-1143)
	Refined grains (total)	44.7 (38.1-52.4)	41.6 (35.1-49)	1129 (961-1328)	1607 (1355-1910)
	Refined rice	10.1 (8.2-12.8)	9.5 (7.7-12.1)	256 (206-325)	368 (294-469)
	Refined wheat	38.6 (32.6-46)	35.6 (29.7-42.3)	974 (823-1161)	1374 (1148-1652)
	Processed meats	7.5 (1.5-25.7)	8.9 (1.8-29.7)	189 (38-645)	343 (70-1151)
	Unprocessed red meats	10.4 (8-14)	12.6 (9.8-16.5)	262 (204-354)	486 (381-636)
	Yogurt	13.2 (12.3-13.9)	13.6 (12.7-14.4)	332 (309-355)	524 (487-563)

Table S5. Proportional and absolute burden per 1M population of T2D incidence attributable to suboptimal diet* and each individual dietary factor† at the national level in 1990 and 2018

Country	Dietary factor	Proportional burden, %		Rate, per 1M population‡	
		1990	2018	1990	2018
	Sugar-sweetened beverages	19.3 (11.6-29.2)	18.1 (11-27.1)	489 (294-731)	700 (421-1045)
	Potatoes	1.3 (0.6-3.2)	5.3 (2.4-11.2)	33 (16-79)	203 (94-433)
	Fruit	3.6 (3.4-3.7)	4.1 (3.9-4.3)	90 (85-95)	160 (151-169)
	Nuts and seeds	1.2 (1-1.4)	1.4 (1.1-1.5)	31 (25-35)	53 (44-60)
	Non-starchy vegetables	1.4 (1.3-1.5)	1.7 (1.5-1.8)	36 (33-38)	64 (58-69)
	Fruit juice	0 (0-0)	0 (0-0)	0 (0-0)	0 (0-0)
Palestine	Suboptimal diet	69.3 (61.5-77.2)	76 (67.6-83.4)	2178 (1923-2433)	4437 (3926-4902)
	Whole grains	33.8 (26-38.6)	34.6 (26.6-39.4)	1063 (813-1219)	2021 (1555-2315)
	Refined grains (total)	26.5 (16.1-38.6)	31.4 (19.5-43.7)	832 (502-1223)	1832 (1140-2561)
	Refined rice	26.5 (16.1-38.6)	31.4 (19.5-43.7)	832 (502-1223)	1832 (1140-2561)
	Refined wheat	0 (0-0)	0 (0-0)	0 (0-0)	0 (0-0)
	Processed meats	19.7 (9.2-36.8)	25.8 (10.9-45.7)	620 (285-1146)	1517 (640-2676)
	Unprocessed red meats	13.9 (9-20.5)	15 (9.7-22.4)	438 (280-649)	881 (565-1313)
	Yogurt	7.7 (5.3-9.8)	7.7 (5-10)	243 (164-308)	454 (292-589)
	Sugar-sweetened beverages	11.2 (7.5-16.5)	15.2 (10.4-21.8)	353 (240-518)	893 (609-1271)
	Potatoes	3.3 (1.8-5.9)	5.8 (3.2-10.3)	104 (57-185)	338 (186-601)
	Fruit	2.5 (2-3)	2.7 (2-3.3)	80 (62-94)	158 (116-193)
	Nuts and seeds	1.1 (0.9-1.2)	0.9 (0.6-1.1)	35 (30-39)	50 (37-63)
	Non-starchy vegetables	0.8 (0.5-1.2)	1 (0.6-1.4)	26 (16-36)	58 (35-80)
	Fruit juice	0.1 (0.1-0.2)	0.1 (0.1-0.3)	4 (2-7)	8 (4-15)
Panama	Suboptimal diet	82.8 (77-89.1)	87.6 (83-92)	2698 (2497-2908)	4830 (4545-5129)
	Whole grains	28.8 (21.2-34.4)	27.5 (19.2-33.9)	936 (694-1117)	1513 (1049-1865)
	Refined grains (total)	16.8 (11.8-22.8)	23.5 (17.2-31.3)	548 (384-741)	1293 (936-1731)
	Refined rice	12.7 (8.7-17.5)	18.3 (13.3-24.7)	414 (285-570)	1004 (723-1365)
	Refined wheat	5 (3.4-7.1)	6.8 (4.8-9.6)	164 (110-231)	375 (262-529)
	Processed meats	41.2 (23.7-60.9)	46.4 (29.5-63.5)	1339 (763-1985)	2554 (1638-3517)
	Unprocessed red meats	27.4 (20.7-34.9)	28.8 (21.7-36.6)	891 (673-1137)	1582 (1195-2013)
	Yogurt	11.2 (9.9-12.2)	11.5 (10.1-12.6)	366 (323-398)	635 (555-700)
	Sugar-sweetened beverages	31.1 (24.9-38.3)	38.6 (31.3-47.1)	1015 (812-1247)	2125 (1717-2603)
	Potatoes	0.4 (0.2-0.7)	2.7 (1.5-5.3)	12 (6-22)	150 (79-291)
	Fruit	2.4 (2.1-2.6)	2.7 (2.4-3)	77 (69-85)	147 (131-164)
	Nuts and seeds	1.3 (1.2-1.4)	0.9 (0.8-1.1)	43 (40-46)	52 (42-61)
	Non-starchy vegetables	1.4 (1.2-1.5)	1.2 (1-1.4)	45 (40-49)	65 (54-75)
	Fruit juice	0.1 (0.1-0.2)	0.2 (0.1-0.3)	5 (3-8)	9 (5-15)
Papua New Guinea	Suboptimal diet	72.1 (65.2-79.5)	82.3 (76.6-86.8)	2880 (2577-3182)	6146 (5678-6598)

Table S5. Proportional and absolute burden per 1M population of T2D incidence attributable to suboptimal diet* and each individual dietary factor† at the national level in 1990 and 2018

Country	Dietary factor	Proportional burden, %		Rate, per 1M population‡	
		1990	2018	1990	2018
	Whole grains	39.6 (34.5-42.9)	12.7 (6.4-21.2)	1579 (1351-1731)	945 (480-1580)
	Refined grains (total)	35.5 (22.2-49.7)	22.1 (14-32.6)	1417 (889-1982)	1651 (1053-2451)
	Refined rice	35.5 (22.2-49.7)	13.6 (8.4-20.8)	1417 (889-1982)	1020 (631-1559)
	Refined wheat	0 (0-0)	10.5 (6.4-16.1)	0 (0-0)	780 (480-1199)
	Processed meats	10.5 (4.4-22.4)	6.5 (1.6-19.6)	418 (177-895)	484 (117-1471)
	Unprocessed red meats	19.3 (13.1-26.9)	66 (57.4-72.7)	771 (519-1077)	4930 (4249-5477)
	Yogurt	14 (12.8-15.1)	14.4 (13.2-15.6)	557 (509-609)	1074 (979-1182)
	Sugar-sweetened beverages	4.2 (2.7-8.3)	0 (0-0)	168 (108-331)	0 (0-0)
	Potatoes	0.2 (0.1-0.4)	0.1 (0-0.1)	8 (4-17)	5 (3-11)
	Fruit	3.6 (3.3-3.9)	4.9 (4.6-5.2)	144 (132-156)	367 (343-393)
	Nuts and seeds	1.3 (1.2-1.5)	1.7 (1.5-1.8)	54 (48-59)	126 (115-139)
	Non-starchy vegetables	1.6 (1.4-1.7)	1.8 (1.6-2)	63 (56-70)	137 (121-154)
	Fruit juice	0 (0-0.1)	0.2 (0.1-0.4)	1 (1-3)	14 (6-34)
Paraguay	Suboptimal diet	83 (76.6-89.3)	85.5 (80.6-90.3)	1826 (1683-1977)	3255 (3046-3463)
	Whole grains	32.3 (25.6-36.6)	27.5 (19.5-33.6)	711 (562-809)	1048 (741-1282)
	Refined grains (total)	32.6 (24.8-40.1)	12.3 (8.4-17)	718 (547-880)	469 (323-649)
	Refined rice	16.1 (11.8-20.5)	2.3 (1.5-3.4)	355 (260-454)	86 (57-132)
	Refined wheat	20.7 (15.3-26.1)	10.3 (7.1-14.5)	456 (338-576)	395 (270-549)
	Processed meats	37.3 (19.3-59.8)	32 (16.8-51.3)	822 (424-1314)	1216 (635-1947)
	Unprocessed red meats	28.6 (21.5-36.9)	46.3 (37.1-55.3)	630 (470-809)	1761 (1404-2122)
	Yogurt	12.1 (11.1-12.8)	12.2 (11.2-13)	265 (243-283)	463 (423-497)
	Sugar-sweetened beverages	17.7 (13.2-23.4)	28.1 (21.4-36.2)	389 (288-516)	1071 (815-1384)
	Potatoes	0.1 (0.1-0.2)	6.7 (3.5-12)	2 (1-5)	253 (133-461)
	Fruit	1.8 (1.6-2.1)	3 (2.8-3.2)	41 (35-47)	115 (105-125)
	Nuts and seeds	1.1 (1-1.2)	1.2 (1-1.3)	24 (21-26)	44 (38-50)
	Non-starchy vegetables	1.5 (1.4-1.6)	1.8 (1.6-1.9)	32 (30-35)	67 (62-72)
	Fruit juice	0.1 (0.1-0.2)	0.2 (0.1-0.3)	3 (2-4)	6 (4-10)
Peru	Suboptimal diet	78.5 (73.5-84.3)	82.1 (77.1-87.4)	1228 (1136-1324)	2362 (2200-2528)
	Whole grains	25.3 (17.8-31.9)	25.7 (17.7-32.3)	396 (276-498)	739 (508-930)
	Refined grains (total)	20.2 (17.4-23.5)	20 (17.3-23.4)	316 (272-368)	576 (492-674)
	Refined rice	13.7 (11.8-16.2)	13.2 (11.4-15.9)	215 (184-252)	380 (323-457)
	Refined wheat	7.5 (6.4-9)	7.8 (6.7-9.5)	117 (99-141)	223 (189-276)
	Processed meats	31 (17.3-47.2)	33.4 (18.8-52)	483 (268-739)	965 (537-1491)
	Unprocessed red meats	30.4 (26.4-34.8)	39.8 (35.7-44.5)	475 (411-547)	1146 (1025-1280)
	Yogurt	11.9 (11.2-12.6)	12.1 (11.3-12.8)	186 (174-198)	346 (323-373)

Table S5. Proportional and absolute burden per 1M population of T2D incidence attributable to suboptimal diet* and each individual dietary factor† at the national level in 1990 and 2018

Country	Dietary factor	Proportional burden, %		Rate, per 1M population‡	
		1990	2018	1990	2018
	Sugar-sweetened beverages	21.1 (14.7-30.1)	20.5 (14.3-29.4)	329 (229-471)	586 (408-856)
	Potatoes	1.1 (0.6-2.4)	1.5 (0.8-3.3)	17 (9-36)	43 (22-95)
	Fruit	2.9 (2.8-3.1)	1.5 (1.2-1.7)	46 (43-48)	42 (34-49)
	Nuts and seeds	1.3 (1.2-1.3)	1.1 (0.9-1.3)	20 (18-21)	32 (27-36)
	Non-starchy vegetables	1.3 (1.2-1.4)	1.5 (1.4-1.6)	20 (19-22)	44 (40-48)
	Fruit juice	0.1 (0.1-0.1)	0.1 (0.1-0.1)	2 (1-2)	3 (2-3)
Philippines	Suboptimal diet	55.8 (53.1-58.9)	66.6 (63.7-69.7)	1378 (1300-1463)	2269 (2154-2392)
	Whole grains	16.1 (12.2-20.3)	16.4 (12.1-20.5)	399 (299-502)	558 (414-702)
	Refined grains (total)	18.9 (16.9-21.4)	24.8 (22.3-27.9)	465 (414-526)	846 (758-958)
	Refined rice	17.2 (15.3-19.5)	22.2 (19.9-25.2)	424 (376-480)	758 (678-863)
	Refined wheat	2 (1.8-2.7)	3.3 (2.9-4)	50 (44-66)	113 (100-139)
	Processed meats	12.4 (9.5-15.9)	27.8 (22.6-33.2)	307 (236-391)	946 (769-1141)
	Unprocessed red meats	7.6 (6-10.2)	8.1 (6.4-10.7)	189 (148-252)	277 (220-367)
	Yogurt	12.4 (11.8-13)	12.7 (12-13.3)	307 (290-323)	433 (409-456)
	Sugar-sweetened beverages	11.2 (9.2-13.7)	9.9 (8.2-12.6)	275 (225-337)	339 (276-428)
	Potatoes	0.1 (0.1-0.1)	0.2 (0.2-0.3)	2 (1-3)	7 (5-9)
	Fruit	2.5 (2.4-2.6)	3.1 (3-3.3)	62 (59-66)	107 (101-113)
	Nuts and seeds	1.3 (1.2-1.3)	1.5 (1.4-1.5)	31 (30-33)	50 (46-53)
	Non-starchy vegetables	1.2 (1.1-1.3)	1.4 (1.3-1.5)	30 (28-32)	47 (44-51)
	Fruit juice	0.1 (0-0.1)	0.1 (0.1-0.1)	2 (1-2)	2 (2-3)
Poland	Suboptimal diet	88.4 (86.2-90.4)	89 (87.2-91)	2998 (2878-3126)	4185 (4057-4337)
	Whole grains	31.6 (29.6-33.6)	31.5 (29.6-33.3)	1073 (1000-1155)	1482 (1380-1574)
	Refined grains (total)	21 (18.1-24.1)	22.3 (19.7-25.7)	712 (614-828)	1050 (926-1217)
	Refined rice	0.5 (0.4-0.6)	0.7 (0.6-0.8)	17 (15-21)	31 (27-38)
	Refined wheat	20.6 (17.8-23.7)	21.8 (19.3-25.2)	698 (602-812)	1026 (904-1189)
	Processed meats	54 (46.1-61.4)	65.3 (60.4-70.6)	1838 (1568-2096)	3062 (2823-3332)
	Unprocessed red meats	36 (32.5-40.3)	28.1 (25.2-32)	1222 (1093-1382)	1319 (1189-1515)
	Yogurt	9 (8.2-9.8)	8.8 (8-9.6)	307 (275-337)	414 (376-452)
	Sugar-sweetened beverages	4.9 (4-7.3)	4.7 (3.9-7)	166 (135-244)	222 (182-327)
	Potatoes	26.9 (23.9-30.8)	18 (15.9-21.3)	912 (814-1049)	846 (745-1008)
	Fruit	1.6 (1.4-1.8)	1.3 (1.1-1.5)	55 (49-62)	61 (52-70)
	Nuts and seeds	1.4 (1.3-1.5)	1.3 (1.2-1.4)	46 (43-50)	62 (57-66)
	Non-starchy vegetables	0.7 (0.7-0.8)	0.8 (0.7-0.9)	25 (22-28)	38 (33-42)
	Fruit juice	0.2 (0.2-0.3)	0.2 (0.2-0.2)	8 (7-9)	9 (8-10)
Portugal	Suboptimal diet	67.5 (65.1-70.2)	68.3 (65.5-71.7)	2634 (2501-2769)	4273 (4061-4504)

Table S5. Proportional and absolute burden per 1M population of T2D incidence attributable to suboptimal diet* and each individual dietary factor† at the national level in 1990 and 2018

Country	Dietary factor	Proportional burden, %		Rate, per 1M population‡	
		1990	2018	1990	2018
	Whole grains	26.9 (25.2-28.5)	26.4 (25-28.2)	1046 (975-1116)	1655 (1547-1778)
	Refined grains (total)	12.9 (11.4-14.8)	12.8 (11.4-14.8)	502 (445-580)	799 (713-930)
	Refined rice	3.1 (2.7-3.8)	2.9 (2.5-3.5)	121 (107-149)	180 (159-220)
	Refined wheat	10 (8.9-11.6)	10.2 (9.1-11.8)	392 (347-456)	636 (566-740)
	Processed meats	10.2 (7.3-14.6)	22.3 (18-29)	396 (282-577)	1393 (1113-1827)
	Unprocessed red meats	21.5 (18.8-25.3)	26.4 (23.4-30.4)	837 (728-992)	1651 (1447-1897)
	Yogurt	6.2 (5.4-7)	5.9 (5.1-6.8)	241 (208-273)	367 (317-423)
	Sugar-sweetened beverages	8.1 (7-10.7)	8.9 (7.5-11.8)	316 (271-416)	556 (471-742)
	Potatoes	23.3 (20.8-27.3)	9 (8-11.4)	911 (803-1071)	561 (502-705)
	Fruit	2.2 (2-2.3)	0.3 (0.2-0.5)	86 (78-93)	22 (15-30)
	Nuts and seeds	1.2 (1.1-1.2)	1.2 (1.1-1.3)	46 (42-49)	75 (69-82)
	Non-starchy vegetables	0.8 (0.7-0.9)	0.9 (0.8-1)	31 (27-34)	56 (50-63)
	Fruit juice	0 (0-0)	0 (0-0)	1 (1-1)	2 (2-2)
Qatar	Suboptimal diet	75.6 (66.2-83.7)	80.1 (71-87.7)	3162 (2737-3559)	7788 (6832-8715)
	Whole grains	35.8 (27.2-40.7)	36.5 (27.3-41.9)	1499 (1125-1741)	3558 (2583-4119)
	Refined grains (total)	28.3 (16.5-42.3)	32.2 (19.3-46.1)	1185 (690-1768)	3123 (1855-4561)
	Refined rice	28.3 (16.5-42.3)	32.2 (19.3-46.1)	1185 (690-1768)	3123 (1855-4561)
	Refined wheat	0 (0-0)	0 (0-0)	0 (0-0)	0 (0-0)
	Processed meats	24.7 (10.4-46.8)	31.5 (13.3-55.4)	1033 (435-1945)	3070 (1304-5490)
	Unprocessed red meats	14.5 (8.7-23.2)	14.2 (8.2-24.4)	606 (367-978)	1380 (789-2419)
	Yogurt	8.2 (5.4-10.8)	8.2 (5.1-11)	344 (224-457)	805 (502-1082)
	Sugar-sweetened beverages	19.2 (12.8-29.4)	22 (14.4-33.7)	803 (529-1223)	2149 (1429-3310)
	Potatoes	3 (1.7-6.3)	5 (2.5-10.3)	126 (71-259)	483 (249-1006)
	Fruit	2.7 (2.1-3.2)	2.9 (2.1-3.5)	111 (85-134)	279 (204-347)
	Nuts and seeds	1.2 (1-1.4)	0.9 (0.7-1.2)	49 (40-58)	91 (64-121)
	Non-starchy vegetables	0.9 (0.5-1.3)	1.1 (0.6-1.5)	38 (22-54)	104 (60-146)
	Fruit juice	0.1 (0.1-0.2)	0.1 (0.1-0.3)	5 (3-10)	14 (7-27)
Romania	Suboptimal diet	95.2 (93.2-96.9)	88.4 (85.7-90.8)	2140 (2058-2226)	2860 (2746-2985)
	Whole grains	38.8 (37.4-40.2)	38.6 (37.1-39.9)	872 (826-916)	1249 (1187-1307)
	Refined grains (total)	30.5 (25.4-36.5)	29.8 (25-35.5)	686 (567-815)	965 (815-1158)
	Refined rice	2 (1.6-2.7)	1.3 (1.1-1.7)	44 (36-60)	42 (34-56)
	Refined wheat	29.1 (24.2-34.9)	28.9 (24.2-34.5)	654 (540-780)	935 (788-1123)
	Processed meats	74.1 (64.8-82.4)	57.2 (49.2-64.7)	1669 (1447-1858)	1850 (1594-2104)
	Unprocessed red meats	54.3 (48.5-60.9)	28 (23.3-33.3)	1223 (1083-1373)	904 (754-1082)
	Yogurt	7 (5.6-8.3)	6.8 (5.4-8.1)	157 (125-187)	221 (172-263)

Table S5. Proportional and absolute burden per 1M population of T2D incidence attributable to suboptimal diet* and each individual dietary factor† at the national level in 1990 and 2018

Country	Dietary factor	Proportional burden, %		Rate, per 1M population‡	
		1990	2018	1990	2018
	Sugar-sweetened beverages	12.6 (10.1-15.8)	12.1 (9.6-15.7)	284 (225-354)	393 (311-510)
	Potatoes	6.1 (5.1-8)	13.9 (11.7-16.8)	137 (115-179)	449 (379-550)
	Fruit	1.5 (1.2-1.9)	1.7 (1.3-2)	35 (28-42)	54 (42-66)
	Nuts and seeds	1.2 (1.1-1.2)	1 (0.9-1.1)	26 (24-28)	33 (29-37)
	Non-starchy vegetables	0.1 (0.1-0.2)	0.1 (0.1-0.2)	3 (2-4)	4 (2-6)
	Fruit juice	0 (0-0.1)	0 (0-0.1)	1 (1-1)	1 (1-2)
Russia	Suboptimal diet	88 (83.7-91.1)	88.7 (84.5-92.3)	1474 (1384-1553)	2220 (2092-2349)
	Whole grains	7.3 (4.7-10.9)	9.6 (6.3-13.3)	122 (79-182)	241 (156-331)
	Refined grains (total)	7.3 (4.6-11.9)	4.9 (3.1-8)	122 (78-201)	122 (76-199)
	Refined rice	0.7 (0.5-1.2)	0.3 (0.2-0.5)	12 (8-21)	8 (5-13)
	Refined wheat	6.6 (4.1-10.9)	4.6 (2.9-7.5)	111 (70-182)	115 (72-187)
	Processed meats	45.3 (34.4-56.5)	58 (47-68.3)	761 (571-948)	1451 (1170-1732)
	Unprocessed red meats	67.3 (60.1-72.9)	59.6 (51.6-67.3)	1127 (997-1230)	1493 (1274-1700)
	Yogurt	6.5 (4.2-8.6)	6 (3.9-8)	108 (71-144)	151 (98-201)
	Sugar-sweetened beverages	4 (3.1-6.7)	8.3 (6.3-11.5)	67 (51-112)	207 (158-288)
	Potatoes	9.9 (8-13)	8.5 (6.9-11.4)	166 (133-216)	214 (171-285)
	Fruit	2.9 (2.7-3.1)	2.9 (2.7-3.1)	49 (46-52)	73 (68-79)
	Nuts and seeds	1.2 (1.1-1.3)	0.9 (0.7-1)	20 (18-22)	22 (18-26)
	Non-starchy vegetables	1.2 (1.1-1.3)	0.7 (0.6-0.9)	20 (18-22)	18 (15-22)
	Fruit juice	0.2 (0.1-0.3)	0.1 (0.1-0.2)	3 (2-4)	4 (2-6)
Rwanda	Suboptimal diet	41 (36-46.9)	86 (82.2-88.7)	788 (691-901)	1810 (1716-1899)
	Whole grains	21.9 (13.7-29.8)	30.5 (23.2-36)	421 (262-571)	644 (487-761)
	Refined grains (total)	12.4 (9.8-16.1)	12 (9.8-15.1)	238 (187-307)	254 (207-320)
	Refined rice	5.9 (4.7-8.2)	7.1 (5.7-9.1)	114 (89-158)	149 (120-193)
	Refined wheat	6.8 (5.4-9.2)	5.3 (4.3-6.9)	131 (102-175)	112 (90-146)
	Processed meats	1.7 (0.5-6.6)	2.2 (0.6-7.9)	32 (9-125)	46 (13-167)
	Unprocessed red meats	0.6 (0.4-3.6)	1.5 (0.9-4.1)	12 (7-68)	32 (19-85)
	Yogurt	12.9 (11.8-13.8)	13 (12.1-13.9)	246 (225-268)	274 (252-295)
	Sugar-sweetened beverages	4.8 (2.7-9.3)	73.9 (67.7-78.2)	92 (51-180)	1557 (1416-1663)
	Potatoes	0.5 (0.3-1)	4.4 (2.5-8.9)	10 (6-20)	94 (51-187)
	Fruit	2.2 (2-2.5)	2.5 (2.3-2.8)	43 (38-48)	53 (47-59)
	Nuts and seeds	1.2 (1-1.3)	1.1 (0.9-1.3)	23 (20-26)	24 (20-28)
	Non-starchy vegetables	0.2 (0.1-0.3)	0.3 (0.2-0.4)	4 (3-6)	6 (4-8)
	Fruit juice	0.1 (0-0.1)	0.1 (0.1-0.1)	1 (1-2)	2 (1-3)
Saint Lucia	Suboptimal diet	67.1 (62.4-72)	67.1 (61.6-73.1)	3880 (3585-4193)	5696 (5220-6261)

Table S5. Proportional and absolute burden per 1M population of T2D incidence attributable to suboptimal diet* and each individual dietary factor† at the national level in 1990 and 2018

Country	Dietary factor	Proportional burden, %		Rate, per 1M population‡	
		1990	2018	1990	2018
	Whole grains	34 (27.8-37.7)	23.2 (14.6-30.2)	1962 (1615-2192)	1966 (1236-2589)
	Refined grains (total)	9.3 (6.3-13.1)	12.2 (8.5-17.6)	538 (362-755)	1036 (715-1498)
	Refined rice	0.8 (0.6-1.4)	1.7 (1.1-2.7)	49 (33-81)	141 (95-224)
	Refined wheat	8.6 (5.7-12.1)	10.8 (7.5-15.7)	495 (332-697)	915 (629-1333)
	Processed meats	7.4 (3.2-16.2)	11.2 (5.2-23.4)	427 (182-945)	954 (439-2003)
	Unprocessed red meats	10.8 (7.2-15.5)	14.9 (10.5-21.3)	626 (418-890)	1265 (889-1825)
	Yogurt	11.3 (10-12.4)	11.8 (10.5-12.9)	657 (576-720)	1005 (891-1100)
	Sugar-sweetened beverages	28.3 (22.3-36.1)	27.8 (21.5-35.4)	1639 (1293-2086)	2366 (1842-3043)
	Potatoes	0.9 (0.5-1.7)	1.7 (0.9-3.2)	49 (27-96)	142 (74-270)
	Fruit	2 (1.4-2.6)	3.3 (2.8-3.7)	118 (80-148)	279 (238-313)
	Nuts and seeds	1.3 (1.2-1.4)	1.5 (1.4-1.6)	74 (69-80)	124 (114-135)
	Non-starchy vegetables	1.4 (1.2-1.6)	1.6 (1.3-1.8)	83 (71-94)	135 (111-157)
	Fruit juice	0.1 (0.1-0.2)	0.1 (0.1-0.2)	6 (3-10)	11 (7-20)
Saint Vincent and the Grenadines	Suboptimal diet	61.5 (56.4-67.9)	88.2 (84.6-91.4)	3274 (2973-3617)	7448 (7076-7811)
	Whole grains	13.7 (7.8-21.2)	32 (25.1-37)	731 (421-1131)	2701 (2111-3114)
	Refined grains (total)	11.8 (8.3-16.4)	15.4 (10.9-21.4)	631 (441-876)	1303 (915-1826)
	Refined rice	5.9 (4-8.4)	5.3 (3.6-7.8)	314 (217-450)	448 (307-654)
	Refined wheat	6.5 (4.4-9.2)	10.9 (7.5-15.4)	346 (239-490)	919 (636-1313)
	Processed meats	11.6 (5.6-23.1)	21.9 (11.8-38.4)	617 (297-1230)	1852 (995-3211)
	Unprocessed red meats	11.9 (8.3-16.7)	22.3 (16.2-29.8)	631 (436-889)	1885 (1367-2528)
	Yogurt	11.2 (10.1-12.1)	11.7 (10.5-12.6)	598 (534-648)	987 (879-1078)
	Sugar-sweetened beverages	26.2 (20.7-32.9)	65.3 (57.9-71.4)	1393 (1094-1755)	5497 (4859-6065)
	Potatoes	0.8 (0.4-1.5)	1.4 (0.7-2.5)	44 (23-84)	115 (60-213)
	Fruit	3 (2.6-3.3)	2.4 (1.6-3)	159 (139-175)	204 (137-254)
	Nuts and seeds	1.3 (1.2-1.4)	1.2 (1-1.3)	70 (66-74)	99 (85-110)
	Non-starchy vegetables	1.6 (1.5-1.8)	1.5 (1.2-1.7)	87 (77-94)	126 (99-147)
	Fruit juice	0.1 (0.1-0.2)	0.1 (0.1-0.2)	7 (4-12)	11 (6-18)
Samoa	Suboptimal diet	69.1 (61.2-76.1)	73.4 (68.4-79.5)	3188 (2812-3536)	6096 (5630-6636)
	Whole grains	5.9 (3.3-10.7)	39.2 (34.3-42.1)	273 (150-488)	3246 (2830-3533)
	Refined grains (total)	34.2 (23.1-45.7)	19.3 (11.8-28.3)	1575 (1071-2111)	1599 (974-2339)
	Refined rice	18.8 (12-26.7)	3.5 (2-5.7)	868 (552-1222)	290 (166-466)
	Refined wheat	20.6 (13.2-29)	16.5 (10-24.6)	951 (608-1334)	1373 (828-2041)
	Processed meats	10.2 (4.6-21.1)	18.1 (9.5-32.4)	468 (212-969)	1504 (787-2682)
	Unprocessed red meats	27.3 (20.5-35.2)	12.8 (8.4-18.3)	1263 (941-1629)	1065 (706-1544)
	Yogurt	13.2 (12.4-14.1)	13.5 (12.5-14.4)	611 (565-655)	1119 (1033-1207)

Table S5. Proportional and absolute burden per 1M population of T2D incidence attributable to suboptimal diet* and each individual dietary factor† at the national level in 1990 and 2018

Country	Dietary factor	Proportional burden, %		Rate, per 1M population‡	
		1990	2018	1990	2018
	Sugar-sweetened beverages	9 (5.7-14.1)	25.8 (17.2-36.2)	415 (264-658)	2143 (1448-3001)
	Potatoes	0.7 (0.3-1.5)	1.2 (0.6-2.7)	30 (16-67)	99 (51-229)
	Fruit	2.5 (2.1-2.9)	2.6 (2.1-3)	117 (97-132)	219 (175-254)
	Nuts and seeds	1.3 (1.2-1.4)	0.8 (0.6-1)	61 (56-66)	67 (52-84)
	Non-starchy vegetables	0.1 (0.1-0.3)	0.2 (0.1-0.3)	6 (3-12)	14 (7-27)
	Fruit juice	0 (0-0.1)	0 (0-0.1)	1 (1-3)	3 (2-5)
Sao Tome and Principe	Suboptimal diet	50 (43.5-59.7)	67.6 (60.5-77.3)	1143 (984-1364)	2034 (1813-2345)
	Whole grains	22.2 (14.7-28.9)	31.8 (24.7-36.9)	508 (331-660)	953 (745-1114)
	Refined grains (total)	17.5 (13.7-22.1)	23.4 (18.8-28.8)	398 (311-506)	704 (559-862)
	Refined rice	8.7 (6.7-11.2)	12.8 (10.1-16.1)	198 (152-255)	385 (302-485)
	Refined wheat	9.7 (7.5-12.4)	12.2 (9.6-15.4)	220 (170-282)	366 (289-462)
	Processed meats	10.3 (3.1-25.8)	23 (9.5-44.9)	235 (71-591)	693 (284-1361)
	Unprocessed red meats	0.6 (0.3-2.3)	2.7 (1.6-5.6)	15 (7-53)	81 (47-166)
	Yogurt	8.8 (6.7-10.3)	9.8 (7.8-11.2)	201 (152-234)	293 (236-339)
	Sugar-sweetened beverages	9.6 (6.3-14.1)	17.1 (11.4-24.5)	218 (144-321)	514 (338-736)
	Potatoes	0.2 (0.1-0.4)	2.7 (1.4-5.2)	4 (2-8)	81 (44-157)
	Fruit	2.9 (2.7-3.1)	2.5 (2.1-2.8)	66 (61-71)	74 (62-84)
	Nuts and seeds	1.2 (1.1-1.3)	1.1 (0.9-1.3)	27 (24-29)	33 (26-39)
	Non-starchy vegetables	1.7 (1.6-1.8)	2.1 (1.9-2.2)	38 (36-41)	62 (57-67)
	Fruit juice	0.1 (0-0.1)	0.1 (0-0.1)	2 (1-2)	2 (1-4)
Saudi Arabia	Suboptimal diet	82.6 (73.9-89.9)	89.3 (81.3-94.7)	2720 (2397-2997)	6031 (5496-6513)
	Whole grains	35.4 (27.9-40.6)	32.7 (23.6-39.7)	1168 (916-1343)	2210 (1598-2692)
	Refined grains (total)	18.5 (11.9-27.3)	32.3 (21.5-44.4)	609 (394-908)	2193 (1454-3029)
	Refined rice	5.1 (3.1-8.5)	17.1 (10.8-24.5)	168 (103-278)	1155 (726-1673)
	Refined wheat	14.4 (9-21.8)	19.5 (12.4-27.9)	473 (300-720)	1321 (834-1896)
	Processed meats	53.7 (33-73.3)	62.7 (38.1-80.1)	1772 (1081-2427)	4233 (2584-5482)
	Unprocessed red meats	18.7 (12.4-26.6)	14.4 (9-21.7)	617 (406-875)	978 (610-1468)
	Yogurt	8.2 (5.4-10.5)	8 (5.1-10.6)	269 (179-346)	539 (348-723)
	Sugar-sweetened beverages	15.2 (10.6-22.3)	23.4 (16.4-33.2)	503 (351-734)	1587 (1109-2251)
	Potatoes	1.9 (1.1-3.8)	9.7 (5.3-16.8)	63 (35-121)	654 (362-1134)
	Fruit	2.9 (2.6-3.1)	2.5 (2.1-3)	94 (84-104)	172 (143-202)
	Nuts and seeds	1.3 (1.2-1.4)	0.9 (0.6-1.1)	43 (39-48)	58 (43-74)
	Non-starchy vegetables	0.6 (0.4-0.8)	1.2 (0.9-1.5)	20 (13-27)	83 (63-100)
	Fruit juice	0.1 (0.1-0.2)	0.1 (0.1-0.3)	4 (2-8)	9 (5-18)
Senegal	Suboptimal diet	67.1 (58.1-78.4)	76.5 (68.7-84.3)	1696 (1461-1981)	2685 (2413-2980)

Table S5. Proportional and absolute burden per 1M population of T2D incidence attributable to suboptimal diet* and each individual dietary factor† at the national level in 1990 and 2018

Country	Dietary factor	Proportional burden, %		Rate, per 1M population‡	
		1990	2018	1990	2018
	Whole grains	16.9 (9.5-24.5)	18.7 (10.7-26.6)	425 (242-624)	661 (375-942)
	Refined grains (total)	13.6 (11.3-16.6)	13.6 (11.3-16.6)	344 (284-421)	479 (399-590)
	Refined rice	11.4 (9.4-14)	10.7 (8.9-13.4)	288 (237-355)	377 (312-467)
	Refined wheat	2.5 (2-3.4)	3.2 (2.7-4.3)	63 (52-87)	114 (95-148)
	Processed meats	25.4 (10.7-48.8)	23 (9.5-45.9)	643 (271-1232)	809 (335-1615)
	Unprocessed red meats	2.7 (1.8-5.6)	2.9 (1.9-6)	68 (46-139)	101 (66-210)
	Yogurt	10.8 (9.2-11.9)	11.1 (9.6-12.3)	272 (231-304)	390 (337-434)
	Sugar-sweetened beverages	30 (21.3-39.4)	48.9 (38.7-59.6)	758 (539-1004)	1716 (1342-2101)
	Potatoes	0.8 (0.4-1.6)	2.7 (1.4-5)	21 (11-40)	93 (49-174)
	Fruit	3 (2.9-3.2)	3.5 (3.3-3.7)	76 (71-81)	122 (115-131)
	Nuts and seeds	0.9 (0.7-1)	1.1 (0.9-1.3)	22 (17-26)	39 (33-45)
	Non-starchy vegetables	1.4 (1.3-1.5)	1.7 (1.5-1.8)	35 (32-38)	58 (53-63)
	Fruit juice	0.1 (0-0.1)	0.1 (0.1-0.1)	2 (1-3)	3 (2-4)
Serbia	Suboptimal diet	88.1 (81-93.2)	79.6 (69.8-88.6)	3485 (3201-3718)	4524 (3966-5035)
	Whole grains	27.7 (19.7-33.4)	2.8 (1.8-4.2)	1094 (792-1332)	157 (102-235)
	Refined grains (total)	40.2 (20.3-52.6)	34.5 (18.8-57)	1592 (804-2089)	1958 (1062-3198)
	Refined rice	40.2 (20.3-52.6)	0.6 (0.3-1.3)	1592 (804-2089)	33 (16-76)
	Refined wheat	0 (0-0)	34.1 (18.5-56.5)	0 (0-0)	1935 (1049-3170)
	Processed meats	42.2 (25.7-62.7)	40.1 (24.4-60.5)	1679 (1019-2485)	2279 (1370-3423)
	Unprocessed red meats	35.8 (25.4-49.6)	28.6 (19.9-40.6)	1415 (1000-1963)	1624 (1123-2322)
	Yogurt	6.5 (4.4-8.4)	6.9 (4.7-8.8)	257 (175-335)	390 (263-501)
	Sugar-sweetened beverages	4.9 (2.4-10.1)	3.2 (1.5-6.9)	195 (94-399)	178 (85-395)
	Potatoes	23.3 (12.6-38.5)	11.9 (6.4-22.3)	924 (493-1525)	671 (363-1254)
	Fruit	2 (1.3-2.6)	2.2 (1.3-2.8)	79 (50-102)	123 (75-160)
	Nuts and seeds	1.3 (1.2-1.4)	1.3 (1-1.4)	50 (46-54)	71 (59-78)
	Non-starchy vegetables	0.4 (0.1-0.8)	0.1 (0-0.1)	14 (4-32)	3 (2-6)
	Fruit juice	0.2 (0.1-0.5)	0.2 (0.1-0.4)	7 (3-18)	9 (4-24)
Seychelles	Suboptimal diet	62.5 (58.2-66.9)	68.2 (63.6-72.7)	2214 (2034-2390)	5838 (5403-6316)
	Whole grains	26.2 (17.3-33.3)	27.5 (18.6-34.8)	931 (613-1182)	2360 (1575-3007)
	Refined grains (total)	14.8 (12.2-18)	15.6 (13.1-19.4)	524 (427-641)	1339 (1107-1667)
	Refined rice	14.8 (12.2-18)	15.6 (13.1-19.4)	524 (427-641)	1339 (1107-1667)
	Refined wheat	0 (0-0)	0 (0-0)	0 (0-0)	0 (0-0)
	Processed meats	11 (7.2-16.6)	16.1 (10.9-23.3)	390 (257-585)	1374 (935-2003)
	Unprocessed red meats	6.7 (5-10)	6.7 (4.9-10.9)	238 (175-358)	575 (416-941)
	Yogurt	11.1 (9.8-12)	11.7 (10.4-12.6)	393 (350-429)	1000 (878-1101)

Table S5. Proportional and absolute burden per 1M population of T2D incidence attributable to suboptimal diet* and each individual dietary factor† at the national level in 1990 and 2018

Country	Dietary factor	Proportional burden, %		Rate, per 1M population‡	
		1990	2018	1990	2018
	Sugar-sweetened beverages	25.5 (20.8-31.4)	28 (22.5-35.1)	900 (732-1116)	2398 (1917-3020)
	Potatoes	2.1 (1.8-3)	5.5 (4.4-7.7)	75 (62-108)	468 (378-653)
	Fruit	2.2 (2-2.4)	2.8 (2.6-3.1)	79 (71-87)	241 (218-266)
	Nuts and seeds	1.1 (1-1.2)	1.1 (0.9-1.2)	40 (35-44)	93 (76-108)
	Non-starchy vegetables	0.6 (0.4-0.7)	0.7 (0.5-0.9)	20 (15-24)	59 (45-73)
	Fruit juice	0 (0-0)	0 (0-0.1)	1 (1-2)	3 (2-5)
Sierra Leone	Suboptimal diet	63.6 (51.7-77.4)	80.2 (69.2-89.9)	634 (515-778)	1411 (1212-1576)
	Whole grains	24.9 (17.8-30.9)	20.1 (12.3-27.9)	249 (178-307)	354 (215-491)
	Refined grains (total)	12.1 (9.6-15.2)	12.1 (10-14.8)	121 (95-152)	212 (175-263)
	Refined rice	11.6 (9.2-14.6)	11.4 (9.4-14)	116 (91-146)	200 (165-248)
	Refined wheat	0.6 (0.4-0.7)	0.8 (0.6-1)	6 (4-7)	14 (11-17)
	Processed meats	34.7 (15.2-59.8)	54.9 (30.1-76.4)	347 (154-591)	968 (531-1347)
	Unprocessed red meats	4 (2.6-6.7)	4.3 (2.8-7.4)	40 (26-68)	75 (49-131)
	Yogurt	11.1 (10.2-11.9)	12.4 (11.4-13.2)	111 (101-120)	218 (200-234)
	Sugar-sweetened beverages	8.1 (5.2-12.2)	27.4 (19-37.1)	81 (52-122)	483 (330-647)
	Potatoes	0.8 (0.4-1.6)	1.7 (0.9-3.6)	8 (4-16)	30 (15-63)
	Fruit	2.3 (2.2-2.5)	2.9 (2.7-3.1)	23 (22-25)	50 (47-55)
	Nuts and seeds	1.1 (1-1.2)	1.3 (1.2-1.4)	11 (10-12)	23 (20-25)
	Non-starchy vegetables	1 (0.9-1.1)	1.3 (1.1-1.4)	10 (9-11)	22 (19-24)
	Fruit juice	0 (0-0)	0 (0-0.1)	0 (0-0)	1 (0-1)
Singapore	Suboptimal diet	71.9 (64.5-78.7)	74.5 (68.2-80.4)	2762 (2442-3058)	3013 (2715-3306)
	Whole grains	40.3 (35.7-43.2)	38.8 (34.2-41.6)	1549 (1352-1679)	1567 (1369-1718)
	Refined grains (total)	39.2 (25-53)	35.7 (22.4-50.2)	1497 (959-2035)	1443 (909-2039)
	Refined rice	39.2 (25-53)	35.7 (22.4-50.2)	1497 (959-2035)	1443 (909-2039)
	Refined wheat	0 (0-0)	0 (0-0)	0 (0-0)	0 (0-0)
	Processed meats	10.3 (6.7-17.4)	15.2 (10.4-22.7)	398 (257-665)	616 (417-926)
	Unprocessed red meats	9 (6.3-15)	18.7 (14.7-24.5)	346 (245-581)	757 (597-990)
	Yogurt	13.3 (11.8-14.6)	12.6 (11.3-13.8)	510 (452-563)	510 (454-568)
	Sugar-sweetened beverages	10.4 (7.8-15.6)	9.9 (7.5-15)	399 (296-599)	401 (301-603)
	Potatoes	0.8 (0.4-1.6)	1 (0.5-2.2)	29 (15-60)	39 (21-89)
	Fruit	2.6 (2.3-2.9)	2.8 (2.4-3.1)	100 (86-114)	113 (98-128)
	Nuts and seeds	1.3 (1.2-1.4)	1.4 (1.2-1.5)	51 (46-56)	56 (50-62)
	Non-starchy vegetables	1.1 (0.9-1.3)	1.2 (1-1.4)	42 (34-50)	49 (41-58)
	Fruit juice	0 (0-0.1)	0 (0-0.1)	2 (1-3)	2 (1-3)
Slovakia	Suboptimal diet	72.8 (67.8-78)	81.1 (76.3-85.9)	2057 (1896-2224)	3112 (2906-3298)

Table S5. Proportional and absolute burden per 1M population of T2D incidence attributable to suboptimal diet* and each individual dietary factor† at the national level in 1990 and 2018

Country	Dietary factor	Proportional burden, %		Rate, per 1M population‡	
		1990	2018	1990	2018
	Whole grains	17.2 (14-20.6)	19 (15.5-22.3)	485 (394-585)	731 (596-860)
	Refined grains (total)	7.3 (5.5-9.9)	7.1 (5.4-9.6)	206 (154-279)	274 (205-371)
	Refined rice	0.8 (0.6-1.2)	0.4 (0.3-0.5)	24 (18-35)	14 (11-20)
	Refined wheat	6.5 (4.9-8.8)	6.8 (5.1-9.2)	183 (137-248)	260 (195-354)
	Processed meats	38.5 (29.1-49)	61.9 (53-71.1)	1087 (826-1385)	2371 (2027-2746)
	Unprocessed red meats	22 (17-27.7)	12.9 (9.4-17.4)	625 (473-782)	497 (360-671)
	Yogurt	8.7 (7.1-10.1)	8.6 (6.8-10)	246 (200-287)	329 (261-386)
	Sugar-sweetened beverages	8.5 (6.7-11.4)	11.8 (9.3-15.6)	240 (186-326)	453 (357-596)
	Potatoes	12.4 (10.1-15.4)	6.5 (5.5-8.8)	351 (285-437)	251 (210-335)
	Fruit	3 (2.8-3.2)	3.2 (3-3.3)	85 (79-91)	121 (113-129)
	Nuts and seeds	1.2 (1.1-1.3)	1.3 (1.2-1.4)	34 (32-37)	50 (47-54)
	Non-starchy vegetables	1.3 (1.2-1.4)	1.4 (1.3-1.5)	35 (32-39)	54 (50-59)
	Fruit juice	0.1 (0.1-0.2)	0.1 (0.1-0.2)	3 (2-5)	4 (3-6)
Slovenia	Suboptimal diet	65.3 (57.9-73.5)	77.3 (69.5-84.5)	2101 (1856-2381)	3091 (2772-3407)
	Whole grains	19.4 (12.7-26.1)	22 (15-28.4)	622 (410-845)	877 (596-1135)
	Refined grains (total)	11.6 (7.9-16.4)	16 (11.3-22.2)	373 (256-529)	639 (454-889)
	Refined rice	0.6 (0.4-1)	1.1 (0.8-1.7)	20 (13-32)	43 (30-69)
	Refined wheat	11.1 (7.6-15.6)	15.1 (10.7-21.1)	356 (244-505)	604 (428-842)
	Processed meats	26.7 (15.7-40.6)	45.4 (29.9-61.9)	862 (515-1306)	1813 (1205-2493)
	Unprocessed red meats	18.6 (12-28.7)	21.2 (14.1-32.1)	596 (386-927)	847 (562-1291)
	Yogurt	6.1 (4-8.1)	5.8 (3.7-8)	195 (128-261)	232 (147-319)
	Sugar-sweetened beverages	6.5 (4.8-9.6)	9.4 (7-12.8)	209 (155-312)	377 (281-511)
	Potatoes	9.7 (6-16)	9.7 (6.1-15.7)	312 (193-516)	387 (242-628)
	Fruit	2.8 (2.6-3)	3.1 (2.9-3.3)	90 (83-96)	126 (117-135)
	Nuts and seeds	1.1 (1-1.3)	1.1 (0.9-1.2)	37 (32-40)	43 (34-50)
	Non-starchy vegetables	1.1 (1-1.3)	1.3 (1.1-1.4)	37 (33-41)	51 (45-57)
	Fruit juice	0.3 (0.2-0.7)	0.3 (0.1-0.7)	11 (5-24)	12 (5-27)
Solomon Islands	Suboptimal diet	74.4 (67.2-81.9)	80.2 (73.6-85.6)	3143 (2829-3471)	5801 (5318-6246)
	Whole grains	29.1 (20.6-35.8)	40.4 (34.8-43.5)	1229 (868-1512)	2920 (2502-3173)
	Refined grains (total)	25.2 (16.5-35)	33.3 (22.6-44.9)	1068 (695-1484)	2403 (1620-3250)
	Refined rice	23 (14.9-32.1)	29 (19.4-39.7)	970 (628-1363)	2093 (1391-2884)
	Refined wheat	3.4 (2.1-5.3)	6.9 (4.3-10.3)	142 (90-220)	501 (311-749)
	Processed meats	21.5 (8.4-41.4)	22.9 (11.2-41.3)	906 (366-1734)	1663 (819-3007)
	Unprocessed red meats	20.4 (14.2-27.6)	23.6 (16.9-31)	861 (598-1169)	1702 (1218-2259)
	Yogurt	13.8 (12.8-14.8)	14.2 (13.2-15.3)	583 (537-631)	1028 (947-1111)

Table S5. Proportional and absolute burden per 1M population of T2D incidence attributable to suboptimal diet* and each individual dietary factor† at the national level in 1990 and 2018

Country	Dietary factor	Proportional burden, %		Rate, per 1M population‡	
		1990	2018	1990	2018
	Sugar-sweetened beverages	18.9 (12.3-27.1)	16.3 (10.4-24.4)	799 (522-1155)	1180 (753-1776)
	Potatoes	0.3 (0.1-0.7)	1.9 (0.9-4.2)	13 (6-29)	135 (66-312)
	Fruit	3.6 (3.4-3.9)	4 (3.7-4.3)	152 (141-165)	291 (266-315)
	Nuts and seeds	1.4 (1.3-1.5)	1.6 (1.5-1.7)	60 (54-65)	117 (106-126)
	Non-starchy vegetables	0.9 (0.5-1.2)	1 (0.6-1.3)	36 (22-49)	71 (43-95)
	Fruit juice	0 (0-0.1)	0.1 (0-0.1)	2 (1-4)	4 (3-8)
South Africa	Suboptimal diet	83.1 (80.1-85.8)	85.1 (82.2-87.9)	2398 (2290-2503)	3852 (3682-4016)
	Whole grains	13.1 (8.9-17.4)	14.8 (9.9-19.2)	379 (259-507)	668 (448-870)
	Refined grains (total)	12.1 (9.6-15.5)	17.4 (14-22.2)	350 (278-452)	788 (633-1012)
	Refined rice	2.7 (2.1-3.7)	6.2 (4.9-8.3)	79 (61-108)	281 (221-376)
	Refined wheat	9.6 (7.7-12.4)	11.9 (9.5-15.4)	279 (221-361)	539 (431-698)
	Processed meats	18.5 (13.1-26)	20.2 (13.6-29.4)	535 (373-756)	914 (615-1323)
	Unprocessed red meats	52.8 (47.9-57.5)	56.3 (51.2-61.2)	1523 (1384-1663)	2545 (2307-2783)
	Yogurt	10.5 (9.1-11.7)	10.4 (8.7-11.8)	303 (260-339)	469 (395-536)
	Sugar-sweetened beverages	33.8 (28.2-40.7)	29 (23.6-36.1)	976 (817-1186)	1311 (1065-1631)
	Potatoes	3.2 (1.8-5.9)	4.3 (2.4-8.1)	92 (52-170)	194 (110-370)
	Fruit	2.9 (2.7-3)	3.8 (3.7-4)	83 (79-88)	174 (164-184)
	Nuts and seeds	1.3 (1.2-1.4)	1.4 (1.3-1.5)	38 (36-40)	66 (61-71)
	Non-starchy vegetables	1 (1-1.1)	1.2 (1.1-1.4)	30 (28-33)	56 (51-62)
	Fruit juice	0 (0-0)	0 (0-0)	0 (0-0)	1 (0-1)
South Korea	Suboptimal diet	64.2 (61.8-67)	61.3 (59.2-63.8)	1732 (1640-1831)	2693 (2559-2843)
	Whole grains	35 (33.6-36.6)	32.6 (31.2-33.9)	945 (893-1002)	1433 (1361-1523)
	Refined grains (total)	33.4 (30.7-37.1)	31 (28.5-34.2)	901 (816-1005)	1364 (1241-1511)
	Refined rice	27.8 (25.5-31.2)	24.6 (22.4-27.5)	750 (677-849)	1081 (978-1214)
	Refined wheat	7.7 (7-9.7)	8.5 (7.8-10.4)	208 (187-264)	375 (338-462)
	Processed meats	2.8 (2-6.2)	1.2 (1-2.8)	77 (54-165)	52 (46-121)
	Unprocessed red meats	13.2 (10.6-17.9)	14.5 (12.2-19)	357 (286-489)	636 (535-840)
	Yogurt	12.1 (11.2-13)	10.7 (9.9-11.5)	327 (300-354)	471 (431-509)
	Sugar-sweetened beverages	3.5 (3-6.5)	2.9 (2.5-5.4)	95 (80-175)	128 (110-239)
	Potatoes	0.9 (0.8-1)	1 (0.9-1.1)	24 (21-27)	43 (39-50)
	Fruit	3.2 (3-3.4)	2.9 (2.7-3)	86 (81-92)	127 (119-135)
	Nuts and seeds	1.3 (1.2-1.4)	1.3 (1.2-1.4)	36 (33-39)	57 (52-62)
	Non-starchy vegetables	1.1 (1-1.2)	1.2 (1.1-1.2)	30 (28-33)	51 (47-55)
	Fruit juice	0 (0-0)	0 (0-0)	1 (1-1)	1 (1-2)
Spain	Suboptimal diet	74.3 (68.6-80.5)	76.4 (70.2-83.6)	3037 (2792-3319)	3858 (3506-4236)

Table S5. Proportional and absolute burden per 1M population of T2D incidence attributable to suboptimal diet* and each individual dietary factor† at the national level in 1990 and 2018

Country	Dietary factor	Proportional burden, %		Rate, per 1M population‡	
		1990	2018	1990	2018
	Whole grains	29.3 (26.2-31.8)	29.5 (26.3-32.3)	1198 (1068-1313)	1489 (1324-1646)
	Refined grains (total)	8.3 (5.8-11.7)	11.8 (8.5-16.2)	337 (238-485)	593 (428-822)
	Refined rice	1 (0.7-1.5)	2 (1.4-2.9)	42 (29-64)	103 (72-148)
	Refined wheat	7.3 (5.2-10.4)	10 (7.1-13.9)	298 (210-430)	504 (362-701)
	Processed meats	28.9 (18.6-44.5)	39.8 (26.9-57.2)	1182 (762-1796)	2018 (1355-2870)
	Unprocessed red meats	22.8 (14.9-33.6)	23 (14.4-32.4)	931 (607-1378)	1161 (717-1652)
	Yogurt	8.1 (6.2-9.5)	8.2 (6.2-9.9)	329 (252-392)	414 (311-500)
	Sugar-sweetened beverages	7.2 (5.7-10.2)	10.6 (8.3-14.3)	294 (233-418)	534 (418-722)
	Potatoes	18.1 (14.3-23.8)	6.2 (4.9-9.1)	740 (585-976)	315 (248-463)
	Fruit	2.2 (2.1-2.4)	3.2 (3-3.4)	91 (83-100)	160 (148-171)
	Nuts and seeds	0.9 (0.8-1)	1 (0.8-1.1)	37 (32-41)	49 (43-56)
	Non-starchy vegetables	1.1 (1-1.2)	1.3 (1.2-1.5)	47 (42-51)	68 (62-74)
	Fruit juice	0.1 (0-0.1)	0.1 (0-0.2)	3 (1-6)	5 (2-10)
Sri Lanka	Suboptimal diet	74 (67.5-81.7)	74.2 (67.4-82.2)	2404 (2174-2660)	6696 (6056-7395)
	Whole grains	24.8 (20.8-28.1)	24.4 (20.2-27.9)	808 (677-919)	2200 (1823-2525)
	Refined grains (total)	41.8 (28.3-58.5)	43.7 (29.6-59.8)	1359 (921-1891)	3936 (2689-5356)
	Refined rice	36 (24.1-51.6)	38.5 (25.8-53.8)	1170 (782-1670)	3467 (2329-4828)
	Refined wheat	9.2 (5.7-14.6)	8.6 (5.4-13.4)	299 (187-475)	776 (484-1204)
	Processed meats	8.4 (4.7-14.1)	7.6 (4.1-14.2)	272 (154-456)	682 (368-1278)
	Unprocessed red meats	24.9 (21.1-29.5)	15.5 (12.6-19.1)	811 (684-965)	1393 (1130-1731)
	Yogurt	11.1 (10.1-12.1)	11 (10-11.9)	362 (328-395)	987 (895-1072)
	Sugar-sweetened beverages	11.2 (8-15.5)	19.4 (14.6-25.4)	363 (261-503)	1735 (1303-2287)
	Potatoes	0.2 (0.1-0.4)	0.9 (0.5-1.6)	7 (4-13)	77 (46-142)
	Fruit	2.8 (2.6-2.9)	3 (2.8-3.2)	90 (85-96)	272 (255-291)
	Nuts and seeds	1.3 (1.2-1.4)	1 (0.7-1.2)	42 (39-45)	88 (64-106)
	Non-starchy vegetables	0.6 (0.5-0.7)	0.7 (0.6-0.9)	20 (17-23)	67 (57-77)
	Fruit juice	0 (0-0)	0 (0-0)	0 (0-1)	1 (0-2)
Sudan	Suboptimal diet	64.7 (58.1-71.4)	69.7 (62-78.1)	1609 (1442-1784)	3180 (2820-3591)
	Whole grains	28.9 (19.5-35.7)	30 (20.3-37.1)	717 (482-893)	1372 (923-1693)
	Refined grains (total)	18.5 (13.3-25.6)	20.1 (14.4-27.3)	461 (330-638)	916 (659-1256)
	Refined rice	18.5 (13.3-25.6)	20.1 (14.4-27.3)	461 (330-638)	916 (659-1256)
	Refined wheat	0 (0-0)	0 (0-0)	0 (0-0)	0 (0-0)
	Processed meats	6.2 (1.9-15.9)	11.8 (3-33.5)	153 (47-397)	535 (135-1512)
	Unprocessed red meats	8.9 (6.1-12.8)	7.2 (4.9-11.3)	221 (153-315)	328 (226-515)
	Yogurt	12.5 (11.5-13.5)	13.1 (11.9-14.1)	312 (283-338)	597 (541-647)

Table S5. Proportional and absolute burden per 1M population of T2D incidence attributable to suboptimal diet* and each individual dietary factor† at the national level in 1990 and 2018

Country	Dietary factor	Proportional burden, %		Rate, per 1M population‡	
		1990	2018	1990	2018
	Sugar-sweetened beverages	25.5 (16.6-36.7)	27 (17.3-39)	632 (411-908)	1237 (790-1796)
	Potatoes	1.2 (0.7-2.3)	5.3 (3-10.1)	29 (17-56)	242 (138-459)
	Fruit	2.8 (2.4-3.2)	3.3 (2.8-3.7)	70 (59-80)	150 (125-171)
	Nuts and seeds	1.2 (1.1-1.3)	1 (0.8-1.2)	30 (27-34)	46 (38-55)
	Non-starchy vegetables	1.1 (0.7-1.4)	1.3 (0.9-1.6)	27 (19-34)	58 (40-74)
	Fruit juice	0.2 (0.1-0.3)	0.2 (0.1-0.3)	4 (3-6)	9 (6-15)
Suriname	Suboptimal diet	80.3 (74.6-86.6)	85.5 (80.2-90.5)	3248 (3002-3533)	7011 (6533-7500)
	Whole grains	36.5 (30.8-39.9)	26.1 (17-33)	1475 (1241-1626)	2140 (1384-2729)
	Refined grains (total)	26 (19-33.7)	21 (14.9-28.2)	1049 (772-1361)	1723 (1235-2329)
	Refined rice	20.8 (15.1-27.5)	15.5 (10.8-21.2)	843 (613-1108)	1272 (895-1742)
	Refined wheat	7 (4.9-9.7)	6.9 (4.7-9.7)	281 (198-390)	564 (384-798)
	Processed meats	37.1 (22.1-54.9)	45.4 (29.9-63)	1501 (893-2244)	3727 (2435-5217)
	Unprocessed red meats	16.7 (11.8-22.8)	28.3 (21.4-36.9)	680 (476-928)	2324 (1758-3044)
	Yogurt	12.1 (10.8-13.1)	12.2 (10.7-13.3)	490 (436-535)	1000 (881-1094)
	Sugar-sweetened beverages	23 (16.1-33.1)	31.7 (22.4-44.1)	931 (658-1320)	2611 (1844-3609)
	Potatoes	0.7 (0.4-1.2)	2.5 (1.3-5.2)	27 (14-49)	207 (107-422)
	Fruit	3.2 (2.8-3.5)	3 (2.4-3.5)	128 (113-141)	248 (198-287)
	Nuts and seeds	1.2 (1-1.3)	1 (0.8-1.1)	47 (42-52)	78 (63-94)
	Non-starchy vegetables	0.8 (0.4-1.1)	0.9 (0.5-1.3)	31 (16-45)	76 (41-109)
	Fruit juice	0.2 (0.1-0.3)	0.2 (0.1-0.3)	6 (4-11)	14 (8-23)
Swaziland	Suboptimal diet	64.2 (55.7-76)	72.5 (67.9-78.1)	1774 (1531-2097)	3257 (3037-3514)
	Whole grains	20.3 (12.7-27.8)	42 (40.7-43.3)	560 (347-767)	1884 (1802-1963)
	Refined grains (total)	9.3 (7.5-11.6)	24.1 (16.9-33.6)	255 (206-323)	1085 (763-1509)
	Refined rice	2.8 (2.2-3.8)	24.1 (16.9-33.6)	76 (61-104)	1085 (763-1509)
	Refined wheat	6.6 (5.4-8.5)	0 (0-0)	184 (148-236)	0 (0-0)
	Processed meats	13.7 (3-40.7)	3.4 (1.1-9.6)	378 (85-1116)	150 (47-438)
	Unprocessed red meats	8.7 (6.2-12.5)	9.2 (6.5-12.8)	240 (172-345)	412 (292-572)
	Yogurt	11.2 (9.8-12.2)	11.2 (9.8-12.3)	308 (270-340)	502 (439-554)
	Sugar-sweetened beverages	27.3 (19.3-37)	33 (23.9-44.7)	751 (527-1021)	1478 (1075-2010)
	Potatoes	5 (2.8-9.4)	3.8 (2.2-7.3)	138 (76-262)	170 (98-327)
	Fruit	2.6 (2.4-2.7)	3 (2.8-3.2)	71 (66-76)	133 (123-143)
	Nuts and seeds	1.3 (1.2-1.3)	1.1 (0.9-1.2)	35 (33-38)	49 (42-55)
	Non-starchy vegetables	0.9 (0.8-1)	0.9 (0.8-1.1)	25 (22-28)	42 (34-50)
	Fruit juice	0.1 (0.1-0.1)	0.1 (0.1-0.2)	2 (2-4)	5 (3-8)
Sweden	Suboptimal diet	68.7 (65.2-72.4)	75.4 (71.9-79.1)	1794 (1681-1919)	2930 (2750-3119)

Table S5. Proportional and absolute burden per 1M population of T2D incidence attributable to suboptimal diet* and each individual dietary factor† at the national level in 1990 and 2018

Country	Dietary factor	Proportional burden, %		Rate, per 1M population‡	
		1990	2018	1990	2018
	Whole grains	24 (21.3-26.3)	27.5 (25.3-29.7)	627 (552-695)	1069 (971-1164)
	Refined grains (total)	1.8 (1.1-2.9)	2.8 (1.7-4.6)	46 (28-77)	110 (68-178)
	Refined rice	0.2 (0.1-0.4)	0.3 (0.2-0.5)	6 (4-10)	12 (8-21)
	Refined wheat	1.5 (1-2.6)	2.5 (1.6-4.1)	40 (25-67)	97 (60-160)
	Processed meats	27.8 (21.8-35.2)	39.9 (32.6-48.1)	723 (571-925)	1547 (1274-1880)
	Unprocessed red meats	27.3 (24.1-31.4)	28.4 (25.4-33.5)	716 (628-824)	1104 (982-1301)
	Yogurt	4.5 (3.4-5.4)	4.3 (3.3-5.5)	116 (91-143)	169 (130-211)
	Sugar-sweetened beverages	7.5 (6.4-10.2)	7.5 (6.3-10.4)	197 (166-271)	292 (244-412)
	Potatoes	6.2 (5.4-8.1)	6.1 (5.4-8.5)	162 (142-214)	238 (209-337)
	Fruit	2.2 (2.1-2.4)	2.5 (2.3-2.6)	59 (54-63)	95 (87-103)
	Nuts and seeds	1.1 (1-1.2)	1.1 (1-1.2)	29 (27-32)	42 (38-46)
	Non-starchy vegetables	1.3 (1.2-1.4)	1 (0.9-1.1)	33 (31-36)	37 (33-41)
	Fruit juice	0.2 (0.2-0.3)	0.3 (0.2-0.3)	6 (5-8)	11 (9-13)
Switzerland	Suboptimal diet	67.2 (62.7-72.2)	65.2 (60.4-70.3)	2003 (1861-2166)	2633 (2426-2857)
	Whole grains	19.4 (15.9-22.9)	20.6 (16.4-24.1)	580 (476-684)	833 (664-970)
	Refined grains (total)	10.9 (8.3-14.8)	8.8 (6.7-12)	326 (246-443)	355 (267-481)
	Refined rice	1 (0.7-1.4)	0.4 (0.3-0.6)	29 (22-42)	18 (13-24)
	Refined wheat	10.1 (7.6-13.7)	8.4 (6.4-11.5)	299 (226-408)	339 (255-461)
	Processed meats	25.3 (19.2-32.5)	27.8 (20.9-36.3)	751 (571-965)	1121 (846-1475)
	Unprocessed red meats	28.9 (22-37.1)	19.8 (14.2-26.5)	858 (652-1103)	800 (574-1068)
	Yogurt	6.1 (4.7-7.5)	6.1 (4.6-7.6)	182 (139-225)	244 (185-305)
	Sugar-sweetened beverages	8.9 (7.2-11.8)	10.4 (8.3-13.6)	265 (213-352)	418 (336-551)
	Potatoes	4.6 (3.6-6.4)	4.5 (3.6-6.1)	138 (109-194)	182 (144-251)
	Fruit	1.7 (1.4-1.9)	2.2 (1.9-2.5)	50 (42-58)	89 (76-100)
	Nuts and seeds	1 (0.9-1.1)	1.1 (0.9-1.2)	30 (26-33)	43 (38-49)
	Non-starchy vegetables	1.1 (1-1.2)	1.3 (1.1-1.4)	33 (29-36)	51 (45-56)
	Fruit juice	0.1 (0.1-0.2)	0.2 (0.1-0.2)	4 (3-6)	8 (6-10)
Syria	Suboptimal diet	68.3 (61.2-76.2)	72.4 (64.4-79.9)	2045 (1829-2296)	3484 (3096-3869)
	Whole grains	35.1 (27.2-39.8)	33.9 (26-38.6)	1049 (815-1193)	1629 (1243-1855)
	Refined grains (total)	27.4 (16.5-40.8)	30.3 (18.6-43.3)	821 (495-1217)	1458 (899-2101)
	Refined rice	27.4 (16.5-40.8)	30.3 (18.6-43.3)	821 (495-1217)	1458 (899-2101)
	Refined wheat	0 (0-0)	0 (0-0)	0 (0-0)	0 (0-0)
	Processed meats	15.6 (7.2-29.8)	21.4 (9.6-39.8)	470 (215-900)	1038 (466-1939)
	Unprocessed red meats	14.3 (8.9-20.5)	13.3 (8.5-20.5)	425 (264-619)	644 (408-1000)
	Yogurt	8.2 (5.6-10.4)	7.8 (5.2-9.9)	246 (167-312)	376 (248-476)

Table S5. Proportional and absolute burden per 1M population of T2D incidence attributable to suboptimal diet* and each individual dietary factor† at the national level in 1990 and 2018

Country	Dietary factor	Proportional burden, %		Rate, per 1M population‡	
		1990	2018	1990	2018
	Sugar-sweetened beverages	9.8 (7-13.7)	11.6 (8.4-16.1)	295 (207-410)	556 (402-782)
	Potatoes	3.9 (2.1-6.8)	6 (3.4-10.9)	116 (63-205)	289 (162-523)
	Fruit	2.7 (2.3-3.1)	2.7 (2.2-3.2)	81 (67-91)	131 (106-155)
	Nuts and seeds	1.2 (1-1.3)	0.9 (0.7-1.1)	34 (30-39)	41 (32-52)
	Non-starchy vegetables	1 (0.8-1.3)	1.1 (0.8-1.4)	31 (23-38)	54 (40-67)
	Fruit juice	0.1 (0.1-0.2)	0.1 (0.1-0.2)	3 (2-6)	6 (3-12)
Taiwan	Suboptimal diet	76 (71.4-80.6)	71.5 (67.1-75.8)	2195 (2041-2333)	3247 (3041-3472)
	Whole grains	38.7 (37.4-39.8)	32.1 (30.2-33.7)	1116 (1068-1162)	1458 (1365-1540)
	Refined grains (total)	32.9 (22.8-43.5)	25.7 (17-35.6)	950 (657-1257)	1163 (778-1621)
	Refined rice	25.9 (17.7-35)	17.4 (11.3-25)	751 (509-1012)	789 (515-1130)
	Refined wheat	10.5 (6.8-15)	10.7 (6.8-15.8)	305 (196-431)	486 (309-716)
	Processed meats	13.5 (9.1-19.7)	14 (10.3-19.3)	391 (258-569)	634 (465-879)
	Unprocessed red meats	23.5 (19.6-27.7)	25.1 (21.2-29.5)	677 (566-804)	1135 (961-1339)
	Yogurt	12.4 (11.5-13.2)	11.7 (10.7-12.6)	358 (331-383)	532 (485-577)
	Sugar-sweetened beverages	18.3 (14.6-23.1)	13.4 (10.6-17.5)	527 (420-659)	608 (479-799)
	Potatoes	0.2 (0.1-0.4)	1.3 (0.7-2.7)	6 (3-11)	61 (31-122)
	Fruit	2.3 (2-2.6)	2.4 (2-2.7)	67 (57-75)	109 (91-124)
	Nuts and seeds	1.2 (1.1-1.3)	1.2 (1.2-1.3)	35 (33-37)	56 (52-61)
	Non-starchy vegetables	0.4 (0.3-0.6)	0.4 (0.2-0.6)	13 (8-19)	17 (10-26)
	Fruit juice	0 (0-0)	0 (0-0)	1 (1-1)	1 (1-2)
Tajikistan	Suboptimal diet	89.1 (82.4-93.5)	91.3 (84.8-95.6)	1504 (1381-1607)	3817 (3527-4069)
	Whole grains	16.2 (9.9-24.3)	25.2 (17.3-32.7)	273 (167-415)	1054 (726-1364)
	Refined grains (total)	16.1 (12-21)	22.1 (16.8-28.5)	272 (202-355)	930 (699-1212)
	Refined rice	1.1 (0.8-1.7)	2.8 (2-4)	19 (14-29)	117 (85-167)
	Refined wheat	15.1 (11.3-19.8)	19.9 (15-25.8)	255 (189-334)	834 (625-1096)
	Processed meats	77.9 (65.3-86.6)	73 (53.7-86.7)	1315 (1097-1475)	3058 (2216-3646)
	Unprocessed red meats	13.9 (9.4-19.6)	25.5 (19.4-32.9)	236 (156-331)	1067 (816-1383)
	Yogurt	8.1 (5.5-10.1)	7.9 (5.4-10)	136 (93-172)	331 (227-422)
	Sugar-sweetened beverages	4.2 (2.5-7.5)	14.9 (10.3-21.2)	71 (43-129)	623 (429-898)
	Potatoes	8.9 (5.3-15.4)	11.4 (7-18.8)	151 (89-260)	477 (291-781)
	Fruit	3 (2.6-3.3)	3.1 (2.7-3.5)	51 (45-57)	131 (113-148)
	Nuts and seeds	1.2 (1.1-1.4)	1.4 (1.2-1.5)	20 (18-23)	58 (52-65)
	Non-starchy vegetables	1.7 (1.5-1.9)	1.9 (1.7-2.1)	29 (26-32)	80 (70-89)
	Fruit juice	0.1 (0.1-0.1)	0.1 (0.1-0.1)	2 (1-2)	4 (3-6)
Tanzania	Suboptimal diet	49.7 (45.2-54.6)	64.4 (59.6-70.1)	650 (588-717)	1315 (1210-1430)

Table S5. Proportional and absolute burden per 1M population of T2D incidence attributable to suboptimal diet* and each individual dietary factor† at the national level in 1990 and 2018

Country	Dietary factor	Proportional burden, %		Rate, per 1M population‡	
		1990	2018	1990	2018
	Whole grains	3.2 (2-5)	4.8 (3-7.4)	42 (26-65)	99 (60-152)
	Refined grains (total)	23 (19.1-27.7)	22.6 (19.2-26.6)	301 (246-364)	461 (391-538)
	Refined rice	20.2 (16.6-24.3)	17.4 (14.6-20.6)	263 (215-321)	355 (297-420)
	Refined wheat	3.6 (2.9-4.8)	6.3 (5.3-8)	46 (37-62)	129 (108-162)
	Processed meats	3.3 (0.9-10.3)	5 (1.4-13.5)	44 (12-133)	102 (29-279)
	Unprocessed red meats	14.3 (11-18.4)	14.3 (11.1-18.5)	186 (144-241)	292 (227-377)
	Yogurt	11.7 (10.7-12.5)	12.7 (11.7-13.5)	153 (140-165)	258 (238-276)
	Sugar-sweetened beverages	1.7 (1.1-3.3)	27.4 (20-36.3)	23 (14-43)	558 (407-736)
	Potatoes	2.1 (1.2-4.1)	2.5 (1.4-4.7)	28 (16-54)	51 (29-95)
	Fruit	2.7 (2.6-2.9)	3.3 (3.1-3.5)	36 (34-38)	68 (63-72)
	Nuts and seeds	1.2 (1.1-1.3)	0.9 (0.7-1.1)	16 (15-17)	18 (15-22)
	Non-starchy vegetables	0.7 (0.6-0.8)	0.9 (0.7-1)	9 (8-10)	18 (15-21)
	Fruit juice	0.1 (0-0.1)	0.1 (0.1-0.1)	1 (1-1)	2 (1-3)
Thailand	Suboptimal diet	64.8 (58.3-72)	65.9 (59.1-72.8)	1550 (1387-1729)	2937 (2619-3262)
	Whole grains	38.4 (35.1-40.4)	31.9 (26.2-35.7)	916 (836-974)	1423 (1163-1598)
	Refined grains (total)	31 (21.3-41.6)	27.4 (18.5-37.3)	742 (511-996)	1220 (829-1679)
	Refined rice	30.5 (20.9-41)	26.1 (17.6-35.7)	731 (502-983)	1164 (791-1608)
	Refined wheat	0.8 (0.5-1.2)	1.9 (1.2-2.9)	18 (11-28)	84 (53-130)
	Processed meats	8.6 (3.6-17.9)	7.9 (3.6-16.1)	205 (87-427)	352 (161-710)
	Unprocessed red meats	12.5 (7.2-19.7)	12.6 (7.1-20.1)	298 (170-477)	564 (320-892)
	Yogurt	12.6 (11.8-13.4)	12 (11.2-12.7)	302 (281-322)	534 (494-572)
	Sugar-sweetened beverages	2.8 (1.3-6.1)	13.7 (7.1-25.8)	67 (31-145)	610 (317-1143)
	Potatoes	0.1 (0-0.1)	0.7 (0.3-1.3)	2 (1-3)	30 (16-60)
	Fruit	2.8 (2.4-3.1)	2.9 (2.4-3.2)	66 (58-74)	127 (109-142)
	Nuts and seeds	1.2 (1.1-1.3)	1.3 (1.2-1.4)	29 (27-32)	59 (54-64)
	Non-starchy vegetables	0.8 (0.5-1.1)	0.9 (0.6-1.2)	19 (12-26)	41 (26-53)
	Fruit juice	0 (0-0)	0 (0-0)	1 (0-1)	1 (1-2)
The Bahamas	Suboptimal diet	87.7 (82.4-91.9)	72.1 (64.8-79.9)	3274 (3059-3503)	4249 (3814-4718)
	Whole grains	25.4 (16.4-32.3)	27.4 (18.4-33.9)	952 (607-1221)	1615 (1085-2007)
	Refined grains (total)	20 (14.4-26.6)	17.5 (12-24.2)	746 (528-1001)	1035 (710-1410)
	Refined rice	10.3 (7.2-14.4)	9.6 (6.5-13.8)	385 (267-547)	566 (380-814)
	Refined wheat	11.2 (7.9-15.7)	9 (6.1-13.2)	421 (294-591)	533 (360-777)
	Processed meats	21.3 (11-36.8)	23.4 (11.9-41.7)	798 (406-1374)	1379 (696-2473)
	Unprocessed red meats	35.2 (26.6-44.6)	18.9 (13.3-26.1)	1316 (987-1666)	1116 (783-1543)
	Yogurt	11.7 (10.3-12.8)	11.5 (10-12.7)	438 (385-485)	680 (589-753)

Table S5. Proportional and absolute burden per 1M population of T2D incidence attributable to suboptimal diet* and each individual dietary factor† at the national level in 1990 and 2018

Country	Dietary factor	Proportional burden, %		Rate, per 1M population‡	
		1990	2018	1990	2018
	Sugar-sweetened beverages	56.7 (42.8-68.8)	21 (13.8-30.3)	2117 (1593-2606)	1238 (812-1778)
	Potatoes	1 (0.5-1.9)	1 (0.5-1.8)	39 (20-72)	58 (30-109)
	Fruit	3 (2.6-3.3)	3.2 (2.7-3.6)	112 (98-125)	189 (156-216)
	Nuts and seeds	1.3 (1.2-1.4)	1.1 (0.9-1.2)	48 (44-52)	63 (52-73)
	Non-starchy vegetables	1.3 (1-1.5)	1.5 (1.2-1.7)	48 (37-57)	88 (69-104)
	Fruit juice	0.1 (0.1-0.2)	0.1 (0.1-0.2)	5 (3-9)	7 (4-12)
The Gambia	Suboptimal diet	78.9 (71.2-85.8)	68.4 (57.4-80.3)	1079 (964-1185)	1613 (1342-1915)
	Whole grains	26.2 (18-32.1)	20.8 (12.8-28.4)	357 (246-441)	489 (300-674)
	Refined grains (total)	12.7 (9.9-16.2)	11.4 (8.9-14.7)	173 (135-221)	269 (209-348)
	Refined rice	11.3 (8.8-14.6)	8.3 (6.5-11)	154 (120-200)	197 (152-260)
	Refined wheat	1.5 (1.2-2.1)	3.3 (2.5-4.5)	21 (16-29)	77 (59-107)
	Processed meats	23.9 (10.3-45.5)	34.3 (15.4-58.6)	328 (140-625)	811 (358-1375)
	Unprocessed red meats	5.3 (3.3-8.5)	2.5 (1.4-5.8)	73 (44-116)	58 (33-139)
	Yogurt	12.6 (11.7-13.4)	12.9 (12-13.8)	172 (159-184)	305 (281-330)
	Sugar-sweetened beverages	51.6 (40-61.9)	21 (14-29.7)	706 (545-849)	493 (328-699)
	Potatoes	0.2 (0.1-0.4)	0.4 (0.2-0.7)	3 (2-6)	8 (5-16)
	Fruit	3.6 (3.4-3.7)	3.8 (3.6-4)	49 (46-51)	89 (83-97)
	Nuts and seeds	0.9 (0.7-1)	1 (0.8-1.2)	12 (10-14)	23 (18-28)
	Non-starchy vegetables	1.5 (1.4-1.6)	1.9 (1.7-2)	21 (19-23)	44 (40-48)
	Fruit juice	0.1 (0.1-0.1)	0.1 (0.1-0.1)	1 (1-2)	2 (1-3)
Timor-Leste	Suboptimal diet	60.2 (55.7-65.1)	73.6 (68.3-79.1)	961 (885-1051)	2818 (2600-3042)
	Whole grains	35.2 (29.4-39.1)	31.5 (25.4-35.8)	563 (471-628)	1204 (967-1376)
	Refined grains (total)	18 (15.1-22)	18.7 (15.6-22.2)	288 (242-352)	718 (597-857)
	Refined rice	17 (14.2-20.7)	17 (14.2-20.3)	271 (228-332)	652 (541-779)
	Refined wheat	1.2 (1-1.6)	2.1 (1.7-2.7)	20 (16-26)	78 (65-103)
	Processed meats	9.6 (4.1-19.5)	17.5 (9.1-32.2)	153 (66-312)	670 (352-1225)
	Unprocessed red meats	11.1 (8.6-14.6)	6.1 (4.4-8.9)	177 (138-233)	232 (169-343)
	Yogurt	13.5 (12.9-14.2)	12.8 (12.2-13.5)	216 (205-229)	490 (464-518)
	Sugar-sweetened beverages	1.9 (1.1-4)	34.9 (27.8-43)	30 (18-64)	1335 (1062-1643)
	Potatoes	4.9 (2.5-10)	3.6 (1.9-7.5)	79 (40-160)	140 (71-290)
	Fruit	3.3 (3.1-3.4)	3.5 (3.3-3.7)	53 (50-55)	134 (127-141)
	Nuts and seeds	1.4 (1.3-1.5)	1.3 (1.2-1.4)	22 (21-24)	51 (47-55)
	Non-starchy vegetables	1 (0.9-1.1)	1.1 (0.9-1.2)	16 (14-18)	41 (36-46)
	Fruit juice	0 (0-0.1)	0.1 (0-0.1)	1 (0-1)	2 (1-3)
Togo	Suboptimal diet	57.2 (47.6-70.3)	85.4 (80.3-90.8)	635 (525-780)	1685 (1579-1799)

Table S5. Proportional and absolute burden per 1M population of T2D incidence attributable to suboptimal diet* and each individual dietary factor† at the national level in 1990 and 2018

Country	Dietary factor	Proportional burden, %		Rate, per 1M population‡	
		1990	2018	1990	2018
	Whole grains	23.1 (15.5-30.4)	20.9 (13.4-28.6)	257 (173-338)	415 (264-566)
	Refined grains (total)	5.6 (4.2-7.5)	11.6 (8.9-14.9)	62 (46-84)	231 (174-296)
	Refined rice	3 (2.2-4.2)	8.5 (6.4-11)	34 (25-47)	168 (126-218)
	Refined wheat	2.6 (2-3.6)	3.4 (2.6-4.6)	29 (22-40)	68 (51-92)
	Processed meats	21.1 (7.7-44.3)	24.5 (9.1-48)	235 (86-491)	483 (178-959)
	Unprocessed red meats	7.8 (5.4-11.2)	6 (4-9.1)	86 (60-123)	119 (79-181)
	Yogurt	12.3 (11.4-13)	12.6 (11.7-13.3)	136 (126-145)	248 (230-265)
	Sugar-sweetened beverages	7.1 (4.6-11.2)	66.8 (58.4-72.7)	79 (50-124)	1319 (1152-1443)
	Potatoes	1 (0.5-2)	2.8 (1.4-5.4)	11 (6-23)	54 (28-107)
	Fruit	3 (2.8-3.2)	3.5 (3.3-3.7)	33 (31-36)	69 (64-74)
	Nuts and seeds	0.9 (0.7-1)	1.1 (0.9-1.2)	10 (8-12)	21 (18-24)
	Non-starchy vegetables	0.8 (0.6-1)	1.1 (0.8-1.3)	8 (6-11)	21 (17-25)
	Fruit juice	0 (0-0)	0 (0-0.1)	0 (0-1)	1 (0-1)
Tonga	Suboptimal diet	70.1 (62.9-76.6)	76.7 (69.8-83.1)	3323 (2989-3669)	5748 (5173-6259)
	Whole grains	36.9 (31.5-40)	38 (32.4-41.3)	1744 (1476-1926)	2848 (2420-3121)
	Refined grains (total)	33.6 (20.8-46.7)	39.2 (25.7-53.2)	1584 (991-2211)	2946 (1907-4006)
	Refined rice	33.6 (20.8-46.7)	39.2 (25.7-53.2)	1584 (991-2211)	2946 (1907-4006)
	Refined wheat	0 (0-0)	0 (0-0)	0 (0-0)	0 (0-0)
	Processed meats	9.8 (4.3-19.4)	11.3 (5.1-22.5)	460 (207-941)	845 (378-1687)
	Unprocessed red meats	18.8 (13.2-26)	14.3 (9.5-20.8)	897 (627-1237)	1065 (716-1557)
	Yogurt	12.9 (11.8-13.9)	13.3 (12.2-14.4)	612 (555-669)	999 (913-1088)
	Sugar-sweetened beverages	6.2 (3.8-10.3)	20.4 (13.8-29.6)	292 (178-487)	1523 (1038-2235)
	Potatoes	0.2 (0.1-0.5)	1.5 (0.7-3.2)	11 (6-22)	116 (55-238)
	Fruit	2.6 (2.2-2.9)	2.7 (2.2-3.1)	124 (107-140)	203 (168-234)
	Nuts and seeds	1.2 (1.1-1.3)	1.3 (1.2-1.5)	58 (50-64)	100 (88-112)
	Non-starchy vegetables	1 (0.8-1.2)	1.2 (1-1.4)	48 (39-57)	89 (72-105)
	Fruit juice	0 (0-0.1)	0 (0-0.1)	2 (1-3)	3 (2-5)
Trinidad and Tobago	Suboptimal diet	79.7 (74.3-85.2)	83.1 (76.9-89.2)	5211 (4817-5649)	7575 (6960-8246)
	Whole grains	38.9 (36.4-41)	27.7 (21.1-33)	2541 (2342-2717)	2533 (1919-3038)
	Refined grains (total)	31.4 (25.8-37.9)	10 (8.1-12.7)	2049 (1690-2494)	914 (735-1163)
	Refined rice	14.1 (11.3-17.9)	2.2 (1.8-2.9)	922 (739-1165)	200 (160-267)
	Refined wheat	20.1 (16.3-25)	7.9 (6.4-10.2)	1317 (1068-1643)	726 (584-933)
	Processed meats	24.9 (12.8-41.2)	39.4 (22.9-58.6)	1632 (835-2694)	3589 (2064-5409)
	Unprocessed red meats	11.6 (7.5-17.9)	25 (18.1-33.3)	761 (489-1168)	2272 (1642-3034)
	Yogurt	11.8 (10.4-12.9)	11.5 (10-12.7)	769 (678-848)	1049 (905-1158)

Table S5. Proportional and absolute burden per 1M population of T2D incidence attributable to suboptimal diet* and each individual dietary factor† at the national level in 1990 and 2018

Country	Dietary factor	Proportional burden, %		Rate, per 1M population‡	
		1990	2018	1990	2018
	Sugar-sweetened beverages	30.2 (21.1-40.4)	34.7 (25.2-47.4)	1974 (1379-2637)	3168 (2324-4324)
	Potatoes	1.4 (0.7-2.7)	3.9 (2.1-7.6)	92 (48-173)	363 (195-692)
	Fruit	3 (2.8-3.2)	3.4 (3.2-3.6)	197 (182-211)	308 (285-329)
	Nuts and seeds	1.3 (1.2-1.4)	1 (0.7-1.2)	87 (80-95)	90 (64-113)
	Non-starchy vegetables	1.6 (1.5-1.7)	1.6 (1.5-1.8)	104 (95-113)	148 (134-161)
	Fruit juice	0.2 (0.1-0.3)	0.2 (0.1-0.3)	10 (6-18)	14 (8-23)
Tunisia	Suboptimal diet	64.9 (60.7-70.6)	68.9 (64.4-74.5)	2012 (1863-2201)	4543 (4208-4922)
	Whole grains	41.2 (39.7-42.5)	41.2 (39.6-42.5)	1275 (1214-1333)	2711 (2567-2841)
	Refined grains (total)	19.8 (12.6-30.9)	20 (12.7-30.9)	615 (391-957)	1317 (834-2050)
	Refined rice	0.2 (0.1-0.3)	0.2 (0.1-0.4)	6 (3-10)	16 (9-27)
	Refined wheat	19.7 (12.5-30.7)	19.8 (12.6-30.6)	611 (388-951)	1304 (826-2033)
	Processed meats	0.8 (0.4-2.1)	1 (0.5-3)	26 (13-67)	65 (32-198)
	Unprocessed red meats	15.1 (8.7-25.3)	14.6 (7.8-24.5)	469 (270-789)	962 (519-1613)
	Yogurt	9.1 (6.5-10.9)	8.6 (6-10.5)	282 (204-340)	563 (395-690)
	Sugar-sweetened beverages	14 (10.1-19.9)	22.6 (15.9-31.4)	433 (311-614)	1488 (1048-2059)
	Potatoes	4.7 (3-7.7)	7.2 (4.6-11.3)	147 (93-239)	471 (305-751)
	Fruit	3.1 (2.9-3.2)	3.5 (3.3-3.7)	95 (89-101)	228 (212-244)
	Nuts and seeds	1.1 (0.9-1.2)	1.1 (1-1.3)	33 (29-37)	73 (63-83)
	Non-starchy vegetables	1.2 (1.1-1.3)	0.7 (0.5-0.8)	38 (34-41)	46 (35-56)
	Fruit juice	0.1 (0.1-0.2)	0.1 (0.1-0.3)	4 (2-7)	9 (5-17)
Turkey	Suboptimal diet	62.8 (59-67.2)	63 (59.1-68.5)	1878 (1759-2015)	2915 (2706-3184)
	Whole grains	40.5 (39.1-41.9)	40.1 (38.5-41.5)	1213 (1160-1267)	1852 (1762-1946)
	Refined grains (total)	12.2 (6.5-21.8)	16.5 (8.9-28.7)	364 (196-652)	762 (414-1326)
	Refined rice	0.7 (0.4-1.4)	2.1 (1.1-4.2)	21 (11-42)	95 (49-195)
	Refined wheat	11.6 (6.2-20.8)	14.8 (8-26)	346 (186-620)	682 (368-1200)
	Processed meats	5.6 (3.1-9.8)	6.2 (3.3-12)	166 (92-294)	289 (153-548)
	Unprocessed red meats	16.6 (10.9-24.2)	19 (12.3-27.8)	494 (322-725)	881 (570-1291)
	Yogurt	3.5 (1.5-6.3)	3.4 (1.5-6.4)	106 (46-191)	158 (69-296)
	Sugar-sweetened beverages	11.1 (8-14.9)	9.8 (7-13.8)	332 (238-449)	454 (323-631)
	Potatoes	4.1 (2-8.2)	1.9 (1-4)	123 (61-247)	90 (45-186)
	Fruit	2.8 (2.7-3)	3.2 (2.9-3.4)	85 (80-91)	146 (135-158)
	Nuts and seeds	0.9 (0.7-1.1)	0.9 (0.7-1.1)	27 (22-32)	42 (34-51)
	Non-starchy vegetables	1.2 (1.1-1.3)	1.3 (1.2-1.5)	37 (34-40)	62 (56-68)
	Fruit juice	0.1 (0.1-0.3)	0.2 (0.1-0.3)	4 (2-8)	7 (4-14)
Turkmenistan	Suboptimal diet	92.4 (87.2-95.9)	88.7 (81.7-93.9)	1523 (1426-1610)	2599 (2383-2779)

Table S5. Proportional and absolute burden per 1M population of T2D incidence attributable to suboptimal diet* and each individual dietary factor† at the national level in 1990 and 2018

Country	Dietary factor	Proportional burden, %		Rate, per 1M population‡	
		1990	2018	1990	2018
	Whole grains	38.9 (33.6-41.9)	11.4 (6.3-18.3)	641 (553-702)	334 (183-535)
	Refined grains (total)	12.7 (8.9-17.8)	18.2 (13.1-24.8)	209 (147-292)	531 (379-731)
	Refined rice	1.3 (0.9-2)	2.7 (1.9-4.1)	21 (14-33)	79 (54-121)
	Refined wheat	11.6 (8.2-16.3)	16.1 (11.5-22)	191 (134-269)	469 (333-652)
	Processed meats	62.7 (41.8-79.3)	67.4 (49.6-81.9)	1028 (689-1314)	1975 (1437-2408)
	Unprocessed red meats	21.9 (16.3-28.3)	37.7 (30.6-45.7)	361 (266-465)	1105 (893-1339)
	Yogurt	7.9 (5.3-9.9)	8.6 (5.9-10.7)	131 (88-164)	252 (175-314)
	Sugar-sweetened beverages	47.7 (39-57)	2.2 (1.3-4.5)	784 (643-945)	65 (38-129)
	Potatoes	6.9 (4.2-11.7)	12 (7.5-18.6)	114 (69-194)	349 (219-546)
	Fruit	3.7 (3.5-3.9)	3.6 (3.3-3.8)	60 (56-65)	105 (97-113)
	Nuts and seeds	1.4 (1.3-1.5)	1.6 (1.5-1.7)	22 (20-25)	46 (43-50)
	Non-starchy vegetables	1.5 (1.4-1.7)	1.4 (1.2-1.6)	26 (23-29)	41 (34-47)
	Fruit juice	0.2 (0.1-0.4)	0.2 (0.1-0.4)	3 (2-6)	6 (4-10)
Uganda	Suboptimal diet	44.5 (41.1-48.3)	68.6 (62.9-75.3)	844 (773-921)	1639 (1495-1808)
	Whole grains	28.2 (22.1-32.3)	29.8 (23.3-34.3)	534 (417-616)	709 (555-820)
	Refined grains (total)	16.2 (12.4-20.3)	16.5 (13.9-20)	307 (237-387)	396 (329-482)
	Refined rice	13.2 (10.1-16.6)	8.5 (7.1-10.7)	250 (192-318)	203 (169-256)
	Refined wheat	3.4 (2.6-4.6)	8.8 (7.3-10.9)	65 (50-88)	209 (173-262)
	Processed meats	1.1 (0.3-4.5)	6.8 (2.1-19.3)	20 (6-84)	164 (49-460)
	Unprocessed red meats	2.3 (1.6-5.3)	5 (3.4-8.4)	45 (30-100)	120 (81-199)
	Yogurt	12.6 (11.6-13.4)	13.5 (12.6-14.3)	238 (219-257)	322 (297-345)
	Sugar-sweetened beverages	0.7 (0.4-1.5)	36.4 (27.3-47.1)	13 (8-29)	868 (659-1133)
	Potatoes	1.2 (0.7-2.4)	0.3 (0.2-0.5)	23 (13-45)	7 (4-13)
	Fruit	3 (2.8-3.1)	3.6 (3.4-3.8)	56 (53-60)	85 (80-91)
	Nuts and seeds	0.9 (0.7-1.1)	1.1 (1-1.3)	17 (14-21)	27 (23-31)
	Non-starchy vegetables	1.1 (1-1.2)	1.4 (1.3-1.5)	22 (20-24)	33 (30-36)
	Fruit juice	0 (0-0.1)	0.1 (0.1-0.1)	1 (1-1)	2 (1-3)
Ukraine	Suboptimal diet	86.7 (79.4-92.4)	79.6 (71.4-86.1)	1611 (1450-1742)	1909 (1701-2099)
	Whole grains	23.8 (16.6-31)	20.5 (13.6-28)	442 (314-578)	492 (326-677)
	Refined grains (total)	11.5 (8.5-15.7)	14.9 (11-19.5)	214 (157-292)	357 (260-474)
	Refined rice	0.2 (0.2-0.3)	0.7 (0.5-1)	4 (3-5)	17 (12-25)
	Refined wheat	11.3 (8.3-15.5)	14.3 (10.5-18.7)	211 (154-287)	342 (249-455)
	Processed meats	56.9 (41-71)	52.6 (39.1-66.6)	1060 (767-1326)	1268 (929-1611)
	Unprocessed red meats	40.2 (24.1-60.2)	16.8 (8.1-30)	746 (450-1121)	404 (194-725)
	Yogurt	6.9 (4.6-8.9)	6.8 (4.3-8.8)	127 (84-167)	162 (105-212)

Table S5. Proportional and absolute burden per 1M population of T2D incidence attributable to suboptimal diet* and each individual dietary factor† at the national level in 1990 and 2018

Country	Dietary factor	Proportional burden, %		Rate, per 1M population‡	
		1990	2018	1990	2018
	Sugar-sweetened beverages	8.3 (6.1-12.2)	5.7 (4-8.8)	154 (113-224)	137 (96-212)
	Potatoes	13.1 (8.1-20.4)	16 (10.1-23.8)	244 (149-377)	384 (240-574)
	Fruit	2.9 (2.7-3.1)	3 (2.8-3.3)	54 (49-58)	73 (66-80)
	Nuts and seeds	1.3 (1.2-1.4)	1.1 (0.9-1.2)	24 (22-26)	25 (21-30)
	Non-starchy vegetables	0.7 (0.6-0.8)	0.8 (0.7-0.9)	13 (11-16)	19 (16-23)
	Fruit juice	0.2 (0.1-0.3)	0.2 (0.1-0.3)	3 (2-6)	5 (3-8)
United Arab Emirates	Suboptimal diet	91.2 (84.8-95.7)	80.1 (69.7-89.2)	3207 (2940-3447)	5919 (5127-6719)
	Whole grains	22 (12.7-31.7)	28 (17.2-36.8)	777 (442-1118)	2057 (1275-2736)
	Refined grains (total)	20.1 (12.3-30.6)	28 (17.8-41.9)	709 (426-1087)	2064 (1324-3072)
	Refined rice	11 (6.5-17.9)	16.1 (9.7-25.3)	388 (229-632)	1186 (728-1873)
	Refined wheat	10.7 (6.3-17.3)	15.1 (9.1-23.5)	376 (219-613)	1113 (681-1760)
	Processed meats	59.2 (36.5-78.7)	41 (17.8-67.3)	2075 (1272-2786)	3025 (1322-5047)
	Unprocessed red meats	43.1 (30.9-54.8)	10.4 (6.2-19.2)	1514 (1092-1950)	769 (454-1424)
	Yogurt	8.1 (5.1-10.5)	8.3 (5.2-10.9)	282 (179-373)	616 (380-816)
	Sugar-sweetened beverages	18.1 (12.1-27.4)	12.6 (8-21.9)	635 (423-977)	937 (583-1625)
	Potatoes	23.1 (13.9-32.9)	15.4 (8.6-27.3)	810 (493-1162)	1142 (638-2037)
	Fruit	2.3 (2-2.7)	3 (2.7-3.3)	83 (71-94)	222 (193-252)
	Nuts and seeds	1 (0.8-1.2)	0.8 (0.5-1)	36 (28-43)	56 (39-77)
	Non-starchy vegetables	1 (0.8-1.1)	1.4 (1.2-1.6)	34 (28-41)	102 (87-120)
	Fruit juice	0.1 (0.1-0.2)	0.1 (0.1-0.3)	4 (2-8)	10 (5-20)
United Kingdom	Suboptimal diet	75.4 (72-78.9)	77.9 (74.1-81.8)	2390 (2255-2543)	4301 (4026-4576)
	Whole grains	21.3 (18.5-24.1)	21.1 (18-24.1)	675 (584-768)	1160 (992-1336)
	Refined grains (total)	6.9 (4.9-9.5)	13 (9.3-17.5)	219 (156-302)	718 (512-966)
	Refined rice	0.4 (0.3-0.6)	1.3 (0.9-1.9)	13 (9-18)	71 (50-104)
	Refined wheat	6.5 (4.6-9)	11.9 (8.4-16)	208 (147-287)	656 (466-882)
	Processed meats	36.7 (30-44.2)	44.3 (36.8-52.7)	1162 (951-1410)	2448 (2032-2922)
	Unprocessed red meats	18.1 (15.2-22.7)	18.4 (15.3-23.3)	574 (482-729)	1015 (836-1294)
	Yogurt	8.1 (6.1-9.7)	8.3 (6.3-10.2)	257 (193-310)	462 (351-561)
	Sugar-sweetened beverages	14.5 (12.2-18.6)	17 (14.3-21.6)	458 (387-595)	937 (792-1193)
	Potatoes	18.9 (15.9-23.6)	11.4 (9.4-15)	601 (499-751)	628 (515-832)
	Fruit	2.4 (2.2-2.5)	2.9 (2.7-3.1)	75 (70-81)	162 (149-175)
	Nuts and seeds	1 (0.9-1.1)	1.1 (1-1.2)	32 (29-35)	62 (56-68)
	Non-starchy vegetables	1 (0.9-1.1)	1.3 (1.2-1.4)	32 (29-35)	71 (64-78)
	Fruit juice	0.2 (0.1-0.2)	0.2 (0.1-0.2)	5 (4-6)	10 (8-13)
United States	Suboptimal diet	76.6 (73.7-80)	73.4 (70.1-77.9)	2778 (2639-2941)	4484 (4249-4809)

Table S5. Proportional and absolute burden per 1M population of T2D incidence attributable to suboptimal diet* and each individual dietary factor† at the national level in 1990 and 2018

Country	Dietary factor	Proportional burden, %		Rate, per 1M population‡	
		1990	2018	1990	2018
	Whole grains	33.3 (31.5-35)	31.5 (29.7-33.3)	1207 (1135-1284)	1923 (1801-2045)
	Refined grains (total)	10.8 (9.7-13.5)	10 (8.9-12)	392 (348-485)	609 (544-734)
	Refined rice	1.6 (1.4-2.2)	1.5 (1.3-1.8)	58 (51-79)	92 (82-109)
	Refined wheat	9.3 (8.3-11.6)	8.6 (7.6-10.4)	338 (301-415)	524 (468-637)
	Processed meats	31.7 (25.6-40.4)	33.3 (26.4-43.3)	1155 (931-1476)	2037 (1611-2664)
	Unprocessed red meats	19.7 (16.9-25.1)	14.6 (12.4-20.9)	717 (612-917)	891 (756-1283)
	Yogurt	11.9 (10.9-12.9)	11.3 (10.3-12.3)	433 (394-472)	688 (625-755)
	Sugar-sweetened beverages	20.8 (18.1-26.1)	16.4 (14.1-21.5)	758 (658-947)	999 (863-1313)
	Potatoes	3 (2.7-4)	2.6 (2.3-3.4)	108 (96-145)	158 (143-207)
	Fruit	2.7 (2.6-2.9)	2.9 (2.7-3.1)	99 (92-106)	178 (166-192)
	Nuts and seeds	1.2 (1.1-1.3)	0.9 (0.8-1)	42 (39-46)	54 (47-62)
	Non-starchy vegetables	1 (1-1.1)	1.1 (1-1.2)	38 (35-42)	69 (62-76)
	Fruit juice	0.4 (0.3-0.4)	0.4 (0.4-0.5)	13 (11-15)	25 (21-28)
Uruguay	Suboptimal diet	82 (76.3-87.7)	85.1 (79.1-90.6)	1095 (1011-1186)	2557 (2358-2747)
	Whole grains	23.9 (15.8-30)	25.3 (16.9-32)	319 (210-403)	760 (504-961)
	Refined grains (total)	18.7 (13.3-26.2)	22.8 (16.4-31.1)	251 (178-352)	685 (483-940)
	Refined rice	3.6 (2.5-5.6)	4.8 (3.3-7.3)	49 (34-75)	144 (97-220)
	Refined wheat	15.8 (11.1-22.4)	19.2 (13.6-26.6)	212 (150-299)	578 (403-800)
	Processed meats	31.5 (17.4-50.1)	45.1 (27.9-62.1)	421 (234-669)	1352 (835-1868)
	Unprocessed red meats	39.5 (29.7-48.3)	26.4 (18.9-34.8)	526 (394-647)	797 (574-1042)
	Yogurt	10.1 (8.8-11.1)	10.6 (9.1-11.8)	135 (118-150)	319 (271-355)
	Sugar-sweetened beverages	24.4 (17-34.6)	32.5 (22.9-44)	328 (227-465)	974 (689-1329)
	Potatoes	3.2 (1.7-6.1)	2.2 (1.1-4.7)	43 (23-81)	65 (34-139)
	Fruit	2.4 (2.2-2.5)	2.9 (2.7-3.1)	32 (29-34)	87 (80-95)
	Nuts and seeds	1 (0.9-1.1)	0.8 (0.6-1)	13 (11-15)	23 (17-29)
	Non-starchy vegetables	1.5 (1.4-1.6)	1 (0.9-1.2)	20 (19-22)	31 (27-35)
	Fruit juice	0.1 (0.1-0.3)	0.2 (0.1-0.3)	2 (1-3)	5 (3-9)
Uzbekistan	Suboptimal diet	84.9 (78.4-90.7)	90.4 (85.7-94.3)	1254 (1148-1365)	3554 (3333-3754)
	Whole grains	17.8 (10.9-25.8)	23.9 (16.1-31.3)	263 (161-381)	941 (635-1224)
	Refined grains (total)	29.9 (23.3-38.2)	21.3 (16.3-27.9)	442 (344-571)	839 (640-1099)
	Refined rice	4.3 (3.2-6.3)	1.2 (0.9-1.9)	64 (48-93)	48 (35-74)
	Refined wheat	26.7 (20.7-34.4)	20.3 (15.5-26.6)	396 (306-514)	800 (609-1052)
	Processed meats	53.2 (35.5-69.8)	54 (36.4-71.6)	788 (519-1038)	2128 (1432-2818)
	Unprocessed red meats	21.4 (16.3-27.9)	44.3 (36.4-52.6)	317 (240-415)	1746 (1424-2070)
	Yogurt	6.6 (3.9-9)	6.8 (4.2-9.2)	98 (57-133)	267 (165-363)

Table S5. Proportional and absolute burden per 1M population of T2D incidence attributable to suboptimal diet* and each individual dietary factor† at the national level in 1990 and 2018

Country	Dietary factor	Proportional burden, %		Rate, per 1M population‡	
		1990	2018	1990	2018
	Sugar-sweetened beverages	19 (13.5-26.9)	11.3 (7.7-16.9)	281 (198-399)	445 (300-664)
	Potatoes	7.9 (4.9-13.7)	28.6 (19.7-37.4)	118 (73-203)	1127 (780-1470)
	Fruit	3 (2.7-3.3)	3.3 (2.9-3.6)	45 (40-50)	129 (113-143)
	Nuts and seeds	1.4 (1.3-1.5)	1.4 (1.3-1.6)	21 (19-23)	57 (51-62)
	Non-starchy vegetables	1.1 (0.9-1.3)	0.6 (0.4-0.8)	16 (13-19)	23 (16-31)
	Fruit juice	0.1 (0.1-0.1)	0.1 (0-0.1)	1 (1-2)	3 (2-5)
Vanuatu	Suboptimal diet	68.1 (63.2-74)	68.8 (63.7-74.5)	2657 (2449-2904)	4715 (4318-5129)
	Whole grains	40 (35.7-42.5)	33.9 (26.7-39)	1559 (1381-1673)	2320 (1816-2670)
	Refined grains (total)	21.4 (13.7-30.4)	23.8 (15.6-34.1)	834 (534-1189)	1633 (1073-2328)
	Refined rice	17 (10.7-24.7)	16.3 (10.3-24)	663 (418-966)	1113 (710-1649)
	Refined wheat	5.7 (3.4-8.8)	9.7 (6-14.8)	222 (134-343)	665 (414-1014)
	Processed meats	13 (5.7-25.6)	7.5 (3.1-15.9)	506 (227-987)	512 (212-1084)
	Unprocessed red meats	20 (14.1-26.8)	21.5 (15.2-28.5)	780 (546-1042)	1471 (1041-1938)
	Yogurt	13.6 (12.5-14.6)	13.9 (12.9-14.9)	529 (482-571)	953 (870-1026)
	Sugar-sweetened beverages	5.7 (3.4-10.3)	10.4 (6.3-16)	222 (130-401)	710 (434-1094)
	Potatoes	0 (0-0)	2.1 (1.1-4.5)	0 (0-1)	147 (75-309)
	Fruit	3.6 (3.4-3.8)	4.1 (3.9-4.4)	140 (131-149)	284 (266-302)
	Nuts and seeds	1.2 (1-1.3)	1.4 (1.2-1.5)	46 (40-52)	94 (81-104)
	Non-starchy vegetables	1.6 (1.4-1.8)	1.9 (1.7-2.1)	62 (55-69)	128 (114-142)
	Fruit juice	0 (0-0.1)	0 (0-0.1)	1 (1-3)	3 (2-6)
Venezuela	Suboptimal diet	82.5 (75.9-89.3)	78.3 (71.7-84.9)	2658 (2417-2922)	4383 (3987-4806)
	Whole grains	28.6 (20.1-34.6)	28.7 (19.7-35.2)	923 (650-1121)	1609 (1107-1968)
	Refined grains (total)	13.8 (9.7-19.5)	25.7 (18.5-33.9)	447 (313-629)	1439 (1026-1908)
	Refined rice	4.9 (3.3-7.2)	17.2 (12-23.6)	157 (107-231)	964 (671-1322)
	Refined wheat	9.6 (6.6-13.7)	10.9 (7.5-15.3)	311 (213-444)	612 (420-864)
	Processed meats	45.4 (27.7-65.5)	36 (20.9-54.2)	1457 (897-2118)	2025 (1174-3044)
	Unprocessed red meats	23.6 (16.6-31.1)	17.2 (11.9-24.8)	761 (532-1007)	966 (662-1386)
	Yogurt	11.6 (10.1-12.6)	11.5 (10-12.7)	372 (325-411)	643 (560-718)
	Sugar-sweetened beverages	29 (22.2-37.3)	18.1 (12.8-24.5)	932 (714-1199)	1014 (715-1375)
	Potatoes	0.5 (0.3-1)	2.7 (1.5-5.2)	17 (9-33)	151 (80-294)
	Fruit	2.3 (1.7-2.7)	2.9 (2.3-3.4)	73 (55-89)	165 (130-193)
	Nuts and seeds	1.3 (1.2-1.4)	1.5 (1.3-1.6)	42 (39-46)	82 (74-90)
	Non-starchy vegetables	1.7 (1.6-1.9)	1.6 (1.3-1.9)	56 (50-62)	91 (74-108)
	Fruit juice	0.1 (0.1-0.2)	0.2 (0.1-0.3)	4 (3-7)	9 (5-16)
Vietnam	Suboptimal diet	59.6 (54-65.8)	61.3 (55.1-67.8)	1231 (1114-1369)	2423 (2171-2673)

Table S5. Proportional and absolute burden per 1M population of T2D incidence attributable to suboptimal diet* and each individual dietary factor† at the national level in 1990 and 2018

Country	Dietary factor	Proportional burden, %		Rate, per 1M population‡	
		1990	2018	1990	2018
	Whole grains	34.1 (29.3-37.1)	28.8 (22.4-33.6)	704 (600-774)	1137 (873-1333)
	Refined grains (total)	25.5 (17.7-35)	23.7 (16.7-32.8)	527 (369-724)	936 (657-1304)
	Refined rice	25.2 (17.5-34.7)	22.8 (16-31.6)	522 (365-717)	899 (629-1257)
	Refined wheat	0.3 (0.2-0.5)	1.2 (0.8-1.9)	7 (5-11)	48 (32-73)
	Processed meats	0.9 (0.3-3.1)	6.7 (2.8-14.5)	18 (7-64)	264 (111-570)
	Unprocessed red meats	15.8 (9.8-23.1)	18.1 (12.1-26.1)	326 (204-481)	717 (475-1030)
	Yogurt	12 (11.2-12.7)	12.1 (11.3-12.8)	247 (230-265)	475 (444-509)
	Sugar-sweetened beverages	3.8 (2.6-6)	4.6 (3.4-7)	79 (54-124)	182 (133-278)
	Potatoes	0.9 (0.5-1.8)	0.7 (0.4-1.5)	19 (10-38)	29 (14-60)
	Fruit	2.8 (2.6-2.9)	3 (2.8-3.2)	57 (53-61)	118 (110-128)
	Nuts and seeds	1.3 (1.2-1.4)	0.8 (0.6-1)	27 (25-28)	31 (24-38)
	Non-starchy vegetables	0.7 (0.6-0.8)	0.9 (0.7-1)	15 (12-17)	34 (28-39)
	Fruit juice	0 (0-0)	0 (0-0)	1 (0-1)	1 (1-2)
Yemen	Suboptimal diet	74.2 (67-82.5)	82.2 (74.3-89.6)	1615 (1451-1813)	2598 (2345-2846)
	Whole grains	36.9 (30.6-41)	32.1 (22.7-38.4)	806 (662-900)	1013 (718-1214)
	Refined grains (total)	14.8 (11.6-19.2)	24.4 (19.4-29.8)	324 (253-422)	773 (614-943)
	Refined rice	2.8 (2.2-4)	7.6 (5.9-10)	61 (47-88)	242 (188-314)
	Refined wheat	12.3 (9.7-16.3)	18.2 (14.3-22.5)	269 (209-356)	576 (453-714)
	Processed meats	13.4 (5.2-30.1)	26.6 (11.9-49.5)	294 (113-648)	841 (379-1571)
	Unprocessed red meats	5.9 (3.8-11.1)	8 (5.3-12.7)	129 (83-246)	253 (170-397)
	Yogurt	11.9 (10.7-13.1)	12 (10.8-13)	259 (234-288)	377 (340-414)
	Sugar-sweetened beverages	34.1 (20.4-52.4)	43.5 (27.1-61.7)	746 (443-1149)	1370 (851-1962)
	Potatoes	7.5 (4-14.2)	6.3 (3.4-11.6)	163 (87-308)	199 (107-363)
	Fruit	3.8 (3.6-4)	3.9 (3.7-4.2)	82 (77-89)	123 (115-132)
	Nuts and seeds	1.2 (1.1-1.4)	1.3 (1.1-1.5)	27 (24-31)	42 (36-48)
	Non-starchy vegetables	1.6 (1.4-1.7)	1.8 (1.7-2)	34 (31-38)	58 (53-64)
	Fruit juice	0.1 (0.1-0.1)	0.1 (0.1-0.2)	2 (2-3)	4 (3-5)
Zambia	Suboptimal diet	76.2 (70.1-82.8)	70.1 (64.9-77.6)	1393 (1278-1520)	1675 (1535-1856)
	Whole grains	18.4 (11.5-26)	23.3 (15.4-30.6)	336 (211-478)	555 (368-732)
	Refined grains (total)	19.1 (16.2-22.5)	19 (16.3-22.4)	349 (294-416)	454 (389-533)
	Refined rice	5 (4.2-6.3)	5.4 (4.5-6.7)	91 (75-115)	128 (107-159)
	Refined wheat	14.9 (12.5-17.7)	14.4 (12.3-17.2)	272 (227-326)	345 (293-410)
	Processed meats	15.4 (5.6-35.4)	15.9 (5.6-37.2)	281 (102-647)	380 (132-895)
	Unprocessed red meats	16.7 (13.5-20.6)	22.9 (19.1-27.6)	304 (245-379)	546 (456-660)
	Yogurt	12.3 (11.4-13)	13 (12.2-13.8)	226 (208-240)	311 (288-331)

Table S5. Proportional and absolute burden per 1M population of T2D incidence attributable to suboptimal diet* and each individual dietary factor† at the national level in 1990 and 2018

Country	Dietary factor	Proportional burden, %		Rate, per 1M population‡	
		1990	2018	1990	2018
	Sugar-sweetened beverages	43.6 (34.2-53.3)	18.3 (12.6-25.3)	799 (622-979)	437 (302-607)
	Potatoes	0.5 (0.3-1.1)	2.1 (1.1-3.9)	10 (5-20)	49 (27-93)
	Fruit	2.6 (2.5-2.8)	3.7 (3.5-3.8)	48 (45-52)	87 (82-93)
	Nuts and seeds	1.2 (1.1-1.3)	0.9 (0.7-1.1)	22 (20-24)	21 (17-26)
	Non-starchy vegetables	0.3 (0.2-0.4)	0.7 (0.6-0.8)	6 (4-7)	17 (14-19)
	Fruit juice	0.1 (0.1-0.1)	0.1 (0.1-0.2)	2 (1-2)	3 (2-4)
Zimbabwe	Suboptimal diet	51.7 (46.5-58)	66.5 (60.3-73.3)	1166 (1044-1318)	2670 (2399-2948)
	Whole grains	3.9 (2.4-6.3)	4.2 (2.5-6.6)	88 (53-142)	166 (101-266)
	Refined grains (total)	14.6 (12.3-17.6)	15.1 (12.8-17.8)	329 (277-399)	603 (513-715)
	Refined rice	1.2 (1-1.6)	4.8 (4-6.1)	27 (23-35)	192 (161-244)
	Refined wheat	13.5 (11.4-16.3)	10.8 (9.1-12.9)	305 (257-371)	432 (365-517)
	Processed meats	6.4 (2-16.1)	9.6 (3.5-22.2)	144 (44-363)	382 (141-889)
	Unprocessed red meats	13.5 (10.5-17.1)	13.7 (10.7-17.1)	305 (235-389)	549 (428-687)
	Yogurt	10.1 (8.6-11.3)	10.8 (9.4-12)	227 (194-257)	435 (377-482)
	Sugar-sweetened beverages	13.9 (9.3-19.8)	35.2 (25.8-45.5)	314 (208-443)	1412 (1035-1818)
	Potatoes	2.5 (1.2-5.6)	3.8 (1.8-8.6)	57 (27-126)	154 (73-346)
	Fruit	2.7 (2.5-2.8)	3.2 (3-3.4)	60 (57-64)	126 (118-135)
	Nuts and seeds	1 (0.8-1.1)	1.3 (1.2-1.4)	22 (19-24)	52 (47-57)
	Non-starchy vegetables	0.9 (0.8-1)	1.1 (1-1.2)	21 (19-23)	46 (41-50)
	Fruit juice	0.1 (0.1-0.2)	0.2 (0.1-0.2)	3 (2-5)	6 7 (5-10)

* Represents the estimated proportional and absolute burden per 1M population of T2D incidence due to suboptimal intake of 11 dietary factors jointly: insufficient intake of whole grains, yogurt, fruit, nuts and seeds, and non-starchy vegetables; and excess intake of refined rice and wheat, processed meats, unprocessed red meat, sugar-sweetened beverages, potatoes, and fruit juice. The burden due to suboptimal diet was estimated using proportional multiplication, assuming that half the benefit of whole grains intake is mediated through replacement of refined rice and wheat intake. We did not truncate any estimates. Estimates with high proportional attributable burdens (e.g., over 90% total) should be interpreted cautiously.

† Proportional and absolute burden per 1M population of T2D for each risk factor (direct and BMI-mediated effects jointly, where relevant) individually. Refined rice and refined wheat modelled and reported separately, and their joint effects reported as refined grains (total).

‡ The absolute burden per 1 million population was calculated by dividing the absolute number of diet-attributable cases by the country population in that year and multiplying by 1 million.
