# Supplementary material for: Incident type 2 diabetes attributable to suboptimal diet in 184 countries
Source: Nat Med. 2023 Apr 17;29(4):982–95. doi: 10.1038/s41591-023-02278-8 (PMC10115653; doi:10.1038/s41591-023-02278-8)
Supplement: Supplementary file 2 — Reporting Summary [file 41591_2023_2278_MOESM2_ESM.pdf]

nature portfolio

Corresponding author(s): Meghan O'Hearn

Last updated by author(s): 02/24/2023

Reporting Summary

Nature Portfolio wishes to improve the reproducibility of the work that we publish. This form provides structure for consistency and transparency in reporting. For further information on Nature Portfolio policies, see our Editorial Policies and the Editorial Policy Checklist.

Statistics

For all statistical analyses, confirm that the following items are present in the figure legend, table legend, main text, or Methods section.

n/a	Confirmed	
☐	☒	The exact sample size (*n*) for each experimental group/condition, given as a discrete number and unit of measurement
☒	☐	A statement on whether measurements were taken from distinct samples or whether the same sample was measured repeatedly
☐	☒	The statistical test(s) used AND whether they are one- or two-sided<br>*Only common tests should be described solely by name; describe more complex techniques in the Methods section.*
☒	☐	A description of all covariates tested
☐	☒	A description of any assumptions or corrections, such as tests of normality and adjustment for multiple comparisons
☐	☒	A full description of the statistical parameters including central tendency (e.g. means) or other basic estimates (e.g. regression coefficient) AND variation (e.g. standard deviation) or associated estimates of uncertainty (e.g. confidence intervals)
☐	☒	For null hypothesis testing, the test statistic (e.g. *F*, *t*, *r*) with confidence intervals, effect sizes, degrees of freedom and *P* value noted<br>*Give P values as exact values whenever suitable.*
☒	☐	For Bayesian analysis, information on the choice of priors and Markov chain Monte Carlo settings
☒	☐	For hierarchical and complex designs, identification of the appropriate level for tests and full reporting of outcomes
☐	☒	Estimates of effect sizes (e.g. Cohen's *d*, Pearson's *r*), indicating how they were calculated

Our web collection on statistics for biologists contains articles on many of the points above.

Software and code

Policy information about availability of computer code

Data collection

No software was used for data collection for this modeling analysis.

Data analysis

ustom code was developed using in R (Version 4.0.0) with two-tailed alpha=0.05, for cleaning, merging, and formatting of all data inputs; calculation of age-adjusted relative risks; comparative risk assessment modeling, including population attributable fraction (PAF) calculation for each dietary factor separately and joint PAF calculation for all dietary factors; summary aggregation of stratum-level PAF estimates at the global, regional, nationals; and data visualizations. Given their computational size and complexity, all comparative risk assessment modeling codes were run on the Tufts University High Performance Computing Cluster (https://it.tufts.edu/high-performance-computing), supported by the National Science Foundation (grant:2018149) under active development by Research Technology, Tufts Technology Services.

The statistical code used for this analysis is not publicly available. The Global Dietary Database can make the statistical code available to researchers upon request. Eligibility criteria for such requests include: utilization for nonprofit purposes only, for appropriate scientific use based on a robust research plan, and by investigators from an academic institution. GDD will nominate co-authors to be included on any papers generated using GDD-generated statistical code. If you are interested in requesting access to the statistical code, please submit the following documents:
1. Proposed Research Plan: Please download and complete the Proposed Research plan form.
2. Data Sharing Agreement: Please download this form and complete the highlighted fields, have someone who is authorized to enter your institution into a binding legal agreement with outside institutions sign the document. Note: this agreement does not apply when Protected Health Information or Personally Identifiable Information are shared.
3. Email items 1 and 2 to info@globaldietarydatabase.org. Please use the subject line "GDD Code Access Request".

Once all documents have been received, the GDD team will be in contact with you regarding subsequent steps.

For manuscripts utilizing custom algorithms or software that are central to the research but not yet described in published literature, software must be made available to editors and reviewers. We strongly encourage code deposition in a community repository (e.g. GitHub). See the Nature Portfolio guidelines for submitting code & software for further information.

Data

Policy information about availability of data

All manuscripts must include a data availability statement. This statement should provide the following information, where applicable:

- Accession codes, unique identifiers, or web links for publicly available datasets
- A description of any restrictions on data availability
- For clinical datasets or third party data, please ensure that the statement adheres to our policy

All data used in this analysis is publicly available from the following sources:
(1) Individual dietary intake estimate distribution data [Global Dietary Database (GDD)- Download 2018 Final Estimates: https://www.globaldietarydatabase.org/data-download]
(2) Stratum-specific global mean BMI, converted to overweight and underweight prevalence distribution data [NCD Risk Factor Collaboration (NCD-RisC) - Data Downloads: https://ncdrisc.org/data-downloads.html]
(3) T2D burden incidence estimate distribution data [Global Health Data Exchange - Global Burden of Disease Study 2019 (GBD 2019) Results Tool: https://vizhub.healthdata.org/gbd-results/]
(4) Linear, BMI-stratified effects of dietary factors on weight gain or weight loss: Mozaffarian, D, Hao, T, Rimm, EB, Willett, WC, & Hu, FB. Changes in diet and lifestyle and long-term weight gain in women and men. N Engl J Med. 2011; 364(25): 2392-2404. doi:10.1056/NEJMoa1014296
(5) Direct, proportional, age-adjusted effects of BMI on T2D: Singh, GM, Danaei, G, Farzadfar, F, et al. The Age-Specific Quantitative Effects of Metabolic Risk Factors on Cardiovascular Diseases and Diabetes: A Pooled Analysis. PLoS ONE. 2013; 8(7): e65174. doi:10.1371/journal.pone.0065174
(6) Direct, proportional, aged-adjusted effects of diet on T2D:
a) whole grains: Reynolds, A, Mann, J, Cummings, J, Winter, N, Mete, E, & Te Morenga, L. Carbohydrate quality and human health: a series of systematic reviews and meta-analyses. Lancet (London, England). 2019; 393(10170): 434-445. doi:10.1016/s0140-6736(18)31809-9
b) all remaining dietary factors: Miller, V, Micha, R, Choi, E, Karageorgou, D, Webb, P, & Mozaffarian, D. Evaluation of the Quality of Evidence of the Association of Foods and Nutrients With Cardiovascular Disease and Diabetes: A Systematic Review. JAMA Network Open. 2022; 5(2): e2146705-e2146705. doi:10.1001/jamanetworkopen.2021.46705
doi:10.1371/journal.pone.0065174
(7) Optimal intake levels for dietary factors: Micha, R, Shulkin, ML, Penalvo, JL, et al. Etiologic effects and optimal intakes of foods and nutrients for risk of cardiovascular diseases and diabetes: Systematic reviews and meta-analyses from the Nutrition and Chronic Diseases Expert Group (NutriCoDE). PLoS ONE. 2017; 12(4): e0175149. doi:10.1371/journal.pone.0175149
(8) Population demographic data: UN population Division (age, sex, urbanicity); Barro, R, & Lee,
J. A New Data Set of Educational Attainment in the World, 1950-2010. Journal of
Developmental Economics. 2013; 104: 184-198
(9) Socio-demographic Index (SDI) data Global Health Data Exchange: Global Burden of Disease Study 2019 (GBD 2019) Socio-Demographic Index (SDI) 1950-2019 - https://ghdx.healthdata.org/record/ihme-data/gbd-2019-socio-demographic-index-sdi-1950-2019.
(10) FAO Food Balance Sheet data for the energy availability of "wheat and products" and "rice and products"(kcal/capita/day): United Nations Food and Agriculture Organization: Food Availability Data - http://www.fao.org/faostat/en/#home
(11) Global glycemic load estimates for wheat and rice products: Atkinson, FS, Foster-Powell, K, & Brand-Miller, JC. International tables of glycemic index and glycemic load values: 2008. Diabetes Care. 2008; 31(12): 2281-2283. doi:10.2337/dc08-1239
(12) Caloric content per 100 g for wheat and rice products: U.S. Department of Agriculture Agricultural Research Service Food and Nutrient Database for Dietary Studies 2017-2018: www.ars.usda.gov/nea/bnrc/fsrg

Human research participants

Policy information about studies involving human research participants and Sex and Gender in Research.

Reporting on sex and gender	This study involved secondary data analysis from publicly available databases. No human research participants were involved in this original analysis.
Population characteristics	NA
Recruitment	NA
Ethics oversight	NA

Note that full information on the approval of the study protocol must also be provided in the manuscript.

Field-specific reporting

Please select the one below that is the best fit for your research. If you are not sure, read the appropriate sections before making your selection.

☐ Life sciences ☒ Behavioural & social sciences ☐ Ecological, evolutionary & environmental sciences

For a reference copy of the document with all sections, see nature.com/documents/nr-reporting-summary-flat.pdf

Behavioural & social sciences study design

All studies must disclose on these points even when the disclosure is negative.

Study description	Quantitative, comparative risk assessment (CRA) modeling analysis incorporating independently derived inputs and parameters on demographics, dietary risk factors, their etiologic effects, and type II diabetes incidence to model attributable burdens at the global, regional, and national levels, and by key sociodemographic factors.
Research sample	Global adult population (20+ years): 35,328 population strata across 184 countries in each year (1990 and 2018)
Sampling strategy	We incorporated dietary data from the Global Dietary Database (GDD), population demographics from the United Nations, adiposity and diabetes distributions from the NCD Risk Factor Collaboration and Global Burden of Disease (GBD) study, direct and BMI-mediated etiologic effects of dietary factors on T2D from pooled multivariable-adjusted analyses, and optimal dietary intakes from published sources into a comparative risk assessment modeling framework to estimate the impact of 11 dietary factors, separately and jointly, on the absolute and proportional burdens of new T2D cases globally.
Data collection	The data sources are all published and publicly available. In brief, estimates of dietary intake, T2D incidence, and overweight/obesity prevalence were sourced from Bayesian hierarchical prediction models, incorporating nationally representative, stratified survey data on the respective outcome (i.e., dietary intake, T2D incidence, etc.). Etiologic effects were extracted from meta-analyses of randomized control trials and prospective cohort studies as well as pooled prospective cohort studies.
Timing	1990 and 2018
Data exclusions	3 countries excluded from GDD prediction models due to lack of FAO food availability covariate data; and 1 additional country was excluded due to lack of T2D incidence data in the Global Burden of Disease study.
Non-participation	NA, only applicable for RCTs and cohort studies
Randomization	NA, only applicable for RCTs

Reporting for specific materials, systems and methods

We require information from authors about some types of materials, experimental systems and methods used in many studies. Here, indicate whether each material, system or method listed is relevant to your study. If you are not sure if a list item applies to your research, read the appropriate section before selecting a response.

Materials & experimental systems

n/a	Involved in the study	
☒	☐	Antibodies
☒	☐	Eukaryotic cell lines
☒	☐	Palaeontology and archaeology
☒	☐	Animals and other organisms
☒	☐	Clinical data
☒	☐	Dual use research of concern

Methods

n/a	Involved in the study	
☒	☐	ChIP-seq
☒	☐	Flow cytometry
☒	☐	MRI-based neuroimaging
